# Age-Related Differential Structural and Transcriptomic Responses in the Hypertensive Heart

**DOI:** 10.3389/fphys.2018.00817

**Published:** 2018-07-09

**Authors:** Francine Z. Marques, Po-Yin Chu, Mark Ziemann, Antony Kaspi, Helen Kiriazis, Xiao-Jun Du, Assam El-Osta, David M. Kaye

**Affiliations:** ^1^Heart Failure Research Group, Baker Heart and Diabetes Institute, Melbourne, VIC, Australia; ^2^Department of Pharmacology, Faculty of Medicine Nursing and Health Sciences, Monash University, Melbourne, VIC, Australia; ^3^Epigenetics in Human Health and Disease, Department of Diabetes, Monash University, Melbourne, VIC, Australia; ^4^Experimental Cardiology Laboratory, Baker Heart and Diabetes Institute, Melbourne, VIC, Australia; ^5^Central Clinical School, Faculty of Medicine Nursing and Health Sciences, Monash University, Melbourne, VIC, Australia; ^6^Hong Kong Institute of Diabetes and Obesity, Prince of Wales Hospital, The Chinese University of Hong Kong, Hong Kong, Hong Kong; ^7^Department of Pathology, The University of Melbourne, Melbourne, VIC, Australia; ^8^Heart Centre, Alfred Hospital, Melbourne, VIC, Australia

**Keywords:** heart failure, aging, fibrosis, microRNAs, miRNAs, miRs, transcriptome, pathways

## Abstract

While aging is a critical risk factor for heart failure, it remains uncertain whether the aging heart responds differentially to a hypertensive stimuli. Here we investigated phenotypic and transcriptomic differences between the young and aging heart using a mineralocorticoid-excess model of hypertension. Ten-week (“young”) and 36-week (“aging”) mice underwent a unilateral uninephrectomy with deoxycorticosterone acetate (DOCA) pellet implantation (*n* = 6–8/group) and were followed for 6 weeks. Cardiac structure and function, blood pressure (BP) and the cardiac transcriptome were subsequently examined. Young and aging DOCA mice had high BP, increased cardiac mass, cardiac hypertrophy, and fibrosis. Left ventricular end-diastolic pressure increased in aging DOCA-treated mice in contrast to young DOCA mice. Interstitial and perivascular fibrosis occurred in response to DOCA, but perivascular fibrosis was greater in aging mice. Transcriptomic analysis showed that young mice had features of higher oxidative stress, likely due to activation of the respiratory electron transport chain. In contrast, aging mice showed up-regulation of collagen formation in association with activation of innate immunity together with markers of inflammation including cytokine and platelet signaling. In comparison to younger mice, aging mice demonstrated different phenotypic and molecular responses to hypertensive stress. These findings have potential implications for the pathogenesis of age-related forms of cardiovascular disease, particularly heart failure.

## Introduction

One of the major and most inevitable risk factor for the development of heart failure is aging, particularly when combined with hypertension (Owan et al., 2006). With improvements in lifespan, the prevalence of heart failure has increased in the past decade, and is estimated to rise by 46% by 2030 (Benjamin et al., 2017). Uncontrolled high-blood pressure (BP), also known as hypertension, leads to cardiac hypertrophy, systolic and diastolic dysfunction, progressing into heart failure. Each increment of 20 mmHg in systolic BP increases the risk of heart failure by 56% (Haider et al., 2003). The prevalence of heart failure particularly with preserved ejection fraction (HFPEF) is projected to rise significantly over the coming decade (Dunlay et al., 2017). In United States, it is projected that the prevalence of heart failure will increase by 25% from 2015 to 2030, with a concomitant 2–3 fold rise in healthcare costs (Heidenreich et al., 2011). While the role of aging *per se* as a cause of heart failure is unclear, the addition of risk factors such as hypertension is well-known to be involved in the development of the disease (Dunlay et al., 2017).

Here we used experimental models to better understand the pathophysiology of the combination of two key clinical determinants of HFPEF, hypertension, and aging to identify mechanisms involved. In this context, we hypothesized that the use of young animals in experimental models might yield different results when compared to aging animals, which do not resemble hypertensive heart failure in humans. Therefore, the aim of the present study was to characterize the cardiac phenotype and transcriptome of young and aging mice undergoing hypertension due to a mineralocorticoid-excess, to determine the molecular mechanisms involved in each age group and to identify which one is more relevant for translational studies.

## Materials and Methods

### Animal Model and Treatment

Male C57Bl/6 mice at either 10- (“young”) or 36-weeks of age (“aging”) underwent a left unilateral uninephrectomy and were randomly allocated to have a slow-release 21 days deoxycorticosterone acetate (DOCA; Innovative Research of America) or a placebo pellet implanted in the right flank while anesthetized with isoflurane (*n* = 6–8 per group). After 21 days, an additional pellet was implanted in the left flank for the remainder of the experiment for a total of 6 weeks, when mice were euthanized at 16- or 42-weeks of age. All animals were provided 1% saline and had access to food and water *ad libitum* during the course of the study. Animals were monitored and weighed regularly over the protocol. All experimental protocols were approved by the Alfred Medical Research and Education Precinct Animal Experimentation Ethics Committee under the guidelines of the National Medical and Health Research Council of Australia. Echocardiography, cardiac catheter, RNA extraction, and library preparation and their analyses were performed blindly. No animals were excluded from the study.

### Functional Measurements

The day prior to the completion of the study, mice were anesthetized using isoflurane and echocardiography was performed to image the left ventricle using a PHILIPS IE33 ultrasound machine (Royal Philips Electronics, Amsterdam, Netherlands) with a 15-MHz linear transducer. Images were analyzed blind. Immediately prior to the completion of the study, mice were anesthetized with isoflurane and a 1.4F microtipped transducer catheter (Millar, Houston, TX, United States) was used to measure arterial BP and determine left ventricular end-diastolic pressure (LVEDP).

### Morphological Analyses

Following cardiac catheter, a blood sample, the heart, kidney, and lung were rapidly removed. The tissues were weighed, before being snap frozen in liquid nitrogen and stored at -80°C or fixed in 10% formalin.

### Histological Analyses

Paraffin sections were cut in 4 μm sections and stained with Masson’s trichrome in order to analyze the collagen present in heart samples. Perivascular and interstitial fibrosis levels were quantified in the heart in 10 random fields of view per section using an Olympus BH2 microscope (400× magnification) and ImagePro Plus software (Adept Electronic Solutions, Pty Ltd., Moorabbin, VIC, Australia). Collagen levels were expressed as a percentage of the area of the region of interest.

### Statistical Analyses

GraphPad Prism (version 6) package was used for the statistical analysis and graphing. Normal distribution of data was verified using Shapiro–Wilk’s normality test. Two-factor analysis of variance (ANOVA; with Benjamini and Hochberg’s false discovery rate adjustment for multiple comparisons – not for repeated measures) was used to compare the data between the disease models (sham and DOCA) and age (young and aging). Values are presented as mean ± SEM, and those with a *P* (or *q* for multiple comparisons when there were more than two groups) <0.05 were considered significant.

### Cardiac Transcriptome

To understand the molecular mechanisms involved in the increase in cardiac fibrosis and worsening of the DOCA phenotype with aging, we examined the cardiac transcriptome between young and aging sham and DOCA (*n* = 6 young sham, others *n* = 4 per group). Tissue was collected as described above. RNA was extracted using the RNeasy (Qiagen) kit, and DNase treated. RNA quality was assessed on MultiNA bioanalyzer (Shimadzu). A total of 200 ng RNA underwent ribosomal RNA (rRNA) depletion using the NEBNext^®^ rRNA Depletion Kit followed by library construction using NEBNext^®^ Ultra^TM^ Directional RNA Library Prep Kit for Illumina^®^ (both from NEB). Library QC was performed by MultiNA Bioanalyzer (Shimadzu) and then pooled to equimolar concentration. Pooled libraries underwent Illumina single read sequencing at the Australian Genome Research Facility (AGRF, Melbourne, VIC, Australia) using HiSeq v4 reagents to generate 100 bp reads.

### Bioinformatic Analyses

Reads were trimmed for quality using a minimum phred value of 20 and minimum length of 18 using FastX Toolkit (Cox and Mann, 2012). Reads were mapped with STAR (Li et al., 2014) to the mouse genome downloaded from Ensembl (GRCm38). rRNA carryover was quantified by mapping reads to 5S, 18S, 28S, and Rs5-8s1 sequenced with BWA aln (Houweling et al., 2005). FeatureCounts (Zhang and Kass, 2011) was used to count reads mapped to gene bodies on the correct strand with a minimum mapping quality of 20 using Ensembl genome annotation (Mus musculus.GRCm38.85.gtf). Genes with <10 reads per sample on average were excluded prior to differential analysis with the EdgeR package (Stec et al., 2000) to determine the contrast between groups (young sham vs. DOCA and aging sham vs. DOCA). EdgeR FDR < 0.05 is considered statistically significant. Mean difference plots were generated in R. Genes were ranked from most up-regulated to most down-regulated by multiplying the sign of the log2 fold change by the inverse of the *P*-value. This preranked list was used for pathway analysis using gene-set enrichment analysis (GSEA) (Glasscock et al., 2015). Gene sets used included REACTOME (Reed et al., 2011), Molecular Signatures Database (MSigDB) (Koch et al., 2013), and Encode TFBS we have used previously (Forman et al., 1997). GSEA FDR < 0.05 is considered statistically significant.

### Gene Set Heat Map

Gene-set enrichment analysis is useful to determine whether a gene set is up- or down-regulated, but it lacks the ability to visualize intragroup variability. We developed Gsheat, which scores gene set activation in each sample. Starting from raw counts, genes are ranked from least expressed (smallest number) to most expressed (biggest number). Gsheat calculates the sum of all the ranks for all member genes in each sample for each gene set. This score is then used to generate a matrix with gene sets as rows and samples as columns Limma package (Ritchie et al., 2015) is used to determine whether the gene sets are differentially expressed between groups. The top gene sets by significance are extracted and used to generate a heat map, with bright yellow indicating high expression and dark red low expression. The results were uploaded into Enrichment Map (Hacker et al., 2006), a plugin of Cytoscape (v3.4.0) (Yang et al., 2008), to build pathway networks.

### Multi-Contrast Enrichment

It is common to perform pathway analysis with a single dimension, but we are also interested in gene set regulation in multidimensional comparisons. In this study, we are looking at gene sets that are altered in two contrasts: young sham vs. young DOCA against aging sham vs. aging DOCA. To achieve this, we first rank genes in each contrast and paste these data side by side. Next, we calculate enrichment score(s) for each gene set in two dimensions as described previously (Cox and Mann, 2012). Gene sets were additionally sourced from the Starbase project webpage to investigate microRNA (miRNA) targets (Li et al., 2014).

## Results

### Hemodynamics

**FIGURE 1:**
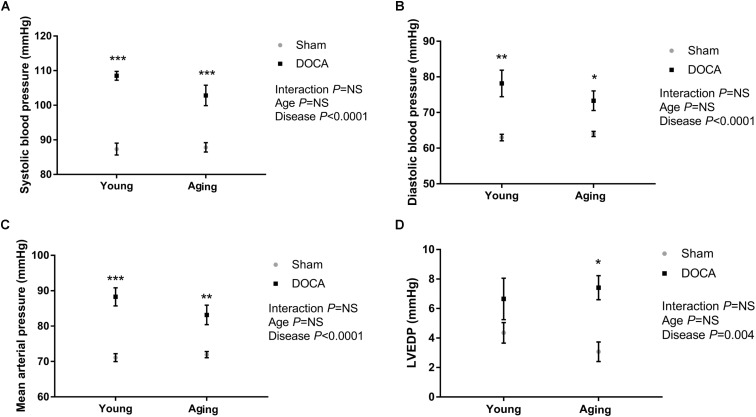
Blood pressure (BP) and left ventricular end-diastolic pressure (LVEDP) of young and aging sham and DOCA mice. **(A)** Systolic BP and **(B)** diastolic BP were higher in young and aging DOCA compared to sham mice. **(C)** Mean arterial pressure was only significantly higher in young DOCA compared to sham mice. **(D)** LVEDP was only significantly higher in aging DOCA compared to sham mice. Values are mean ± SEM. ^∗^*q* < 0.05, ^∗∗^*q* < 0.01, ^∗∗∗^*q* < 0.001 DOCA vs. sham control. Sample size: aging DOCA *n* = 8, all other groups *n* = 6.

As expected, DOCA administration led to a significant increase in systolic (**Figure 1A**) and diastolic BP (**Figure 1B**) independent of age. DOCA was the only determinant of the increase in BP, accounting for 78% of high-systolic BP and 54% of high-diastolic BP. Mean arterial pressure (MAP) was significantly higher in young and aged DOCA mice compared to age-matched sham mice (**Figure 1C**). Administration of DOCA only led to a significant increase in LVEDP in aging mice (*q* = 0.02, **Figure 1D**), but there was no difference between young and aging DOCA mice (*q* = 0.57).

### Cardiac Remodeling

Young DOCA-treated mice had increased heart:body weight ratio compared to age-matched sham mice (mean ± SEM: sham 5.1 ± 0.2 mg/g vs. DOCA 6.6 ± 0.2 mg/g, *P* = 0.005), while no difference was observed between aging sham vs. DOCA mice (sham 4.9 ± 0.1 mg/g vs. DOCA 5.4 ± 0.4 mg/g, *P* = 0.47). Both young and aging DOCA mice had higher kidney:body weight ratio compared to age-matched controls (young sham 9.6 ± 0.7 mg/g vs. DOCA 16.6 ± 0.3 mg/g, *P* < 0.0001; aging sham 9.13 ± 0.3 mg/g vs. DOCA 12 ± 0.8 mg/g, *P* = 0.005), but young DOCA had a significantly higher ratio than aging DOCA mice (*P* < 0.0001). There was no difference in lung:body weight ratio in either age or disease groups (data not shown).

Independent of age, DOCA mice displayed echocardiographic features of cardiac hypertrophy. This included an increase in the left ventricular posterior wall dimension at diastole (LVPWd) compared to age-matched sham mice (**Table 1**). The LVPWd was significantly greater in the context of both age and disease response as indicated by the interaction term (**Table 1**). Intraventricular septum in systole (IVSs) was increased in aged DOCA only (**Table 1**).

**Table 1:** Serial echocardiographic examination in sham and DOCA mice between young and aging groups.

	Young		Aging		Two-way ANOVA %, *P*-value		
	**Sham**	**DOCA**	**Sham**	**DOCA**	**Interaction**	**Age**	**Disease**
				
IVSd (mm)	0.69 ± 0.03	1.02 ± 0.05	0.67 ± 0.03	1.01 ± 0.03	0.004%, *P* = 0.95	0.33%, *P* = 0.56	80.6%, *P* < 0.0001
IVSs (mm)	1.14 ± 0.03	1.31 ± 0.04	1.04 ± 0.03	1.37 ± 0.04^∗^	12.8%, *P* = 0.052	3.8%, *P* = 0.27	26.2%, *P* = 0.0078
LVIDd (mm)	3.94 ± 0.07	3.72 ± 0.16	3.85 ± 0.05	3.66 ± 0.13	0.04%, *P* = 0.93	1.7%, *P* = 0.53	12.3%, *P* = 0.10
LVIDs (mm)	2.55 ± 0.07	2.48 ± 0.16	2.68 ± 0.04	2.41 ± 0.17	2.5%, *P* = 0.46	0.3%, *P* = 0.81	7.5%, *P* = 0.21
LVPWd (mm)	0.72 ± 0.03	0.85 ± 0.04^∗^	0.73 ± 0.02	0.88 ± 0.01^∗∗^	1.7%, *P* = 0.40	2.6%, *P* = 0.31	46%, *P* < 0.0001
LVPWs (mm)	1.12 ± 0.04	1.13 ± 0.03	0.96 ± 0.04	1.21 ± 0.05^∗∗^	19.4%, *P* = 0.017	2.0%, *P* = 0.41	24.1%, *P* = 0.0086
Fractional shortening (%)	35% ± 2	34% ± 3	30% ± 2	35% ± 3	5.5%, *P* = 0.28	3.0%, *P* = 0.42	1.6%, *P* = 0.56


*All results are shown as mean ± SEM. Sample size: *n* = 6 per group for all groups besides aging DOCA (*n* = 7). Sham vs. DOCA. ^∗^*q* < 0.05, ^∗∗^*q* < 0.01. LVIDs, left ventricular internal dimension systolic; LVIDd, left ventricular internal dimension diastolic; FS, fractional shortening; LVPWd, left ventricular posterior wall dimension at diastole; LVPWs, left ventricular posterior wall dimension at systole; IVSd, interventricular septal dimension at diastole; IVSs, interventricular septal dimension at systole.*

**FIGURE 2:**
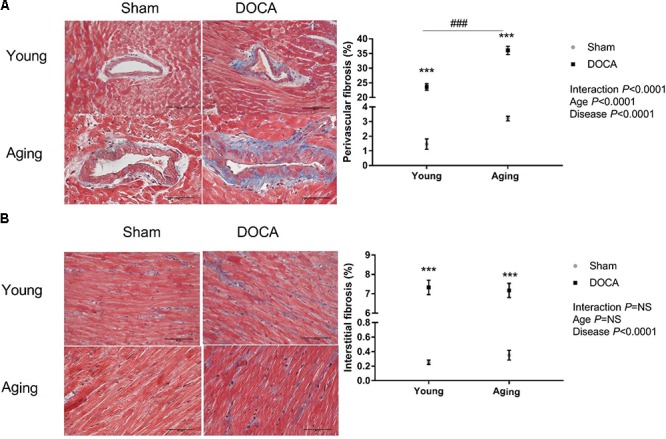
Cardiac fibrosis of young and aging sham and DOCA mice. **(A)** Young and aging DOCA mice had higher perivascular and **(B)** interstitial fibrosis than sham mice. However, aging mice had more perivascular fibrosis than young mice. Values are mean ± SEM. ^∗^*q* < 0.05, ^∗∗^*q* < 0.01, ^∗∗∗^*q* < 0.001 DOCA vs. sham control. ^###^*P* < 0.001 young vs. aging DOCA. Scale bar: 50 μm. Sample size: aging DOCA *n* = 8, all other groups *n* = 6.

Consistent with prior studies, DOCA treated mice developed extensive perivascular and interstitial cardiac fibrosis (**Figures 2A,B**). The perivascular collagen volume fraction was 23.6 ± 1.1% (mean ± SEM) in young DOCA, while this was significantly greater in aging DOCA mice at 36.1 ± 1.4% (*P* < 0.0001). In contrast, the extent of interstitial fibrosis was 7.33 ± 0.37% in young DOCA mice and 7.17 ± 0.37% in aging DOCA mice and the difference was not significantly difference (*q* = 0.818).

### Transcriptomic Analyses

The Supplementary Table S1 summarizes the quality metrics supplied by STAR. On average, the read length was 99 nt. Uniquely mapped read count was high at >80% for all samples except one sample (14AH2), which contained a higher proportion of rRNA reads and belongs to the young DOCA group. We then assigned the reads to genes (Supplementary Table S2). Prior to differential expression analysis, we perform a filtering to remove genes that have fewer than 10 reads on average. From 48,526 genes we initially began with, we are left with 14,410 after filtering.

### Young Sham vs. DOCA

**FIGURE 3:**
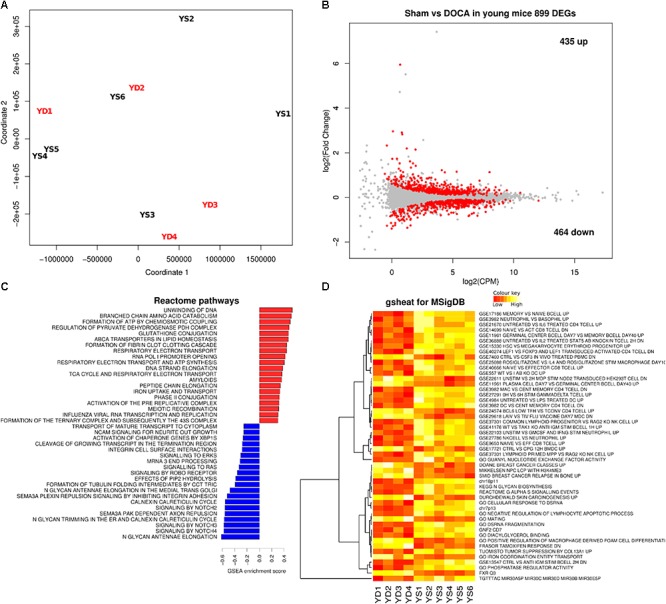
Cardiac transcriptome in young sham vs. DOCA mice. **(A)** Principal component analyses shows lack of sample group clustering on the first two dimensions for young sham vs. DOCA mice. **(B)** MA plot showing 899 differentially expressed genes (FDR < 0.05; Supplementary Table S3). Mechanisms differentially regulated between young sham and DOCA mice in the **(C)** GSEA analysis of Reactome Database and **(D)** Gsheat analysis of Molecular Signatures Database (MSigDB) (Supplementary Table S4). Sample size: *n* = 6 young sham vs. *n* = 4 young DOCA mice. CPM, counts per million; YD, young DOCA; YS, young sham.

We next compared the cardiac transcriptome of young sham vs. DOCA mice. While the principal component analyses (PCA) did not show a clear cluster on the first two principle components (**Figure 3A**), there were 899 differentially expressed genes (FDR < 0.05), of which 435 were up- and 464 were down-regulated (**Figure 3B** and Supplementary Table S3). This included up-regulation of genes previously associated with heart failure including genes for the natriuretic peptide A (*Nppa*) (Houweling et al., 2005), phosphodiesterase 5A (*Pde5a*) (Zhang and Kass, 2011), and fibrinogen (*Fgb*) (Stec et al., 2000). Pathways related to respiratory electron chain were up-regulated, while those related to immune system, cytokine and platelet signaling, and nerve growth factor were down-regulated (**Figures 3C,D** and Supplementary Table S4). Moreover, the unsupervised heat map analysis of the MSigDB supports different clustering between young sham and DOCA mice (**Figure 3D**).

### Aging Sham vs. DOCA

**FIGURE 4:**
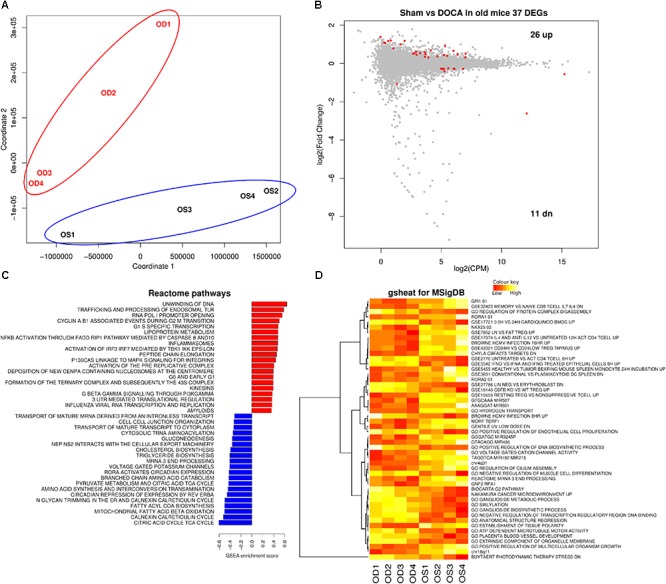
Cardiac transcriptome in aging sham vs. DOCA mice. **(A)** Principal component analysis confirmed that aging sham and age-matched DOCA mice formed two distinctive groups. **(B)** MA plot showing 37 genes were differentially expressed between the two groups (FDR < 0.05; Supplementary Table S5). Mechanisms differentially regulated between aging sham and DOCA mice in the **(C)** GSEA analysis of Reactome Database and **(D)** Gsheat analysis of Molecular Signatures Database (MSigDB) (Supplementary Table S6). Sample size: *n* = 4 per group. CPM, counts per million; OD, aging DOCA; OS, aging sham.

Principal component analyses confirmed that aging sham and age-matched DOCA mice formed two distinctive groups (**Figure 4A**). Thirty seven genes were differentially expressed between the two groups, 11 genes were down- and 26 were up-regulated (**Figure 4B** and Supplementary Table S5). This included the up-regulation of the gene for collagen VIII (*Col8a1*), and the down-regulation of the mineralocorticoid receptor *Nr3c2*. Potassium voltage-gate *Kcna1* was highly down-regulated and has been previously associated with atrial fibrillation (Glasscock et al., 2015). Overall in aging DOCA we observed a significant increase in pathways for collagen formation and apoptosis, an enrichment of the innate immune system, inflammasomes, and platelet activation, signaling and aggregation (Supplementary Table S6). We also observed a decrease in voltage-gated potassium channels (**Figures 4C,D**).

### Genes in Common Between Young and Aging DOCA

**FIGURE 5:**
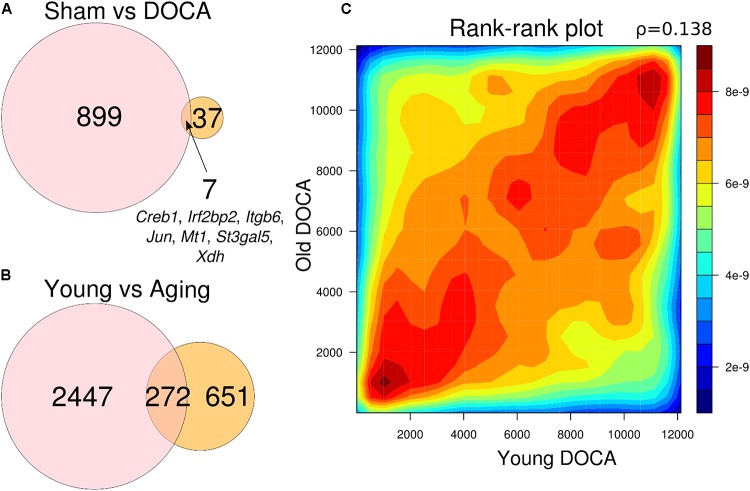
Venn diagrams showing number of genes differentially expressed between **(A)** sham vs. DOCA mice according to age group (young vs. aging) and genes in common between the two groups; and **(B)** young and aging mice according to cardiovascular health (sham vs. DOCA). All genes in **(A)** and **(B)** are FDR < 0.05. **(C)** Rank–rank plot of all detected genes between young and aging DOCA mice showing they have similar patterns of gene expression.

When comparing the two age groups, only seven genes were consistently differentially expressed between sham and DOCA below the significance threshold (FDR < 0.05): *Creb1, Irf2bp2, Itgb6, Jun, Mt1, St3gal5*, and *Xdh* (**Figure 5A**). This suggests that different genes and, potentially, mechanisms are involved in the development of heart disease with aging in the DOCA model. Moreover, we found that only 272 genes were in common between young vs. aging sham (equivalent to only 11% of all differentially expressed genes) or DOCA (42% of all differentially expressed genes) (**Figure 5B**). This also suggests that mechanisms of aging are different between healthy and diseased hearts. However, when we performed a rank–rank plot to visualize how similarly genes behave at the transcriptome-wide level, we found that the two age contrasts have similar patterns of gene expression (**Figure 5C**). This prompted us to explore in detail the pathways involved in the development of heart failure with aging in the DOCA model.

### Pathways in Opposite Direction Between Aging Sham and DOCA

**FIGURE 6:**
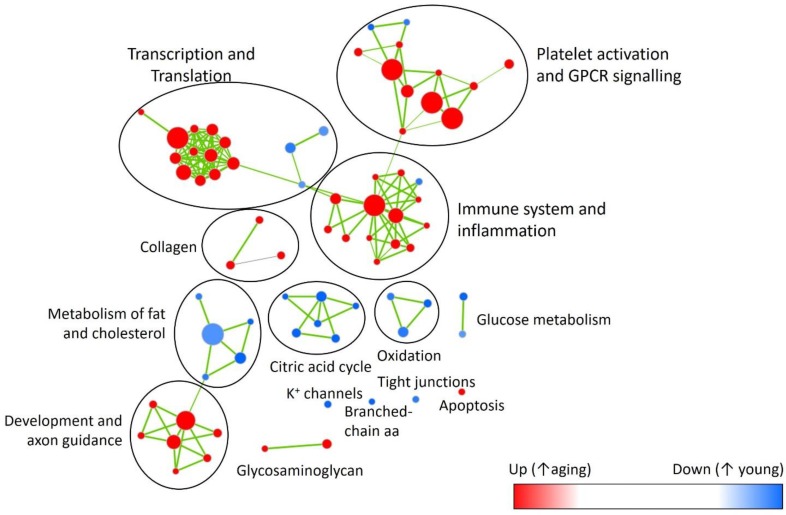
Enrichment map highlighting major pathway up- (*red*) or down-regulated (*blue*) with aging. Showing reactome pathways (Supplementary Table S8) that had false discovery rate <0.05 in the gene-set enrichment analysis.

In factorial analysis of gene expression, 30 genes were up-regulated and 32 were down-regulated between young and aging DOCA (Supplementary Figure S1 and Supplementary Table S7). Using the Reactome database for young DOCA, there was the up-regulation of pathways related to respiratory electron transport chain and oxidation, nitric oxide signaling, potassium channels, and cholesterol biosynthesis (**Figure 6** and Supplementary Table S8). Some of these findings, particularly the increase in oxidative stress and respiratory electron transport chain, were also apparent in the analysis of an independent database (MsigDB) (Supplementary Table S9). In aging DOCA, we observed an increase in cytokine and platelet signaling, (innate) immune system, inflammasomes, angiogenesis, response to wound healing, TNF-alpha, and collagen formation (Supplementary Tables S8, S9). Commonly up-regulated pathways in both young and aging DOCA related to cell cycle (e.g., DNA replication) and down-regulation of fatty acid metabolism and endoplasmic reticulum *N*-glycan/calnexin pathways. These findings are summarized in **Figure 6**, which shows the contrast between ages and disease state according to MsigDB pathways, and Supplementary Figures S2, S3 which show an enrichment map for the pathways dysregulated according to age and support that age is a stronger phenotype than disease.

**FIGURE 7:**
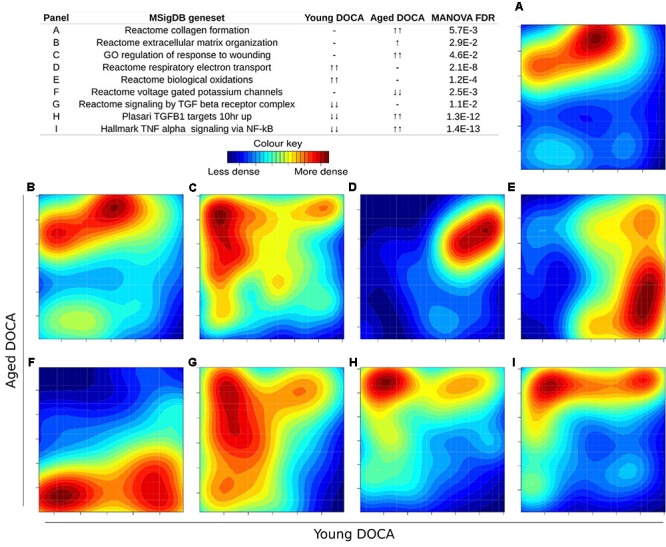
Two-dimensional pathway analysis showing differences between young and aging DOCA mice. Compared to young mice, aging DOCA mice had higher **(A)** collagen formation, **(B)** extracellular matrix organization, and **(C)** wound healing. **(D)** Both young and aging DOCA mice had activation of the respiratory electron transport chain. **(E)** Young DOCA mice had activation of oxidation and **(F)** potassium channels. On the other hand, aging DOCA had higher activation of TGF-beta **(G)** receptor and **(H)** targets, and **(I)** TNF-alpha genes. Sample size: *n* = 6 young DOCA vs. *n* = 4 aging DOCA mice.

**FIGURE 8:**
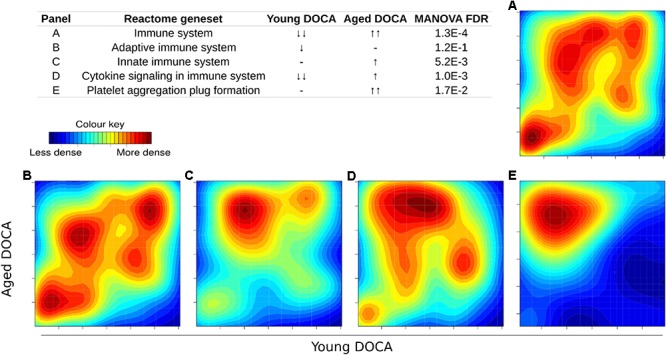
Two-dimensional pathway analysis showing the activation of the immune system and inflammation in DOCA mice. **(A)** The immune system and **(B)** adaptive immune system were up-regulated in both ages. **(C)** The innate immune system, **(D)** cytokine, and **(E)** platelet signaling, among other inflammatory markers, were specifically up-regulated in aging DOCA mice only. Sample size: *n* = 6 young DOCA vs. *n* = 4 aging DOCA mice.

We then performed two-dimensional (2D) pathway analysis to identify patterns of DOCA responsive regulation in young and aging mice. These analyses are unique as they allow us to visualize the regulation of genes that are specific to each age. For example, they show clearly the activation of pathways that are a hallmark of fibrosis including collagen formation, extracellular matrix organization, and wound healing in aging DOCA only (**Figures 7A–C**). These are most likely independent of activation of pathways associated with the respiratory electron chain transport, as this was up-regulated in both ages (**Figure 7D**), while oxidation was only up-regulated in young DOCA (**Figure 7E**) and potassium channels were down-regulated in aged DOCA as compared to young DOCA (**Figure 7F**). On the other hand, aging DOCA had higher activation of TGF-beta receptor and TNF-alpha genes (**Figures 7G–I**). Importantly, while the (adaptive) immune system in general was up-regulated in both ages, the innate immune system, cytokine, and platelet signaling, among other inflammatory markers, were specifically up-regulated in aging DOCA only (**Figure 8**).

**FIGURE 9:**
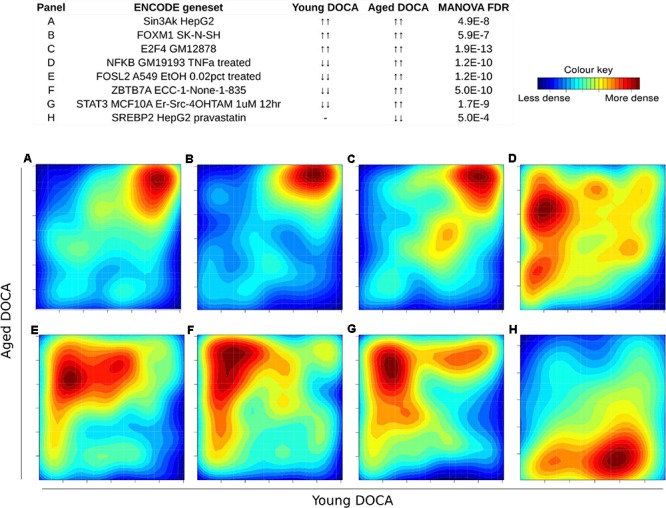
Two-dimensional pathway analysis of transcription factors, showing up-regulation of **(A)** Sin3A, **(B)** FOXM1, and **(C)** E2F4 targets. However, it caught our attention that very few were down-regulated in both ages with the DOCA phenotype. There were several transcription factors with increased expression in older hearts and lower expression in younger hearts, including **(D)** *Nfkb*, **(E)** *Fosl2*, **(F)** *Zbtb7a*, and **(G)** *Stat3*. **(H)** On the other hand there were few with increases in young hearts and decreases in older hearts, the best example being *Srebp2*. Sample size: *n* = 6 young DOCA vs. *n* = 4 aging DOCA mice.

**FIGURE 10:**
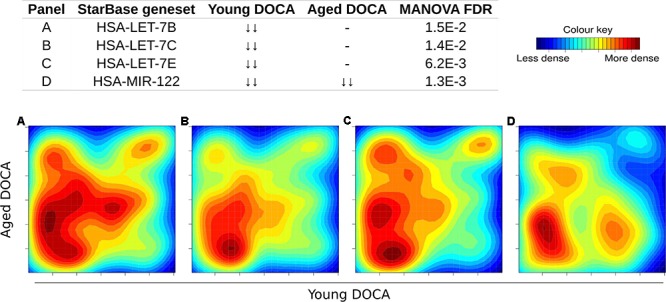
Two-dimensional pathway analysis of microRNAs whose targets were down-regulated in both young and aging DOCA mice (full list at Supplementary Table S10). Showing some examples including **(A)** let-7B, **(B)** let-7c, **(C)** let-7e, and **(D)** miR-122.

We also performed a transcription factor target analysis which showed the common up-regulation of *E2f4, Sin3A*, and *Foxm1* targets (**Figures 9A–C**). However, it was apparent that expression levels of very few target genes were down-regulated in both ages with the DOCA phenotype. There were several transcription factor target sets with increased expression in older hearts and lower expression in younger hearts, including *Nfkb, Fosl2, Zbtb7a*, and *Stat3* (**Figure 9**). On the other hand there were few with increases in young hearts and decreases in older hearts, the best example being *Srebp2* (**Figure 9**). To address the observation that expression of only a few transcription factors targets were elevated in DOCA mice we explored the hypothesis that this may be due to general transcriptional activation due to stress and that the down-regulated genes could be the result of other mechanisms of regulation including small-RNA based degradation. We ran a 2D pathway analysis using gene sets previously mined from starBase which contains curated lists of targets of miRNAs determined empirically. Of interest, the results were dominated by miRNAs whose targets are repressed in both young and older mice, including a list of 267 miRNAs (Supplementary Table S10 and examples in **Figure 10**).

## Discussion

This study reports, for the first time, a detailed integrative study of phenotype and cardiac transcriptomics in regard to the effects of aging in the context of a hypertensive stimulus. Importantly, although the diseased cardiovascular phenotype was mostly consistent between different ages, aging mice had higher markers of cardiac stiffening, such as LVEDP and perivascular fibrosis, than young mice exposed to the same phenotype. Combined with the transcriptomic results, our findings suggest that hypertensive heart failure might develop through different mechanisms according to age, and as such these findings are of direct relevance to human disease. In young mice, hypertension was associated with higher expression of genes involved in oxidative stress in the heart, likely due to activation of the respiratory electron transport chain, while in aging mice collagen formation (measured as mRNA and protein) was associated with high transcriptomic activity of the innate immune system and several markers of inflammation including cytokine and platelet signaling. Only seven genes, including *Creb1, Irf2bp2, Itgb6, Jun, Mt1, St3gal5*, and *Xdh* were concomitantly dysregulated in both ages in DOCA mice, but targets for 267 miRNAs were down-regulated.

Aging models of cardiac dysfunction have been previously proposed. This includes the spontaneous senescence-prone (SAMP8) mice (Reed et al., 2011), male FVB/N mice (Koch et al., 2013), Fischer 344 rats (Forman et al., 1997), and crossed between Fischer 344 and Brown Norway rats (Hacker et al., 2006). These models, however, are not specific of HFPEF and present several other aging phenotypes. SAMP8 mice, for example, did not develop heart failure despite evidence of diastolic dysfunction (Reed et al., 2011). In others, the development of heart failure was sex-specific (Forman et al., 1997; Koch et al., 2013). A common factor in aging models, however, was increased cardiac fibrosis with higher inflammatory markers (Hacker et al., 2006; Reed et al., 2011), which is consistent with the present study. While we observed a clear cluster in the PCA of aged mice (**Figure 3A**), this was not detected in young mice (**Figure 2A**), but the heat map (**Figure 3D**) supports different pathways in young sham and DOCA mice (**Figure 2**). However, when we compared young vs. aged DOCA mice (Supplementary Table S9), it is clear that young DOCA mice are a distinctive group.

Significant age-dependency of cardiovascular responses following disease insult have previously been reported. We (X-JD) previously reported that the incidence of cardiac rupture and remodeling following myocardial infarction was higher in 12-month old compared to 3-month old mice (Yang et al., 2008). This included higher BP and left ventricle chamber dilation, but also inflammatory cytokines and collagen levels (Yang et al., 2008). Along this line, our present study reveals significant differences in cardiac phenotypes between 42 weeks and younger (16 weeks) mice. Such age-gap might not appear very large, but it is sufficient to affect cardiovascular responses to pathological stress.

Our findings of increased transcription of oxidative stress genes in young DOCA mice are consistent with previous studies that used similarly younger mice (Mohammed et al., 2010; Lovelock et al., 2012). In aging DOCA, we observed higher levels of collagen protein, collagen formation pathways and expression of the *Col8a1*. *Col8a1* is a short-chain collagen which protein is present in the adult and developing heart, smooth, and skeletal muscle among other tissues (Sage and Iruela-Arispe, 1990; Uhlen et al., 2015). Lack of *Col8a1* reduced cardiac fibrosis in a transverse aortic constriction (TAC) model, and decreased myofibroblast differentiation *in vitro* (Skrbic et al., 2015). Human macrophages were shown to secrete *Col8a1 in vitro* (Weitkamp et al., 1999). While there appears to be a link between macrophages, *Col8a1* and vascular remodeling in response to injury (Sibinga et al., 1997; Weitkamp et al., 1999), a role for macrophages secreting *Col8a1* in the hypertensive heart is still to be determined. Compared to young DOCA mice, aging DOCA mice also had activation of axon guidance (Supplementary Table S8), which might be related to the activation of cardiac sympathetic innervation, known to have a role in heart failure and the aging human heart (Kaye and Esler, 2005).

The DOCA model was also associated with down-regulation of targets for 267 miRNAs (Supplementary Table S10). Among these were targets of miRNAs we previously described to be released from the failing heart, including let-7b-5p, let-7c-5p, let-7e-5p, and miR-122-5p (**Figure 8**; Marques et al., 2016). Levels of miR-30c, miR-146a, and miR-221 were lower in both types of heart failure (Watson et al., 2015), while miR-190 and miR-545-5p were specific to HFPEF patients relative to control subjects (Wong et al., 2015). The miR-29 family was recently shown to regulate the Wtn signaling, resulting in cardiac hypertrophy and fibrosis (Sassi et al., 2017). This is consistent with our findings that support that hundreds of targets of miR-29a, miR-29b, and miR-29c were down-regulated in young and aging DOCA (Supplementary Table S10).

To further understand the molecular mechanisms behind heart failure in the aging hypertensive heart, future studies should take advantage of new single-cell RNA-sequencing techniques, or isolation of specific cell lines as recently done by us (MZ, AK, and AE-O) (Quaife-Ryan et al. (2017). Specifically, in the current study we demonstrated evidence of accentuated perivascular fibrosis in aging mice. The origin of the perivascular fibrosis was not determined in our study. Previously, we showed that only a modest proportion of the cardiac fibroblasts in the fibrotic myocardium are derived from bone marrow (Chu et al., 2010) suggesting that the majority of fibrosis arises from resident cardiac fibroblasts. As such, in the context of perivascular fibrosis, it is possible that proliferation of pericytes play an important role (Travers et al., 2016). Future studies should also explore aging in other models of cardiovascular disease, such as the angiotensin II and TAC models. Our findings also need to be replicated in female mice, as half of heart failure patients are women (Dunlay et al., 2017). Finally, we did not validate the pathways identified due to lack of tissue. Although our data was supported by findings in previous studies, including in human heart failure, further studies will have to establish the reasons behind the preferred transcriptome regulation by miRNAs instead of transcription factors.

Rodent models are particularly useful to study human disease due to their small size, lower cost, and shorter lifespan compared to large animal models. Although animal models are invaluable to advance medical research, few pre-clinical models indeed fulfill the clinical features of HFPEF (Conceicao et al., 2016). The current crisis in lack of reproducibility of findings from pre-clinical to clinical studies and, thus, low translation of findings to the clinic, has unleashed a call for improved methodological rigor (Ramirez et al., 2017). Rodent models of HFPEF have been reviewed somewhere else (Conceicao et al., 2016). Particularly interesting was the combination of DOCA with TAC models as a model of HFPEF (Mohammed et al., 2010). We propose, however, that using models that resemble more closely heart failure such as combining hypertension and aging might improve translation, as the molecular mechanisms behind the phenotype would be more relevant.

## Conclusion

Our cardiac transcriptomic findings support that the molecular mechanisms behind the development of hypertension change in the context of aging. With aging, there was the up-regulation the (innate) immune system, together with activation of platelet and cytokine signaling, supporting higher levels of inflammation that may lead to the higher collagen formation observed (both in phenotype and molecular level). These data provide new insights into potential therapeutic targets for hypertensive heart failure and also highlight the importance of using aging mice in experimental studies.

## Author Contributions

FM and DK: conception and design. FM, P-YC, MZ, and HK: collection of data. FM, P-YC, MZ, AK, HK, and DK: analysis and interpretation of data. FM, MZ, and DK: drafting of the manuscript. FM, P-YC, MZ, AK, HK, X-JD, AE-O, and DK: manuscript revision for important intellectual content. FM, P-YC, MZ, AK, HK, X-JD, AE-O, and DK: final approval of the manuscript submitted.

## Conflict of Interest Statement

The authors declare that the research was conducted in the absence of any commercial or financial relationships that could be construed as a potential conflict of interest.
