# Supplementary material for: Age-Related Differential Structural and Transcriptomic Responses in the Hypertensive Heart
Source: Front Physiol. 2018 Jul 9;9:817. doi: 10.3389/fphys.2018.00817 (PMC6046461; doi:10.3389/fphys.2018.00817)
Supplement: Supplementary file 1 [file Data_Sheet_1.DOCX]

**Supplementary Figures S1-S4 and Tables S1-S8 for**

**Age-Related Differential Structural and Transcriptomic Responses in the Hypertensive Heart**

Francine Z. Marques, PhD^1,2^*; Po-Yin Chu, PhD^1^*; Mark Ziemann, PhD^3^; Antony Kaspi, MEng;^3^ Helen Kiriazis, PhD;^4^ Xiao-Jun Du, PhD;^4,7^ Assam El-Osta, PhD^3^; [David M. Kaye](http://circheartfailure.ahajournals.org/search?author1=David+M.+Kaye&sortspec=date&submit=Submit), MD, PhD^1,5,6^

^1^Heart Failure Research Group, Baker Heart and Diabetes Institute, Melbourne, Australia;

^2^Department of Pharmacology, Faculty of Medicine Nursing and Health Sciences, Monash University, Melbourne, Australia; ^3^Epigenetics in Human Health and Disease, Department of Diabetes, Monash University, Melbourne, Australia; ^4^Experimental Cardiology Laboratory, Baker Heart and Diabetes Institute, Melbourne, Australia; ^5^Department of Pathology, The University of Melbourne, Melbourne, Australia; ^5^Central Clinical School, Faculty of Medicine Nursing and Health Sciences, Monash University, Melbourne, Australia; ^6^Heart Centre, Alfred Hospital, Melbourne, Australia; ^7^Hong Kong Institute of Diabetes and Obesity, Prince of Wales Hospital, The Chinese University of Hong Kong, Hong Kong SAR

*contributed equally for this study

**
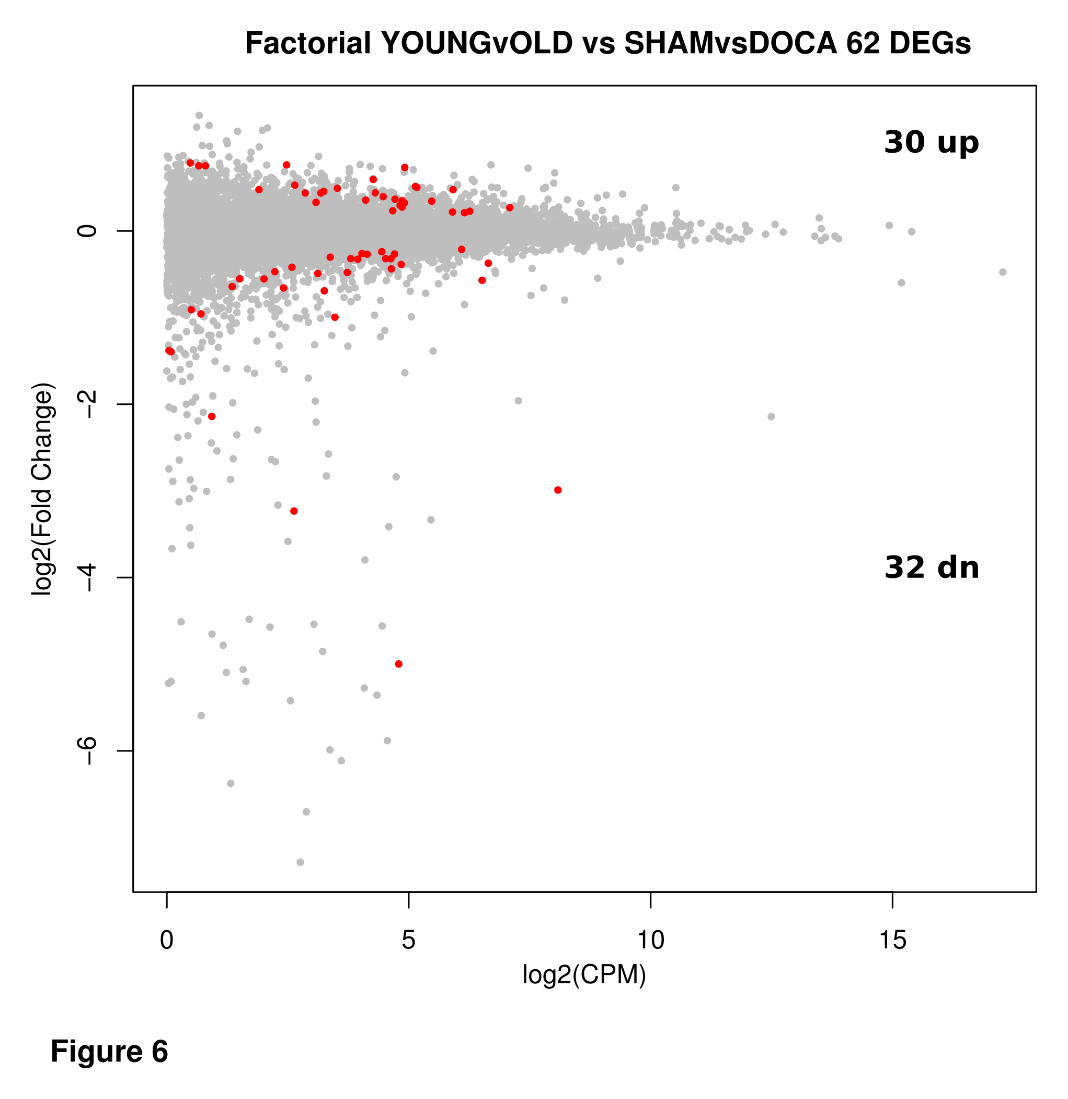
**

**Figure S1**. **Factorial analysis comparing young versus ageing DOCA after adjustment for ageing.** Legend: CPM, counts per million.

**
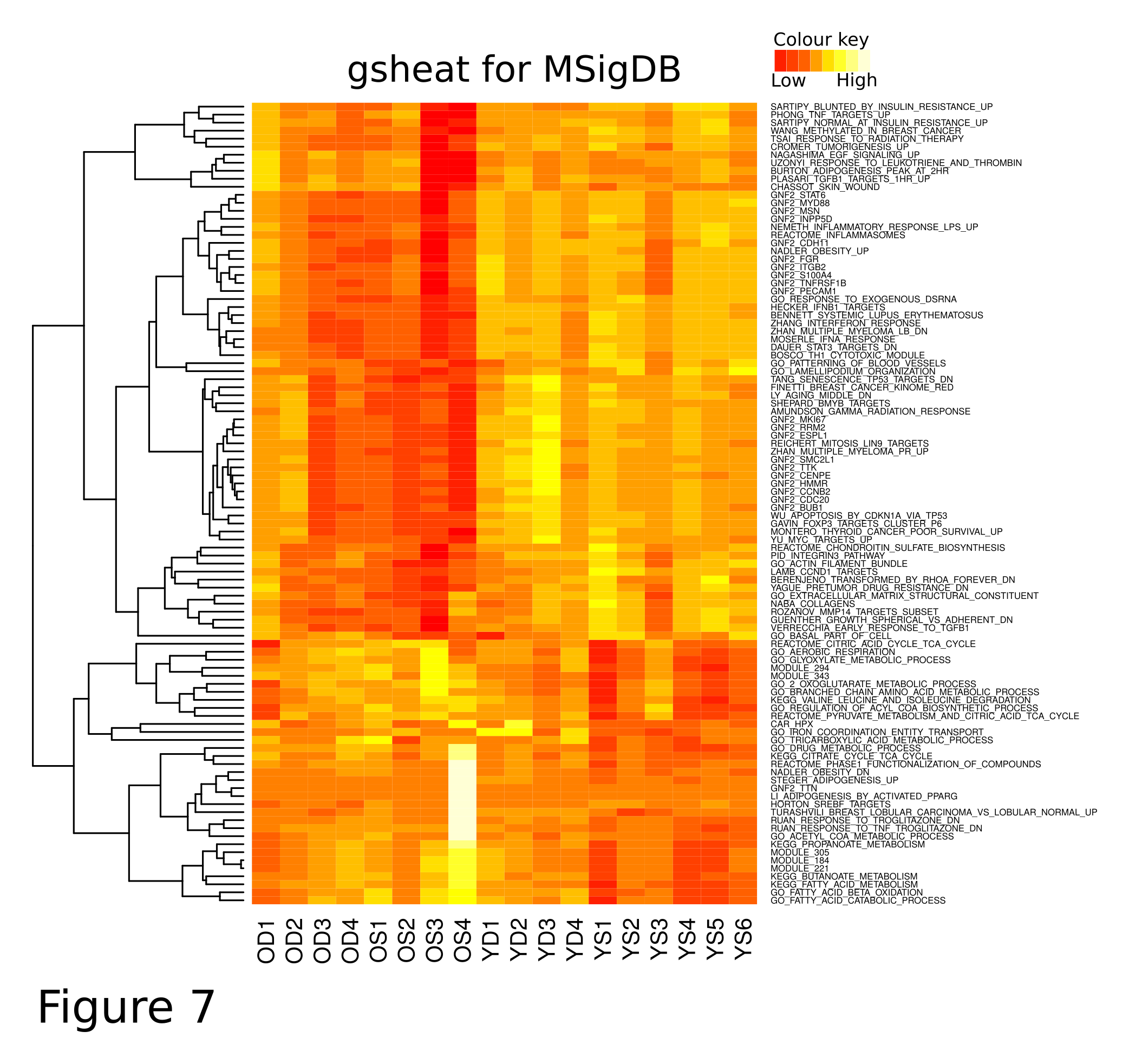
 Figure S2**. **Unsupervised hierarchical clustering analysis showing contrast of differentially regulated pathways of the Molecular Signatures Database (MSigDB) group together based on age and disease state (Table S9).** Legend: OD, ageing DOCA; OS, ageing sham; YD, young DOCA; YS, young sham.

**Figure S3.** **Principal component analyses shows young and aged DOCA mice clusters are distinct.** Legend: OD, ageing DOCA; YD, young DOCA.

**Table S1.** Quality metrics supplied by STAR.

| **Metric** | **1AH1** | **2AH1** | **3AH1** | **4AH1** | **5AH1** | **6AH1** | **10AH1** | **12AH1** | **13AH1** | **14AH2** |
| --- | --- | --- | --- | --- | --- | --- | --- | --- | --- | --- |
| Number_of_input_reads_ | 10179493 | 25401455 | 15449120 | 30552654 | 28263956 | 26639334 | 30409815 | 23945391 | 11860958 | 14408142 |
| Uniquely mapped reads % | 86.05% | 84.48% | 80.17% | 79.10% | 81.42% | 81.37% | 80.90% | 82.58% | 81.05% | 75.02% |
| Mismatch rate per base, % | 0.16% | 0.16% | 0.17% | 0.20% | 0.17% | 0.17% | 0.17% | 0.16% | 0.16% | 0.22% |
| % of reads mapped to multiple loci | 10.92% | 12.25% | 12.85% | 12.12% | 12.60% | 12.64% | 13.43% | 13.61% | 12.92% | 12.46% |
| % of reads mapped to too many loci | 0.31% | 0.31% | 0.25% | 0.26% | 0.27% | 0.27% | 0.28% | 0.28% | 0.25% | 0.23% |
| % of reads unmapped: too short | 2.40% | 2.71% | 6.53% | 8.30% | 5.49% | 5.50% | 5.18% | 3.28% | 5.54% | 12.11% |
| rRNA proportion* | 1.10% | 1.81% | 6.46% | 8.02% | 5.07% | 4.66% | 4.34% | 2.39% | 4.09% | 13.02% |

| **Metric** | **14AH2** | **31AH2** | **32AH2** | **36AH2** | **37AH2** | **54AH2** | **55AH2** | **56AH2** | **57AH2** |
| --- | --- | --- | --- | --- | --- | --- | --- | --- | --- |
| Number_of_input_reads_ | 14408142 | 10243750 | 31873743 | 21599097 | 23687175 | 25403930 | 17849337 | 9710957 | 8163796 |
| Uniquely mapped reads % | 75.02% | 79.62% | 80.74% | 80.10% | 82.04% | 81.51% | 81.65% | 81.40% | 81.59% |
| Mismatch rate per base, % | 0.22% | 0.17% | 0.15% | 0.16% | 0.14% | 0.14% | 0.13% | 0.12% | 0.13% |
| % of reads mapped to multiple loci | 12.46% | 12.45% | 13.02% | 12.72% | 12.29% | 14.80% | 15.62% | 15.98% | 15.29% |
| % of reads mapped to too many loci | 0.23% | 0.24% | 0.26% | 0.23% | 0.27% | 0.28% | 0.28% | 0.26% | 0.28% |
| % of reads unmapped: too short | 12.11% | 7.43% | 5.70% | 6.71% | 5.16% | 3.14% | 2.19% | 2.10% | 2.58% |
| rRNA proportion* | 13.02% | 7.25% | 5.06% | 6.51% | 5.01% | 2.34% | 1.11% | 1.20% | 1.23% |

Legend: *determined by mapping of 1 million reads to rRNA fragments 5S, 18S, 28S and Rs5-8s1.

**Table S2.** Assignment of reads to genes.

| **Status** | **10AH1** | **12AH1** | **13AH1** | **14AH2** | **1AH1** | **2AH1** | **31AH2** | **32AH2** | **36AH2** |
| --- | --- | --- | --- | --- | --- | --- | --- | --- | --- |
| Assigned | 18255059 | 14149401 | 6845302 | 8287051 | 5901788 | 15534127 | 5665708 | 18023373 | 12770552 |
|  |  |  |  |  |  |  |  |  |  |
| Unassigned: Ambiguity | 171232 | 138574 | 64003 | 73984 | 63476 | 164686 | 47196 | 158220 | 116348 |
|  |  |  |  |  |  |  |  |  |  |
| Unassigned: | 6173996 | 5487142 | 2704225 | 2448381 | 2793726 | 5759980 | 2443232 | 7552473 | 4414454 |
| No Features |  |  |  |  |  |  |  |  |  |
|  |  |  |  |  |  |  |  |  |  |
| Unassigned: | 9673247 | 7760603 | 3614084 | 4317313 | 2719930 | 7602678 | 3020342 | 9852011 | 6471770 |
| Mapping Quality |  |  |  |  |  |  |  |  |  |
|  |  |  |  |  |  |  |  |  |  |
| **Status** | **37AH2** | **3AH1** | **4AH1** | **54AH2** | **55AH2** | **56AH2** | **57AH2** | **5AH1** | **6AH1** |
| Assigned | 14109895 | 9245629 | 17430241 | 14460211 | 10073125 | 5476860 | 4577589 | 16889003 | 15803770 |
|  |  |  |  |  |  |  |  |  |  |
| Unassigned: Ambiguity | 132042 | 76583 | 167057 | 147033 | 101139 | 53552 | 44444 | 162489 | 157321 |
|  |  |  |  |  |  |  |  |  |  |
| Unassigned: | 5190117 | 3063180 | 6569927 | 6098918 | 4399176 | 2374181 | 2038513 | 5961041 | 5715615 |
| No Features |  |  |  |  |  |  |  |  |  |
|  |  |  |  |  |  |  |  |  |  |
| Unassigned: | 6886203 | 4658440 | 8966453 | 8926235 | 6520301 | 3607571 | 2860887 | 8446266 | 8079183 |
| Mapping Quality |  |  |  |  |  |  |  |  |  |

| **Table S3.** Cardiac genes differentially expressed between young sham vs DOCA mice (FDR<0.05). | | | | | | |
| --- | --- | --- | --- | --- | --- | --- |
| **Transcript access** | **Gene symbol** | **logFC** | **Fold change** | **logCPM** | ***P*-Value** | **FDR** |
| ENSMUSG00000024673 | *Ms4a1* | -1.39 | -4.03 | -0.02 | 9.73E-05 | 0.004622 |
| ENSMUSG00000075224 | *Lrrc55* | -1.36 | -3.89 | 1.27 | 0.001314 | 0.028646 |
| ENSMUSG00000030724 | *Cd19* | -1.28 | -3.61 | 0.08 | 4.10E-08 | 0.000010 |
| ENSMUSG00000032053 | *Pou2af1* | -1.27 | -3.55 | -0.41 | 0.000285 | 0.009624 |
| ENSMUSG00000039264 | *Gimap3* | -1.25 | -3.51 | 1.53 | 8.90E-17 | 0.000000 |
| ENSMUSG00000014030 | *Pax5* | -1.24 | -3.46 | 0.26 | 4.50E-08 | 0.000010 |
| ENSMUSG00000050600 | *Zfp831* | -1.14 | -3.14 | -0.07 | 4.39E-05 | 0.002443 |
| ENSMUSG00000074497 | *A430078G23Rik* | -1.14 | -3.13 | 0.28 | 3.12E-07 | 0.000053 |
| ENSMUSG00000034796 | *Cpne7* | -1.05 | -2.85 | 1.03 | 1.89E-11 | 0.000000 |
| ENSMUSG00000003882 | *Il7r* | -1.04 | -2.82 | 0.63 | 0.000267 | 0.009252 |
| ENSMUSG00000060716 | *Plekhh1* | -1.01 | -2.74 | 0.95 | 0.000431 | 0.012940 |
| ENSMUSG00000109245 | *Gm44860* | -1.00 | -2.72 | 1.52 | 0.00017 | 0.006905 |
| ENSMUSG00000052013 | *Btla* | -1.00 | -2.72 | 0.78 | 4.65E-09 | 0.000002 |
| ENSMUSG00000066170 | *E230001N04Rik* | -0.99 | -2.69 | 0.82 | 4.61E-06 | 0.000443 |
| ENSMUSG00000050921 | *P2ry10* | -0.98 | -2.67 | 0.54 | 0.001732 | 0.034178 |
| ENSMUSG00000041538 | *H2-Ob* | -0.97 | -2.64 | 1.21 | 6.02E-11 | 0.000000 |
| ENSMUSG00000104213 | *Ighd* | -0.96 | -2.62 | 0.88 | 1.14E-06 | 0.000147 |
| ENSMUSG00000041653 | *Pnpla3* | -0.95 | -2.59 | 1.55 | 0.001155 | 0.026200 |
| ENSMUSG00000107352 | *Gm43660* | -0.90 | -2.46 | 2.11 | 0.00017 | 0.006905 |
| ENSMUSG00000108897 | *Gm44861* | -0.89 | -2.43 | 0.40 | 0.000116 | 0.005195 |
| ENSMUSG00000026358 | *Rgs1* | -0.87 | -2.39 | 0.97 | 0.000664 | 0.017472 |
| ENSMUSG00000068227 | *Il2rb* | -0.87 | -2.38 | 1.41 | 0.000312 | 0.010147 |
| ENSMUSG00000098900 | *Gm18190* | -0.84 | -2.33 | -0.14 | 0.001423 | 0.030097 |
| ENSMUSG00000034028 | *Cd226* | -0.84 | -2.32 | 0.51 | 0.001914 | 0.035675 |
| ENSMUSG00000001588 | *Acap1* | -0.84 | -2.31 | 0.75 | 0.000137 | 0.005855 |
| ENSMUSG00000073421 | *H2-Ab1* | -0.83 | -2.30 | 6.31 | 0.000195 | 0.007566 |
| ENSMUSG00000030000 | *Add2* | -0.83 | -2.28 | 1.37 | 0.001744 | 0.034179 |
| ENSMUSG00000022951 | *Rcan1* | -0.82 | -2.27 | 7.02 | 0.00056 | 0.015285 |
| ENSMUSG00000059659 | *Gm10069* | -0.82 | -2.26 | -0.06 | 0.002614 | 0.044153 |
| ENSMUSG00000099757 | *BE692007* | -0.81 | -2.25 | 0.39 | 2.95E-05 | 0.001839 |
| ENSMUSG00000011256 | *Adam19* | -0.80 | -2.23 | 6.36 | 5.33E-16 | 0.000000 |
| ENSMUSG00000055116 | *Arntl* | -0.80 | -2.23 | 4.01 | 0.000229 | 0.008426 |
| ENSMUSG00000043243 | *Fam129c* | -0.80 | -2.23 | 0.24 | 7.83E-05 | 0.003903 |
| ENSMUSG00000037548 | *H2-DMb2* | -0.80 | -2.23 | 0.04 | 0.000443 | 0.013196 |
| ENSMUSG00000041552 | *Ptchd1* | -0.78 | -2.18 | 1.81 | 0.000675 | 0.017683 |
| ENSMUSG00000035407 | *Kank4* | -0.78 | -2.18 | 2.63 | 0.001889 | 0.035613 |
| ENSMUSG00000020427 | *Igfbp3* | -0.75 | -2.13 | 4.66 | 9.26E-09 | 0.000003 |
| ENSMUSG00000058145 | *Adamts17* | -0.74 | -2.10 | 0.72 | 0.002501 | 0.042791 |
| ENSMUSG00000041633 | *Kctd12b* | -0.74 | -2.10 | 5.34 | 1.57E-17 | 0.000000 |
| ENSMUSG00000060586 | *H2-Eb1* | -0.73 | -2.08 | 6.56 | 0.000252 | 0.009062 |
| ENSMUSG00000052485 | *Tmem171* | -0.73 | -2.08 | 1.29 | 0.001703 | 0.033931 |
| ENSMUSG00000030365 | *Clec2i* | -0.72 | -2.06 | 0.17 | 0.000474 | 0.013789 |
| ENSMUSG00000020431 | *Adcy1* | -0.72 | -2.05 | 2.31 | 3.68E-06 | 0.000379 |
| ENSMUSG00000033450 | *Tagap* | -0.72 | -2.05 | 1.94 | 5.32E-08 | 0.000011 |
| ENSMUSG00000030577 | *Cd22* | -0.71 | -2.04 | 0.18 | 0.002622 | 0.044234 |
| ENSMUSG00000021403 | *Serpinb9b* | -0.70 | -2.02 | 0.32 | 0.000622 | 0.016495 |
| ENSMUSG00000027843 | *Ptpn22* | -0.70 | -2.01 | 2.10 | 0.000499 | 0.014121 |
| ENSMUSG00000049281 | *Scn3b* | -0.70 | -2.01 | 2.75 | 0.003075 | 0.049342 |
| ENSMUSG00000036594 | *H2-Aa* | -0.70 | -2.01 | 6.72 | 0.000379 | 0.011689 |
| ENSMUSG00000018168 | *Ikzf3* | -0.69 | -2.00 | 1.02 | 2.60E-05 | 0.001668 |
| ENSMUSG00000026971 | *Itgb6* | -0.68 | -1.98 | 5.05 | 8.36E-22 | 0.000000 |
| ENSMUSG00000048489 | *8430408G22Rik* | -0.68 | -1.98 | 4.87 | 1.48E-05 | 0.001057 |
| ENSMUSG00000022504 | *Ciita* | -0.68 | -1.97 | 3.31 | 0.000165 | 0.006829 |
| ENSMUSG00000047085 | *Lrrc4b* | -0.67 | -1.96 | 3.62 | 0.0024 | 0.041773 |
| ENSMUSG00000075270 | *Pde11a* | -0.67 | -1.95 | 1.68 | 1.75E-05 | 0.001215 |
| ENSMUSG00000108460 | *Gm44557* | -0.67 | -1.95 | 0.76 | 6.22E-05 | 0.003256 |
| ENSMUSG00000026077 | *Npas2* | -0.66 | -1.94 | 3.19 | 0.000716 | 0.018458 |
| ENSMUSG00000005947 | *Itgae* | -0.65 | -1.92 | 0.64 | 0.00051 | 0.014353 |
| ENSMUSG00000024610 | *Cd74* | -0.65 | -1.91 | 7.70 | 0.002764 | 0.045675 |
| ENSMUSG00000102533 | *Gm37226* | -0.65 | -1.91 | 0.10 | 0.00207 | 0.037608 |
| ENSMUSG00000053541 | *Gm4759* | -0.63 | -1.88 | 0.91 | 0.002342 | 0.041049 |
| ENSMUSG00000097585 | *E230029C05Rik* | -0.63 | -1.88 | 1.80 | 0.002046 | 0.037317 |
| ENSMUSG00000048521 | *Cxcr6* | -0.63 | -1.87 | 0.20 | 0.001782 | 0.034509 |
| ENSMUSG00000048251 | *Bcl11b* | -0.62 | -1.86 | 0.95 | 0.000176 | 0.007049 |
| ENSMUSG00000037169 | *Mycn* | -0.62 | -1.86 | 3.59 | 0.000421 | 0.012662 |
| ENSMUSG00000005672 | *Kit* | -0.62 | -1.86 | 3.49 | 0.001629 | 0.032781 |
| ENSMUSG00000010751 | *Tnfrsf22* | -0.61 | -1.84 | 2.04 | 1.94E-08 | 0.000006 |
| ENSMUSG00000054978 | *Kbtbd13* | -0.61 | -1.84 | 1.50 | 0.00188 | 0.035613 |
| ENSMUSG00000026832 | *Cytip* | -0.60 | -1.83 | 2.45 | 0.00177 | 0.034377 |
| ENSMUSG00000040350 | *Trim7* | -0.59 | -1.81 | 4.53 | 7.34E-09 | 0.000003 |
| ENSMUSG00000038390 | *Gpr162* | -0.59 | -1.81 | 0.63 | 0.000467 | 0.013683 |
| ENSMUSG00000105822 | *Gm42969* | -0.59 | -1.80 | 1.31 | 0.000774 | 0.019575 |
| ENSMUSG00000057948 | *Unc13d* | -0.58 | -1.79 | 1.40 | 6.51E-05 | 0.003385 |
| ENSMUSG00000070803 | *Cited4* | -0.58 | -1.79 | 3.56 | 2.00E-07 | 0.000036 |
| ENSMUSG00000015533 | *Itga2* | -0.58 | -1.78 | 1.39 | 0.001469 | 0.030406 |
| ENSMUSG00000085936 | *2610307P16Rik* | -0.58 | -1.78 | 1.44 | 4.60E-05 | 0.002530 |
| ENSMUSG00000020335 | *Zfp354b* | -0.57 | -1.77 | 0.80 | 0.00175 | 0.034179 |
| ENSMUSG00000100658 | *F730311O21Rik* | -0.56 | -1.75 | 2.74 | 2.11E-05 | 0.001412 |
| ENSMUSG00000037649 | *H2-DMa* | -0.55 | -1.74 | 2.82 | 0.000327 | 0.010543 |
| ENSMUSG00000055489 | *Ano5* | -0.55 | -1.73 | 2.60 | 0.002466 | 0.042641 |
| ENSMUSG00000052373 | *Mpp3* | -0.55 | -1.73 | 2.91 | 0.001713 | 0.033955 |
| ENSMUSG00000030149 | *Klrk1* | -0.55 | -1.72 | 0.94 | 0.001903 | 0.035675 |
| ENSMUSG00000042351 | *Grap2* | -0.53 | -1.71 | 0.83 | 0.0029 | 0.047324 |
| ENSMUSG00000043085 | *Tmem82* | -0.53 | -1.71 | 4.16 | 6.09E-06 | 0.000531 |
| ENSMUSG00000027217 | *Tspan18* | -0.53 | -1.70 | 3.73 | 4.29E-05 | 0.002403 |
| ENSMUSG00000037922 | *Bank1* | -0.53 | -1.69 | 2.00 | 4.82E-05 | 0.002611 |
| ENSMUSG00000103313 | *Gm38357* | -0.52 | -1.69 | 2.71 | 8.14E-05 | 0.004015 |
| ENSMUSG00000050147 | *F2rl3* | -0.52 | -1.68 | 0.90 | 0.001541 | 0.031273 |
| ENSMUSG00000028654 | *Mycl* | -0.52 | -1.68 | 0.65 | 0.001919 | 0.035682 |
| ENSMUSG00000001493 | *Meox1* | -0.51 | -1.67 | 5.29 | 0.000955 | 0.022677 |
| ENSMUSG00000109243 | *RP23-423B21.6* | -0.50 | -1.65 | 1.39 | 0.002784 | 0.045893 |
| ENSMUSG00000071347 | *C1qtnf9* | -0.50 | -1.65 | 5.46 | 2.37E-07 | 0.000042 |
| ENSMUSG00000097357 | *Gm16793* | -0.50 | -1.65 | 2.92 | 3.28E-05 | 0.002008 |
| ENSMUSG00000006731 | *B4galnt1* | -0.49 | -1.63 | 2.11 | 0.000432 | 0.012940 |
| ENSMUSG00000053604 | *Rpia* | -0.49 | -1.63 | 3.25 | 2.05E-07 | 0.000037 |
| ENSMUSG00000004891 | *Nes* | -0.49 | -1.63 | 7.18 | 1.05E-05 | 0.000830 |
| ENSMUSG00000027221 | *Chst1* | -0.48 | -1.62 | 2.05 | 0.000257 | 0.009140 |
| ENSMUSG00000044813 | *Shb* | -0.48 | -1.61 | 4.41 | 1.19E-05 | 0.000897 |
| ENSMUSG00000024427 | *Spry4* | -0.47 | -1.60 | 5.35 | 2.91E-08 | 0.000008 |
| ENSMUSG00000024440 | *Pcdh12* | -0.47 | -1.60 | 4.76 | 0.000354 | 0.011133 |
| ENSMUSG00000019850 | *Tnfaip3* | -0.47 | -1.60 | 3.45 | 0.0005 | 0.014125 |
| ENSMUSG00000023439 | *Gnb3* | -0.46 | -1.59 | 2.22 | 0.002171 | 0.038777 |
| ENSMUSG00000036036 | *Zfp57* | -0.46 | -1.59 | 1.63 | 0.001146 | 0.026096 |
| ENSMUSG00000024053 | *Emilin2* | -0.46 | -1.59 | 5.49 | 7.20E-07 | 0.000107 |
| ENSMUSG00000039377 | *Hlx* | -0.46 | -1.58 | 3.54 | 0.000484 | 0.013944 |
| ENSMUSG00000021203 | *Otub2* | -0.45 | -1.57 | 2.13 | 3.10E-05 | 0.001910 |
| ENSMUSG00000022508 | *Bcl6* | -0.45 | -1.57 | 4.77 | 0.00027 | 0.009292 |
| ENSMUSG00000036718 | *Micall2* | -0.45 | -1.57 | 3.67 | 0.00143 | 0.030097 |
| ENSMUSG00000073491 | *Pydc4* | -0.45 | -1.56 | 4.23 | 7.40E-05 | 0.003740 |
| ENSMUSG00000068854 | *Hist2h2be* | -0.45 | -1.56 | 2.73 | 1.69E-06 | 0.000207 |
| ENSMUSG00000035105 | *Egln3* | -0.45 | -1.56 | 6.80 | 0.000137 | 0.005855 |
| ENSMUSG00000030323 | *Ift122* | -0.44 | -1.56 | 5.76 | 0.000227 | 0.008402 |
| ENSMUSG00000021806 | *Nid2* | -0.44 | -1.56 | 5.94 | 0.002706 | 0.045258 |
| ENSMUSG00000038587 | *Akap12* | -0.44 | -1.56 | 6.71 | 0.000632 | 0.016728 |
| ENSMUSG00000070436 | *Serpinh1* | -0.44 | -1.55 | 7.73 | 0.000372 | 0.011553 |
| ENSMUSG00000030123 | *Plxnd1* | -0.44 | -1.55 | 7.64 | 1.18E-05 | 0.000892 |
| ENSMUSG00000027435 | *Cd93* | -0.44 | -1.55 | 8.15 | 2.35E-05 | 0.001552 |
| ENSMUSG00000034714 | *Ttyh2* | -0.44 | -1.55 | 3.60 | 0.00049 | 0.014020 |
| ENSMUSG00000021256 | *Vash1* | -0.43 | -1.54 | 5.07 | 0.000186 | 0.007312 |
| ENSMUSG00000050592 | *Fam78a* | -0.43 | -1.53 | 4.78 | 4.33E-05 | 0.002416 |
| ENSMUSG00000056313 | *1810011O10Rik* | -0.43 | -1.53 | 5.25 | 0.00051 | 0.014353 |
| ENSMUSG00000030036 | *Mogs* | -0.43 | -1.53 | 3.37 | 0.002097 | 0.037873 |
| ENSMUSG00000025969 | *Nrp2* | -0.42 | -1.53 | 6.79 | 0.000103 | 0.004772 |
| ENSMUSG00000030616 | *Sytl2* | -0.42 | -1.53 | 1.68 | 0.00135 | 0.028915 |
| ENSMUSG00000020926 | *Adam11* | -0.42 | -1.52 | 3.98 | 0.001739 | 0.034179 |
| ENSMUSG00000020099 | *Unc5b* | -0.42 | -1.52 | 5.41 | 0.001913 | 0.035675 |
| ENSMUSG00000043505 | *Gimap5* | -0.42 | -1.52 | 2.96 | 0.000489 | 0.014020 |
| ENSMUSG00000054435 | *Gimap4* | -0.41 | -1.51 | 5.11 | 3.11E-15 | 0.000000 |
| ENSMUSG00000055612 | *Cdca7* | -0.41 | -1.51 | 1.33 | 0.001264 | 0.027921 |
| ENSMUSG00000071042 | *Rasgrp3* | -0.41 | -1.51 | 5.51 | 9.27E-09 | 0.000003 |
| ENSMUSG00000036862 | *Dchs1* | -0.41 | -1.50 | 5.87 | 0.000294 | 0.009837 |
| ENSMUSG00000020658 | *Efr3b* | -0.41 | -1.50 | 4.69 | 0.000458 | 0.013552 |
| ENSMUSG00000054720 | *Lrrc8c* | -0.40 | -1.50 | 6.30 | 0.000197 | 0.007600 |
| ENSMUSG00000062960 | *Kdr* | -0.40 | -1.50 | 7.75 | 0.001778 | 0.034474 |
| ENSMUSG00000016356 | *Col20a1* | -0.40 | -1.49 | 1.50 | 0.000937 | 0.022347 |
| ENSMUSG00000039405 | *Prss23* | -0.39 | -1.48 | 4.46 | 0.001827 | 0.035063 |
| ENSMUSG00000027329 | *Spef1* | -0.39 | -1.48 | 2.64 | 8.99E-07 | 0.000125 |
| ENSMUSG00000048450 | *Msx1* | -0.39 | -1.48 | 1.77 | 0.000261 | 0.009195 |
| ENSMUSG00000032125 | *Robo4* | -0.39 | -1.48 | 6.02 | 0.000906 | 0.022006 |
| ENSMUSG00000023951 | *Vegfa* | -0.39 | -1.48 | 8.10 | 0.001093 | 0.025179 |
| ENSMUSG00000027377 | *Mall* | -0.39 | -1.47 | 3.46 | 0.001579 | 0.031987 |
| ENSMUSG00000090698 | *Apold1* | -0.39 | -1.47 | 6.17 | 0.000123 | 0.005393 |
| ENSMUSG00000002043 | *Trappc6a* | -0.38 | -1.46 | 2.57 | 2.61E-06 | 0.000291 |
| ENSMUSG00000032220 | *Myo1e* | -0.38 | -1.46 | 4.98 | 5.87E-07 | 0.000091 |
| ENSMUSG00000029093 | *Sorcs2* | -0.37 | -1.45 | 3.53 | 2.94E-07 | 0.000051 |
| ENSMUSG00000044469 | *Tnfaip8l1* | -0.37 | -1.45 | 2.43 | 0.001834 | 0.035063 |
| ENSMUSG00000021990 | *Spata13* | -0.37 | -1.45 | 5.50 | 1.03E-05 | 0.000824 |
| ENSMUSG00000063060 | *Sox7* | -0.37 | -1.45 | 4.74 | 0.001238 | 0.027526 |
| ENSMUSG00000070315 | *4930581F22Rik* | -0.37 | -1.44 | 1.78 | 0.001518 | 0.030987 |
| ENSMUSG00000040624 | *Plekhg1* | -0.37 | -1.44 | 5.69 | 0.000822 | 0.020519 |
| ENSMUSG00000087497 | *2810001G20Rik* | -0.37 | -1.44 | 1.60 | 0.002711 | 0.045258 |
| ENSMUSG00000032402 | *Smad3* | -0.37 | -1.44 | 4.97 | 5.61E-07 | 0.000088 |
| ENSMUSG00000058173 | *Smco4* | -0.37 | -1.44 | 3.25 | 1.63E-05 | 0.001159 |
| ENSMUSG00000049588 | *Ccdc69* | -0.37 | -1.44 | 2.08 | 0.002833 | 0.046379 |
| ENSMUSG00000030022 | *Adamts9* | -0.37 | -1.44 | 6.30 | 0.00038 | 0.011697 |
| ENSMUSG00000026858 | *Fam73b* | -0.37 | -1.44 | 5.87 | 6.70E-07 | 0.000102 |
| ENSMUSG00000057897 | *Camk2b* | -0.36 | -1.44 | 2.89 | 2.75E-06 | 0.000301 |
| ENSMUSG00000020593 | *Lpin1* | -0.36 | -1.43 | 7.78 | 0.000119 | 0.005289 |
| ENSMUSG00000087543 | *Gm16576* | -0.36 | -1.43 | 2.80 | 0.000148 | 0.006236 |
| ENSMUSG00000051335 | *Gfod1* | -0.35 | -1.42 | 6.02 | 0.000445 | 0.013211 |
| ENSMUSG00000033361 | *Prrg3* | -0.35 | -1.42 | 4.40 | 0.000122 | 0.005370 |
| ENSMUSG00000018427 | *Ypel2* | -0.35 | -1.42 | 4.84 | 2.18E-06 | 0.000258 |
| ENSMUSG00000050271 | *D8Ertd82e* | -0.35 | -1.42 | 4.18 | 0.001889 | 0.035613 |
| ENSMUSG00000031389 | *Arhgap4* | -0.34 | -1.40 | 2.82 | 0.000169 | 0.006905 |
| ENSMUSG00000032846 | *Zswim6* | -0.34 | -1.40 | 4.35 | 3.36E-05 | 0.002018 |
| ENSMUSG00000051124 | *Gimap9* | -0.33 | -1.40 | 1.93 | 0.000931 | 0.022258 |
| ENSMUSG00000029417 | *Cxcl9* | -0.33 | -1.39 | 3.11 | 7.43E-05 | 0.003744 |
| ENSMUSG00000074272 | *Ceacam1* | -0.33 | -1.39 | 3.41 | 0.000254 | 0.009073 |
| ENSMUSG00000042284 | *Itga1* | -0.33 | -1.39 | 6.13 | 0.000106 | 0.004876 |
| ENSMUSG00000020486 | *Sep-04* | -0.33 | -1.39 | 4.70 | 0.000288 | 0.009724 |
| ENSMUSG00000043557 | *Mdga1* | -0.33 | -1.39 | 2.93 | 4.42E-06 | 0.000431 |
| ENSMUSG00000000628 | *Hk2* | -0.32 | -1.38 | 8.40 | 0.000602 | 0.016141 |
| ENSMUSG00000066877 | *Nck2* | -0.32 | -1.38 | 3.97 | 0.00183 | 0.035063 |
| ENSMUSG00000026640 | *Plxna2* | -0.32 | -1.38 | 6.93 | 0.000413 | 0.012502 |
| ENSMUSG00000047867 | *Gimap6* | -0.32 | -1.38 | 5.52 | 4.40E-10 | 0.000000 |
| ENSMUSG00000052496 | *Pkdrej* | -0.32 | -1.38 | 2.99 | 0.000167 | 0.006855 |
| ENSMUSG00000083012 | *Fam220a* | -0.32 | -1.38 | 5.41 | 3.78E-13 | 0.000000 |
| ENSMUSG00000020674 | *Pxdn* | -0.31 | -1.37 | 6.92 | 0.002303 | 0.040617 |
| ENSMUSG00000057751 | *Megf6* | -0.31 | -1.37 | 3.15 | 6.90E-05 | 0.003548 |
| ENSMUSG00000021025 | *Nfkbia* | -0.31 | -1.37 | 5.02 | 1.49E-06 | 0.000185 |
| ENSMUSG00000032035 | *Ets1* | -0.31 | -1.37 | 6.90 | 0.000114 | 0.005140 |
| ENSMUSG00000037754 | *Ppp1r16b* | -0.31 | -1.36 | 4.69 | 0.002347 | 0.041084 |
| ENSMUSG00000046182 | *Gsg1l* | -0.31 | -1.36 | 3.01 | 9.84E-05 | 0.004649 |
| ENSMUSG00000047875 | *Gpr157* | -0.31 | -1.36 | 5.96 | 3.88E-05 | 0.002218 |
| ENSMUSG00000032193 | *Ldlr* | -0.31 | -1.36 | 3.89 | 0.000278 | 0.009529 |
| ENSMUSG00000064342 | *mt-Ti* | -0.30 | -1.36 | 6.92 | 0.001349 | 0.028915 |
| ENSMUSG00000109511 | *Nup62* | -0.30 | -1.35 | 4.17 | 1.21E-09 | 0.000001 |
| ENSMUSG00000044847 | *Lsm11* | -0.30 | -1.35 | 2.93 | 0.001435 | 0.030097 |
| ENSMUSG00000018411 | *Mapt* | -0.30 | -1.35 | 6.13 | 2.10E-05 | 0.001412 |
| ENSMUSG00000020331 | *Hcn2* | -0.30 | -1.35 | 3.71 | 0.002108 | 0.038007 |
| ENSMUSG00000022865 | *Cxadr* | -0.30 | -1.35 | 5.47 | 4.85E-11 | 0.000000 |
| ENSMUSG00000005628 | *Tmod4* | -0.30 | -1.35 | 3.73 | 0.002202 | 0.039219 |
| ENSMUSG00000042500 | *Ago4* | -0.30 | -1.34 | 4.28 | 5.40E-06 | 0.000502 |
| ENSMUSG00000022895 | *Ets2* | -0.30 | -1.34 | 6.79 | 0.00052 | 0.014544 |
| ENSMUSG00000051495 | *Irf2bp2* | -0.29 | -1.34 | 7.02 | 1.34E-06 | 0.000171 |
| ENSMUSG00000028527 | *Ak4* | -0.29 | -1.34 | 4.97 | 9.44E-08 | 0.000019 |
| ENSMUSG00000020121 | *Srgap1* | -0.29 | -1.34 | 3.43 | 7.27E-06 | 0.000609 |
| ENSMUSG00000070462 | *Mesdc1* | -0.29 | -1.34 | 4.60 | 2.60E-05 | 0.001668 |
| ENSMUSG00000029591 | *Ung* | -0.29 | -1.34 | 3.76 | 0.000603 | 0.016157 |
| ENSMUSG00000054715 | *Zscan22* | -0.29 | -1.34 | 2.42 | 0.001267 | 0.027921 |
| ENSMUSG00000064302 | *Clasp1* | -0.29 | -1.34 | 8.81 | 5.14E-06 | 0.000484 |
| ENSMUSG00000058006 | *Mdn1* | -0.29 | -1.33 | 5.81 | 3.39E-05 | 0.002018 |
| ENSMUSG00000044317 | *Gpr4* | -0.29 | -1.33 | 3.22 | 0.000311 | 0.010132 |
| ENSMUSG00000032724 | *Abtb2* | -0.29 | -1.33 | 4.79 | 0.000901 | 0.021960 |
| ENSMUSG00000020935 | *Dcakd* | -0.29 | -1.33 | 4.45 | 3.10E-08 | 0.000008 |
| ENSMUSG00000040270 | *Bach2* | -0.28 | -1.33 | 3.34 | 0.000462 | 0.013611 |
| ENSMUSG00000028184 | *Adgrl2* | -0.28 | -1.33 | 6.80 | 0.000115 | 0.005163 |
| ENSMUSG00000021108 | *Prkch* | -0.28 | -1.32 | 5.24 | 1.87E-05 | 0.001281 |
| ENSMUSG00000025986 | *Slc39a10* | -0.28 | -1.32 | 4.38 | 0.000126 | 0.005507 |
| ENSMUSG00000036155 | *Mgat5* | -0.28 | -1.32 | 5.82 | 7.40E-05 | 0.003740 |
| ENSMUSG00000069893 | *9930111J21Rik1* | -0.28 | -1.32 | 2.78 | 0.002034 | 0.037144 |
| ENSMUSG00000044456 | *Rin3* | -0.28 | -1.32 | 4.69 | 0.000173 | 0.007015 |
| ENSMUSG00000021754 | *Map3k1* | -0.28 | -1.32 | 5.92 | 6.79E-07 | 0.000102 |
| ENSMUSG00000056917 | *Sipa1* | -0.27 | -1.32 | 5.24 | 0.000404 | 0.012304 |
| ENSMUSG00000060187 | *Lrrc10* | -0.27 | -1.32 | 6.76 | 0.00121 | 0.027117 |
| ENSMUSG00000051373 | *Plpp7* | -0.27 | -1.32 | 4.21 | 3.70E-07 | 0.000061 |
| ENSMUSG00000015176 | *Nolc1* | -0.27 | -1.31 | 4.28 | 0.00014 | 0.005936 |
| ENSMUSG00000028164 | *Manba* | -0.27 | -1.31 | 4.77 | 0.000195 | 0.007566 |
| ENSMUSG00000030047 | *Arhgap25* | -0.27 | -1.31 | 3.03 | 0.000951 | 0.022615 |
| ENSMUSG00000027858 | *Tspan2* | -0.27 | -1.31 | 4.45 | 0.000709 | 0.018406 |
| ENSMUSG00000033706 | *Smyd5* | -0.27 | -1.31 | 3.37 | 4.22E-05 | 0.002383 |
| ENSMUSG00000020806 | *Rhbdf2* | -0.27 | -1.31 | 4.38 | 5.84E-05 | 0.003093 |
| ENSMUSG00000026305 | *Lrrfip1* | -0.27 | -1.31 | 6.36 | 0.00144 | 0.030162 |
| ENSMUSG00000028073 | *Pear1* | -0.27 | -1.31 | 4.74 | 0.000678 | 0.017731 |
| ENSMUSG00000061589 | *Dot1l* | -0.27 | -1.30 | 5.91 | 0.002971 | 0.048154 |
| ENSMUSG00000020612 | *Prkar1a* | -0.26 | -1.30 | 9.14 | 2.45E-06 | 0.000280 |
| ENSMUSG00000041235 | *Chd7* | -0.26 | -1.30 | 5.71 | 3.18E-08 | 0.000008 |
| ENSMUSG00000035164 | *Zc3h12c* | -0.26 | -1.30 | 4.72 | 1.03E-06 | 0.000139 |
| ENSMUSG00000027111 | *Itga6* | -0.26 | -1.30 | 7.09 | 0.000374 | 0.011570 |
| ENSMUSG00000032816 | *Igdcc4* | -0.26 | -1.30 | 4.03 | 0.00308 | 0.049366 |
| ENSMUSG00000035891 | *Cerk* | -0.26 | -1.30 | 5.72 | 0.000143 | 0.006075 |
| ENSMUSG00000085385 | *Snhg17* | -0.26 | -1.30 | 2.86 | 0.002027 | 0.037117 |
| ENSMUSG00000054690 | *Emcn* | -0.26 | -1.30 | 5.34 | 0.000967 | 0.022833 |
| ENSMUSG00000039115 | *Itga9* | -0.26 | -1.30 | 6.53 | 0.002147 | 0.038427 |
| ENSMUSG00000064262 | *Gimap8* | -0.26 | -1.30 | 4.20 | 0.000289 | 0.009724 |
| ENSMUSG00000039202 | *Abhd2* | -0.26 | -1.29 | 6.11 | 6.77E-06 | 0.000582 |
| ENSMUSG00000028525 | *Pde4b* | -0.26 | -1.29 | 5.87 | 1.20E-05 | 0.000901 |
| ENSMUSG00000029309 | *Sparcl1* | -0.26 | -1.29 | 8.10 | 0.000928 | 0.022258 |
| ENSMUSG00000032434 | *Cmtm6* | -0.25 | -1.29 | 5.39 | 2.54E-08 | 0.000007 |
| ENSMUSG00000031955 | *Bcar1* | -0.25 | -1.29 | 4.74 | 0.002739 | 0.045629 |
| ENSMUSG00000034206 | *Polq* | -0.25 | -1.29 | 3.70 | 0.000717 | 0.018458 |
| ENSMUSG00000068566 | *Myadm* | -0.25 | -1.29 | 8.01 | 0.000406 | 0.012319 |
| ENSMUSG00000042793 | *Lgr6* | -0.25 | -1.29 | 5.25 | 0.002979 | 0.048183 |
| ENSMUSG00000035900 | *Gramd4* | -0.25 | -1.28 | 5.45 | 5.80E-06 | 0.000518 |
| ENSMUSG00000027669 | *Gnb4* | -0.25 | -1.28 | 4.65 | 4.35E-06 | 0.000427 |
| ENSMUSG00000033149 | *Phldb2* | -0.25 | -1.28 | 5.56 | 0.001 | 0.023545 |
| ENSMUSG00000004665 | *Cnn2* | -0.25 | -1.28 | 5.43 | 0.000178 | 0.007089 |
| ENSMUSG00000022463 | *Srebf2* | -0.25 | -1.28 | 5.06 | 6.79E-06 | 0.000582 |
| ENSMUSG00000020015 | *Cdk17* | -0.24 | -1.28 | 5.22 | 4.28E-05 | 0.002403 |
| ENSMUSG00000000325 | *Arvcf* | -0.24 | -1.28 | 4.12 | 4.94E-07 | 0.000079 |
| ENSMUSG00000032575 | *Manf* | -0.24 | -1.28 | 4.74 | 0.000488 | 0.013998 |
| ENSMUSG00000021109 | *Hif1a* | -0.24 | -1.28 | 6.89 | 6.08E-08 | 0.000013 |
| ENSMUSG00000001995 | *Sipa1l2* | -0.24 | -1.27 | 6.27 | 0.000238 | 0.008631 |
| ENSMUSG00000032232 | *Cgnl1* | -0.24 | -1.27 | 6.07 | 0.002966 | 0.048133 |
| ENSMUSG00000024219 | *Anks1* | -0.24 | -1.27 | 6.31 | 1.67E-05 | 0.001176 |
| ENSMUSG00000020571 | *Pdia6* | -0.24 | -1.27 | 6.01 | 0.000259 | 0.009183 |
| ENSMUSG00000034731 | *Dgkh* | -0.24 | -1.27 | 3.96 | 0.001487 | 0.030644 |
| ENSMUSG00000032624 | *Eml4* | -0.24 | -1.27 | 4.16 | 0.001267 | 0.027921 |
| ENSMUSG00000028990 | *Lzic* | -0.24 | -1.27 | 4.52 | 1.05E-06 | 0.000140 |
| ENSMUSG00000024732 | *Ccdc86* | -0.24 | -1.27 | 3.18 | 0.000211 | 0.007934 |
| ENSMUSG00000029544 | *Cabp1* | -0.24 | -1.27 | 3.22 | 0.000169 | 0.006905 |
| ENSMUSG00000027200 | *Sema6d* | -0.24 | -1.27 | 6.35 | 0.001342 | 0.028915 |
| ENSMUSG00000040596 | *Pogk* | -0.24 | -1.27 | 4.71 | 0.000362 | 0.011317 |
| ENSMUSG00000060862 | *Zbtb40* | -0.24 | -1.27 | 3.89 | 0.001185 | 0.026674 |
| ENSMUSG00000071757 | *Zhx2* | -0.23 | -1.26 | 5.18 | 4.12E-10 | 0.000000 |
| ENSMUSG00000095253 | *Zfp799* | -0.23 | -1.26 | 4.06 | 0.001203 | 0.027002 |
| ENSMUSG00000035735 | *Dagla* | -0.23 | -1.26 | 3.12 | 0.001401 | 0.029827 |
| ENSMUSG00000029673 | *Auts2* | -0.23 | -1.26 | 5.28 | 0.00181 | 0.034977 |
| ENSMUSG00000029312 | *Klhl8* | -0.23 | -1.26 | 3.56 | 0.001428 | 0.030097 |
| ENSMUSG00000020740 | *Gga3* | -0.23 | -1.26 | 4.34 | 1.35E-05 | 0.000984 |
| ENSMUSG00000002233 | *Rhoc* | -0.23 | -1.26 | 6.02 | 0.002764 | 0.045675 |
| ENSMUSG00000030602 | *Pak4* | -0.23 | -1.26 | 2.94 | 0.001039 | 0.024181 |
| ENSMUSG00000027822 | *Slc33a1* | -0.23 | -1.26 | 4.10 | 1.13E-06 | 0.000146 |
| ENSMUSG00000022237 | *Ankrd33b* | -0.23 | -1.26 | 5.67 | 9.21E-05 | 0.004426 |
| ENSMUSG00000021281 | *Tnfaip2* | -0.23 | -1.26 | 5.37 | 0.000531 | 0.014690 |
| ENSMUSG00000029171 | *Pgm1* | -0.23 | -1.25 | 3.28 | 0.000264 | 0.009218 |
| ENSMUSG00000030768 | *Disp1* | -0.23 | -1.25 | 3.83 | 0.000221 | 0.008221 |
| ENSMUSG00000026672 | *Optn* | -0.23 | -1.25 | 6.16 | 3.31E-05 | 0.002012 |
| ENSMUSG00000006219 | *Fblim1* | -0.23 | -1.25 | 7.25 | 0.000299 | 0.009971 |
| ENSMUSG00000056204 | *Pgpep1* | -0.23 | -1.25 | 5.05 | 0.00048 | 0.013878 |
| ENSMUSG00000004677 | *Myo9b* | -0.23 | -1.25 | 6.27 | 0.002367 | 0.041348 |
| ENSMUSG00000006205 | *Htra1* | -0.22 | -1.25 | 5.65 | 1.16E-05 | 0.000887 |
| ENSMUSG00000060935 | *Tmem263* | -0.22 | -1.25 | 4.76 | 7.19E-06 | 0.000608 |
| ENSMUSG00000034525 | *Ice1* | -0.22 | -1.25 | 6.30 | 7.86E-07 | 0.000112 |
| ENSMUSG00000039831 | *Arhgap29* | -0.22 | -1.25 | 7.11 | 8.65E-08 | 0.000018 |
| ENSMUSG00000024276 | *Zfp397* | -0.22 | -1.25 | 5.40 | 0.00171 | 0.033942 |
| ENSMUSG00000028410 | *Dnaja1* | -0.22 | -1.25 | 5.23 | 2.95E-05 | 0.001839 |
| ENSMUSG00000042688 | *Mapk6* | -0.22 | -1.25 | 5.39 | 0.000794 | 0.020034 |
| ENSMUSG00000033960 | *9430020K01Rik* | -0.22 | -1.25 | 7.23 | 0.000812 | 0.020453 |
| ENSMUSG00000018417 | *Myo1b* | -0.22 | -1.25 | 5.77 | 0.000841 | 0.020851 |
| ENSMUSG00000040653 | *Ppp1r14c* | -0.22 | -1.25 | 6.63 | 0.003066 | 0.049244 |
| ENSMUSG00000015342 | *Xk* | -0.22 | -1.24 | 3.47 | 0.001233 | 0.027492 |
| ENSMUSG00000023079 | *Gtf2ird1* | -0.22 | -1.24 | 3.93 | 1.44E-05 | 0.001037 |
| ENSMUSG00000055044 | *Pdlim1* | -0.22 | -1.24 | 5.55 | 0.002649 | 0.044642 |
| ENSMUSG00000057329 | *Bcl2* | -0.22 | -1.24 | 4.57 | 0.00067 | 0.017591 |
| ENSMUSG00000005609 | *Ctr9* | -0.22 | -1.24 | 4.44 | 0.000619 | 0.016488 |
| ENSMUSG00000038143 | *Stox2* | -0.22 | -1.24 | 5.38 | 6.95E-10 | 0.000000 |
| ENSMUSG00000036019 | *Tmtc2* | -0.22 | -1.24 | 3.76 | 0.002581 | 0.043809 |
| ENSMUSG00000027800 | *Tm4sf1* | -0.22 | -1.24 | 6.32 | 0.000921 | 0.022183 |
| ENSMUSG00000062115 | *Rai1* | -0.21 | -1.24 | 5.31 | 0.002504 | 0.042803 |
| ENSMUSG00000010064 | *Slc38a3* | -0.21 | -1.24 | 5.82 | 0.000755 | 0.019165 |
| ENSMUSG00000054364 | *Rhob* | -0.21 | -1.24 | 7.28 | 0.000399 | 0.012178 |
| ENSMUSG00000028796 | *Phc2* | -0.21 | -1.24 | 5.32 | 0.001978 | 0.036496 |
| ENSMUSG00000017376 | *Nlk* | -0.21 | -1.24 | 4.82 | 7.21E-06 | 0.000608 |
| ENSMUSG00000046079 | *Lrrc8d* | -0.21 | -1.23 | 4.13 | 0.000272 | 0.009369 |
| ENSMUSG00000015501 | *Hivep2* | -0.21 | -1.23 | 6.13 | 3.87E-06 | 0.000390 |
| ENSMUSG00000026799 | *Med27* | -0.21 | -1.23 | 3.11 | 0.001833 | 0.035063 |
| ENSMUSG00000048277 | *Syngr2* | -0.21 | -1.23 | 5.97 | 0.00019 | 0.007430 |
| ENSMUSG00000016664 | *Pacsin2* | -0.21 | -1.23 | 7.05 | 9.59E-07 | 0.000130 |
| ENSMUSG00000021559 | *Dapk1* | -0.21 | -1.23 | 5.55 | 1.10E-05 | 0.000859 |
| ENSMUSG00000002845 | *Tmem39a* | -0.21 | -1.23 | 3.72 | 0.00061 | 0.016307 |
| ENSMUSG00000018381 | *Abi3* | -0.20 | -1.23 | 3.98 | 0.001929 | 0.035810 |
| ENSMUSG00000039262 | *Prrc2b* | -0.20 | -1.23 | 7.82 | 0.002033 | 0.037144 |
| ENSMUSG00000025810 | *Nrp1* | -0.20 | -1.23 | 8.04 | 0.000846 | 0.020934 |
| ENSMUSG00000026657 | *Frmd4a* | -0.20 | -1.23 | 5.85 | 0.000537 | 0.014770 |
| ENSMUSG00000030199 | *Etv6* | -0.20 | -1.23 | 5.29 | 0.000102 | 0.004725 |
| ENSMUSG00000046691 | *Chtf8* | -0.20 | -1.23 | 5.11 | 0.000358 | 0.011223 |
| ENSMUSG00000026470 | *Stx6* | -0.20 | -1.22 | 4.63 | 0.000499 | 0.014121 |
| ENSMUSG00000030357 | *Fkbp4* | -0.20 | -1.22 | 7.50 | 0.000443 | 0.013196 |
| ENSMUSG00000002871 | *Tpra1* | -0.20 | -1.22 | 4.49 | 0.000237 | 0.008631 |
| ENSMUSG00000026489 | *Adck3* | -0.20 | -1.22 | 8.44 | 0.000291 | 0.009786 |
| ENSMUSG00000074212 | *Dnajb14* | -0.20 | -1.22 | 5.17 | 1.23E-08 | 0.000004 |
| ENSMUSG00000050953 | *Gja1* | -0.20 | -1.22 | 9.09 | 0.000433 | 0.012955 |
| ENSMUSG00000017679 | *Ttpal* | -0.20 | -1.22 | 4.56 | 0.000735 | 0.018800 |
| ENSMUSG00000067586 | *S1pr3* | -0.19 | -1.22 | 5.08 | 0.002805 | 0.046051 |
| ENSMUSG00000021413 | *Prpf4b* | -0.19 | -1.21 | 5.64 | 1.88E-05 | 0.001284 |
| ENSMUSG00000001089 | *Luzp1* | -0.19 | -1.21 | 6.54 | 0.001667 | 0.033455 |
| ENSMUSG00000017999 | *Ddx27* | -0.19 | -1.21 | 3.69 | 0.000824 | 0.020535 |
| ENSMUSG00000027248 | *Pdia3* | -0.19 | -1.21 | 7.12 | 0.000324 | 0.010478 |
| ENSMUSG00000027722 | *Spata5* | -0.19 | -1.21 | 3.72 | 0.002532 | 0.043227 |
| ENSMUSG00000042605 | *Atxn2* | -0.19 | -1.21 | 6.36 | 4.62E-05 | 0.002531 |
| ENSMUSG00000035954 | *Dock4* | -0.19 | -1.21 | 5.52 | 2.74E-05 | 0.001732 |
| ENSMUSG00000030315 | *Vgll4* | -0.19 | -1.21 | 4.74 | 2.46E-05 | 0.001605 |
| ENSMUSG00000028034 | *Fubp1* | -0.19 | -1.21 | 6.45 | 4.57E-05 | 0.002523 |
| ENSMUSG00000032059 | *Alg9* | -0.19 | -1.21 | 3.26 | 0.00271 | 0.045258 |
| ENSMUSG00000040511 | *Pvr* | -0.19 | -1.20 | 5.29 | 6.24E-05 | 0.003256 |
| ENSMUSG00000003868 | *Ruvbl2* | -0.19 | -1.20 | 3.85 | 0.000254 | 0.009073 |
| ENSMUSG00000063888 | *Rpl7l1* | -0.18 | -1.20 | 5.20 | 5.79E-06 | 0.000518 |
| ENSMUSG00000070730 | *Rmdn3* | -0.18 | -1.20 | 3.61 | 0.000821 | 0.020519 |
| ENSMUSG00000022403 | *St13* | -0.18 | -1.20 | 6.52 | 3.94E-07 | 0.000065 |
| ENSMUSG00000068270 | *Shroom4* | -0.18 | -1.20 | 5.89 | 0.002093 | 0.037867 |
| ENSMUSG00000018363 | *Smurf2* | -0.18 | -1.20 | 6.12 | 0.002381 | 0.041514 |
| ENSMUSG00000025995 | *Wdr75* | -0.18 | -1.20 | 4.18 | 0.000177 | 0.007075 |
| ENSMUSG00000039410 | *Prdm16* | -0.18 | -1.20 | 4.52 | 0.002459 | 0.042615 |
| ENSMUSG00000042508 | *Dmtf1* | -0.18 | -1.20 | 4.69 | 0.000129 | 0.005630 |
| ENSMUSG00000037750 | *Fam222b* | -0.18 | -1.20 | 3.91 | 0.000251 | 0.009049 |
| ENSMUSG00000058013 | *Sep-11* | -0.18 | -1.20 | 6.30 | 0.001348 | 0.028915 |
| ENSMUSG00000029701 | *Rbm28* | -0.18 | -1.20 | 4.83 | 7.30E-07 | 0.000107 |
| ENSMUSG00000018974 | *Sart3* | -0.18 | -1.20 | 4.03 | 0.002752 | 0.045675 |
| ENSMUSG00000031749 | *St3gal2* | -0.18 | -1.20 | 5.25 | 0.000321 | 0.010400 |
| ENSMUSG00000031681 | *Smad1* | -0.18 | -1.20 | 4.79 | 0.002565 | 0.043581 |
| ENSMUSG00000031864 | *Ints10* | -0.18 | -1.20 | 4.59 | 0.001457 | 0.030284 |
| ENSMUSG00000074457 | *S100a16* | -0.18 | -1.20 | 4.74 | 2.14E-05 | 0.001426 |
| ENSMUSG00000038807 | *Rap1gap2* | -0.18 | -1.20 | 6.37 | 0.001099 | 0.025229 |
| ENSMUSG00000039316 | *Rftn1* | -0.18 | -1.20 | 5.39 | 0.001329 | 0.028798 |
| ENSMUSG00000041225 | *Arhgap12* | -0.18 | -1.20 | 5.35 | 2.64E-05 | 0.001684 |
| ENSMUSG00000032244 | *Fem1b* | -0.18 | -1.19 | 5.55 | 0.000579 | 0.015632 |
| ENSMUSG00000067150 | *Xpo5* | -0.18 | -1.19 | 4.82 | 1.09E-06 | 0.000143 |
| ENSMUSG00000026932 | *Nacc2* | -0.18 | -1.19 | 5.41 | 6.16E-05 | 0.003241 |
| ENSMUSG00000033285 | *Wdr3* | -0.18 | -1.19 | 3.96 | 0.000309 | 0.010132 |
| ENSMUSG00000047466 | *8030462N17Rik* | -0.18 | -1.19 | 5.20 | 3.67E-05 | 0.002122 |
| ENSMUSG00000071172 | *Srsf3* | -0.18 | -1.19 | 6.04 | 0.000529 | 0.014663 |
| ENSMUSG00000039242 | *B3galnt2* | -0.18 | -1.19 | 4.80 | 0.000342 | 0.010809 |
| ENSMUSG00000032352 | *Lrrc1* | -0.17 | -1.19 | 4.17 | 0.001683 | 0.033676 |
| ENSMUSG00000038387 | *Rras* | -0.17 | -1.19 | 4.46 | 0.000529 | 0.014663 |
| ENSMUSG00000023809 | *Rps6ka2* | -0.17 | -1.19 | 5.43 | 0.000914 | 0.022140 |
| ENSMUSG00000074305 | *Peak1* | -0.17 | -1.19 | 6.76 | 0.000337 | 0.010746 |
| ENSMUSG00000034485 | *Uaca* | -0.17 | -1.19 | 6.35 | 5.72E-05 | 0.003065 |
| ENSMUSG00000021892 | *Sh3bp5* | -0.17 | -1.19 | 5.43 | 0.001707 | 0.033931 |
| ENSMUSG00000037253 | *Mex3c* | -0.17 | -1.19 | 4.75 | 0.001858 | 0.035405 |
| ENSMUSG00000035152 | *Ap2b1* | -0.17 | -1.19 | 6.21 | 0.00012 | 0.005303 |
| ENSMUSG00000038025 | *Phf2* | -0.17 | -1.19 | 5.55 | 0.001425 | 0.030097 |
| ENSMUSG00000013698 | *Pea15a* | -0.17 | -1.18 | 6.33 | 0.001971 | 0.036460 |
| ENSMUSG00000053477 | *Tcf4* | -0.17 | -1.18 | 8.04 | 0.000405 | 0.012319 |
| ENSMUSG00000026626 | *Ppp2r5a* | -0.17 | -1.18 | 6.43 | 0.001411 | 0.029934 |
| ENSMUSG00000072235 | *Tuba1a* | -0.17 | -1.18 | 5.35 | 0.003043 | 0.048989 |
| ENSMUSG00000028630 | *Dyrk2* | -0.17 | -1.18 | 4.41 | 0.001679 | 0.033646 |
| ENSMUSG00000020823 | *Sec14l1* | -0.17 | -1.18 | 6.81 | 0.002328 | 0.040956 |
| ENSMUSG00000026074 | *Map4k4* | -0.17 | -1.18 | 7.56 | 0.000304 | 0.010074 |
| ENSMUSG00000057637 | *Prdm2* | -0.17 | -1.18 | 5.91 | 3.73E-05 | 0.002143 |
| ENSMUSG00000025958 | *Creb1* | -0.17 | -1.18 | 5.61 | 0.001069 | 0.024775 |
| ENSMUSG00000033955 | *Tnks1bp1* | -0.17 | -1.18 | 6.09 | 0.001974 | 0.036460 |
| ENSMUSG00000020716 | *Nf1* | -0.17 | -1.18 | 6.71 | 0.001525 | 0.031032 |
| ENSMUSG00000024622 | *Hmgxb3* | -0.16 | -1.18 | 4.79 | 0.001688 | 0.033735 |
| ENSMUSG00000011877 | *Git1* | -0.16 | -1.18 | 5.91 | 0.000359 | 0.011242 |
| ENSMUSG00000071650 | *Ganab* | -0.16 | -1.18 | 6.08 | 0.001413 | 0.029945 |
| ENSMUSG00000070544 | *Top1* | -0.16 | -1.18 | 6.08 | 0.000203 | 0.007751 |
| ENSMUSG00000055065 | *Ddx17* | -0.16 | -1.18 | 7.31 | 0.000216 | 0.008070 |
| ENSMUSG00000058881 | *Zfp516* | -0.16 | -1.18 | 4.70 | 0.001435 | 0.030097 |
| ENSMUSG00000028163 | *Nfkb1* | -0.16 | -1.18 | 6.03 | 0.001719 | 0.034030 |
| ENSMUSG00000044452 | *Zfp507* | -0.16 | -1.17 | 4.54 | 0.001442 | 0.030166 |
| ENSMUSG00000029833 | *Trim24* | -0.16 | -1.17 | 5.74 | 0.001101 | 0.025229 |
| ENSMUSG00000038205 | *Prkab2* | -0.16 | -1.17 | 5.70 | 0.000113 | 0.005100 |
| ENSMUSG00000034342 | *Cbl* | -0.16 | -1.17 | 6.26 | 0.000596 | 0.016018 |
| ENSMUSG00000015937 | *H2afy* | -0.16 | -1.17 | 5.01 | 0.000469 | 0.013693 |
| ENSMUSG00000021311 | *Mtr* | -0.16 | -1.17 | 6.55 | 0.000528 | 0.014663 |
| ENSMUSG00000029528 | *Pxn* | -0.16 | -1.17 | 5.77 | 0.000418 | 0.012624 |
| ENSMUSG00000033799 | *Fam208b* | -0.16 | -1.17 | 5.49 | 1.99E-06 | 0.000237 |
| ENSMUSG00000045962 | *Wnk1* | -0.16 | -1.17 | 9.71 | 0.000711 | 0.018406 |
| ENSMUSG00000050567 | *Maml1* | -0.16 | -1.17 | 4.62 | 0.001021 | 0.023927 |
| ENSMUSG00000024054 | *Smchd1* | -0.16 | -1.17 | 5.82 | 0.001746 | 0.034179 |
| ENSMUSG00000026499 | *Acbd3* | -0.15 | -1.17 | 5.19 | 0.000476 | 0.013802 |
| ENSMUSG00000049658 | *Bdp1* | -0.15 | -1.17 | 5.87 | 0.001463 | 0.030354 |
| ENSMUSG00000014426 | *Map3k4* | -0.15 | -1.17 | 5.75 | 0.000245 | 0.008874 |
| ENSMUSG00000020114 | *Cand1* | -0.15 | -1.16 | 5.71 | 0.000465 | 0.013634 |
| ENSMUSG00000032329 | *Hmg20a* | -0.15 | -1.16 | 4.74 | 0.001464 | 0.030354 |
| ENSMUSG00000021395 | *Spin1* | -0.15 | -1.16 | 5.80 | 0.000683 | 0.017796 |
| ENSMUSG00000028530 | *Jak1* | -0.15 | -1.16 | 7.37 | 8.69E-05 | 0.004229 |
| ENSMUSG00000053819 | *Camk2d* | -0.15 | -1.16 | 7.57 | 0.001626 | 0.032776 |
| ENSMUSG00000018547 | *Pip4k2b* | -0.15 | -1.16 | 5.40 | 3.38E-05 | 0.002018 |
| ENSMUSG00000034928 | *Rnf44* | -0.15 | -1.16 | 5.50 | 0.002246 | 0.039849 |
| ENSMUSG00000028343 | *Erp44* | -0.15 | -1.16 | 4.78 | 0.000872 | 0.021470 |
| ENSMUSG00000032366 | *Tpm1* | -0.14 | -1.16 | 12.04 | 0.001037 | 0.024181 |
| ENSMUSG00000058070 | *Eml1* | -0.14 | -1.16 | 6.18 | 0.000898 | 0.021938 |
| ENSMUSG00000037876 | *Jmjd1c* | -0.14 | -1.15 | 6.89 | 0.000543 | 0.014892 |
| ENSMUSG00000063317 | *Usp31* | -0.14 | -1.15 | 5.19 | 0.002218 | 0.039412 |
| ENSMUSG00000032116 | *Stt3a* | -0.14 | -1.15 | 5.94 | 0.000567 | 0.015351 |
| ENSMUSG00000020611 | *Gna13* | -0.14 | -1.15 | 6.10 | 0.000137 | 0.005855 |
| ENSMUSG00000017615 | *Tnfaip1* | -0.14 | -1.15 | 6.47 | 0.000132 | 0.005711 |
| ENSMUSG00000026889 | *Rbm18* | -0.14 | -1.15 | 5.07 | 0.001145 | 0.026096 |
| ENSMUSG00000034557 | *Zfyve9* | -0.14 | -1.15 | 5.89 | 0.000147 | 0.006226 |
| ENSMUSG00000031441 | *Atp11a* | -0.14 | -1.15 | 7.19 | 0.000614 | 0.016381 |
| ENSMUSG00000056234 | *Ncoa4* | -0.14 | -1.15 | 4.59 | 0.00281 | 0.046059 |
| ENSMUSG00000037331 | *Larp1* | -0.14 | -1.15 | 6.91 | 0.000924 | 0.022234 |
| ENSMUSG00000024811 | *Tnks2* | -0.14 | -1.15 | 7.11 | 2.96E-05 | 0.001839 |
| ENSMUSG00000034613 | *Ppm1h* | -0.14 | -1.15 | 4.54 | 0.000512 | 0.014375 |
| ENSMUSG00000030275 | *Etnk1* | -0.14 | -1.15 | 6.43 | 5.18E-06 | 0.000484 |
| ENSMUSG00000006932 | *Ctnnb1* | -0.14 | -1.15 | 8.33 | 9.03E-05 | 0.004365 |
| ENSMUSG00000020290 | *Xpo1* | -0.14 | -1.15 | 6.41 | 2.48E-05 | 0.001607 |
| ENSMUSG00000020189 | *Osbpl8* | -0.14 | -1.15 | 7.45 | 9.98E-05 | 0.004683 |
| ENSMUSG00000024981 | *Acsl5* | -0.14 | -1.15 | 4.80 | 0.000279 | 0.009529 |
| ENSMUSG00000020919 | *Stat5b* | -0.14 | -1.14 | 5.92 | 0.002496 | 0.042791 |
| ENSMUSG00000039000 | *Ube3c* | -0.13 | -1.14 | 6.29 | 0.000199 | 0.007662 |
| ENSMUSG00000057551 | *Zfp317* | -0.13 | -1.14 | 4.75 | 0.001518 | 0.030987 |
| ENSMUSG00000022185 | *Acin1* | -0.13 | -1.14 | 6.39 | 9.57E-05 | 0.004565 |
| ENSMUSG00000040565 | *Btaf1* | -0.13 | -1.14 | 5.46 | 0.002806 | 0.046051 |
| ENSMUSG00000000915 | *Hip1r* | -0.13 | -1.14 | 4.72 | 0.002058 | 0.037440 |
| ENSMUSG00000037933 | *Bicd2* | -0.13 | -1.14 | 6.87 | 0.002697 | 0.045235 |
| ENSMUSG00000034761 | *Map4k5* | -0.13 | -1.14 | 5.53 | 0.00259 | 0.043851 |
| ENSMUSG00000022967 | *Ifnar1* | -0.13 | -1.13 | 5.39 | 0.00211 | 0.038007 |
| ENSMUSG00000049791 | *Fzd4* | -0.13 | -1.13 | 6.47 | 4.51E-05 | 0.002499 |
| ENSMUSG00000027598 | *Itch* | -0.13 | -1.13 | 5.79 | 0.00037 | 0.011505 |
| ENSMUSG00000030007 | *Cct7* | -0.12 | -1.13 | 6.48 | 0.000206 | 0.007798 |
| ENSMUSG00000040945 | *Rcc2* | -0.12 | -1.13 | 4.76 | 0.002172 | 0.038777 |
| ENSMUSG00000022136 | *Dnajc3* | -0.12 | -1.13 | 6.11 | 0.001753 | 0.034179 |
| ENSMUSG00000045576 | *St7l* | -0.12 | -1.13 | 4.61 | 0.00175 | 0.034179 |
| ENSMUSG00000003033 | *Ap1m1* | -0.12 | -1.13 | 4.92 | 0.001707 | 0.033931 |
| ENSMUSG00000021327 | *Zkscan3* | -0.12 | -1.13 | 5.73 | 0.001241 | 0.027548 |
| ENSMUSG00000030447 | *Cyfip1* | -0.12 | -1.12 | 6.23 | 0.001604 | 0.032423 |
| ENSMUSG00000021693 | *Kif2a* | -0.11 | -1.12 | 4.90 | 0.002022 | 0.037062 |
| ENSMUSG00000021111 | *Papola* | -0.11 | -1.12 | 6.83 | 0.001091 | 0.025179 |
| ENSMUSG00000000194 | *Gpr107* | -0.11 | -1.12 | 5.74 | 0.000914 | 0.022140 |
| ENSMUSG00000032536 | *Trak1* | -0.11 | -1.12 | 8.34 | 0.001036 | 0.024181 |
| ENSMUSG00000053110 | *Yap1* | -0.11 | -1.12 | 6.80 | 9.75E-05 | 0.004622 |
| ENSMUSG00000037503 | *Fam168b* | -0.11 | -1.12 | 6.10 | 0.002714 | 0.045258 |
| ENSMUSG00000029169 | *Dhx15* | -0.11 | -1.11 | 6.39 | 0.000267 | 0.009252 |
| ENSMUSG00000041846 | *Smek1* | -0.11 | -1.11 | 5.42 | 0.000752 | 0.019109 |
| ENSMUSG00000029501 | *Ankle2* | -0.11 | -1.11 | 5.52 | 0.001643 | 0.033024 |
| ENSMUSG00000024241 | *Sos1* | -0.10 | -1.11 | 5.25 | 0.001072 | 0.024800 |
| ENSMUSG00000032481 | *Smarcc1* | -0.10 | -1.11 | 5.42 | 0.001868 | 0.035556 |
| ENSMUSG00000037926 | *Ssh2* | -0.10 | -1.11 | 7.37 | 0.002383 | 0.041514 |
| ENSMUSG00000023175 | *Bsg* | -0.10 | -1.11 | 9.34 | 0.000332 | 0.010638 |
| ENSMUSG00000040029 | *Ipo8* | -0.10 | -1.10 | 5.63 | 0.000497 | 0.014121 |
| ENSMUSG00000040865 | *Ino80d* | -0.08 | -1.09 | 6.09 | 0.002294 | 0.040505 |
| ENSMUSG00000022108 | *Itm2b* | 0.09 | 1.10 | 8.60 | 0.000816 | 0.020491 |
| ENSMUSG00000019132 | *BC005537* | 0.09 | 1.10 | 7.78 | 0.001484 | 0.030631 |
| ENSMUSG00000002107 | *Celf2* | 0.10 | 1.10 | 6.78 | 0.002976 | 0.048178 |
| ENSMUSG00000026176 | *Ctdsp1* | 0.10 | 1.11 | 6.22 | 0.001489 | 0.030644 |
| ENSMUSG00000032355 | *Mlip* | 0.11 | 1.12 | 6.69 | 0.002585 | 0.043825 |
| ENSMUSG00000032046 | *Abhd12* | 0.12 | 1.13 | 5.12 | 0.000658 | 0.017397 |
| ENSMUSG00000061751 | *Kalrn* | 0.13 | 1.14 | 5.21 | 0.000663 | 0.017457 |
| ENSMUSG00000008036 | *Ap2s1* | 0.13 | 1.14 | 4.41 | 0.00189 | 0.035613 |
| ENSMUSG00000025337 | *Sbds* | 0.13 | 1.14 | 4.81 | 0.001916 | 0.035675 |
| ENSMUSG00000018820 | *Zfyve27* | 0.14 | 1.14 | 4.52 | 0.000876 | 0.021545 |
| ENSMUSG00000022641 | *Bbx* | 0.14 | 1.15 | 5.51 | 0.000564 | 0.015330 |
| ENSMUSG00000020190 | *Mknk2* | 0.14 | 1.15 | 7.46 | 0.001456 | 0.030284 |
| ENSMUSG00000021589 | *Rhobtb3* | 0.14 | 1.15 | 4.37 | 0.002262 | 0.040082 |
| ENSMUSG00000031666 | *Rbl2* | 0.15 | 1.16 | 5.71 | 0.001094 | 0.025179 |
| ENSMUSG00000033701 | *Acbd6* | 0.15 | 1.16 | 4.11 | 0.001816 | 0.034978 |
| ENSMUSG00000039701 | *Usp53* | 0.15 | 1.16 | 4.20 | 0.000747 | 0.019088 |
| ENSMUSG00000022008 | *Gpalpp1* | 0.15 | 1.16 | 4.86 | 0.001726 | 0.034116 |
| ENSMUSG00000003235 | *Eif2b5* | 0.15 | 1.16 | 5.48 | 0.000274 | 0.009414 |
| ENSMUSG00000022261 | *Sdc2* | 0.15 | 1.17 | 5.58 | 9.42E-07 | 0.000129 |
| ENSMUSG00000034480 | *Diaph2* | 0.16 | 1.17 | 5.64 | 0.000714 | 0.018431 |
| ENSMUSG00000051390 | *Zbtb22* | 0.16 | 1.17 | 4.11 | 0.000573 | 0.015485 |
| ENSMUSG00000032127 | *Vps11* | 0.16 | 1.17 | 4.51 | 0.000828 | 0.020617 |
| ENSMUSG00000021607 | *Mrpl36* | 0.16 | 1.17 | 3.97 | 0.001757 | 0.034215 |
| ENSMUSG00000006494 | *Pdk1* | 0.16 | 1.17 | 7.57 | 8.27E-05 | 0.004052 |
| ENSMUSG00000022505 | *Emp2* | 0.16 | 1.17 | 5.62 | 0.002937 | 0.047770 |
| ENSMUSG00000078429 | *Ctdsp2* | 0.16 | 1.17 | 6.84 | 1.27E-05 | 0.000939 |
| ENSMUSG00000026510 | *Trp53bp2* | 0.16 | 1.17 | 3.81 | 0.001346 | 0.028915 |
| ENSMUSG00000000532 | *Acvr1b* | 0.16 | 1.18 | 4.40 | 0.000888 | 0.021735 |
| ENSMUSG00000027371 | *Fahd2a* | 0.16 | 1.18 | 3.77 | 0.002838 | 0.046416 |
| ENSMUSG00000035967 | *Ddx26b* | 0.16 | 1.18 | 4.31 | 0.001301 | 0.028453 |
| ENSMUSG00000020228 | *Helb* | 0.16 | 1.18 | 4.11 | 0.000455 | 0.013498 |
| ENSMUSG00000026102 | *Inpp1* | 0.16 | 1.18 | 4.59 | 0.00082 | 0.020519 |
| ENSMUSG00000019929 | *Dcn* | 0.16 | 1.18 | 8.45 | 0.000308 | 0.010132 |
| ENSMUSG00000022419 | *Deptor* | 0.17 | 1.18 | 4.39 | 0.000178 | 0.007099 |
| ENSMUSG00000031295 | *Phka2* | 0.17 | 1.18 | 4.69 | 0.001267 | 0.027921 |
| ENSMUSG00000029103 | *Lrpap1* | 0.17 | 1.18 | 5.27 | 4.07E-06 | 0.000402 |
| ENSMUSG00000045613 | *Chrm2* | 0.17 | 1.18 | 7.30 | 0.000498 | 0.014121 |
| ENSMUSG00000032180 | *Tmed1* | 0.17 | 1.18 | 4.26 | 0.002486 | 0.042772 |
| ENSMUSG00000029328 | *Hnrnpdl* | 0.17 | 1.18 | 6.23 | 0.000102 | 0.004739 |
| ENSMUSG00000021846 | *Peli2* | 0.17 | 1.19 | 3.95 | 0.000329 | 0.010578 |
| ENSMUSG00000109901 | *Chmp1b* | 0.17 | 1.19 | 4.58 | 0.000267 | 0.009252 |
| ENSMUSG00000036372 | *Tmem258* | 0.17 | 1.19 | 4.73 | 0.000235 | 0.008563 |
| ENSMUSG00000035024 | *Ncapd3* | 0.17 | 1.19 | 4.51 | 0.001494 | 0.030714 |
| ENSMUSG00000020530 | *Ggnbp2* | 0.17 | 1.19 | 5.78 | 0.00068 | 0.017760 |
| ENSMUSG00000029683 | *Lmod2* | 0.17 | 1.19 | 8.96 | 0.000487 | 0.013998 |
| ENSMUSG00000024228 | *Nudt12* | 0.18 | 1.19 | 4.07 | 0.002655 | 0.044698 |
| ENSMUSG00000049709 | *Nlrp10* | 0.18 | 1.19 | 4.67 | 0.002889 | 0.047200 |
| ENSMUSG00000019878 | *Hsf2* | 0.18 | 1.20 | 3.97 | 0.001281 | 0.028127 |
| ENSMUSG00000041328 | *Pcf11* | 0.18 | 1.20 | 5.83 | 0.001905 | 0.035675 |
| ENSMUSG00000047045 | *Tmem164* | 0.18 | 1.20 | 5.67 | 7.23E-05 | 0.003683 |
| ENSMUSG00000042903 | *Foxo4* | 0.18 | 1.20 | 5.36 | 6.56E-06 | 0.000569 |
| ENSMUSG00000040562 | *Gstm2* | 0.18 | 1.20 | 5.50 | 0.00234 | 0.041049 |
| ENSMUSG00000039745 | *Htatip2* | 0.18 | 1.20 | 3.47 | 0.001693 | 0.033797 |
| ENSMUSG00000003992 | *Ssbp2* | 0.18 | 1.20 | 4.44 | 0.000528 | 0.014663 |
| ENSMUSG00000030161 | *Gabarapl1* | 0.18 | 1.20 | 6.14 | 0.000904 | 0.022001 |
| ENSMUSG00000025656 | *Arhgef9* | 0.19 | 1.20 | 4.25 | 0.001339 | 0.028915 |
| ENSMUSG00000019066 | *Rab3d* | 0.19 | 1.20 | 4.21 | 0.00075 | 0.019105 |
| ENSMUSG00000038332 | *Sesn1* | 0.19 | 1.21 | 6.34 | 0.00151 | 0.030915 |
| ENSMUSG00000045838 | *A430105I19Rik* | 0.19 | 1.21 | 5.66 | 0.000215 | 0.008059 |
| ENSMUSG00000052920 | *Prkg1* | 0.19 | 1.21 | 4.15 | 0.000958 | 0.022707 |
| ENSMUSG00000018166 | *Erbb3* | 0.19 | 1.21 | 4.09 | 0.002488 | 0.042772 |
| ENSMUSG00000033863 | *Klf9* | 0.19 | 1.21 | 6.64 | 0.002178 | 0.038840 |
| ENSMUSG00000032009 | *Sesn3* | 0.19 | 1.21 | 5.12 | 1.30E-05 | 0.000953 |
| ENSMUSG00000021811 | *Dnajc9* | 0.19 | 1.21 | 3.10 | 0.002794 | 0.046003 |
| ENSMUSG00000022507 | *1810013L24Rik* | 0.19 | 1.21 | 6.23 | 1.27E-05 | 0.000939 |
| ENSMUSG00000020385 | *Clk4* | 0.19 | 1.21 | 5.18 | 2.37E-05 | 0.001557 |
| ENSMUSG00000002948 | *Map2k7* | 0.20 | 1.22 | 4.97 | 0.000229 | 0.008426 |
| ENSMUSG00000070867 | *Trabd2b* | 0.20 | 1.22 | 7.15 | 0.000348 | 0.010981 |
| ENSMUSG00000033429 | *Mcee* | 0.20 | 1.22 | 4.93 | 0.00094 | 0.022395 |
| ENSMUSG00000022617 | *Chkb* | 0.20 | 1.22 | 4.81 | 5.72E-06 | 0.000518 |
| ENSMUSG00000023927 | *Satb1* | 0.20 | 1.22 | 4.77 | 0.001873 | 0.035596 |
| ENSMUSG00000053965 | *Pde5a* | 0.20 | 1.22 | 3.02 | 0.002676 | 0.044987 |
| ENSMUSG00000059456 | *Ptk2b* | 0.20 | 1.22 | 4.18 | 0.001736 | 0.034179 |
| ENSMUSG00000028884 | *Rpa2* | 0.20 | 1.22 | 3.02 | 0.002605 | 0.044052 |
| ENSMUSG00000026014 | *Raph1* | 0.20 | 1.22 | 7.78 | 0.000267 | 0.009252 |
| ENSMUSG00000021215 | *Net1* | 0.20 | 1.22 | 3.44 | 0.000341 | 0.010809 |
| ENSMUSG00000028995 | *Fam126a* | 0.20 | 1.23 | 5.98 | 0.001895 | 0.035613 |
| ENSMUSG00000056666 | *Retsat* | 0.20 | 1.23 | 5.21 | 0.00011 | 0.005050 |
| ENSMUSG00000006221 | *Hspb7* | 0.21 | 1.23 | 10.54 | 0.002471 | 0.042641 |
| ENSMUSG00000069601 | *Ank3* | 0.21 | 1.23 | 6.65 | 0.000201 | 0.007710 |
| ENSMUSG00000055799 | *Tcf7l1* | 0.21 | 1.23 | 3.27 | 0.000562 | 0.015317 |
| ENSMUSG00000057315 | *Arhgap24* | 0.21 | 1.23 | 4.19 | 0.000194 | 0.007541 |
| ENSMUSG00000087260 | *Lamtor5* | 0.21 | 1.24 | 4.52 | 0.002749 | 0.045675 |
| ENSMUSG00000034853 | *Acot11* | 0.21 | 1.24 | 4.65 | 0.00012 | 0.005303 |
| ENSMUSG00000002108 | *Nr1h3* | 0.21 | 1.24 | 3.79 | 7.79E-05 | 0.003896 |
| ENSMUSG00000029802 | *Abcg2* | 0.21 | 1.24 | 5.08 | 0.000702 | 0.018246 |
| ENSMUSG00000052253 | *Zfp622* | 0.22 | 1.24 | 4.03 | 3.21E-06 | 0.000342 |
| ENSMUSG00000042178 | *Armc5* | 0.22 | 1.24 | 3.57 | 0.000181 | 0.007165 |
| ENSMUSG00000096957 | *E230013L22Rik* | 0.22 | 1.24 | 3.91 | 0.002461 | 0.042615 |
| ENSMUSG00000019822 | *Smpd2* | 0.22 | 1.24 | 4.18 | 0.003027 | 0.048842 |
| ENSMUSG00000005836 | *Gata6* | 0.22 | 1.24 | 5.70 | 7.13E-06 | 0.000608 |
| ENSMUSG00000023707 | *Ogfod2* | 0.22 | 1.24 | 3.05 | 0.001432 | 0.030097 |
| ENSMUSG00000028496 | *Mllt3* | 0.22 | 1.24 | 4.32 | 0.000918 | 0.022162 |
| ENSMUSG00000042363 | *Lgalsl* | 0.22 | 1.25 | 5.09 | 8.43E-07 | 0.000118 |
| ENSMUSG00000062980 | *Cped1* | 0.22 | 1.25 | 5.23 | 0.000281 | 0.009529 |
| ENSMUSG00000022827 | *Rabl3* | 0.22 | 1.25 | 2.87 | 0.001331 | 0.028801 |
| ENSMUSG00000038086 | *Hspb2* | 0.22 | 1.25 | 4.78 | 0.000395 | 0.012095 |
| ENSMUSG00000024121 | *Atp6v0c* | 0.22 | 1.25 | 3.49 | 0.000311 | 0.010132 |
| ENSMUSG00000042961 | *Egflam* | 0.22 | 1.25 | 3.32 | 0.002213 | 0.039359 |
| ENSMUSG00000025358 | *Cdk2* | 0.23 | 1.25 | 3.22 | 0.000311 | 0.010132 |
| ENSMUSG00000028399 | *Ptprd* | 0.23 | 1.25 | 4.40 | 0.000262 | 0.009208 |
| ENSMUSG00000026721 | *Rabgap1l* | 0.23 | 1.25 | 6.11 | 0.00033 | 0.010578 |
| ENSMUSG00000034810 | *Scn7a* | 0.23 | 1.25 | 5.88 | 8.16E-06 | 0.000672 |
| ENSMUSG00000030101 | *Sumf1* | 0.23 | 1.25 | 4.02 | 5.95E-05 | 0.003140 |
| ENSMUSG00000045103 | *Dmd* | 0.23 | 1.26 | 8.56 | 0.001065 | 0.024707 |
| ENSMUSG00000020265 | *Sumo3* | 0.23 | 1.26 | 5.33 | 5.51E-07 | 0.000087 |
| ENSMUSG00000002409 | *Dyrk1b* | 0.23 | 1.26 | 4.04 | 6.98E-05 | 0.003581 |
| ENSMUSG00000073535 | *Gm5532* | 0.23 | 1.26 | 5.82 | 0.001161 | 0.026229 |
| ENSMUSG00000024101 | *Wash1* | 0.23 | 1.26 | 3.76 | 0.002546 | 0.043357 |
| ENSMUSG00000040111 | *Gramd1b* | 0.23 | 1.26 | 5.22 | 4.03E-05 | 0.002296 |
| ENSMUSG00000002996 | *Hbp1* | 0.23 | 1.26 | 6.13 | 3.57E-06 | 0.000373 |
| ENSMUSG00000027378 | *Nphp1* | 0.24 | 1.27 | 3.12 | 0.000201 | 0.007710 |
| ENSMUSG00000028693 | *Nasp* | 0.24 | 1.27 | 3.87 | 0.001508 | 0.030911 |
| ENSMUSG00000026187 | *Xrcc5* | 0.24 | 1.27 | 4.25 | 0.002091 | 0.037867 |
| ENSMUSG00000039485 | *Tspyl4* | 0.24 | 1.27 | 4.29 | 9.61E-06 | 0.000778 |
| ENSMUSG00000039782 | *Cpeb2* | 0.24 | 1.27 | 5.87 | 0.00028 | 0.009529 |
| ENSMUSG00000028047 | *Thbs3* | 0.24 | 1.27 | 2.89 | 0.001316 | 0.028646 |
| ENSMUSG00000026098 | *Pms1* | 0.24 | 1.27 | 2.91 | 0.000495 | 0.014121 |
| ENSMUSG00000024548 | *Setbp1* | 0.24 | 1.27 | 4.47 | 0.001323 | 0.028757 |
| ENSMUSG00000023885 | *Thbs2* | 0.24 | 1.27 | 4.90 | 0.002702 | 0.045258 |
| ENSMUSG00000033910 | *Gucy1a3* | 0.24 | 1.27 | 4.71 | 0.002768 | 0.045684 |
| ENSMUSG00000050628 | *Ubald2* | 0.24 | 1.27 | 2.94 | 0.002963 | 0.048133 |
| ENSMUSG00000018841 | *Rad51d* | 0.24 | 1.27 | 3.37 | 0.001581 | 0.031987 |
| ENSMUSG00000027834 | *Serpini1* | 0.24 | 1.27 | 3.27 | 0.002414 | 0.041957 |
| ENSMUSG00000006050 | *Sra1* | 0.24 | 1.27 | 5.11 | 1.16E-08 | 0.000004 |
| ENSMUSG00000024660 | *Incenp* | 0.24 | 1.28 | 2.86 | 0.002694 | 0.045235 |
| ENSMUSG00000035064 | *Eef2k* | 0.24 | 1.28 | 5.22 | 3.41E-05 | 0.002018 |
| ENSMUSG00000018931 | *Natd1* | 0.24 | 1.28 | 3.49 | 0.000179 | 0.007118 |
| ENSMUSG00000096472 | *Cdkn2d* | 0.25 | 1.28 | 2.94 | 0.00213 | 0.038175 |
| ENSMUSG00000021591 | *Glrx* | 0.25 | 1.28 | 3.89 | 0.000268 | 0.009252 |
| ENSMUSG00000019828 | *Grm1* | 0.25 | 1.28 | 5.52 | 3.50E-05 | 0.002055 |
| ENSMUSG00000036894 | *Rap2b* | 0.25 | 1.28 | 3.95 | 0.0003 | 0.009986 |
| ENSMUSG00000026956 | *Uap1l1* | 0.25 | 1.28 | 3.02 | 0.000832 | 0.020659 |
| ENSMUSG00000008384 | *Sertad1* | 0.25 | 1.28 | 3.02 | 0.001156 | 0.026200 |
| ENSMUSG00000007589 | *Tinf2* | 0.25 | 1.29 | 3.17 | 5.21E-05 | 0.002799 |
| ENSMUSG00000029228 | *Lnx1* | 0.26 | 1.29 | 3.63 | 1.18E-05 | 0.000892 |
| ENSMUSG00000026495 | *Efcab2* | 0.26 | 1.29 | 6.71 | 0.001813 | 0.034978 |
| ENSMUSG00000031767 | *Nudt7* | 0.26 | 1.29 | 5.45 | 8.32E-05 | 0.004063 |
| ENSMUSG00000037885 | *Stk35* | 0.26 | 1.30 | 4.85 | 6.23E-07 | 0.000096 |
| ENSMUSG00000031561 | *Tenm3* | 0.26 | 1.30 | 2.88 | 0.00047 | 0.013709 |
| ENSMUSG00000021255 | *Esrrb* | 0.26 | 1.30 | 4.63 | 0.001997 | 0.036746 |
| ENSMUSG00000046329 | *Slc25a23* | 0.26 | 1.30 | 5.23 | 4.48E-07 | 0.000073 |
| ENSMUSG00000031490 | *Eif4ebp1* | 0.27 | 1.30 | 5.44 | 0.000231 | 0.008475 |
| ENSMUSG00000021240 | *Abcd4* | 0.27 | 1.30 | 2.91 | 0.002311 | 0.040716 |
| ENSMUSG00000029001 | *Fbxo44* | 0.27 | 1.31 | 3.54 | 2.71E-05 | 0.001723 |
| ENSMUSG00000037447 | *Arid5a* | 0.27 | 1.31 | 5.21 | 3.51E-05 | 0.002055 |
| ENSMUSG00000090330 | *9130221H12Rik* | 0.27 | 1.31 | 2.21 | 0.002 | 0.036751 |
| ENSMUSG00000078919 | *Dpm1* | 0.27 | 1.31 | 2.89 | 0.000374 | 0.011570 |
| ENSMUSG00000022338 | *Eny2* | 0.27 | 1.31 | 4.25 | 5.07E-06 | 0.000481 |
| ENSMUSG00000038370 | *Pcp4l1* | 0.27 | 1.31 | 6.50 | 0.001171 | 0.026410 |
| ENSMUSG00000029287 | *Tgfbr3* | 0.27 | 1.31 | 6.11 | 7.92E-05 | 0.003922 |
| ENSMUSG00000021779 | *Thrb* | 0.27 | 1.31 | 4.79 | 0.001881 | 0.035613 |
| ENSMUSG00000038622 | *Med30* | 0.27 | 1.31 | 2.78 | 0.000523 | 0.014604 |
| ENSMUSG00000040213 | *Ccbl2* | 0.27 | 1.31 | 4.24 | 0.000251 | 0.009059 |
| ENSMUSG00000045867 | *Cradd* | 0.27 | 1.31 | 2.71 | 0.000176 | 0.007049 |
| ENSMUSG00000038156 | *Spon1* | 0.27 | 1.31 | 5.09 | 0.001909 | 0.035675 |
| ENSMUSG00000056427 | *Slit3* | 0.27 | 1.31 | 4.45 | 0.001811 | 0.034977 |
| ENSMUSG00000032666 | *1700025G04Rik* | 0.27 | 1.31 | 6.07 | 5.31E-08 | 0.000011 |
| ENSMUSG00000042429 | *Adora1* | 0.27 | 1.32 | 3.44 | 0.000482 | 0.013930 |
| ENSMUSG00000068874 | *Selenbp1* | 0.28 | 1.32 | 5.57 | 1.71E-05 | 0.001193 |
| ENSMUSG00000027244 | *Atg13* | 0.28 | 1.32 | 5.88 | 1.95E-06 | 0.000234 |
| ENSMUSG00000051341 | *Zfp52* | 0.28 | 1.32 | 3.91 | 0.000174 | 0.007015 |
| ENSMUSG00000035828 | *Pim3* | 0.28 | 1.32 | 5.77 | 0.000111 | 0.005076 |
| ENSMUSG00000047216 | *Cdh19* | 0.28 | 1.32 | 3.12 | 0.001106 | 0.025291 |
| ENSMUSG00000055041 | *Commd5* | 0.28 | 1.32 | 2.50 | 0.001221 | 0.027300 |
| ENSMUSG00000022197 | *Pdzd2* | 0.28 | 1.32 | 7.10 | 0.000559 | 0.015285 |
| ENSMUSG00000022665 | *Ccdc80* | 0.28 | 1.32 | 6.40 | 0.001222 | 0.027300 |
| ENSMUSG00000027227 | *Sord* | 0.28 | 1.32 | 6.65 | 0.000558 | 0.015285 |
| ENSMUSG00000059436 | *Max* | 0.28 | 1.32 | 4.96 | 0.000137 | 0.005855 |
| ENSMUSG00000038065 | *Mturn* | 0.29 | 1.33 | 4.07 | 0.002114 | 0.038027 |
| ENSMUSG00000058729 | *Lin9* | 0.29 | 1.33 | 2.23 | 0.000917 | 0.022162 |
| ENSMUSG00000005220 | *Corin* | 0.29 | 1.34 | 7.84 | 0.000225 | 0.008356 |
| ENSMUSG00000035296 | *Sgcg* | 0.29 | 1.34 | 6.83 | 1.09E-15 | 0.000000 |
| ENSMUSG00000027245 | *Hypk* | 0.29 | 1.34 | 2.49 | 0.001476 | 0.030513 |
| ENSMUSG00000024066 | *Xdh* | 0.29 | 1.34 | 6.82 | 0.00019 | 0.007430 |
| ENSMUSG00000052684 | *Jun* | 0.29 | 1.34 | 6.25 | 3.68E-06 | 0.000379 |
| ENSMUSG00000003526 | *Prodh* | 0.29 | 1.34 | 3.91 | 2.45E-05 | 0.001602 |
| ENSMUSG00000085793 | *Lin52* | 0.30 | 1.34 | 2.94 | 0.0001 | 0.004700 |
| ENSMUSG00000054619 | *Mettl7a1* | 0.30 | 1.34 | 4.68 | 1.08E-05 | 0.000848 |
| ENSMUSG00000037605 | *Adgrl3* | 0.30 | 1.35 | 2.01 | 0.002549 | 0.043371 |
| ENSMUSG00000100826 | *Snhg14* | 0.30 | 1.35 | 3.25 | 0.000962 | 0.022749 |
| ENSMUSG00000036944 | *Tmem71* | 0.30 | 1.35 | 3.73 | 3.61E-05 | 0.002095 |
| ENSMUSG00000044068 | *Zrsr1* | 0.30 | 1.35 | 3.67 | 0.001745 | 0.034179 |
| ENSMUSG00000031748 | *Gnao1* | 0.30 | 1.35 | 4.09 | 0.002756 | 0.045675 |
| ENSMUSG00000019124 | *Scrn1* | 0.30 | 1.35 | 3.62 | 0.000281 | 0.009529 |
| ENSMUSG00000001156 | *Mxd1* | 0.31 | 1.36 | 3.40 | 0.001876 | 0.035613 |
| ENSMUSG00000051169 | *Rpusd3* | 0.31 | 1.36 | 1.81 | 0.003032 | 0.048873 |
| ENSMUSG00000047205 | *Dusp18* | 0.31 | 1.36 | 5.52 | 0.00042 | 0.012647 |
| ENSMUSG00000037138 | *Aff3* | 0.31 | 1.36 | 2.77 | 1.33E-05 | 0.000973 |
| ENSMUSG00000085322 | *Gm14261* | 0.31 | 1.37 | 1.80 | 0.001835 | 0.035063 |
| ENSMUSG00000050410 | *Tcf19* | 0.31 | 1.37 | 1.73 | 0.002286 | 0.040417 |
| ENSMUSG00000056091 | *St3gal5* | 0.31 | 1.37 | 5.49 | 0.00228 | 0.040368 |
| ENSMUSG00000032625 | *Thsd7a* | 0.31 | 1.37 | 4.58 | 1.53E-07 | 0.000029 |
| ENSMUSG00000029311 | *Hsd17b11* | 0.31 | 1.37 | 4.32 | 4.88E-05 | 0.002635 |
| ENSMUSG00000026574 | *Dpt* | 0.31 | 1.37 | 5.96 | 2.49E-05 | 0.001607 |
| ENSMUSG00000031770 | *Herpud1* | 0.32 | 1.37 | 6.71 | 0.000712 | 0.018406 |
| ENSMUSG00000007877 | *Tcap* | 0.32 | 1.37 | 9.44 | 0.000749 | 0.019101 |
| ENSMUSG00000034235 | *Usp54* | 0.32 | 1.38 | 5.33 | 2.57E-06 | 0.000290 |
| ENSMUSG00000027346 | *Gpcpd1* | 0.32 | 1.38 | 7.99 | 0.000365 | 0.011395 |
| ENSMUSG00000042717 | *Ppp1r3a* | 0.32 | 1.38 | 8.42 | 7.66E-07 | 0.000110 |
| ENSMUSG00000078866 | *Gm14420* | 0.32 | 1.38 | 5.09 | 8.34E-07 | 0.000118 |
| ENSMUSG00000037375 | *Hhat* | 0.32 | 1.38 | 2.09 | 0.000383 | 0.011774 |
| ENSMUSG00000038193 | *Hand2* | 0.32 | 1.38 | 4.69 | 0.000878 | 0.021545 |
| ENSMUSG00000049871 | *Nlrc3* | 0.32 | 1.38 | 2.54 | 0.001957 | 0.036284 |
| ENSMUSG00000028057 | *Rit1* | 0.32 | 1.38 | 5.08 | 0.000885 | 0.021690 |
| ENSMUSG00000021902 | *Phf7* | 0.33 | 1.38 | 2.19 | 0.000204 | 0.007751 |
| ENSMUSG00000026824 | *Kcnj3* | 0.33 | 1.39 | 6.22 | 0.000109 | 0.005002 |
| ENSMUSG00000040850 | *Psme4* | 0.33 | 1.39 | 8.83 | 3.57E-09 | 0.000001 |
| ENSMUSG00000100457 | *D830032E09Rik* | 0.33 | 1.39 | 3.33 | 0.002758 | 0.045675 |
| ENSMUSG00000037784 | *Dzip1l* | 0.33 | 1.39 | 2.83 | 1.09E-05 | 0.000856 |
| ENSMUSG00000024778 | *Fas* | 0.33 | 1.39 | 3.53 | 0.002119 | 0.038070 |
| ENSMUSG00000043635 | *Adamts3* | 0.33 | 1.39 | 2.64 | 0.00127 | 0.027947 |
| ENSMUSG00000041540 | *Sox5* | 0.33 | 1.40 | 2.25 | 8.94E-05 | 0.004338 |
| ENSMUSG00000048756 | *Foxo3* | 0.33 | 1.40 | 6.91 | 3.19E-06 | 0.000342 |
| ENSMUSG00000035184 | *Fam124a* | 0.33 | 1.40 | 3.15 | 4.60E-06 | 0.000443 |
| ENSMUSG00000038059 | *Smim3* | 0.34 | 1.40 | 3.16 | 7.54E-06 | 0.000624 |
| ENSMUSG00000002835 | *Chaf1a* | 0.34 | 1.40 | 2.20 | 0.001161 | 0.026229 |
| ENSMUSG00000058396 | *Gpr182* | 0.34 | 1.41 | 2.36 | 0.002803 | 0.046051 |
| ENSMUSG00000049537 | *Tecrl* | 0.34 | 1.41 | 6.30 | 3.79E-06 | 0.000384 |
| ENSMUSG00000042029 | *Ncapg2* | 0.34 | 1.41 | 3.23 | 9.08E-05 | 0.004374 |
| ENSMUSG00000041476 | *Smpx* | 0.34 | 1.41 | 7.22 | 0.000209 | 0.007867 |
| ENSMUSG00000015850 | *Adamtsl4* | 0.34 | 1.41 | 5.35 | 2.37E-14 | 0.000000 |
| ENSMUSG00000025511 | *Tspan4* | 0.35 | 1.41 | 4.46 | 9.26E-05 | 0.004431 |
| ENSMUSG00000040648 | *Ppip5k2* | 0.35 | 1.42 | 7.57 | 0.000174 | 0.007015 |
| ENSMUSG00000039461 | *Tcta* | 0.35 | 1.42 | 5.09 | 7.92E-08 | 0.000016 |
| ENSMUSG00000024084 | *Qpct* | 0.35 | 1.42 | 2.36 | 0.000854 | 0.021094 |
| ENSMUSG00000001366 | *Fbxo9* | 0.35 | 1.42 | 4.71 | 5.11E-09 | 0.000002 |
| ENSMUSG00000039347 | *Atp6v0e2* | 0.35 | 1.42 | 2.58 | 0.000226 | 0.008371 |
| ENSMUSG00000020649 | *Rrm2* | 0.35 | 1.42 | 2.09 | 0.001288 | 0.028213 |
| ENSMUSG00000051359 | *Ncald* | 0.35 | 1.42 | 2.99 | 1.93E-05 | 0.001306 |
| ENSMUSG00000073437 | *D330041H03Rik* | 0.36 | 1.43 | 1.63 | 0.00141 | 0.029934 |
| ENSMUSG00000057133 | *Chd6* | 0.36 | 1.43 | 6.94 | 5.04E-08 | 0.000011 |
| ENSMUSG00000046345 | *Smco1* | 0.36 | 1.43 | 3.51 | 1.82E-05 | 0.001258 |
| ENSMUSG00000025269 | *Apex2* | 0.36 | 1.43 | 3.87 | 1.11E-05 | 0.000860 |
| ENSMUSG00000021876 | *Rnase4* | 0.36 | 1.43 | 5.35 | 0.000302 | 0.010042 |
| ENSMUSG00000028832 | *Stmn1* | 0.36 | 1.43 | 1.92 | 0.001286 | 0.028213 |
| ENSMUSG00000002265 | *Peg3* | 0.36 | 1.43 | 4.13 | 1.54E-10 | 0.000000 |
| ENSMUSG00000025950 | *Idh1* | 0.36 | 1.43 | 5.33 | 2.38E-14 | 0.000000 |
| ENSMUSG00000022357 | *Klhl38* | 0.36 | 1.43 | 5.00 | 0.000159 | 0.006642 |
| ENSMUSG00000001930 | *Vwf* | 0.36 | 1.44 | 6.35 | 0.002762 | 0.045675 |
| ENSMUSG00000022894 | *Adamts5* | 0.36 | 1.44 | 5.76 | 1.76E-08 | 0.000005 |
| ENSMUSG00000030306 | *Tmtc1* | 0.36 | 1.44 | 7.97 | 3.09E-06 | 0.000334 |
| ENSMUSG00000047473 | *Zfp30* | 0.36 | 1.44 | 2.88 | 3.07E-05 | 0.001899 |
| ENSMUSG00000022754 | *Tmem45a* | 0.37 | 1.44 | 2.60 | 0.002478 | 0.042707 |
| ENSMUSG00000028289 | *Epha7* | 0.37 | 1.45 | 2.86 | 1.88E-07 | 0.000035 |
| ENSMUSG00000064288 | *Hist1h4k* | 0.37 | 1.45 | 1.57 | 0.00162 | 0.032687 |
| ENSMUSG00000032122 | *Slc37a2* | 0.37 | 1.45 | 2.91 | 0.000342 | 0.010809 |
| ENSMUSG00000054641 | *Mmrn1* | 0.37 | 1.45 | 3.68 | 3.88E-11 | 0.000000 |
| ENSMUSG00000050288 | *Fzd2* | 0.37 | 1.45 | 1.88 | 0.000165 | 0.006813 |
| ENSMUSG00000029402 | *Snrnp35* | 0.37 | 1.45 | 1.32 | 0.001835 | 0.035063 |
| ENSMUSG00000053199 | *Arhgap20* | 0.37 | 1.45 | 5.70 | 5.03E-08 | 0.000011 |
| ENSMUSG00000038175 | *Mylip* | 0.38 | 1.46 | 3.93 | 1.73E-05 | 0.001203 |
| ENSMUSG00000027306 | *Nusap1* | 0.38 | 1.46 | 1.62 | 0.001125 | 0.025700 |
| ENSMUSG00000108994 | *Gm31663* | 0.38 | 1.46 | 2.62 | 0.000341 | 0.010809 |
| ENSMUSG00000024127 | *Prepl* | 0.38 | 1.46 | 6.49 | 2.50E-06 | 0.000283 |
| ENSMUSG00000029452 | *Tmem116* | 0.38 | 1.46 | 2.29 | 3.42E-05 | 0.002018 |
| ENSMUSG00000020990 | *Cdkl1* | 0.38 | 1.47 | 3.54 | 0.000855 | 0.021094 |
| ENSMUSG00000031877 | *Ces2g* | 0.39 | 1.47 | 2.04 | 0.002541 | 0.043331 |
| ENSMUSG00000040929 | *Rfx3* | 0.39 | 1.47 | 2.90 | 1.03E-05 | 0.000824 |
| ENSMUSG00000105827 | *Hist2h2bb* | 0.39 | 1.48 | 1.22 | 0.001523 | 0.031032 |
| ENSMUSG00000022548 | *Apod* | 0.39 | 1.48 | 4.48 | 0.000122 | 0.005370 |
| ENSMUSG00000049092 | *Gpr137c* | 0.39 | 1.48 | 1.72 | 0.00016 | 0.006690 |
| ENSMUSG00000073471 | *Rsph3a* | 0.39 | 1.48 | 2.29 | 3.93E-06 | 0.000393 |
| ENSMUSG00000047298 | *Kcnv2* | 0.39 | 1.48 | 3.45 | 0.001894 | 0.035613 |
| ENSMUSG00000046312 | *AI464131* | 0.40 | 1.49 | 3.30 | 9.29E-06 | 0.000756 |
| ENSMUSG00000023032 | *Slc4a8* | 0.40 | 1.49 | 2.25 | 0.002913 | 0.047421 |
| ENSMUSG00000095217 | *Hist1h2bn* | 0.40 | 1.49 | 1.61 | 0.000205 | 0.007773 |
| ENSMUSG00000036768 | *Kif15* | 0.40 | 1.49 | 1.47 | 0.002497 | 0.042791 |
| ENSMUSG00000042851 | *Zc3h6* | 0.40 | 1.49 | 3.14 | 0.002498 | 0.042791 |
| ENSMUSG00000031497 | *Tnfsf13b* | 0.40 | 1.49 | 1.83 | 5.83E-05 | 0.003093 |
| ENSMUSG00000039958 | *Mettl20* | 0.40 | 1.49 | 3.74 | 2.30E-06 | 0.000265 |
| ENSMUSG00000029722 | *Agfg2* | 0.40 | 1.49 | 3.61 | 5.66E-06 | 0.000517 |
| ENSMUSG00000030218 | *Mgp* | 0.40 | 1.49 | 6.28 | 1.10E-08 | 0.000004 |
| ENSMUSG00000074264 | *Amy1* | 0.40 | 1.50 | 3.07 | 7.72E-05 | 0.003874 |
| ENSMUSG00000085148 | *Mir22hg* | 0.41 | 1.50 | 4.55 | 1.39E-13 | 0.000000 |
| ENSMUSG00000104026 | *Gm37212* | 0.41 | 1.50 | 1.22 | 0.001351 | 0.028915 |
| ENSMUSG00000037336 | *Mfsd2b* | 0.41 | 1.50 | 1.10 | 0.001531 | 0.031116 |
| ENSMUSG00000020185 | *E2f7* | 0.41 | 1.51 | 2.08 | 0.00093 | 0.022258 |
| ENSMUSG00000037990 | *Sh3rf3* | 0.42 | 1.52 | 1.38 | 0.000336 | 0.010745 |
| ENSMUSG00000024253 | *Dync2li1* | 0.42 | 1.52 | 1.39 | 0.00031 | 0.010132 |
| ENSMUSG00000015957 | *Wnt11* | 0.42 | 1.53 | 1.92 | 0.002991 | 0.048321 |
| ENSMUSG00000031380 | *Figf* | 0.42 | 1.53 | 2.44 | 0.001399 | 0.029827 |
| ENSMUSG00000036086 | *Zranb3* | 0.42 | 1.53 | 2.49 | 8.51E-06 | 0.000697 |
| ENSMUSG00000019789 | *Hey2* | 0.42 | 1.53 | 2.54 | 3.18E-08 | 0.000008 |
| ENSMUSG00000086451 | *4933431K23Rik* | 0.43 | 1.53 | 2.31 | 1.71E-06 | 0.000207 |
| ENSMUSG00000064493 | *Snora28* | 0.43 | 1.53 | 0.90 | 0.002056 | 0.037440 |
| ENSMUSG00000024056 | *Ndc80* | 0.43 | 1.53 | 0.91 | 0.001896 | 0.035613 |
| ENSMUSG00000022425 | *Enpp2* | 0.43 | 1.54 | 3.48 | 1.47E-06 | 0.000185 |
| ENSMUSG00000022853 | *Ehhadh* | 0.43 | 1.54 | 3.34 | 0.000464 | 0.013634 |
| ENSMUSG00000020684 | *Rasl10b* | 0.43 | 1.54 | 6.20 | 0.000102 | 0.004725 |
| ENSMUSG00000008035 | *Mid1ip1* | 0.44 | 1.55 | 6.28 | 1.23E-05 | 0.000922 |
| ENSMUSG00000024471 | *Myot* | 0.44 | 1.55 | 5.30 | 5.55E-06 | 0.000513 |
| ENSMUSG00000026950 | *Neb* | 0.44 | 1.55 | 4.25 | 1.40E-07 | 0.000027 |
| ENSMUSG00000042453 | *Reln* | 0.44 | 1.55 | 3.37 | 2.41E-07 | 0.000042 |
| ENSMUSG00000041417 | *Pik3r1* | 0.44 | 1.56 | 7.72 | 1.64E-23 | 0.000000 |
| ENSMUSG00000022758 | *P2rx6* | 0.45 | 1.56 | 1.35 | 0.00072 | 0.018488 |
| ENSMUSG00000029436 | *Mmp17* | 0.45 | 1.56 | 0.94 | 0.001401 | 0.029827 |
| ENSMUSG00000025089 | *Gfra1* | 0.45 | 1.57 | 3.03 | 0.001265 | 0.027921 |
| ENSMUSG00000028702 | *Rad54l* | 0.45 | 1.57 | 0.96 | 0.000929 | 0.022258 |
| ENSMUSG00000020847 | *Rph3al* | 0.45 | 1.57 | 2.11 | 2.76E-06 | 0.000301 |
| ENSMUSG00000005699 | *Pard6a* | 0.45 | 1.57 | 1.44 | 0.001453 | 0.030284 |
| ENSMUSG00000025815 | *Dhtkd1* | 0.45 | 1.58 | 2.65 | 1.04E-07 | 0.000020 |
| ENSMUSG00000039910 | *Cited2* | 0.46 | 1.58 | 4.73 | 1.67E-05 | 0.001177 |
| ENSMUSG00000019732 | *Calr3* | 0.46 | 1.58 | 3.10 | 5.82E-05 | 0.003093 |
| ENSMUSG00000026473 | *Glul* | 0.46 | 1.58 | 8.51 | 8.53E-19 | 0.000000 |
| ENSMUSG00000008540 | *Mgst1* | 0.46 | 1.59 | 5.06 | 6.91E-10 | 0.000000 |
| ENSMUSG00000040170 | *Fmo2* | 0.47 | 1.60 | 5.09 | 0.000119 | 0.005289 |
| ENSMUSG00000046318 | *Ccbe1* | 0.47 | 1.60 | 3.40 | 2.20E-06 | 0.000258 |
| ENSMUSG00000090084 | *Srpx* | 0.47 | 1.60 | 2.76 | 0.001994 | 0.036733 |
| ENSMUSG00000022309 | *Angpt1* | 0.47 | 1.60 | 5.42 | 0.000475 | 0.013802 |
| ENSMUSG00000046598 | *Bdh1* | 0.47 | 1.60 | 6.71 | 8.20E-05 | 0.004031 |
| ENSMUSG00000032010 | *Usp2* | 0.47 | 1.61 | 6.49 | 2.24E-05 | 0.001490 |
| ENSMUSG00000030088 | *Aldh1l1* | 0.47 | 1.61 | 1.56 | 0.001851 | 0.035333 |
| ENSMUSG00000003545 | *Fosb* | 0.48 | 1.61 | 2.70 | 0.001009 | 0.023712 |
| ENSMUSG00000001739 | *Cldn15* | 0.48 | 1.62 | 2.36 | 4.51E-08 | 0.000010 |
| ENSMUSG00000024222 | *Fkbp5* | 0.48 | 1.62 | 4.96 | 0.002471 | 0.042641 |
| ENSMUSG00000020258 | *Glyctk* | 0.49 | 1.63 | 1.18 | 9.94E-05 | 0.004679 |
| ENSMUSG00000032411 | *Tfdp2* | 0.49 | 1.63 | 6.71 | 1.50E-17 | 0.000000 |
| ENSMUSG00000059974 | *Ntm* | 0.49 | 1.64 | 0.48 | 0.002419 | 0.041990 |
| ENSMUSG00000046179 | *E2f8* | 0.49 | 1.64 | 1.32 | 0.001039 | 0.024181 |
| ENSMUSG00000057719 | *Sh3rf2* | 0.50 | 1.64 | 4.47 | 3.40E-07 | 0.000057 |
| ENSMUSG00000031963 | *Bmper* | 0.50 | 1.65 | 2.38 | 0.000661 | 0.017437 |
| ENSMUSG00000043953 | *Ccrl2* | 0.50 | 1.65 | 3.39 | 2.90E-05 | 0.001827 |
| ENSMUSG00000005470 | *Asf1b* | 0.50 | 1.66 | 0.81 | 0.000387 | 0.011863 |
| ENSMUSG00000045064 | *Zc2hc1c* | 0.51 | 1.66 | 1.57 | 1.48E-05 | 0.001057 |
| ENSMUSG00000094655 | *Gm25360* | 0.52 | 1.67 | 3.92 | 0.001751 | 0.034179 |
| ENSMUSG00000020623 | *Map2k6* | 0.52 | 1.68 | 2.24 | 3.41E-07 | 0.000057 |
| ENSMUSG00000074521 | *Gm14327* | 0.52 | 1.68 | 2.58 | 1.63E-08 | 0.000005 |
| ENSMUSG00000036745 | *Ttll7* | 0.52 | 1.69 | 3.68 | 9.96E-09 | 0.000003 |
| ENSMUSG00000026204 | *Ptprn* | 0.53 | 1.69 | 2.20 | 0.000588 | 0.015833 |
| ENSMUSG00000002068 | *Ccne1* | 0.53 | 1.70 | 0.51 | 0.001914 | 0.035675 |
| ENSMUSG00000039683 | *Sdk1* | 0.53 | 1.70 | 1.41 | 5.94E-06 | 0.000522 |
| ENSMUSG00000023829 | *Slc22a1* | 0.53 | 1.70 | 0.70 | 0.002358 | 0.041240 |
| ENSMUSG00000025170 | *Rab40b* | 0.53 | 1.71 | 1.54 | 0.000158 | 0.006619 |
| ENSMUSG00000070424 | *Art5* | 0.54 | 1.71 | 3.25 | 0.000164 | 0.006802 |
| ENSMUSG00000022856 | *Tmem41a* | 0.54 | 1.72 | 2.75 | 1.12E-05 | 0.000860 |
| ENSMUSG00000081822 | *Gm15626* | 0.54 | 1.72 | 2.37 | 0.000533 | 0.014703 |
| ENSMUSG00000003541 | *Ier3* | 0.55 | 1.73 | 4.61 | 3.25E-08 | 0.000008 |
| ENSMUSG00000037818 | *Abhd18* | 0.55 | 1.73 | 5.72 | 1.41E-06 | 0.000179 |
| ENSMUSG00000029915 | *Clec5a* | 0.55 | 1.73 | 1.87 | 0.000816 | 0.020491 |
| ENSMUSG00000057880 | *Abat* | 0.55 | 1.74 | 4.32 | 5.60E-14 | 0.000000 |
| ENSMUSG00000047604 | *Frat2* | 0.55 | 1.74 | 0.49 | 0.001047 | 0.024326 |
| ENSMUSG00000042510 | *AA986860* | 0.56 | 1.74 | 0.28 | 0.002129 | 0.038175 |
| ENSMUSG00000062329 | *Cytl1* | 0.56 | 1.75 | 1.80 | 0.000972 | 0.022933 |
| ENSMUSG00000060548 | *Tnfrsf19* | 0.56 | 1.75 | 1.70 | 0.00046 | 0.013590 |
| ENSMUSG00000048070 | *Pirt* | 0.56 | 1.75 | 4.26 | 3.99E-06 | 0.000397 |
| ENSMUSG00000049539 | *Hist1h1a* | 0.56 | 1.75 | 1.89 | 0.001234 | 0.027492 |
| ENSMUSG00000040612 | *Ildr2* | 0.57 | 1.76 | 2.26 | 0.000182 | 0.007172 |
| ENSMUSG00000035365 | *Parpbp* | 0.57 | 1.77 | 0.29 | 0.001448 | 0.030245 |
| ENSMUSG00000037661 | *Gpr160* | 0.57 | 1.77 | 1.35 | 4.85E-06 | 0.000462 |
| ENSMUSG00000030711 | *Sult1a1* | 0.58 | 1.79 | 4.08 | 0.000136 | 0.005855 |
| ENSMUSG00000031765 | *Mt1* | 0.58 | 1.79 | 6.48 | 0.000765 | 0.019366 |
| ENSMUSG00000004328 | *Hif3a* | 0.59 | 1.80 | 1.44 | 0.001328 | 0.028798 |
| ENSMUSG00000056494 | *Cngb3* | 0.59 | 1.81 | 2.04 | 0.000535 | 0.014745 |
| ENSMUSG00000040537 | *Adam22* | 0.59 | 1.81 | 4.82 | 2.34E-13 | 0.000000 |
| ENSMUSG00000110298 | *RP23-385G14.1* | 0.60 | 1.83 | 0.83 | 4.68E-05 | 0.002552 |
| ENSMUSG00000025271 | *Pfkfb1* | 0.60 | 1.83 | 3.43 | 0.000112 | 0.005100 |
| ENSMUSG00000031756 | *Cenpn* | 0.61 | 1.84 | 0.41 | 0.000258 | 0.009156 |
| ENSMUSG00000097644 | *Gm26862* | 0.61 | 1.84 | 1.40 | 7.61E-07 | 0.000110 |
| ENSMUSG00000029279 | *Brdt* | 0.61 | 1.85 | -0.01 | 0.003049 | 0.049028 |
| ENSMUSG00000048442 | *Smim5* | 0.62 | 1.85 | 3.22 | 2.56E-08 | 0.000007 |
| ENSMUSG00000024076 | *Vit* | 0.62 | 1.87 | 1.38 | 2.24E-06 | 0.000260 |
| ENSMUSG00000075324 | *Fign* | 0.62 | 1.87 | 4.41 | 3.29E-05 | 0.002008 |
| ENSMUSG00000106262 | *Gm43375* | 0.63 | 1.88 | 0.17 | 0.000566 | 0.015351 |
| ENSMUSG00000033207 | *Mamdc2* | 0.63 | 1.89 | 1.12 | 9.73E-06 | 0.000783 |
| ENSMUSG00000039457 | *Ppl* | 0.64 | 1.89 | 3.17 | 0.0011 | 0.025229 |
| ENSMUSG00000028996 | *Rbp7* | 0.64 | 1.90 | 3.16 | 7.84E-08 | 0.000016 |
| ENSMUSG00000044835 | *Ankrd45* | 0.64 | 1.90 | 1.34 | 0.000519 | 0.014544 |
| ENSMUSG00000070570 | *Slc17a7* | 0.64 | 1.90 | 3.73 | 2.86E-09 | 0.000001 |
| ENSMUSG00000087236 | *Gm1305* | 0.64 | 1.90 | 0.33 | 0.00196 | 0.036307 |
| ENSMUSG00000021047 | *Nova1* | 0.65 | 1.92 | 1.85 | 9.53E-09 | 0.000003 |
| ENSMUSG00000041592 | *Sdk2* | 0.68 | 1.96 | 1.40 | 5.82E-06 | 0.000518 |
| ENSMUSG00000085162 | *Gm12295* | 0.68 | 1.98 | 2.79 | 7.91E-10 | 0.000000 |
| ENSMUSG00000030972 | *Acsm5* | 0.69 | 1.99 | 2.18 | 0.000724 | 0.018554 |
| ENSMUSG00000037139 | *Myom3* | 0.69 | 1.99 | 1.43 | 1.11E-09 | 0.000001 |
| ENSMUSG00000034258 | *Mfsd7c* | 0.70 | 2.01 | 1.86 | 0.000232 | 0.008497 |
| ENSMUSG00000055866 | *Per2* | 0.70 | 2.01 | 4.33 | 0.000621 | 0.016495 |
| ENSMUSG00000086645 | *Gm15743* | 0.71 | 2.03 | 0.93 | 1.98E-07 | 0.000036 |
| ENSMUSG00000056073 | *Grik2* | 0.71 | 2.03 | 0.32 | 3.40E-05 | 0.002018 |
| ENSMUSG00000053166 | *Cdh22* | 0.72 | 2.05 | 1.35 | 0.001203 | 0.027002 |
| ENSMUSG00000104164 | *Gm38248* | 0.72 | 2.06 | 0.58 | 3.24E-06 | 0.000343 |
| ENSMUSG00000055254 | *Ntrk2* | 0.73 | 2.08 | -0.05 | 0.00022 | 0.008221 |
| ENSMUSG00000015090 | *Ptgds* | 0.75 | 2.12 | 7.20 | 0.00019 | 0.007430 |
| ENSMUSG00000028036 | *Ptgfr* | 0.75 | 2.12 | 2.09 | 7.86E-05 | 0.003905 |
| ENSMUSG00000034472 | *Rasd2* | 0.76 | 2.15 | 1.64 | 4.05E-05 | 0.002296 |
| ENSMUSG00000030889 | *Vwa3a* | 0.77 | 2.16 | 2.46 | 1.06E-06 | 0.000140 |
| ENSMUSG00000011171 | *Vipr2* | 0.78 | 2.18 | 0.29 | 0.001155 | 0.026200 |
| ENSMUSG00000041272 | *Tox* | 0.79 | 2.19 | 0.39 | 0.000297 | 0.009920 |
| ENSMUSG00000025407 | *Gli1* | 0.79 | 2.20 | 0.26 | 0.002341 | 0.041049 |
| ENSMUSG00000055560 | *Zfp459* | 0.79 | 2.21 | 0.15 | 1.44E-05 | 0.001037 |
| ENSMUSG00000032064 | *Dixdc1* | 0.80 | 2.24 | 3.57 | 3.52E-05 | 0.002056 |
| ENSMUSG00000066361 | *Serpina3c* | 0.82 | 2.27 | -0.29 | 0.001501 | 0.030806 |
| ENSMUSG00000040205 | *Cuzd1* | 0.83 | 2.28 | 0.79 | 0.00013 | 0.005653 |
| ENSMUSG00000045725 | *Prr15* | 0.83 | 2.29 | 0.41 | 3.47E-06 | 0.000365 |
| ENSMUSG00000037196 | *Pacrg* | 0.84 | 2.31 | -0.39 | 0.000257 | 0.009140 |
| ENSMUSG00000037143 | *Cfap61* | 0.84 | 2.32 | 1.59 | 6.56E-05 | 0.003403 |
| ENSMUSG00000038486 | *Sv2a* | 0.84 | 2.32 | 1.87 | 4.42E-08 | 0.000010 |
| ENSMUSG00000039252 | *Lgi2* | 0.85 | 2.33 | 2.95 | 4.69E-05 | 0.002552 |
| ENSMUSG00000008845 | *Cd163* | 0.85 | 2.34 | 2.86 | 2.30E-08 | 0.000007 |
| ENSMUSG00000009185 | *Ccl8* | 0.87 | 2.39 | 2.34 | 3.73E-05 | 0.002143 |
| ENSMUSG00000090799 | *Klhl33* | 0.88 | 2.40 | 2.41 | 7.40E-06 | 0.000616 |
| ENSMUSG00000025900 | *Rp1* | 0.88 | 2.41 | 1.50 | 8.36E-11 | 0.000000 |
| ENSMUSG00000064343 | *mt-Tq* | 0.88 | 2.42 | 3.07 | 1.92E-08 | 0.000006 |
| ENSMUSG00000058297 | *Spock2* | 0.88 | 2.42 | 3.25 | 6.69E-11 | 0.000000 |
| ENSMUSG00000022868 | *Ahsg* | 0.89 | 2.44 | 1.81 | 0.002087 | 0.037865 |
| ENSMUSG00000097805 | *Gm17473* | 0.91 | 2.47 | 0.69 | 1.69E-06 | 0.000207 |
| ENSMUSG00000006235 | *Epor* | 0.92 | 2.51 | -0.24 | 0.000115 | 0.005163 |
| ENSMUSG00000060275 | *Nrg2* | 0.93 | 2.53 | 0.81 | 5.66E-06 | 0.000517 |
| ENSMUSG00000005089 | *Slc1a2* | 0.93 | 2.53 | 0.26 | 2.73E-06 | 0.000301 |
| ENSMUSG00000025776 | *Crispld1* | 0.94 | 2.57 | 0.97 | 9.28E-12 | 0.000000 |
| ENSMUSG00000074207 | *Adh1* | 0.96 | 2.61 | 2.17 | 3.39E-05 | 0.002018 |
| ENSMUSG00000033022 | *Cdo1* | 1.00 | 2.71 | 0.77 | 8.69E-11 | 0.000000 |
| ENSMUSG00000006014 | *Prg4* | 1.03 | 2.79 | 1.71 | 6.87E-05 | 0.003548 |
| ENSMUSG00000103642 | *Gm37769* | 1.06 | 2.88 | -0.58 | 0.000162 | 0.006729 |
| ENSMUSG00000078307 | *AI593442* | 1.12 | 3.08 | 4.35 | 4.06E-10 | 0.000000 |
| ENSMUSG00000041616 | *Nppa* | 1.13 | 3.10 | 8.02 | 5.86E-06 | 0.000518 |
| ENSMUSG00000064347 | *mt-Ta* | 1.13 | 3.11 | 0.92 | 0.000204 | 0.007751 |
| ENSMUSG00000021091 | *Serpina3n* | 1.15 | 3.17 | 5.32 | 0.000263 | 0.009208 |
| ENSMUSG00000056596 | *Trnp1* | 1.20 | 3.32 | 0.71 | 3.75E-06 | 0.000383 |
| ENSMUSG00000030317 | *Timp4* | 1.22 | 3.39 | 4.09 | 0.001304 | 0.028463 |
| ENSMUSG00000046694 | *Fam46b* | 1.29 | 3.62 | 0.57 | 1.90E-05 | 0.001294 |
| ENSMUSG00000056054 | *S100a8* | 1.34 | 3.81 | 1.56 | 0.001391 | 0.029742 |
| ENSMUSG00000056071 | *S100a9* | 1.42 | 4.13 | 1.95 | 0.001763 | 0.034279 |
| ENSMUSG00000027524 | *Edn3* | 1.53 | 4.60 | 1.73 | 1.25E-08 | 0.000004 |
| ENSMUSG00000044349 | *Snhg11* | 1.53 | 4.61 | 0.63 | 7.11E-05 | 0.003630 |
| ENSMUSG00000029304 | *Spp1* | 1.62 | 5.03 | 1.49 | 3.47E-08 | 0.000008 |
| ENSMUSG00000026442 | *Nfasc* | 1.73 | 5.64 | 0.04 | 0.000261 | 0.009195 |
| ENSMUSG00000040809 | *Chil3* | 1.74 | 5.68 | 0.41 | 0.000316 | 0.010263 |
| ENSMUSG00000031722 | *Hp* | 2.15 | 8.55 | 1.94 | 1.10E-07 | 0.000021 |
| ENSMUSG00000025479 | *Cyp2e1* | 2.19 | 8.97 | 1.22 | 0.002095 | 0.037867 |
| ENSMUSG00000024990 | *Rbp4* | 2.33 | 10.23 | -0.41 | 0.001019 | 0.023907 |
| ENSMUSG00000030895 | *Hpx* | 2.83 | 16.88 | 0.85 | 0.002908 | 0.047396 |
| ENSMUSG00000040026 | *Saa3* | 2.91 | 18.36 | 0.82 | 0.000309 | 0.010132 |
| ENSMUSG00000030483 | *Cyp2b10* | 2.96 | 19.34 | 0.08 | 1.30E-14 | 0.000000 |
| ENSMUSG00000033831 | *Fgb* | 5.95 | 383.82 | 0.68 | 0.002014 | 0.036970 |
| Legend: FDR, false discovery rate |  |  |  |  |  |  |

| **Table S4.** Pathways based on the Reactome database that are up- and down-regulated in young DOCA mice compared to age-matched sham mice. | | | | | |
| --- | --- | --- | --- | --- | --- |
| **Pathway name** | **Number of genes in pathway** | **ES** | **NES** | ***P*-value** | **FDR** |
| AXON GUIDANCE | 208 | -0.19 | -3.08 | <0.001 | <0.001 |
| INTEGRIN CELL SURFACE INTERACTIONS | 73 | -0.28 | -2.81 | <0.001 | 0.0013 |
| GPCR DOWNSTREAM SIGNALING | 230 | -0.16 | -2.71 | <0.001 | 0.0034 |
| DEVELOPMENTAL BIOLOGY | 312 | -0.13 | -2.63 | <0.001 | 0.0044 |
| SIGNALING BY GPCR | 298 | -0.13 | -2.57 | <0.001 | 0.0055 |
| N GLYCAN TRIMMING IN THE ER AND CALNEXIN CALRETICULIN CYCLE | 13 | -0.57 | -2.54 | <0.001 | 0.0052 |
| SEMA3A PAK DEPENDENT AXON REPULSION | 14 | -0.56 | -2.52 | <0.001 | 0.0059 |
| N GLYCAN ANTENNAE ELONGATION | 11 | -0.62 | -2.47 | <0.001 | 0.0091 |
| SIGNALLING BY NGF | 193 | -0.15 | -2.34 | <0.001 | 0.0223 |
| SIGNALING BY NOTCH4 | 11 | -0.57 | -2.30 | <0.001 | 0.0252 |
| UNFOLDED PROTEIN RESPONSE | 71 | -0.23 | -2.29 | <0.001 | 0.0248 |
| ASPARAGINE N LINKED GLYCOSYLATION | 75 | -0.23 | -2.29 | 0.0019 | 0.0227 |
| SIGNALING BY NOTCH3 | 11 | -0.57 | -2.29 | <0.001 | 0.0220 |
| POST TRANSLATIONAL PROTEIN MODIFICATION | 135 | -0.18 | -2.28 | 0.0020 | 0.0204 |
| SEMAPHORIN INTERACTIONS | 62 | -0.25 | -2.27 | <0.001 | 0.0204 |
| SEMA3A PLEXIN REPULSION SIGNALING BY INHIBITING INTEGRIN ADHESION | 13 | -0.52 | -2.26 | <0.001 | 0.0210 |
| N GLYCAN ANTENNAE ELONGATION IN THE MEDIAL TRANS GOLGI | 15 | -0.48 | -2.22 | <0.001 | 0.0253 |
| CALNEXIN CALRETICULIN CYCLE | 11 | -0.55 | -2.21 | <0.001 | 0.0252 |
| SIGNALING BY ROBO RECEPTOR | 27 | -0.36 | -2.21 | <0.001 | 0.0246 |
| DIABETES PATHWAYS | 105 | -0.19 | -2.19 | 0.0019 | 0.0258 |
| INTERFERON SIGNALING | 109 | -0.18 | -2.18 | 0.0058 | 0.0258 |
| NCAM SIGNALING FOR NEURITE OUT GROWTH | 53 | -0.25 | -2.18 | 0.0020 | 0.0255 |
| NGF SIGNALLING VIA TRKA FROM THE PLASMA MEMBRANE | 127 | -0.16 | -2.18 | <0.001 | 0.0250 |
| PLATELET ACTIVATION SIGNALING AND AGGREGATION | 164 | -0.15 | -2.17 | <0.001 | 0.0249 |
| L1CAM INTERACTIONS | 69 | -0.22 | -2.17 | 0.0038 | 0.0242 |
| ANTIVIRAL MECHANISM BY IFN STIMULATED GENES | 56 | -0.24 | -2.17 | <0.001 | 0.0241 |
| SIGNALING BY NOTCH2 | 10 | -0.56 | -2.12 | <0.001 | 0.0313 |
| CYTOKINE SIGNALING IN IMMUNE SYSTEM | 205 | -0.12 | -2.08 | 0.0060 | 0.0410 |
| G ALPHA S SIGNALLING EVENTS | 55 | -0.24 | -2.07 | 0.0081 | 0.0411 |
| CLEAVAGE OF GROWING TRANSCRIPT IN THE TERMINATION REGION | 38 | -0.27 | -2.07 | 0.0079 | 0.0411 |
| IMMUNE SYSTEM | 700 | -0.07 | -2.06 | <0.001 | 0.0421 |
| ACTIVATION OF CHAPERONE GENES BY XBP1S | 42 | -0.26 | -2.04 | 0.0040 | 0.0443 |
| TRANSPORT OF MATURE TRANSCRIPT TO CYTOPLASM | 48 | -0.25 | -2.04 | 0.0021 | 0.0436 |
| MRNA 3 END PROCESSING | 30 | -0.31 | -2.04 | 0.0039 | 0.0424 |
| HEMOSTASIS | 344 | -0.10 | -2.03 | 0.0040 | 0.0424 |
| SIGNALLING TO RAS | 25 | -0.33 | -2.01 | 0.0041 | 0.0477 |
| GASTRIN CREB SIGNALLING PATHWAY VIA PKC AND MAPK | 109 | -0.17 | -2.01 | 0.0117 | 0.0464 |
| FORMATION OF TUBULIN FOLDING INTERMEDIATES BY CCT TRIC | 18 | -0.39 | -2.00 | 0.0019 | 0.0455 |
| EFFECTS OF PIP2 HYDROLYSIS | 19 | -0.38 | -2.00 | 0.0061 | 0.0458 |
| INTEGRATION OF ENERGY METABOLISM | 88 | -0.18 | -2.00 | <0.001 | 0.0456 |
| SIGNALLING TO ERKS | 33 | -0.30 | -1.98 | 0.0083 | 0.0489 |
| CHROMOSOME MAINTENANCE | 75 | 0.19 | 1.91 | 0.0063 | 0.0401 |
| ABCA TRANSPORTERS IN LIPID HOMEOSTASIS | 12 | 0.47 | 1.95 | 0.0081 | 0.0321 |
| CDT1 ASSOCIATION WITH THE CDC6 ORC ORIGIN COMPLEX | 49 | 0.23 | 1.95 | 0.0142 | 0.0312 |
| PHASE1 FUNCTIONALIZATION OF COMPOUNDS | 32 | 0.29 | 1.97 | 0.0041 | 0.0288 |
| REGULATION OF PYRUVATE DEHYDROGENASE PDH COMPLEX | 12 | 0.48 | 2.00 | 0.0077 | 0.0252 |
| G2 M CHECKPOINTS | 39 | 0.28 | 2.01 | 0.0081 | 0.0237 |
| RNA POL I PROMOTER OPENING | 16 | 0.42 | 2.01 | 0.0039 | 0.0238 |
| ACTIVATION OF THE MRNA UPON BINDING OF THE CAP BINDING COMPLEX AND EIFS AND SUBSEQUENT BINDING TO 43S | 42 | 0.26 | 2.02 | 0.0040 | 0.0231 |
| ACTIVATION OF THE PRE REPLICATIVE COMPLEX | 28 | 0.32 | 2.03 | 0.0020 | 0.0230 |
| PYRUVATE METABOLISM AND CITRIC ACID TCA CYCLE | 39 | 0.29 | 2.08 | 0.0039 | 0.0169 |
| MEIOTIC RECOMBINATION | 33 | 0.31 | 2.08 | 0.0021 | 0.0166 |
| GLUTATHIONE CONJUGATION | 14 | 0.47 | 2.09 | <0.001 | 0.0162 |
| SCFSKP2 MEDIATED DEGRADATION OF P27 P21 | 48 | 0.25 | 2.10 | 0.0019 | 0.0157 |
| ASSEMBLY OF THE PRE REPLICATIVE COMPLEX | 58 | 0.23 | 2.12 | 0.0020 | 0.0151 |
| FORMATION OF THE TERNARY COMPLEX AND SUBSEQUENTLY THE 43S COMPLEX | 35 | 0.30 | 2.13 | <0.001 | 0.0148 |
| FORMATION OF FIBRIN CLOT CLOTTING CASCADE | 16 | 0.44 | 2.14 | <0.001 | 0.0135 |
| PHASE II CONJUGATION | 31 | 0.32 | 2.16 | 0.0079 | 0.0125 |
| TRANSLATION | 105 | 0.18 | 2.16 | 0.0020 | 0.0126 |
| AMYLOIDS | 26 | 0.36 | 2.16 | <0.001 | 0.0127 |
| CYCLIN E ASSOCIATED EVENTS DURING G1 S TRANSITION | 57 | 0.24 | 2.16 | <0.001 | 0.0131 |
| CELL CYCLE CHECKPOINTS | 105 | 0.18 | 2.18 | <0.001 | 0.0125 |
| ORC1 REMOVAL FROM CHROMATIN | 59 | 0.25 | 2.18 | <0.001 | 0.0125 |
| IRON UPTAKE AND TRANSPORT | 29 | 0.33 | 2.19 | <0.001 | 0.0127 |
| NONSENSE MEDIATED DECAY ENHANCED BY THE EXON JUNCTION COMPLEX | 70 | 0.22 | 2.19 | 0.0020 | 0.0130 |
| UNWINDING OF DNA | 11 | 0.53 | 2.22 | 0.0019 | 0.0115 |
| FORMATION OF ATP BY CHEMIOSMOTIC COUPLING | 13 | 0.50 | 2.24 | <0.001 | 0.0104 |
| TELOMERE MAINTENANCE | 43 | 0.29 | 2.28 | 0.0021 | 0.0080 |
| SRP DEPENDENT COTRANSLATIONAL PROTEIN TARGETING TO MEMBRANE | 73 | 0.23 | 2.31 | <0.001 | 0.0065 |
| M G1 TRANSITION | 72 | 0.24 | 2.38 | <0.001 | 0.0038 |
| DNA STRAND ELONGATION | 30 | 0.38 | 2.43 | <0.001 | 0.0026 |
| MITOTIC G1 G1 S PHASES | 117 | 0.19 | 2.46 | <0.001 | 0.0026 |
| MITOTIC M M G1 PHASES | 151 | 0.18 | 2.47 | <0.001 | 0.0023 |
| METABOLISM OF AMINO ACIDS AND DERIVATIVES | 136 | 0.19 | 2.51 | <0.001 | 0.0020 |
| G1 S TRANSITION | 96 | 0.22 | 2.53 | <0.001 | 0.0019 |
| BRANCHED CHAIN AMINO ACID CATABOLISM | 17 | 0.52 | 2.53 | 0.0020 | 0.0021 |
| CELL CYCLE | 334 | 0.12 | 2.55 | <0.001 | 0.0019 |
| CELL CYCLE MITOTIC | 283 | 0.14 | 2.58 | <0.001 | 0.0016 |
| 3 UTR MEDIATED TRANSLATIONAL REGULATION | 67 | 0.28 | 2.69 | <0.001 | 0.0005 |
| BIOLOGICAL OXIDATIONS | 63 | 0.30 | 2.71 | <0.001 | 0.0006 |
| INFLUENZA VIRAL RNA TRANSCRIPTION AND REPLICATION | 66 | 0.30 | 2.83 | <0.001 | 0.0002 |
| PEPTIDE CHAIN ELONGATION | 51 | 0.34 | 2.85 | <0.001 | 0.0001 |
| SYNTHESIS OF DNA | 84 | 0.27 | 2.88 | <0.001 | 0.0001 |
| DNA REPLICATION | 170 | 0.20 | 2.98 | <0.001 | <0.001 |
| S PHASE | 100 | 0.26 | 3.04 | <0.001 | <0.001 |
| RESPIRATORY ELECTRON TRANSPORT | 59 | 0.44 | 4.05 | <0.001 | <0.001 |
| RESPIRATORY ELECTRON TRANSPORT ATP SYNTHESIS BY CHEMIOSMOTIC COUPLING AND HEAT PRODUCTION BY UNCOUPLING PROTEINS | 75 | 0.40 | 4.05 | <0.001 | <0.001 |
| TCA CYCLE AND RESPIRATORY ELECTRON TRANSPORT | 110 | 0.37 | 4.51 | <0.001 | <0.001 |
| Legend: ES, enrichment score; NES, normalized enrichment score; FDR, false discovery rate. | |  |  |  |  |

| **Table S5.** Cardiac genes differentially expressed between aged sham vs DOCA mice (FDR<0.05). | | | | | | |
| --- | --- | --- | --- | --- | --- | --- |
| **Transcript access** | **Gene symbol** | **logFC** | **Fold change** | **logCPM** | ***P*-Value** | **FDR** |
| ENSMUSG00000076258 | *Gm23935* | -2.61 | -13.66 | 12.08 | 0.00000007 | 0.001 |
| ENSMUSG00000047976 | *Kcna1* | -1.07 | -2.92 | 1.31 | 0.00001744 | 0.018 |
| ENSMUSG00000064337 | *mt-Rnr1* | -0.56 | -1.75 | 15.22 | 0.00005319 | 0.031 |
| ENSMUSG00000026971 | *Itgb6* | -0.41 | -1.51 | 5.26 | 0.00002112 | 0.018 |
| ENSMUSG00000000751 | *Rpa1* | -0.28 | -1.33 | 5.21 | 0.00004429 | 0.029 |
| ENSMUSG00000004319 | *Clcn3* | -0.28 | -1.33 | 6.10 | 0.00001504 | 0.017 |
| ENSMUSG00000031618 | *Nr3c2* | -0.28 | -1.33 | 5.12 | 0.00010025 | 0.045 |
| ENSMUSG00000051495 | *Irf2bp2* | -0.28 | -1.32 | 6.82 | 0.00000323 | 0.007 |
| ENSMUSG00000058756 | *Thra* | -0.27 | -1.31 | 6.03 | 0.00007267 | 0.037 |
| ENSMUSG00000020160 | *Meis1* | -0.27 | -1.31 | 4.98 | 0.00012042 | 0.047 |
| ENSMUSG00000025958 | *Creb1* | -0.25 | -1.29 | 5.58 | 0.00001994 | 0.018 |
| ENSMUSG00000041598 | *Cdc42ep4* | 0.31 | 1.36 | 3.94 | 0.00004644 | 0.029 |
| ENSMUSG00000024066 | *Xdh* | 0.32 | 1.37 | 6.98 | 0.00010293 | 0.045 |
| ENSMUSG00000003418 | *St8sia6* | 0.35 | 1.42 | 3.64 | 0.00002865 | 0.020 |
| ENSMUSG00000037966 | *Ninj1* | 0.37 | 1.45 | 4.67 | 0.00004945 | 0.030 |
| ENSMUSG00000056091 | *St3gal5* | 0.40 | 1.49 | 5.48 | 0.00011817 | 0.047 |
| ENSMUSG00000031342 | *Gpm6b* | 0.43 | 1.54 | 5.22 | 0.00006269 | 0.033 |
| ENSMUSG00000109812 | *RP23-235I2.1* | 0.45 | 1.57 | 2.96 | 0.00002508 | 0.019 |
| ENSMUSG00000052684 | *Jun* | 0.48 | 1.62 | 6.36 | 0.00000234 | 0.006 |
| ENSMUSG00000032925 | *Itgbl1* | 0.49 | 1.63 | 3.69 | 0.00000101 | 0.004 |
| ENSMUSG00000064080 | *Fbln2* | 0.51 | 1.66 | 7.01 | 0.00000422 | 0.008 |
| ENSMUSG00000051669 | *AU021092* | 0.51 | 1.67 | 2.67 | 0.00008512 | 0.041 |
| ENSMUSG00000022440 | *C1qtnf6* | 0.53 | 1.70 | 2.77 | 0.00000821 | 0.013 |
| ENSMUSG00000102153 | *Gm37474* | 0.54 | 1.72 | 3.25 | 0.00009260 | 0.043 |
| ENSMUSG00000039994 | *Timeless* | 0.59 | 1.80 | 3.62 | 0.00000215 | 0.006 |
| ENSMUSG00000031253 | *Srpx2* | 0.61 | 1.85 | 2.92 | 0.00001141 | 0.015 |
| ENSMUSG00000031765 | *Mt1* | 0.66 | 1.93 | 6.01 | 0.00008319 | 0.041 |
| ENSMUSG00000078202 | *Nrarp* | 0.69 | 1.99 | 4.14 | 0.00000068 | 0.003 |
| ENSMUSG00000103046 | *Gm37309* | 0.79 | 2.20 | 1.02 | 0.00006235 | 0.033 |
| ENSMUSG00000068196 | *Col8a1* | 0.91 | 2.48 | 5.32 | 0.00000945 | 0.014 |
| ENSMUSG00000001228 | *Uhrf1* | 1.00 | 2.72 | 1.64 | 0.00011426 | 0.047 |
| ENSMUSG00000048647 | *Exd1* | 1.07 | 2.91 | 0.34 | 0.00001485 | 0.017 |
| ENSMUSG00000043157 | *Arl11* | 1.08 | 2.95 | 0.26 | 0.00002408 | 0.019 |
| ENSMUSG00000042489 | *Clspn* | 1.18 | 3.24 | 0.61 | 0.00011780 | 0.047 |
| ENSMUSG00000038943 | *Prc1* | 1.18 | 3.27 | 1.96 | 0.00000054 | 0.003 |
| ENSMUSG00000034311 | *Kif4* | 1.19 | 3.30 | 0.82 | 0.00002604 | 0.019 |
| ENSMUSG00000028718 | *Stil* | 1.39 | 4.01 | -0.03 | 0.00002138 | 0.018 |
| Legend: FDR, false discovery rate |  |  |  |  |  |  |

| Table S6. Pathways based on the database that are up- and down-regulated in aged DOCA mice compared to age-matched sham mice. | | | | | |
| --- | --- | --- | --- | --- | --- |
| **Pathway name** | **Number of genes in pathway** | **ES** | **NES** | ***P*-value** | **FDR** |
| FATTY ACID TRIACYLGLYCEROL AND KETONE BODY METABOLISM | 154 | -0.24 | -3.44 | <0.001 | <0.001 |
| PYRUVATE METABOLISM AND CITRIC ACID TCA CYCLE | 39 | -0.43 | -3.14 | <0.001 | <0.001 |
| CITRIC ACID CYCLE TCA CYCLE | 19 | -0.61 | -3.13 | <0.001 | <0.001 |
| TRANSPORT OF MATURE TRANSCRIPT TO CYTOPLASM | 48 | -0.33 | -2.74 | <0.001 | 0.0007 |
| PROCESSING OF CAPPED INTRON CONTAINING PRE MRNA | 125 | -0.21 | -2.72 | <0.001 | 0.0007 |
| TRIGLYCERIDE BIOSYNTHESIS | 33 | -0.40 | -2.72 | <0.001 | 0.0006 |
| GLUCOSE METABOLISM | 55 | -0.29 | -2.62 | <0.001 | 0.0011 |
| MRNA 3 END PROCESSING | 30 | -0.41 | -2.62 | <0.001 | 0.0011 |
| MRNA SPLICING | 99 | -0.22 | -2.51 | 0.0020 | 0.0032 |
| CIRCADIAN REPRESSION OF EXPRESSION BY REV ERBA | 20 | -0.45 | -2.45 | <0.001 | 0.0050 |
| MRNA PROCESSING | 142 | -0.18 | -2.42 | <0.001 | 0.0065 |
| TRNA AMINOACYLATION | 42 | -0.30 | -2.34 | <0.001 | 0.0104 |
| RORA ACTIVATES CIRCADIAN EXPRESSION | 22 | -0.42 | -2.33 | <0.001 | 0.0106 |
| CLEAVAGE OF GROWING TRANSCRIPT IN THE TERMINATION REGION | 38 | -0.32 | -2.30 | <0.001 | 0.0116 |
| MITOCHONDRIAL FATTY ACID BETA OXIDATION | 14 | -0.50 | -2.28 | 0.0020 | 0.0126 |
| VOLTAGE GATED POTASSIUM CHANNELS | 22 | -0.41 | -2.26 | 0.0019 | 0.0148 |
| GLUCONEOGENESIS | 26 | -0.36 | -2.22 | <0.001 | 0.0184 |
| FATTY ACYL COA BIOSYNTHESIS | 14 | -0.50 | -2.21 | 0.0020 | 0.0185 |
| N GLYCAN TRIMMING IN THE ER AND CALNEXIN CALRETICULIN CYCLE | 13 | -0.50 | -2.14 | 0.0058 | 0.0270 |
| CALNEXIN CALRETICULIN CYCLE | 11 | -0.52 | -2.12 | 0.0061 | 0.0295 |
| BRANCHED CHAIN AMINO ACID CATABOLISM | 17 | -0.42 | -2.12 | 0.0020 | 0.0289 |
| METABOLISM OF LIPIDS AND LIPOPROTEINS | 371 | -0.10 | -2.11 | <0.001 | 0.0293 |
| AMINO ACID SYNTHESIS AND INTERCONVERSION TRANSAMINATION | 15 | -0.44 | -2.09 | <0.001 | 0.0316 |
| NEP NS2 INTERACTS WITH THE CELLULAR EXPORT MACHINERY | 24 | -0.36 | -2.06 | 0.0020 | 0.0363 |
| PPARA ACTIVATES GENE EXPRESSION | 95 | -0.19 | -2.06 | <0.001 | 0.0365 |
| CELL CELL JUNCTION ORGANIZATION | 27 | -0.33 | -2.04 | 0.0060 | 0.0370 |
| CYTOSOLIC TRNA AMINOACYLATION | 24 | -0.35 | -2.03 | <0.001 | 0.0383 |
| CHOLESTEROL BIOSYNTHESIS | 19 | -0.39 | -2.02 | 0.0040 | 0.0388 |
| TRANSPORT OF MATURE MRNA DERIVED FROM AN INTRONLESS TRANSCRIPT | 29 | -0.32 | -2.01 | <0.001 | 0.0414 |
| TRANSCRIPTIONAL REGULATION OF WHITE ADIPOCYTE DIFFERENTIATION | 66 | -0.21 | -2.01 | 0.0085 | 0.0404 |
| REGULATION OF MITOTIC CELL CYCLE | 72 | 0.19 | 1.88 | 0.0043 | 0.0488 |
| NFKB ACTIVATION THROUGH FADD RIP1 PATHWAY MEDIATED BY CASPASE 8 AND10 | 11 | 0.48 | 1.89 | 0.0098 | 0.0475 |
| G BETA GAMMA SIGNALLING THROUGH PI3KGAMMA | 19 | 0.37 | 1.91 | 0.0061 | 0.0414 |
| RNA POL I TRANSCRIPTION | 39 | 0.26 | 1.92 | 0.0122 | 0.0408 |
| MITOTIC PROMETAPHASE | 75 | 0.19 | 1.93 | 0.0039 | 0.0397 |
| VIF MEDIATED DEGRADATION OF APOBEC3G | 44 | 0.25 | 1.93 | 0.0119 | 0.0399 |
| G2 M CHECKPOINTS | 39 | 0.26 | 1.93 | 0.0078 | 0.0397 |
| NFKB ACTIVATION THROUGH FADD RIP1 PATHWAY MEDIATED BY CASPASE 8 AND10 | 11 | 0.48 | 1.93 | 0.0061 | 0.0399 |
| ACTIVATION OF IRF3 IRF7 MEDIATED BY TBK1 IKK EPSILON | 12 | 0.47 | 1.93 | 0.0077 | 0.0405 |
| INTEGRIN ALPHAIIB BETA3 SIGNALING | 27 | 0.31 | 1.94 | 0.0042 | 0.0385 |
| NCAM SIGNALING FOR NEURITE OUT GROWTH | 53 | 0.23 | 1.95 | 0.0060 | 0.0384 |
| RESPONSE TO ELEVATED PLATELET CYTOSOLIC CA2 | 68 | 0.21 | 1.98 | 0.0062 | 0.0326 |
| APOPTOSIS | 122 | 0.15 | 1.98 | 0.0019 | 0.0326 |
| MEIOTIC RECOMBINATION | 33 | 0.30 | 2.05 | 0.0039 | 0.0220 |
| ER PHAGOSOME PATHWAY | 48 | 0.25 | 2.05 | 0.0078 | 0.0222 |
| PLATELET AGGREGATION PLUG FORMATION | 28 | 0.32 | 2.06 | 0.0039 | 0.0220 |
| SCFSKP2 MEDIATED DEGRADATION OF P27 P21 | 48 | 0.25 | 2.06 | 0.0059 | 0.0217 |
| P130CAS LINKAGE TO MAPK SIGNALING FOR INTEGRINS | 15 | 0.44 | 2.07 | 0.0019 | 0.0216 |
| G1 S SPECIFIC TRANSCRIPTION | 13 | 0.48 | 2.08 | <0.001 | 0.0211 |
| G0 AND EARLY G1 | 20 | 0.39 | 2.10 | 0.0039 | 0.0184 |
| E2F MEDIATED REGULATION OF DNA REPLICATION | 28 | 0.33 | 2.12 | 0.0081 | 0.0165 |
| CROSS PRESENTATION OF SOLUBLE EXOGENOUS ANTIGENS ENDOSOMES | 42 | 0.28 | 2.12 | 0.0020 | 0.0166 |
| KINESINS | 21 | 0.39 | 2.14 | 0.0082 | 0.0147 |
| P53 INDEPENDENT G1 S DNA DAMAGE CHECKPOINT | 44 | 0.27 | 2.14 | 0.0020 | 0.0143 |
| METABOLISM OF MRNA | 168 | 0.15 | 2.18 | 0.0020 | 0.0115 |
| HEMOSTASIS | 344 | 0.10 | 2.18 | <0.001 | 0.0117 |
| CDK MEDIATED PHOSPHORYLATION AND REMOVAL OF CDC6 | 42 | 0.28 | 2.18 | 0.0040 | 0.0117 |
| CYCLIN A B1 ASSOCIATED EVENTS DURING G2 M TRANSITION | 13 | 0.50 | 2.18 | 0.0039 | 0.0117 |
| AMYLOIDS | 26 | 0.36 | 2.20 | 0.0041 | 0.0105 |
| AUTODEGRADATION OF THE E3 UBIQUITIN LIGASE COP1 | 43 | 0.29 | 2.20 | <0.001 | 0.0108 |
| EXTRACELLULAR MATRIX ORGANIZATION | 58 | 0.25 | 2.21 | 0.0020 | 0.0108 |
| NCAM1 INTERACTIONS | 30 | 0.34 | 2.22 | <0.001 | 0.0104 |
| INFLUENZA LIFE CYCLE | 97 | 0.19 | 2.24 | <0.001 | 0.0094 |
| TRAFFICKING AND PROCESSING OF ENDOSOMAL TLR | 10 | 0.58 | 2.24 | <0.001 | 0.0094 |
| INFLAMMASOMES | 15 | 0.47 | 2.24 | 0.0021 | 0.0096 |
| TOLL RECEPTOR CASCADES | 105 | 0.19 | 2.26 | <0.001 | 0.0086 |
| INNATE IMMUNE SYSTEM | 182 | 0.14 | 2.28 | 0.0039 | 0.0073 |
| CDT1 ASSOCIATION WITH THE CDC6 ORC ORIGIN COMPLEX | 49 | 0.28 | 2.33 | <0.001 | 0.0050 |
| ANTIGEN PROCESSING CROSS PRESENTATION | 60 | 0.26 | 2.37 | <0.001 | 0.0042 |
| CELL CYCLE CHECKPOINTS | 105 | 0.20 | 2.42 | <0.001 | 0.0031 |
| INTERFERON ALPHA BETA SIGNALING | 37 | 0.33 | 2.44 | <0.001 | 0.0027 |
| GLYCOSAMINOGLYCAN METABOLISM | 80 | 0.23 | 2.44 | <0.001 | 0.0028 |
| DEPOSITION OF NEW CENPA CONTAINING NUCLEOSOMES AT THE CENTROMERE | 27 | 0.39 | 2.46 | <0.001 | 0.0028 |
| P53 DEPENDENT G1 DNA DAMAGE RESPONSE | 49 | 0.30 | 2.50 | <0.001 | 0.0022 |
| LIPOPROTEIN METABOLISM | 19 | 0.48 | 2.52 | <0.001 | 0.0017 |
| ACTIVATION OF THE PRE REPLICATIVE COMPLEX | 28 | 0.41 | 2.53 | <0.001 | 0.0017 |
| INTEGRIN CELL SURFACE INTERACTIONS | 73 | 0.26 | 2.55 | <0.001 | 0.0015 |
| UNWINDING OF DNA | 11 | 0.64 | 2.55 | <0.001 | 0.0015 |
| COLLAGEN FORMATION | 46 | 0.32 | 2.57 | <0.001 | 0.0013 |
| ORC1 REMOVAL FROM CHROMATIN | 59 | 0.29 | 2.58 | <0.001 | 0.0014 |
| SYNTHESIS OF DNA | 84 | 0.25 | 2.61 | <0.001 | 0.0010 |
| ACTIVATION OF THE MRNA UPON BINDING OF THE CAP BINDING COMPLEX AND EIFS AND SUBSEQUENT BINDING TO 43S | 42 | 0.35 | 2.66 | <0.001 | 0.0007 |
| S PHASE | 100 | 0.23 | 2.66 | <0.001 | 0.0007 |
| CELL CYCLE | 334 | 0.13 | 2.70 | <0.001 | 0.0006 |
| CELL CYCLE MITOTIC | 283 | 0.14 | 2.71 | <0.001 | 0.0005 |
| RNA POL I PROMOTER OPENING | 16 | 0.56 | 2.72 | <0.001 | 0.0005 |
| FORMATION OF THE TERNARY COMPLEX AND SUBSEQUENTLY THE 43S COMPLEX | 35 | 0.39 | 2.72 | <0.001 | 0.0005 |
| NONSENSE MEDIATED DECAY ENHANCED BY THE EXON JUNCTION COMPLEX | 70 | 0.27 | 2.73 | <0.001 | 0.0005 |
| ASSEMBLY OF THE PRE REPLICATIVE COMPLEX | 58 | 0.30 | 2.74 | <0.001 | 0.0005 |
| PLATELET ACTIVATION SIGNALING AND AGGREGATION | 164 | 0.19 | 2.84 | <0.001 | 0.0003 |
| DNA REPLICATION | 170 | 0.19 | 2.88 | <0.001 | 0.0001 |
| MITOTIC M M G1 PHASES | 151 | 0.21 | 3.00 | <0.001 | 0.0001 |
| SRP DEPENDENT COTRANSLATIONAL PROTEIN TARGETING TO MEMBRANE | 73 | 0.31 | 3.11 | <0.001 | <0.001 |
| TRANSLATION | 105 | 0.26 | 3.14 | <0.001 | <0.001 |
| MITOTIC G1 G1 S PHASES | 117 | 0.26 | 3.16 | <0.001 | <0.001 |
| M G1 TRANSITION | 72 | 0.32 | 3.25 | <0.001 | <0.001 |
| G1 S TRANSITION | 96 | 0.29 | 3.32 | <0.001 | <0.001 |
| INFLUENZA VIRAL RNA TRANSCRIPTION AND REPLICATION | 66 | 0.37 | 3.54 | <0.001 | <0.001 |
| 3 UTR MEDIATED TRANSLATIONAL REGULATION | 67 | 0.37 | 3.59 | <0.001 | <0.001 |
| PEPTIDE CHAIN ELONGATION | 51 | 0.45 | 3.87 | <0.001 | <0.001 |
| Legend: ES, enrichment score; NES, normalized enrichment score; FDR, false discovery rate. | |  |  |  |  |

| **Table S7.** Interaction between age and DOCA treatment (FDR<0.05). | | | | |  |  |  |  |  |  |
| --- | --- | --- | --- | --- | --- | --- | --- | --- | --- | --- |
| **Transcript access** | **Gene symbol** | **logFC** | **Fold change** | **logCPM** | ***P*-Value** | **FDR** | **Mean young sham** | **Mean young DOCA** | **Mean old sham** | **Mean old DOCA** |
| ENSMUSG00000027559 | *Car3* | -5.00 | -148.11 | 4.79 | 9.71E-05 | 0.0341 | 2.64 | 8.62 | 1404.51 | 3.75 |
| ENSMUSG00000031722 | *Hp* | -3.23 | -25.35 | 2.63 | 6.76E-06 | 0.0077 | 12.01 | 82.55 | 179.74 | 12.24 |
| ENSMUSG00000037071 | *Scd1* | -2.99 | -19.89 | 8.08 | 0.00019 | 0.0472 | 278.88 | 404.11 | 13332.65 | 272.44 |
| ENSMUSG00000030483 | *Cyp2b10* | -2.14 | -8.50 | 0.93 | 1.90E-05 | 0.0136 | 1.62 | 27.40 | 34.59 | 24.71 |
| ENSMUSG00000028033 | *Kcnq5* | -1.40 | -4.04 | 0.09 | 0.0002 | 0.0473 | 7.78 | 12.41 | 19.14 | 3.82 |
| ENSMUSG00000103642 | *Gm37769* | -1.38 | -3.98 | 0.05 | 4.94E-05 | 0.0206 | 4.30 | 10.21 | 22.56 | 6.84 |
| ENSMUSG00000039457 | *Ppl* | -1.00 | -2.71 | 3.47 | 0.000107 | 0.0341 | 90.76 | 143.12 | 220.44 | 77.70 |
| ENSMUSG00000052854 | *Nrk* | -0.96 | -2.61 | 0.71 | 0.000165 | 0.0433 | 22.78 | 26.65 | 14.85 | 4.03 |
| ENSMUSG00000045725 | *Prr15* | -0.91 | -2.48 | 0.51 | 0.00018 | 0.0454 | 11.10 | 20.89 | 19.76 | 9.29 |
| ENSMUSG00000064343 | *mt-Tq* | -0.69 | -2.00 | 3.25 | 2.59E-05 | 0.0151 | 74.86 | 150.43 | 135.87 | 93.18 |
| ENSMUSG00000028132 | *Tmem56* | -0.66 | -1.93 | 2.41 | 4.46E-05 | 0.0201 | 47.08 | 50.56 | 110.11 | 42.24 |
| ENSMUSG00000110298 | *RP23-385G14.1* | -0.64 | -1.90 | 1.35 | 0.000109 | 0.0341 | 17.33 | 26.03 | 47.35 | 25.86 |
| ENSMUSG00000026824 | *Kcnj3* | -0.57 | -1.77 | 6.52 | 2.77E-05 | 0.0154 | 891.48 | 1036.10 | 1671.38 | 785.48 |
| ENSMUSG00000037605 | *Adgrl3* | -0.55 | -1.74 | 2.01 | 6.95E-06 | 0.0077 | 47.09 | 53.75 | 56.66 | 26.68 |
| ENSMUSG00000104026 | *Gm37212* | -0.55 | -1.74 | 1.51 | 1.11E-05 | 0.0100 | 25.35 | 31.98 | 47.48 | 24.74 |
| ENSMUSG00000028289 | *Epha7* | -0.49 | -1.63 | 3.12 | 1.77E-05 | 0.0134 | 84.38 | 100.48 | 147.20 | 79.04 |
| ENSMUSG00000042453 | *Reln* | -0.48 | -1.62 | 3.73 | 4.11E-07 | 0.0013 | 115.83 | 150.38 | 226.96 | 134.97 |
| ENSMUSG00000004187 | *Kifc2* | -0.47 | -1.60 | 2.23 | 0.00014 | 0.0394 | 51.15 | 52.69 | 76.44 | 36.52 |
| ENSMUSG00000028399 | *Ptprd* | -0.44 | -1.55 | 4.64 | 6.40E-07 | 0.0015 | 263.06 | 276.23 | 424.99 | 216.68 |
| ENSMUSG00000018347 | *Zkscan6* | -0.42 | -1.52 | 2.59 | 4.84E-05 | 0.0206 | 75.59 | 77.91 | 82.69 | 42.42 |
| ENSMUSG00000021779 | *Thrb* | -0.39 | -1.47 | 4.84 | 0.000148 | 0.0394 | 339.29 | 373.36 | 409.26 | 234.57 |
| ENSMUSG00000024127 | *Prepl* | -0.37 | -1.45 | 6.64 | 0.000122 | 0.0366 | 1050.51 | 1286.15 | 1455.71 | 947.45 |
| ENSMUSG00000056476 | *Med12l* | -0.33 | -1.39 | 3.95 | 9.49E-05 | 0.0341 | 174.15 | 174.91 | 238.62 | 135.75 |
| ENSMUSG00000024548 | *Setbp1* | -0.32 | -1.38 | 4.63 | 0.000106 | 0.0341 | 275.01 | 293.32 | 372.38 | 226.46 |
| ENSMUSG00000037795 | *N4bp2* | -0.32 | -1.38 | 4.52 | 8.39E-06 | 0.0081 | 269.96 | 257.28 | 356.62 | 194.24 |
| ENSMUSG00000033308 | *Dpyd* | -0.32 | -1.38 | 3.80 | 5.22E-05 | 0.0209 | 174.76 | 177.99 | 189.28 | 110.21 |
| ENSMUSG00000001518 | *Itfg2* | -0.30 | -1.35 | 3.38 | 1.28E-05 | 0.0102 | 130.56 | 128.96 | 143.61 | 83.00 |
| ENSMUSG00000018166 | *Erbb3* | -0.27 | -1.31 | 4.14 | 4.99E-05 | 0.0206 | 213.13 | 217.28 | 241.50 | 150.98 |
| ENSMUSG00000001366 | *Fbxo9* | -0.27 | -1.31 | 4.71 | 0.000103 | 0.0341 | 308.17 | 366.54 | 319.05 | 233.49 |
| ENSMUSG00000019878 | *Hsf2* | -0.26 | -1.30 | 4.03 | 3.47E-05 | 0.0167 | 199.00 | 200.15 | 228.04 | 142.13 |
| ENSMUSG00000035967 | *Ddx26b* | -0.24 | -1.27 | 4.44 | 4.00E-06 | 0.0064 | 253.75 | 251.43 | 314.67 | 199.10 |
| ENSMUSG00000004319 | *Clcn3* | -0.21 | -1.24 | 6.09 | 3.36E-05 | 0.0167 | 929.60 | 796.26 | 941.57 | 534.94 |
| ENSMUSG00000027329 | *Spef1* | 0.53 | 1.70 | 2.65 | 1.31E-08 | 0.0002 | 95.00 | 54.51 | 63.67 | 67.77 |
| ENSMUSG00000071347 | *C1qtnf9* | 0.50 | 1.65 | 5.17 | 3.95E-07 | 0.0013 | 710.79 | 368.12 | 284.83 | 263.90 |
| ENSMUSG00000038943 | *Prc1* | 0.76 | 2.14 | 2.48 | 4.04E-07 | 0.0013 | 86.25 | 85.09 | 21.30 | 54.05 |
| ENSMUSG00000070462 | *Mesdc1* | 0.40 | 1.49 | 4.47 | 4.54E-07 | 0.0013 | 366.65 | 233.36 | 205.01 | 201.23 |
| ENSMUSG00000044317 | *Gpr4* | 0.46 | 1.58 | 3.24 | 3.41E-06 | 0.0064 | 139.27 | 88.44 | 96.40 | 102.72 |
| ENSMUSG00000074457 | *S100a16* | 0.23 | 1.26 | 4.67 | 3.79E-06 | 0.0064 | 393.13 | 279.10 | 266.31 | 232.40 |
| ENSMUSG00000078202 | *Nrarp* | 0.59 | 1.81 | 4.27 | 4.75E-06 | 0.0068 | 296.54 | 216.25 | 143.48 | 212.49 |
| ENSMUSG00000044043 | *Pcdhb14* | 0.44 | 1.55 | 2.86 | 5.48E-06 | 0.0072 | 101.35 | 66.14 | 76.99 | 82.20 |
| ENSMUSG00000011877 | *Git1* | 0.22 | 1.24 | 5.90 | 8.12E-06 | 0.0081 | 876.79 | 631.24 | 674.70 | 585.09 |
| ENSMUSG00000032220 | *Myo1e* | 0.37 | 1.44 | 4.72 | 1.19E-05 | 0.0101 | 490.75 | 286.72 | 222.90 | 192.64 |
| ENSMUSG00000001493 | *Meox1* | 0.73 | 2.08 | 4.92 | 2.08E-05 | 0.0136 | 636.38 | 325.71 | 172.66 | 217.19 |
| ENSMUSG00000033960 | *9430020K01Rik* | 0.27 | 1.31 | 7.09 | 2.08E-05 | 0.0136 | 2232.46 | 1521.00 | 1338.44 | 1178.27 |
| ENSMUSG00000020121 | *Srgap1* | 0.44 | 1.55 | 3.19 | 2.53E-05 | 0.0151 | 162.23 | 102.07 | 69.84 | 71.77 |
| ENSMUSG00000041707 | *1810011H11Rik* | 0.75 | 2.12 | 0.80 | 2.63E-05 | 0.0151 | 24.46 | 16.85 | 11.30 | 19.80 |
| ENSMUSG00000044813 | *Shb* | 0.44 | 1.55 | 4.31 | 3.09E-05 | 0.0165 | 339.65 | 179.96 | 203.35 | 176.66 |
| ENSMUSG00000023886 | *Smoc2* | 0.21 | 1.23 | 6.15 | 3.32E-05 | 0.0167 | 1064.95 | 801.36 | 744.35 | 668.00 |
| ENSMUSG00000079426 | *Arpc4* | 0.30 | 1.35 | 4.82 | 3.76E-05 | 0.0175 | 456.47 | 338.90 | 251.98 | 251.47 |
| ENSMUSG00000041633 | *Kctd12b* | 0.51 | 1.67 | 5.14 | 8.73E-05 | 0.0340 | 695.36 | 284.48 | 351.99 | 261.39 |
| ENSMUSG00000072244 | *Trim6* | 0.75 | 2.12 | 0.66 | 9.77E-05 | 0.0341 | 25.56 | 15.97 | 8.74 | 13.93 |
| ENSMUSG00000034485 | *Uaca* | 0.23 | 1.25 | 6.26 | 9.96E-05 | 0.0341 | 1193.65 | 851.37 | 795.29 | 692.26 |
| ENSMUSG00000021773 | *Comtd1* | 0.48 | 1.61 | 1.90 | 0.000108 | 0.0341 | 53.47 | 37.98 | 33.13 | 40.70 |
| ENSMUSG00000047307 | *Pcdhb13* | 0.78 | 2.19 | 0.49 | 0.000118 | 0.0361 | 18.62 | 11.50 | 9.98 | 16.44 |
| ENSMUSG00000027663 | *Zmat3* | 0.34 | 1.41 | 5.48 | 0.000128 | 0.0376 | 814.06 | 587.35 | 310.09 | 321.21 |
| ENSMUSG00000020092 | *Pald1* | 0.35 | 1.42 | 4.86 | 0.000136 | 0.0393 | 501.13 | 329.92 | 255.08 | 242.53 |
| ENSMUSG00000002603 | *Tgfb1* | 0.32 | 1.38 | 4.91 | 0.000146 | 0.0394 | 485.68 | 369.40 | 254.62 | 269.81 |
| ENSMUSG00000029171 | *Pgm1* | 0.33 | 1.39 | 3.08 | 0.000147 | 0.0394 | 142.59 | 96.44 | 73.15 | 69.65 |
| ENSMUSG00000022440 | *C1qtnf6* | 0.49 | 1.64 | 3.53 | 0.00017 | 0.0436 | 220.64 | 156.55 | 61.16 | 76.52 |
| ENSMUSG00000054435 | *Gimap4* | 0.27 | 1.32 | 4.86 | 0.000193 | 0.0472 | 546.14 | 308.52 | 271.74 | 200.24 |
| ENSMUSG00000070639 | *Lrrc8b* | 0.36 | 1.43 | 4.11 | 0.000196 | 0.0472 | 293.78 | 194.53 | 150.06 | 144.87 |
| ENSMUSG00000000489 | *Pdgfb* | 0.48 | 1.61 | 5.92 | 0.000212 | 0.0492 | 1002.35 | 629.05 | 538.60 | 582.49 |
| Legend: FDR, false discovery rate | |  |  |  |  |  |  |  |  |  |

| **Table S8.** Pathways based on the Reactome database that interact between age and DOCA treatment. | | |  |  |  |
| --- | --- | --- | --- | --- | --- |
| **Pathway name** | **Number of genes in pathway** | **ES** | **NES** | ***P*-value** | **FDR** |
| TCA CYCLE AND RESPIRATORY ELECTRON TRANSPORT | 110 | -0.35 | -4.30 | <0.001 | <0.001 |
| PYRUVATE METABOLISM AND CITRIC ACID TCA CYCLE | 39 | -0.50 | -3.69 | <0.001 | <0.001 |
| RESPIRATORY ELECTRON TRANSPORT ATP SYNTHESIS BY CHEMIOSMOTIC COUPLING AND HEAT PRODUCTION BY UNCOUPLING PROTEINS | 75 | -0.33 | -3.32 | <0.001 | <0.001 |
| CITRIC ACID CYCLE TCA CYCLE | 19 | -0.60 | -3.17 | <0.001 | <0.001 |
| RESPIRATORY ELECTRON TRANSPORT | 59 | -0.33 | -3.02 | <0.001 | <0.001 |
| PHASE1 FUNCTIONALIZATION OF COMPOUNDS | 32 | -0.41 | -2.72 | <0.001 | 0.0007 |
| MITOCHONDRIAL FATTY ACID BETA OXIDATION | 14 | -0.59 | -2.71 | <0.001 | 0.0008 |
| BRANCHED CHAIN AMINO ACID CATABOLISM | 17 | -0.53 | -2.71 | <0.001 | 0.0008 |
| FATTY ACID TRIACYLGLYCEROL AND KETONE BODY METABOLISM | 154 | -0.19 | -2.69 | <0.001 | 0.0008 |
| REGULATION OF PYRUVATE DEHYDROGENASE PDH COMPLEX | 12 | -0.59 | -2.49 | <0.001 | 0.0037 |
| NITRIC OXIDE STIMULATES GUANYLATE CYCLASE | 17 | -0.51 | -2.49 | <0.001 | 0.0033 |
| GLUCOSE METABOLISM | 55 | -0.27 | -2.35 | <0.001 | 0.0092 |
| BIOLOGICAL OXIDATIONS | 63 | -0.25 | -2.31 | 0.0019 | 0.0119 |
| CYTOCHROME P450 ARRANGED BY SUBSTRATE TYPE | 19 | -0.44 | -2.30 | 0.0019 | 0.0121 |
| CGMP EFFECTS | 11 | -0.56 | -2.26 | 0.0019 | 0.0145 |
| VOLTAGE GATED POTASSIUM CHANNELS | 22 | -0.39 | -2.21 | <0.001 | 0.0190 |
| RORA ACTIVATES CIRCADIAN EXPRESSION | 22 | -0.40 | -2.18 | 0.0019 | 0.0217 |
| METABOLISM OF LIPIDS AND LIPOPROTEINS | 371 | -0.10 | -2.17 | 0.0038 | 0.0236 |
| VITAMIN B5 PANTOTHENATE METABOLISM | 11 | -0.52 | -2.16 | 0.0061 | 0.0227 |
| CHOLESTEROL BIOSYNTHESIS | 19 | -0.41 | -2.16 | 0.0020 | 0.0226 |
| MRNA SPLICING | 99 | -0.18 | -2.11 | 0.0040 | 0.0304 |
| TIGHT JUNCTION INTERACTIONS | 11 | -0.51 | -2.09 | 0.0020 | 0.0315 |
| GLUCONEOGENESIS | 26 | -0.34 | -2.06 | 0.0043 | 0.0360 |
| PROCESSING OF CAPPED INTRON CONTAINING PRE MRNA | 125 | -0.16 | -2.05 | 0.0020 | 0.0357 |
| COMPLEMENT CASCADE | 15 | -0.43 | -2.05 | 0.0020 | 0.0352 |
| PYRUVATE METABOLISM | 17 | -0.41 | -2.03 | 0.0060 | 0.0385 |
| GASTRIN CREB SIGNALLING PATHWAY VIA PKC AND MAPK | 109 | 0.16 | 1.92 | 0.0143 | 0.0482 |
| REGULATION OF SIGNALING BY CBL | 18 | 0.37 | 1.92 | 0.0152 | 0.0487 |
| INTRINSIC PATHWAY FOR APOPTOSIS | 23 | 0.33 | 1.94 | 0.0020 | 0.0454 |
| NUCLEOTIDE BINDING DOMAIN LEUCINE RICH REPEAT CONTAINING RECEPTOR NLR SIGNALING PATHWAYS | 41 | 0.26 | 1.94 | 0.0019 | 0.0441 |
| N GLYCAN ANTENNAE ELONGATION IN THE MEDIAL TRANS GOLGI | 15 | 0.42 | 1.95 | 0.0080 | 0.0434 |
| NFKB ACTIVATION THROUGH FADD RIP1 PATHWAY MEDIATED BY CASPASE 8 AND10 | 11 | 0.49 | 1.95 | 0.0041 | 0.0433 |
| G ALPHA1213 SIGNALLING EVENTS | 62 | 0.22 | 1.97 | 0.0038 | 0.0408 |
| CHONDROITIN SULFATE DERMATAN SULFATE METABOLISM | 38 | 0.27 | 1.98 | 0.0082 | 0.0397 |
| SEMA3A PLEXIN REPULSION SIGNALING BY INHIBITING INTEGRIN ADHESION | 13 | 0.46 | 1.99 | 0.0057 | 0.0384 |
| THROMBIN SIGNALLING THROUGH PROTEINASE ACTIVATED RECEPTORS PARS | 23 | 0.35 | 1.99 | 0.0099 | 0.0381 |
| RNA POL I PROMOTER OPENING | 16 | 0.42 | 1.99 | 0.0059 | 0.0383 |
| G PROTEIN BETA GAMMA SIGNALLING | 22 | 0.36 | 2.01 | 0.0122 | 0.0367 |
| G BETA GAMMA SIGNALLING THROUGH PI3KGAMMA | 19 | 0.38 | 2.01 | 0.0043 | 0.0358 |
| NONSENSE MEDIATED DECAY ENHANCED BY THE EXON JUNCTION COMPLEX | 70 | 0.21 | 2.05 | 0.0041 | 0.0286 |
| CYTOKINE SIGNALING IN IMMUNE SYSTEM | 205 | 0.13 | 2.09 | 0.0099 | 0.0222 |
| INTERFERON SIGNALING | 109 | 0.17 | 2.10 | <0.001 | 0.0225 |
| THE NLRP3 INFLAMMASOME | 10 | 0.55 | 2.11 | 0.0021 | 0.0206 |
| INTEGRIN ALPHAIIB BETA3 SIGNALING | 27 | 0.35 | 2.12 | 0.0079 | 0.0207 |
| N GLYCAN ANTENNAE ELONGATION | 11 | 0.53 | 2.12 | <0.001 | 0.0207 |
| TRAF6 MEDIATED NFKB ACTIVATION | 17 | 0.43 | 2.13 | <0.001 | 0.0203 |
| GPVI MEDIATED ACTIVATION CASCADE | 30 | 0.34 | 2.16 | 0.0038 | 0.0171 |
| RIG I MDA5 MEDIATED INDUCTION OF IFN ALPHA BETA PATHWAYS | 51 | 0.26 | 2.17 | 0.0038 | 0.0164 |
| METABOLISM OF MRNA | 168 | 0.15 | 2.19 | <0.001 | 0.0153 |
| INTERFERON GAMMA SIGNALING | 40 | 0.29 | 2.23 | 0.0020 | 0.0113 |
| TOLL RECEPTOR CASCADES | 105 | 0.18 | 2.24 | 0.0021 | 0.0111 |
| L1CAM INTERACTIONS | 69 | 0.23 | 2.24 | 0.0021 | 0.0115 |
| PLATELET AGGREGATION PLUG FORMATION | 28 | 0.36 | 2.26 | <0.001 | 0.0102 |
| ACTIVATION OF THE MRNA UPON BINDING OF THE CAP BINDING COMPLEX AND EIFS AND SUBSEQUENT BINDING TO 43S | 42 | 0.30 | 2.28 | <0.001 | 0.0090 |
| INTERFERON ALPHA BETA SIGNALING | 37 | 0.32 | 2.30 | 0.0020 | 0.0081 |
| INFLUENZA LIFE CYCLE | 97 | 0.20 | 2.30 | <0.001 | 0.0083 |
| NCAM1 INTERACTIONS | 30 | 0.36 | 2.31 | <0.001 | 0.0080 |
| INNATE IMMUNE SYSTEM | 182 | 0.15 | 2.33 | <0.001 | 0.0077 |
| TRAFFICKING AND PROCESSING OF ENDOSOMAL TLR | 10 | 0.60 | 2.34 | <0.001 | 0.0073 |
| METABOLISM OF PROTEINS | 331 | 0.11 | 2.38 | <0.001 | 0.0058 |
| SEMA3A PAK DEPENDENT AXON REPULSION | 14 | 0.54 | 2.38 | <0.001 | 0.0061 |
| FORMATION OF THE TERNARY COMPLEX AND SUBSEQUENTLY THE 43S COMPLEX | 35 | 0.34 | 2.41 | <0.001 | 0.0050 |
| SIGNALING BY GPCR | 298 | 0.12 | 2.42 | <0.001 | 0.0048 |
| GLYCOSAMINOGLYCAN METABOLISM | 80 | 0.24 | 2.48 | <0.001 | 0.0031 |
| EXTRACELLULAR MATRIX ORGANIZATION | 58 | 0.28 | 2.48 | <0.001 | 0.0031 |
| CHONDROITIN SULFATE BIOSYNTHESIS | 14 | 0.56 | 2.49 | <0.001 | 0.0030 |
| IMMUNE SYSTEM | 700 | 0.08 | 2.50 | 0.0020 | 0.0029 |
| GPCR DOWNSTREAM SIGNALING | 230 | 0.14 | 2.51 | <0.001 | 0.0029 |
| SEMAPHORIN INTERACTIONS | 62 | 0.27 | 2.52 | <0.001 | 0.0030 |
| NCAM SIGNALING FOR NEURITE OUT GROWTH | 53 | 0.29 | 2.56 | <0.001 | 0.0027 |
| SRP DEPENDENT COTRANSLATIONAL PROTEIN TARGETING TO MEMBRANE | 73 | 0.26 | 2.56 | <0.001 | 0.0029 |
| DEVELOPMENTAL BIOLOGY | 312 | 0.13 | 2.64 | <0.001 | 0.0018 |
| INFLAMMASOMES | 15 | 0.58 | 2.73 | <0.001 | 0.0012 |
| TRANSLATION | 105 | 0.24 | 2.79 | <0.001 | 0.0006 |
| INFLUENZA VIRAL RNA TRANSCRIPTION AND REPLICATION | 66 | 0.31 | 2.80 | <0.001 | 0.0007 |
| COLLAGEN FORMATION | 46 | 0.36 | 2.87 | <0.001 | 0.0003 |
| PEPTIDE CHAIN ELONGATION | 51 | 0.36 | 3.02 | <0.001 | <0.001 |
| INTEGRIN CELL SURFACE INTERACTIONS | 73 | 0.30 | 3.09 | <0.001 | <0.001 |
| HEMOSTASIS | 344 | 0.14 | 3.11 | <0.001 | <0.001 |
| 3 UTR MEDIATED TRANSLATIONAL REGULATION | 67 | 0.34 | 3.31 | <0.001 | <0.001 |
| AXON GUIDANCE | 208 | 0.21 | 3.53 | <0.001 | <0.001 |
| PLATELET ACTIVATION SIGNALING AND AGGREGATION | 164 | 0.24 | 3.57 | <0.001 | <0.001 |
| Legend: ES, enrichment score; NES, normalized enrichment score; FDR, false discovery rate. | |  |  |  |  |
