# Supplementary material for: Age-Related Differential Structural and Transcriptomic Responses in the Hypertensive Heart
Source: Front Physiol. 2018 Jul 9;9:817. doi: 10.3389/fphys.2018.00817 (PMC6046461; doi:10.3389/fphys.2018.00817)
Supplement: Supplementary file 2 [file Table_9.PDF]

Table S9. Pathways based on the MsigDB database that interact between age and DOCA treatment.

Pathway name	Number of genes in pathway	ES	NES	*P* -value	FDR
HALLMARK_OXIDATIVE_PHOSPHORYLATION	191	-0.31	-5.00	<0.001	<0.001
HSIAO_LIVER_SPECIFIC_GENES	128	-0.37	-4.96	<0.001	<0.001
FLECHNER_BIOPSY_KIDNEY_TRANSPLANT_REJECTED_VS_OK_DN	457	-0.20	-4.84	<0.001	<0.001
GO_ORGANIC_ACID_CATABOLIC_PROCESS	150	-0.32	-4.58	<0.001	<0.001
VECCHI_GASTRIC_CANCER_EARLY_DN	235	-0.25	-4.57	<0.001	<0.001
GO_SMALL_MOLECULE_METABOLIC_PROCESS	1303	-0.11	-4.50	<0.001	<0.001
GO_MITOCHONDRIAL_PART	814	-0.14	-4.50	<0.001	<0.001
GO_OXIDATION_REDUCTION_PROCESS	670	-0.16	-4.41	<0.001	<0.001
BURTON_ADIPOGENESIS_6	174	-0.27	-4.36	<0.001	<0.001
MOOTHA_MITOCHONDRIA	405	-0.19	-4.36	<0.001	<0.001
REACTOME_TCA_CYCLE_AND_RESPIRATORY_ELECTRON_TRANSPORT	110	-0.35	-4.31	<0.001	<0.001
GO_MITOCHONDRION	1360	-0.11	-4.31	<0.001	<0.001
GO_MONOCARBOXYLIC_ACID_METABOLIC_PROCESS	347	-0.20	-4.29	<0.001	<0.001
MOOTHA_HUMAN_MITODB_6_2002	388	-0.19	-4.29	<0.001	<0.001
GSE32533_WT_VS_MIR17_OVEREXPRESS_ACT_CD4_TCELL_DN	182	-0.27	-4.23	<0.001	<0.001
GO_ORGANIC_ACID_METABOLIC_PROCESS	686	-0.14	-4.10	<0.001	<0.001
GO_FATTY_ACID_METABOLIC_PROCESS	218	-0.24	-4.06	<0.001	<0.001
LEE_BMP2_TARGETS_UP	611	-0.14	-4.03	<0.001	<0.001
GO_OXIDOREDUCTASE_ACTIVITY	508	-0.16	-4.03	<0.001	<0.001
GO_SMALL_MOLECULE_CATABOLIC_PROCESS	237	-0.23	-4.01	<0.001	<0.001
MODULE_221	28	-0.62	-3.91	<0.001	<0.001
SHETH_LIVER_CANCER_VS_TXNIP_LOSS_PAM4	182	-0.25	-3.88	<0.001	<0.001
MODULE_184	27	-0.62	-3.87	<0.001	<0.001
RODWELL_AGING_KIDNEY_NO_BLOOD_DN	119	-0.31	-3.86	<0.001	<0.001
HALLMARK_ADIPOGENESIS	192	-0.24	-3.85	<0.001	<0.001
KEGG_VALINE_LEUCINE_AND_ISOLEUCINE_DEGRADATION	42	-0.49	-3.84	<0.001	<0.001
GO_MITOCHONDRIAL_MATRIX	369	-0.17	-3.83	<0.001	<0.001
HALLMARK_FATTY_ACID_METABOLISM	134	-0.28	-3.82	<0.001	<0.001
RODWELL_AGING_KIDNEY_DN	108	-0.32	-3.80	<0.001	<0.001
HOSHIDA_LIVER_CANCER_SUBCLASS_S3	184	-0.24	-3.78	<0.001	<0.001
GO_ORGANELLE_INNER_MEMBRANE	441	-0.16	-3.74	<0.001	<0.001
GO_MONOCARBOXYLIC_ACID_CATABOLIC_PROCESS	78	-0.36	-3.74	<0.001	<0.001

GO_CELLULAR_RESPIRATION	127	-0.29	-3.71	<0.001	<0.001
REACTOME_PYRUVATE_METABOLISM_AND_CITRIC_ACID_TCA_CYCLE	39	-0.50	-3.70	<0.001	<0.001
GO_TRICARBOXYLIC_ACID_METABOLIC_PROCESS	35	-0.53	-3.69	<0.001	<0.001
KEGG_CITRATE_CYCLE_TCA_CYCLE	29	-0.56	-3.66	<0.001	<0.001
TGGAAA_NFAT_Q4_01	1465	-0.09	-3.64	<0.001	<0.001
KEGG_PEROXISOME	68	-0.37	-3.62	<0.001	<0.001
TURASHVILI_BREAST_LOBULAR_CARCINOMA_VS_LOBULAR_NORMAL_UP	64	-0.39	-3.60	<0.001	<0.001
GO_FATTY_ACID_CATABOLIC_PROCESS	63	-0.39	-3.59	<0.001	<0.001
GO_MITOCHONDRIAL_ENVELOPE	584	-0.13	-3.58	<0.001	<0.001
SMID_BREAST_CANCER_BASAL_DN	495	-0.14	-3.57	<0.001	<0.001
GO_GENERATION_OF_PRECURSOR_METABOLITES_AND_ENERGY	242	-0.20	-3.56	<0.001	<0.001
GO_CELLULAR_AMINO_ACID_CATABOLIC_PROCESS	76	-0.34	-3.54	<0.001	<0.001
GO_FATTY_ACID_BETA_OXIDATION	47	-0.43	-3.52	<0.001	<0.001
GO_COFACTOR_BINDING	215	-0.21	-3.50	<0.001	<0.001
GO_AEROBIC_RESPIRATION	46	-0.43	-3.50	<0.001	<0.001
GO_LIPID_METABOLIC_PROCESS	809	-0.11	-3.47	<0.001	<0.001
KIM_ALL_DISORDERS_CALB1_CORR_UP	462	-0.14	-3.46	<0.001	<0.001
OCT1_02	137	-0.25	-3.46	<0.001	<0.001
GO_ENERGY_DERIVATION_BY_OXIDATION_OF_ORGANIC_COMPOUNDS	187	-0.22	-3.45	<0.001	<0.001
MODULE_152	117	-0.27	-3.45	<0.001	<0.001
GSE31622_WT_VS_KLF3_KO_BCELL_UP	162	-0.23	-3.44	<0.001	<0.001
SHEN_SMARCA2_TARGETS_UP	403	-0.15	-3.42	<0.001	<0.001
GO_COFACTOR_METABOLIC_PROCESS	259	-0.19	-3.41	<0.001	<0.001
WONG_MITOCHONDRIA_GENE_MODULE	209	-0.21	-3.40	<0.001	<0.001
MORF_DEK	241	-0.19	-3.38	<0.001	<0.001
HORTON_SREBF_TARGETS	21	-0.62	-3.36	<0.001	<0.001
RUAN_RESPONSE_TO_TNF_DN	81	-0.32	-3.35	<0.001	<0.001
REACTOME_RESPIRATORY_ELECTRON_TRANSPORT_ATP_SYNTHESIS_BY_CHEMI	75	-0.33	-3.34	<0.001	<0.001
MODULE_93	138	-0.24	-3.32	<0.001	1.30E-05
KEGG_BUTANOATE_METABOLISM	26	-0.54	-3.31	<0.001	1.28E-05
GO_OXIDATIVE_PHOSPHORYLATION	75	-0.33	-3.31	<0.001	1.26E-05
KAAB_HEART_ATRIUM_VS_VENTRICLE_DN	239	-0.18	-3.30	<0.001	1.24E-05
DIAZ_CHRONIC_MEYLOGENOUS_LEUKEMIA_UP	1280	-0.08	-3.29	<0.001	2.45E-05
MORF_HAT1	163	-0.22	-3.29	<0.001	2.41E-05

CAR_HPX	22	-0.58	-3.28	<0.001	2.37E-05
GO_CELLULAR_LIPID_METABOLIC_PROCESS	671	-0.11	-3.26	<0.001	2.34E-05
GO_LIPID_OXIDATION	62	-0.35	-3.26	<0.001	2.31E-05
RUAN_RESPONSE_TO_TNF_TROGLITAZONE_DN	39	-0.44	-3.26	<0.001	3.39E-05
MORF_BUB3	263	-0.17	-3.25	<0.001	3.34E-05
GO_OXIDOREDUCTASE_ACTIVITY_ACTING_ON_THE_CH_OH_GROUP_OF_DONO	73	-0.32	-3.25	<0.001	3.30E-05
BLALOCK_ALZHEIMERS_DISEASE_DN	1024	-0.09	-3.24	<0.001	3.25E-05
SCHLOSSER_SERUM_RESPONSE_DN	615	-0.11	-3.24	<0.001	3.21E-05
KEGG_PROPANOATE_METABOLISM	29	-0.51	-3.24	<0.001	3.17E-05
LEE_LIVER_CANCER_SURVIVAL_UP	108	-0.27	-3.23	<0.001	4.15E-05
GO_MICROBODY	113	-0.26	-3.23	<0.001	4.10E-05
GO_OXIDOREDUCTASE_ACTIVITY_ACTING_ON_CH_OH_GROUP_OF_DONORS	88	-0.29	-3.22	<0.001	4.04E-05
GO_LYASE_ACTIVITY	141	-0.24	-3.20	<0.001	3.99E-05
NAKAYAMA_SOFT_TISSUE_TUMORS_PCA2_DN	65	-0.34	-3.20	<0.001	3.94E-05
NADLER_OBESITY_DN	44	-0.40	-3.19	<0.001	3.89E-05
GO_THIOESTER_METABOLIC_PROCESS	60	-0.34	-3.18	<0.001	3.85E-05
FOXO3_01	185	-0.20	-3.18	<0.001	3.80E-05
GO_COENZYME_BINDING	154	-0.22	-3.18	<0.001	3.75E-05
GO_REGULATION_OF_FATTY_ACID_METABOLIC_PROCESS	67	-0.33	-3.16	<0.001	3.71E-05
KEGG_FATTY_ACID_METABOLISM	34	-0.46	-3.16	<0.001	3.67E-05
HALLMARK_XENOBIOTIC_METABOLISM	154	-0.22	-3.16	<0.001	3.62E-05
GO_ELECTRON_TRANSPORT_CHAIN	85	-0.28	-3.15	<0.001	3.58E-05
TTANWNANTGGM_UNKNOWN	490	-0.12	-3.14	<0.001	5.32E-05
GO_DICARBOXYLIC_ACID_METABOLIC_PROCESS	73	-0.32	-3.13	<0.001	5.27E-05
GO_ENVELOPE	904	-0.09	-3.11	<0.001	6.07E-05
REACTOME_CITRIC_ACID_CYCLE_TCA_CYCLE	19	-0.60	-3.11	<0.001	6.01E-05
FOX_Q2	146	-0.23	-3.10	<0.001	6.79E-05
GO_ALPHA_AMINO_ACID_CATABOLIC_PROCESS	60	-0.34	-3.09	<0.001	6.72E-05
HALLMARK_BILE_ACID_METABOLISM	81	-0.30	-3.08	<0.001	8.31E-05
GNF2_HPX	56	-0.36	-3.08	<0.001	8.22E-05
SNACANNNYSYAGA_UNKNOWN	260	-0.17	-3.08	<0.001	8.13E-05
GO_SULFUR_COMPOUND_METABOLIC_PROCESS	260	-0.16	-3.04	<0.001	1.21E-04
AAAYWAACM_HFH4_01	1352	-0.08	-3.04	<0.001	1.20E-04
GO_ACETYL_COA_METABOLIC_PROCESS	18	-0.59	-3.04	<0.001	1.18E-04

GO_COENZYME_METABOLIC_PROCESS	204	-0.18	-3.03	<0.001	1.25E-04
MORF_CSNK2B	276	-0.16	-3.02	<0.001	1.47E-04
KEGG_PARKINSONS_DISEASE	97	-0.26	-3.02	<0.001	1.45E-04
GO_GLYOXYLATE_METABOLIC_PROCESS	23	-0.52	-3.01	<0.001	1.74E-04
GO_CELLULAR_LIPID_CATABOLIC_PROCESS	114	-0.25	-3.01	<0.001	1.73E-04
GO_MITOCHONDRIAL_PROTEIN_COMPLEX	123	-0.23	-3.01	<0.001	1.71E-04
MORF_HDAC1	240	-0.17	-3.01	<0.001	1.69E-04
MORF_SOD1	266	-0.16	-2.99	<0.001	1.75E-04
KEGG_OXIDATIVE_PHOSPHORYLATION	100	-0.25	-2.98	<0.001	1.81E-04
MODULE_294	13	-0.67	-2.98	<0.001	1.79E-04
GSE14000_TRANSLATED_RNA_VS_MRNA_4H_LPS_DC_UP	170	-0.20	-2.97	<0.001	1.92E-04
GO_INNER_MITOCHONDRIAL_MEMBRANE_PROTEIN_COMPLEX	96	-0.25	-2.95	<0.001	2.19E-04
MODULE_305	18	-0.58	-2.95	<0.001	2.17E-04
MODULE_40	61	-0.32	-2.95	<0.001	2.15E-04
GO_MITOCHONDRIAL_MEMBRANE_PART	147	-0.20	-2.93	<0.001	2.54E-04
RUAN_RESPONSE_TO_TROGLITAZONE_DN	17	-0.60	-2.93	<0.001	2.59E-04
LI_ADIPOGENESIS_BY_ACTIVATED_PPARG	17	-0.59	-2.93	<0.001	2.63E-04
REACTOME_RESPIRATORY_ELECTRON_TRANSPORT	59	-0.33	-2.93	<0.001	2.61E-04
MORF_HDAC2	260	-0.16	-2.92	<0.001	2.72E-04
GNF2_LCAT	53	-0.33	-2.91	<0.001	3.29E-04
MODULE_343	19	-0.56	-2.90	<0.001	3.33E-04
GO_CELLULAR_AMINO_ACID_METABOLIC_PROCESS	255	-0.15	-2.89	<0.001	3.69E-04
GO_SULFUR_COMPOUND_BINDING	169	-0.19	-2.89	<0.001	3.66E-04
TGGNNNNNNKCCAR_UNKNOWN	681	-0.10	-2.89	<0.001	3.70E-04
CART1_01	140	-0.21	-2.88	<0.001	3.98E-04
MORF_UBE2N	88	-0.26	-2.88	<0.001	4.20E-04
GNF2_TST	51	-0.34	-2.88	<0.001	4.23E-04
ZHANG_BREAST_CANCER_PROGENITORS_UP	384	-0.13	-2.87	<0.001	4.20E-04
MORF_PTPN11	103	-0.23	-2.86	<0.001	4.35E-04
POU1F1_Q6	147	-0.20	-2.86	<0.001	4.50E-04
MOOTHA_VOXPHOS	83	-0.26	-2.85	<0.001	4.46E-04
RODRIGUES_THYROID_CARCINOMA_POORLY_DIFFERENTIATED_DN	646	-0.10	-2.85	<0.001	4.49E-04
GO_2_OXOGLUTARATE_METABOLIC_PROCESS	18	-0.57	-2.85	<0.001	4.57E-04
GO_MICROBODY_PART	78	-0.27	-2.85	<0.001	4.66E-04

GO_RESPIRATORY_CHAIN	71	-0.29	-2.85	<0.001	4.68E-04
GO_MRNA_PROCESSING	374	-0.13	-2.85	<0.001	4.65E-04
GNF2_TTN	21	-0.52	-2.84	<0.001	4.73E-04
GO_LIPID_CATABOLIC_PROCESS	159	-0.19	-2.84	<0.001	4.81E-04
MORF_SMC1L1	58	-0.32	-2.84	<0.001	4.77E-04
GO_SINGLE_ORGANISM_CATABOLIC_PROCESS	690	-0.10	-2.84	<0.001	4.79E-04
GO_RNA_SPLICING	323	-0.14	-2.83	<0.001	5.04E-04
GO_MITOCHONDRION_ORGANIZATION	500	-0.11	-2.82	<0.001	5.06E-04
GSE22935_WT_VS_MYD88_KO_MACROPHAGE_48H_MBOVIS_BCG_STIM_UP	174	-0.18	-2.82	<0.001	5.03E-04
GO_MONOVALENT_INORGANIC_CATION_TRANSMEMBRANE_TRANSPORTER_A[illegible]	198	-0.17	-2.82	<0.001	5.16E-04
ACEVEDO_FGFR1_TARGETS_IN_PROSTATE_CANCER_MODEL_DN	212	-0.17	-2.82	<0.001	5.34E-04
GSE6674_CPG_VS_PL2_3_STIM_BCELL_UP	165	-0.19	-2.81	<0.001	5.57E-04
GO_IRON_COORDINATION_ENTITY_TRANSPORT	10	-0.71	-2.81	<0.001	5.54E-04
GO_BRANCHED_CHAIN_AMINO_ACID_METABOLIC_PROCESS	23	-0.49	-2.80	<0.001	5.77E-04
MODULE_43	77	-0.27	-2.80	<0.001	5.78E-04
STEGER_ADIPOGENESIS_UP	20	-0.52	-2.80	<0.001	5.74E-04
MODULE_62	85	-0.27	-2.79	<0.001	5.96E-04
GNF2_HPN	57	-0.31	-2.79	<0.001	5.98E-04
GSE19888_CTRL_VS_TCELL_MEMBRANES_ACT_MAST_CELL_PRETREAT_A3R_IN[illegible]	159	-0.19	-2.79	<0.001	6.25E-04
LEE_LIVER_CANCER_DENA_DN	42	-0.37	-2.79	<0.001	6.47E-04
RNTCANNRNNYNATTW_UNKNOWN	381	-0.13	-2.78	<0.001	6.83E-04
GO_DRUG_METABOLIC_PROCESS	17	-0.57	-2.78	<0.001	7.15E-04
CHIANG_LIVER_CANCER_SUBCLASS_PROLIFERATION_DN	99	-0.24	-2.77	<0.001	7.35E-04
REACTOME_PHASE1_FUNCTIONALIZATION_OF_COMPOUNDS	32	-0.41	-2.77	<0.001	7.51E-04
GO_MRNA_METABOLIC_PROCESS	508	-0.11	-2.76	<0.001	7.81E-04
GO_REGULATION_OF_ACYL_COA_BIOSYNTHETIC_PROCESS	13	-0.60	-2.75	<0.001	8.89E-04
KEGG_PPAR_SIGNALING_PATHWAY	50	-0.33	-2.75	<0.001	8.88E-04
GO_PROTEIN_ACETYLATION	107	-0.23	-2.74	<0.001	9.61E-04
GO_PURINE_CONTAINING_COMPOUND_METABOLIC_PROCESS	311	-0.13	-2.73	<0.001	0.001013463
GO_THIOESTER_BIOSYNTHETIC_PROCESS	40	-0.36	-2.73	<0.001	0.001016967
GO_REGULATION_OF_CELLULAR_KETONE_METABOLIC_PROCESS	132	-0.20	-2.72	<0.001	0.001082809
GO_NUCLEOBASE_CONTAINING_SMALL_MOLECULE_METABOLIC_PROCESS	418	-0.12	-2.72	<0.001	0.00114323
KEGG_DRUG_METABOLISM_CYTOCHROME_P450	26	-0.45	-2.71	<0.001	0.001160095
GO_PROTEIN_MONOUBIQUITINATION	47	-0.33	-2.71	<0.001	0.001204895

GSE27241_WT_VS_RORGT_KO_TH17_POLARIZED_CD4_TCELL_DN	151	-0.19	-2.71	<0.001	0.001197765
FOXO1_02	186	-0.17	-2.70	<0.001	0.001227781
MORF_RRM1	96	-0.23	-2.70	<0.001	0.001271463
FOXJ2_01	132	-0.20	-2.69	<0.001	0.001287047
REACTOME_BRANCHED_CHAIN_AMINO_ACID_CATABOLISM	17	-0.53	-2.69	<0.001	0.001279608
GO_U2_TYPE_SPLICEOSOMAL_COMPLEX	28	-0.42	-2.69	<0.001	0.001317553
GO_REGULATION_OF_CARDIAC_CONDUCTION	56	-0.30	-2.69	<0.001	0.001319035
GO_HISTONE_METHYLTRANSFERASE_COMPLEX	59	-0.29	-2.69	<0.001	0.001311541
GO_MICROBODY_LUMEN	31	-0.41	-2.68	<0.001	0.001339801
KEGG_METABOLISM_OF_XENOBIOTICS_BY_CYTOCHROME_P450	23	-0.47	-2.68	<0.001	0.001332274
GO_NAD_BINDING	48	-0.32	-2.68	<0.001	0.001377804
REACTOME_FATTY_ACID_TRIACYLGLYCEROL_AND_KETONE_BODY_METABOLISM	154	-0.19	-2.67	<0.001	0.001427247
GO_FATTY_ACID_BETA_OXIDATION_USING_ACYL_COA_DEHYDROGENASE	16	-0.55	-2.67	<0.001	0.001441212
TAL1BETAE47_01	162	-0.18	-2.67	<0.001	0.001476809
TTCYRGAA_UNKNOWN	798	-0.08	-2.67	<0.001	0.001477395
GO_REGULATION_OF_COFACTOR_METABOLIC_PROCESS	43	-0.33	-2.67	<0.001	0.001495092
GO_NUCLEOPLASM_PART	626	-0.09	-2.66	<0.001	0.001516876
GO_METHYLTRANSFERASE_COMPLEX	76	-0.27	-2.66	<0.001	0.001534366
GO_CELLULAR_ALDEHYDE_METABOLIC_PROCESS	65	-0.28	-2.65	<0.001	0.001669885
GSE37301_MULTIPOTENT_PROGENITOR_VS_CD4_TCELL_DN	173	-0.17	-2.65	<0.001	0.001694657
SENGUPTA_NASOPHARYNGEAL_CARCINOMA_WITH_LMP1_DN	76	-0.25	-2.65	<0.001	0.001727402
SENGUPTA_NASOPHARYNGEAL_CARCINOMA_DN	147	-0.19	-2.65	<0.001	0.001726652
REACTOME_MITOCHONDRIAL_FATTY_ACID_BETA_OXIDATION	14	-0.59	-2.65	<0.001	0.001730055
GO_REGULATION_OF_HEART_CONTRACTION	174	-0.17	-2.64	0.002	0.001758124
CHIANG_LIVER_CANCER_SUBCLASS_CTNNB1_UP	125	-0.20	-2.64	<0.001	0.001785948
OCT1_04	148	-0.18	-2.64	<0.001	0.001776742
GO_REGULATION_OF_LIPID_METABOLIC_PROCESS	222	-0.15	-2.64	<0.001	0.001775703
GO_RNA_SPLICING_VIA_TRANSESTERIFICATION_REACTIONS	241	-0.15	-2.64	<0.001	0.001794915
BOQUEST_STEM_CELL_UP	226	-0.15	-2.63	<0.001	0.001866082
GO_RESPONSE_TO_INSULIN	171	-0.18	-2.63	<0.001	0.001856657
GO_PROTEIN_ACTIVATION_CASCADE	36	-0.36	-2.63	<0.001	0.001938579
GO_LIGASE_ACTIVITY_FORMING_CARBON_SULFUR_BONDS	32	-0.40	-2.62	<0.001	0.002007891
GO_FIBROBLAST_GROWTH_FACTOR_RECEPTOR_BINDING	15	-0.55	-2.62	<0.001	0.002084604
GO_CILIARY_BASAL_BODY	68	-0.27	-2.62	<0.001	0.00208603

GNF2_GSTM1	48	-0.31	-2.61	<0.001	0.00215731
GO_CATALYTIC_COMPLEX	907	-0.08	-2.61	<0.001	0.002185561
SABATES_COLORECTAL_ADENOMA_DN	159	-0.18	-2.60	<0.001	0.002298635
GO_ORGANONITROGEN_COMPOUND_METABOLIC_PROCESS	1327	-0.07	-2.60	<0.001	0.002341284
GO_MONOVALENT_INORGANIC_CATION_TRANSPORT	262	-0.14	-2.60	<0.001	0.002352918
GO_LIPID_MODIFICATION	172	-0.17	-2.60	<0.001	0.002352957
MORF_RAD23A	328	-0.13	-2.60	<0.001	0.002341699
MORF_PRDX3	82	-0.25	-2.59	<0.001	0.002439529
CAIRO_LIVER_DEVELOPMENT_DN	145	-0.19	-2.59	<0.001	0.002427967
GO_OXIDOREDUCTASE_ACTIVITY_ACTING_ON_THE_CH_CH_GROUP_OF_DONOI	48	-0.32	-2.59	<0.001	0.002536058
MOOTHA_TCA	16	-0.53	-2.59	<0.001	0.002524152
KEGG_PYRUVATE_METABOLISM	33	-0.38	-2.58	<0.001	0.002560187
GNF2_MLH1	37	-0.36	-2.58	<0.001	0.002662308
GO_BIOTIN_METABOLIC_PROCESS	11	-0.63	-2.58	<0.001	0.002671936
GO_ALPHA_AMINO_ACID_METABOLIC_PROCESS	166	-0.17	-2.58	<0.001	0.002685237
GO_PRESPLICEOSOME	20	-0.47	-2.58	<0.001	0.002694766
FAC1_01	164	-0.17	-2.58	<0.001	0.002689672
MORF_SKP1A	190	-0.16	-2.57	<0.001	0.002699016
HUMMERICH_BENIGN_SKIN_TUMOR_DN	15	-0.55	-2.57	<0.001	0.002725982
MODULE_137	378	-0.11	-2.57	<0.001	0.002799009
GO_TRNA_METABOLIC_PROCESS	161	-0.17	-2.57	<0.001	0.002786457
GO_SYSTEM_PROCESS	809	-0.08	-2.57	<0.001	0.002798768
GCM_MLL	155	-0.18	-2.56	<0.001	0.002839007
GO_ORGANONITROGEN_COMPOUND_CATABOLIC_PROCESS	240	-0.15	-2.56	<0.001	0.002843826
GO_SMALL_MOLECULE_BIOSYNTHETIC_PROCESS	326	-0.12	-2.56	<0.001	0.002838229
MORF_GMPS	51	-0.30	-2.56	<0.001	0.002936663
WOO_LIVER_CANCER_RECURRENCE_DN	51	-0.30	-2.55	<0.001	0.002989426
FARMER_BREAST_CANCER_CLUSTER_7	15	-0.54	-2.55	<0.001	0.003062212
GO_NUCLEIC_ACID_PHOSPHODIESTER_BOND_HYDROLYSIS	207	-0.15	-2.55	0.001972387	0.003059218
GO_DICARBOXYLIC_ACID_CATABOLIC_PROCESS	11	-0.63	-2.55	<0.001	0.00312781
MORF_XRCC5	221	-0.15	-2.55	<0.001	0.003127963
GO_ACYL_COA_DEHYDROGENASE_ACTIVITY	15	-0.54	-2.54	<0.001	0.003141651
GNF2_RPA1	26	-0.42	-2.54	<0.001	0.003148411
MODULE_212	246	-0.14	-2.54	<0.001	0.003239009

HALLMARK_PEROXISOME	84	-0.23	-2.53	<0.001	0.003392345
SCHMIDT_POR_TARGETS_IN_LIMB_BUD_UP	21	-0.47	-2.53	<0.001	0.003421324
BOYAULT_LIVER_CANCER_SUBCLASS_G123_DN	34	-0.37	-2.52	<0.001	0.003638768
MOOTHA_FFA_OXYDATION	22	-0.46	-2.52	<0.001	0.003643501
REACTOME_REGULATION_OF_PYRUVATE_DEHYDROGENASE_PDH_COMPLEX	12	-0.59	-2.52	<0.001	0.003671185
GO_HYDROGEN_TRANSPORT	102	-0.21	-2.51	<0.001	0.00382573
VERNOCHET_ADIPOGENESIS	16	-0.53	-2.51	<0.001	0.003995457
TNCATNTCCYR_UNKNOWN	179	-0.16	-2.50	<0.001	0.004131571
JAEGER_METASTASIS_DN	138	-0.18	-2.50	<0.001	0.004185841
MORF_CAMK4	157	-0.17	-2.50	<0.001	0.004204209
GSE6674_ANTI_IGM_VS_PL2_3_STIM_BCELL_UP	170	-0.16	-2.50	<0.001	0.004251248
STARK_PREFRONTAL_CORTEX_22Q11_DELETION_DN	439	-0.11	-2.50	<0.001	0.004313745
GSE16385_UNTREATED_VS_12H_ROSIGLITAZONE_IL4_TREATED_MACROPHAGE	148	-0.17	-2.50	<0.001	0.004375918
GO_U2_TYPE_PRESPLICEOSOME	15	-0.54	-2.50	<0.001	0.004380592
REACTOME_NITRIC_OXIDE_STIMULATES_GUANYLATE_CYCLASE	17	-0.51	-2.50	<0.001	0.004363139
LE_EGR2_TARGETS_DN	93	-0.22	-2.49	0.00409836	0.004395916
MORF_EIF3S2	232	-0.14	-2.49	<0.001	0.004381679
GO_OXIDOREDUCTASE_ACTIVITY_ACTING_ON_THE_ALDEHYDE_OR_OXO_GROU	25	-0.42	-2.49	<0.001	0.004557411
GO_CHEMICAL_HOMEOSTASIS	584	-0.09	-2.49	<0.001	0.004554931
GO_PEROXISOMAL_TRANSPORT	17	-0.50	-2.49	<0.001	0.004537139
GO_STEROID_HYDROXYLASE_ACTIVITY	13	-0.57	-2.49	<0.001	0.004544112
GO_ELECTRON_CARRIER_ACTIVITY	89	-0.22	-2.49	<0.001	0.004538823
ICHIBA_GRAFT_VERSUS_HOST_DISEASE_D7_DN	23	-0.44	-2.48	<0.001	0.004585625
GO_RESPONSE_TO_XENOBIOTIC_STIMULUS	54	-0.29	-2.48	<0.001	0.004765646
MODULE_88	527	-0.09	-2.48	<0.001	0.004805058
GO_OXALOACETATE_METABOLIC_PROCESS	11	-0.61	-2.48	<0.001	0.004816811
GO_CELLULAR_RESPONSE_TO_HORMONE_STIMULUS	409	-0.11	-2.47	<0.001	0.005036199
GO_OXIDOREDUCTASE_ACTIVITY_ACTING_ON_THE_ALDEHYDE_OR_OXO_GROU	33	-0.36	-2.47	<0.001	0.00508592
SMID_BREAST_CANCER_LUMINAL_B_UP	109	-0.20	-2.46	<0.001	0.005335638
GO_REGULATION_OF_CARBOHYDRATE_METABOLIC_PROCESS	135	-0.18	-2.46	<0.001	0.005410708
GO_ACETYLTRANSFERASE_COMPLEX	88	-0.23	-2.46	<0.001	0.005494028
GO_PEPTIDE_METABOLIC_PROCESS	433	-0.10	-2.45	<0.001	0.005998546
GSE17721_ALL_VS_24H_PAM3CSK4_BMDC_DN	170	-0.16	-2.45	<0.001	0.005985042
GO_REGULATION_OF_SULFUR_METABOLIC_PROCESS	19	-0.47	-2.44	<0.001	0.006176542

SRY_02	185	-0.15	-2.44	<0.001	0.00615375
GO_CARBON_CARBON_LYASE_ACTIVITY	40	-0.33	-2.44	<0.001	0.006241587
MORF_FBL	129	-0.18	-2.44	<0.001	0.006262102
CAIRO_HEPATOBLASTOMA_DN	177	-0.16	-2.44	<0.001	0.006392518
GO_GLYCOSYL_COMPOUND_METABOLIC_PROCESS	285	-0.12	-2.43	<0.001	0.006513109
KEGG_ALZHEIMERS_DISEASE	137	-0.18	-2.43	<0.001	0.006489511
GO_CILIUM_MOVEMENT	13	-0.54	-2.43	<0.001	0.006543214
DELYS_THYROID_CANCER_DN	182	-0.15	-2.43	<0.001	0.006585349
GO_OXIDOREDUCTASE_COMPLEX	87	-0.22	-2.43	<0.001	0.006575896
GO_RNA_PROCESSING	710	-0.08	-2.43	<0.001	0.006648632
GO_TETRAPYRROLE_BINDING	67	-0.25	-2.42	<0.001	0.006968643
MORF_SUPT3H	181	-0.16	-2.42	<0.001	0.006966421
WWTAAGGC_UNKNOWN	1340	-0.06	-2.42	<0.001	0.006950158
GNF2_DEK	51	-0.28	-2.42	<0.001	0.006970142
GO_VOLTAGE_GATED_POTASSIUM_CHANNEL_ACTIVITY	45	-0.30	-2.42	<0.001	0.007103795
YOSHIMURA_MAPK8_TARGETS_UP	772	-0.08	-2.42	<0.001	0.007078957
GATA1_02	165	-0.16	-2.42	<0.001	0.007054291
YANG_MUC2_TARGETS_DUODENUM_3MO_DN	13	-0.56	-2.42	<0.001	0.007065697
GO_REGULATION_OF_GLUCOSE_METABOLIC_PROCESS	86	-0.23	-2.41	<0.001	0.007118131
HFH1_01	172	-0.16	-2.41	<0.001	0.007109978
MORF_PPP1CC	152	-0.17	-2.41	<0.001	0.007175205
GO_RESPONSE_TO_HORMONE	654	-0.08	-2.41	<0.001	0.007153306
GO_PEPTIDE_N_ACETYLTRANSFERASE_ACTIVITY	56	-0.27	-2.41	<0.001	0.007131603
FARMER_BREAST_CANCER_APOCRINE_VS_BASAL	267	-0.13	-2.41	<0.001	0.00714501
GSE32423_MEMORY_VS_NAIVE_CD8_TCELL_DN	182	-0.15	-2.41	<0.001	0.007161008
FREAC4_01	103	-0.20	-2.41	<0.001	0.007230434
MODULE_55	525	-0.09	-2.40	<0.001	0.007536107
VERHAAK_GLIOBLASTOMA_NEURAL	96	-0.21	-2.40	<0.001	0.007534713
YAATNRNNNYNATT_UNKNOWN	880	-0.07	-2.40	<0.001	0.007565093
TCTGATA_MIR361	78	-0.23	-2.40	<0.001	0.007808814
GSE22935_UNSTIM_VS_48H_MBOVIS_BCG_STIM_MYD88_KO_MACROPHAGE_U	157	-0.16	-2.39	<0.001	0.00810359
TMTCGCGANR_UNKNOWN	178	-0.16	-2.39	<0.001	0.008176562
BURTON_ADIPOGENESIS_5	111	-0.19	-2.39	<0.001	0.008343102
GO_ACETYLTRANSFERASE_ACTIVITY	84	-0.23	-2.38	0.001945525	0.008641133

GO_ORGANOPHOSPHATE_METABOLIC_PROCESS	698	-0.08	-2.38	0.001890359	0.008750346
ACCTGTTG_UNKNOWN	392	-0.10	-2.38	<0.001	0.008788927
GO_SPLICEOSOMAL_COMPLEX	157	-0.16	-2.38	<0.001	0.009002531
GO_CELLULAR_RESPONSE_TO_INSULIN_STIMULUS	125	-0.19	-2.37	0.00209205	0.009243009
GO_COFACTOR_BIOSYNTHETIC_PROCESS	138	-0.17	-2.37	<0.001	0.00923089
GO_REGULATION_OF_HEART_RATE	72	-0.24	-2.37	0.001915709	0.009361757
CHR2Q24	22	-0.42	-2.37	<0.001	0.009382518
HOELZEL_NF1_TARGETS_DN	77	-0.23	-2.37	<0.001	0.009398107
MODULE_432	14	-0.53	-2.36	0.001926782	0.009663861
GO_ACID_THIOL_LIGASE_ACTIVITY	15	-0.52	-2.36	<0.001	0.00990032
MORF_RAN	235	-0.13	-2.36	0.002028398	0.010044752
WANG_CLASSIC_ADIPOGENIC_TARGETS_OF_PPARG	24	-0.41	-2.36	<0.001	0.010043112
MORF_EI24	136	-0.17	-2.34	<0.001	0.010841629
GO_TRANSFERASE_ACTIVITY_TRANSFERRING_NITROGENOUS_GROUPS	15	-0.50	-2.34	<0.001	0.011131032
GO_IRON_ION_HOMEOSTASIS	55	-0.26	-2.33	<0.001	0.011510167
LEE_LIVER_CANCER_MYC_E2F1_DN	35	-0.32	-2.33	<0.001	0.011503888
REACTOME_GLUCOSE_METABOLISM	55	-0.27	-2.33	<0.001	0.011677568
NAKAYAMA_SOFT_TISSUE_TUMORS_PCA1_DN	52	-0.28	-2.33	<0.001	0.011800836
GO_PROTEIN_ACYLATION	133	-0.17	-2.33	<0.001	0.011896575
DODD_NASOPHARYNGEAL_CARCINOMA_UP	986	-0.07	-2.33	<0.001	0.01199165
GO_OXIDOREDUCTASE_ACTIVITY_ACTING_ON_PAIRED_DONORS_WITH_INCORF	10	-0.61	-2.33	<0.001	0.012025274
GO_SHORT_CHAIN_FATTY_ACID_METABOLIC_PROCESS	11	-0.58	-2.32	<0.001	0.012413107
FOXJ2_02	163	-0.16	-2.32	<0.001	0.012447746
CAR_IGFBP1	15	-0.48	-2.32	<0.001	0.01250124
GO_CILIARY_PART	205	-0.14	-2.32	<0.001	0.012463242
GO_VOLTAGE_GATED_CATION_CHANNEL_ACTIVITY	69	-0.24	-2.31	<0.001	0.012780101
SMID_BREAST_CANCER_LUMINAL_A_UP	63	-0.24	-2.31	<0.001	0.01298298
GSE46606_UNSTIM_VS_CD40L_IL2_IL5_3DAY_STIMULATED_IRF4HIGH_SORTED_	101	-0.19	-2.31	<0.001	0.012948588
GTGGGTGK_UNKNOWN	287	-0.12	-2.31	<0.001	0.01298797
CTCNANGTGNY_UNKNOWN	56	-0.26	-2.31	0.002024292	0.013285598
GO_MONOSACCHARIDE_BIOSYNTHETIC_PROCESS	45	-0.29	-2.31	<0.001	0.013257721
FREAC3_01	175	-0.15	-2.30	0.00408998	0.013420451
REACTOME_BIOLOGICAL_OXIDATIONS	63	-0.25	-2.30	<0.001	0.013406438
CHR6Q22	40	-0.31	-2.30	0.002061856	0.013408897

SOX9_B1	184	-0.15	-2.30	0.001953125	0.013469539
PKCA_DN.V1_UP	91	-0.21	-2.30	0.004132231	0.013450825
FARMER_BREAST_CANCER_APOCRINE_VS_LUMINAL	249	-0.12	-2.30	<0.001	0.013664077
GO_CATION_CHANNEL_COMPLEX	98	-0.20	-2.30	<0.001	0.013679445
GO_REGULATION_OF_SYSTEM_PROCESS	357	-0.11	-2.30	<0.001	0.013664829
GO_HISTONE_UBIQUITINATION	31	-0.35	-2.30	<0.001	0.013629722
GO_STEROL_BIOSYNTHETIC_PROCESS	34	-0.33	-2.30	<0.001	0.013608531
TURASHVILI_BREAST_LOBULAR_CARCINOMA_VS_DUCTAL_NORMAL_DN	51	-0.28	-2.30	<0.001	0.013729383
GSE411_UNSTIM_VS_400MIN_IL6_STIM_MACROPHAGE_DN	179	-0.14	-2.30	0.001879699	0.013698882
GO_NUCLEAR_SPECK	176	-0.15	-2.30	0.001915709	0.013779961
MODULE_11	382	-0.10	-2.29	0.004166667	0.013883288
GSE18791_UNSTIM_VS_NEWCATSLE_VIRUS_DC_1H_UP	70	-0.24	-2.29	<0.001	0.013927043
GO_HYDROGEN_ION_TRANSMEMBRANE_TRANSPORT	75	-0.23	-2.29	<0.001	0.014133086
TGANNYRGCA_TCF11MAFG_01	509	-0.09	-2.29	0.001915709	0.014151228
PLASARI_TGFB1_TARGETS_10HR_DN	223	-0.13	-2.29	<0.001	0.01415594
GO_FATTY_ACYL_COA_BINDING	27	-0.37	-2.29	<0.001	0.014386216
GO_PEROXISOME_ORGANIZATION	32	-0.35	-2.28	<0.001	0.014688399
REACTOME_CYTOCHROME_P450_ARRANGED_BY_SUBSTRATE_TYPE	19	-0.44	-2.28	<0.001	0.014826943
GO_ION_TRANSPORT	756	-0.07	-2.28	<0.001	0.015266733
MORF_GNB1	287	-0.11	-2.28	<0.001	0.01528817
MORF_RAD51L3	218	-0.13	-2.28	0.002008032	0.015384455
GO_POLY_A_RNA_BINDING	1013	-0.06	-2.28	<0.001	0.015343952
GO_TRANSFERASE_COMPLEX	634	-0.08	-2.28	0.00203666	0.015308056
GO_PEPTIDYL_LYSINE_MODIFICATION	265	-0.12	-2.27	<0.001	0.01534013
YAUCH_HEDGEHOG_SIGNALING_PARACRINE_DN	149	-0.16	-2.27	<0.001	0.01530004
WAKABAYASHI_ADIPOGENESIS_PPARG_RXRA_BOUND_WITH_H4K20ME1_MARI	137	-0.17	-2.27	0.002053388	0.015397216
REACTOME_CGMP_EFFECTS	11	-0.56	-2.27	<0.001	0.0154525
MCMURRAY_TP53_HRAS_COOPERATION_RESPONSE_DN	52	-0.27	-2.27	<0.001	0.015442559
GO_TRANSFERASE_ACTIVITY_TRANSFERRING_ACYL_GROUPS	182	-0.15	-2.27	0.001984127	0.015551651
LANDIS_ERBB2_BREAST_PRENEOPLASTIC_DN	53	-0.27	-2.27	<0.001	0.01551802
MODULE_306	22	-0.40	-2.27	<0.001	0.015750276
YYCATTCAWW_UNKNOWN	372	-0.10	-2.27	0.001984127	0.01586825
MODULE_99	194	-0.14	-2.26	<0.001	0.016175387
GNF2_PA2G4	74	-0.23	-2.26	0.001919386	0.016267864

LEE_LIVER_CANCER_E2F1_DN	36	-0.31	-2.26	0.002008032	0.016523158
GO_VOLTAGE_GATED_ION_CHANNEL_ACTIVITY	101	-0.19	-2.26	0.001886793	0.016868185
MODULE_100	379	-0.10	-2.26	0.001945525	0.016857022
MORF_PTPRB	140	-0.17	-2.25	0.001996008	0.017115347
MODULE_373	13	-0.53	-2.25	0.003952569	0.01721682
GO_GATED_CHANNEL_ACTIVITY	153	-0.15	-2.25	<0.001	0.017722113
GO_NADH_DEHYDROGENASE_COMPLEX	41	-0.30	-2.25	0.003937008	0.017796345
GO_CHROMATIN_ORGANIZATION	487	-0.09	-2.25	<0.001	0.017822351
GO_NUCLEOSIDE_TRIPHOSPHATE_METABOLIC_PROCESS	186	-0.14	-2.24	<0.001	0.018208945
GO_STEROID_BIOSYNTHETIC_PROCESS	67	-0.23	-2.24	<0.001	0.018179841
GO_MUSCLE_SYSTEM_PROCESS	228	-0.13	-2.24	<0.001	0.018361857
NOUSHMEHR_GBM_SILENCED_BY_METHYLATION	32	-0.33	-2.24	0.00203666	0.018450163
HOWLIN_CITED1_TARGETS_1_UP	26	-0.37	-2.23	0.00203666	0.018938333
GSE32986_CURDLAN_LOWDOSE_VS_GMCSF_AND_CURDLAN_LOWDOSE_STIM_	177	-0.15	-2.23	0.001886793	0.019075789
GSE27786_LSK_VS_MONO_MAC_UP	174	-0.14	-2.23	<0.001	0.019087896
GO_WATER_SOLUBLE_VITAMIN_METABOLIC_PROCESS	66	-0.24	-2.23	<0.001	0.01920012
HFH4_01	134	-0.17	-2.23	0.002024292	0.019431235
MODULE_66	385	-0.10	-2.23	<0.001	0.019426115
GO_EXCITATORY_POSTSYNAPTIC_POTENTIAL	16	-0.47	-2.23	0.004024145	0.01950175
GCTNWTTGK_UNKNOWN	86	-0.21	-2.23	0.006048387	0.019494409
LEE_LIVER_CANCER_CIPROFIBRATE_DN	30	-0.34	-2.23	0.003891051	0.01944884
GO_ROUGH_ENDOPLASMIC_RETICULUM	55	-0.26	-2.23	<0.001	0.019575799
GSE37563_WT_VS_CTLA4_KO_CD4_TCELL_D4_POST_IMMUNIZATION_UP	120	-0.17	-2.23	<0.001	0.019546233
REACTOME_RORA_ACTIVATES_CIRCADIAN_EXPRESSION	22	-0.40	-2.22	<0.001	0.019800238
GO_CATALYTIC_STEP_2_SPLICEOSOME	83	-0.21	-2.22	0.00408998	0.0198022
HAND1E47_01	181	-0.14	-2.22	0.002040816	0.02000293
EVI1_04	163	-0.15	-2.22	<0.001	0.020331565
GSE45365_CTRL_VS_MCMV_INFECTION_NK_CELL_UP	164	-0.15	-2.22	<0.001	0.020359932
GO_RNA_POLYMERASE_II_TRANSCRIPTION_COFACTOR_ACTIVITY	74	-0.22	-2.22	<0.001	0.020561479
GO_COENZYME_BIOSYNTHETIC_PROCESS	100	-0.19	-2.21	<0.001	0.020842819
GSE14769_UNSTIM_VS_20MIN_LPS_BMDM_UP	102	-0.19	-2.21	<0.001	0.020836253
GO_CELLULAR_AMIDE_METABOLIC_PROCESS	548	-0.08	-2.21	0.001992032	0.020841418
MORF_PCNA	79	-0.21	-2.21	<0.001	0.021127544
MORF_UNG	74	-0.22	-2.21	0.001923077	0.021268433

LEE_LIVER_CANCER_MYC_DN	39	-0.30	-2.21	<0.001	0.021367786
GO_REGULATION_OF_MUSCLE_SYSTEM_PROCESS	161	-0.15	-2.21	<0.001	0.021323157
GO_LONG_CHAIN_FATTY_ACID_TRANSPORT	28	-0.34	-2.21	<0.001	0.0212942
GO_RELAXATION_OF_MUSCLE	18	-0.43	-2.21	0.001988072	0.021354046
TAL1ALPHAE47_01	175	-0.14	-2.21	0.003960396	0.021350121
GR_Q6	178	-0.14	-2.21	<0.001	0.02146531
FOXO4_02	192	-0.14	-2.21	0.00390625	0.021497592
KEGG_TRYPTOPHAN_METABOLISM	25	-0.36	-2.21	0.00203252	0.021459062
MISHRA_CARCINOMA_ASSOCIATED_FIBROBLAST_DN	20	-0.41	-2.21	<0.001	0.021466482
SCHUETZ_BREAST_CANCER_DUCTAL_INVASIVE_DN	65	-0.23	-2.20	<0.001	0.021702101
GO_TRANSITION_METAL_ION_HOMEOSTASIS	83	-0.21	-2.20	<0.001	0.02174857
YAGI_AML_WITH_T_9_11_TRANSLOCATION	106	-0.19	-2.20	<0.001	0.021830853
NKX25_02	162	-0.15	-2.20	<0.001	0.021827871
CHRXP11	81	-0.21	-2.20	0.001953125	0.021779673
GO_REGULATION_OF_HORMONE_LEVELS	281	-0.11	-2.20	<0.001	0.021752328
GO_REGULATION_OF_BLOOD_CIRCULATION	223	-0.13	-2.20	0.002008032	0.02171756
GO_REGULATION_OF_MUSCLE_CONTRACTION	116	-0.17	-2.20	<0.001	0.021742998
REACTOME_VOLTAGE_GATED_POTASSIUM_CHANNELS	22	-0.39	-2.20	0.00203252	0.022204831
RICKMAN_HEAD_AND_NECK_CANCER_D	15	-0.47	-2.20	<0.001	0.022232547
SCHAEFFER_PROSTATE_DEVELOPMENT_12HR_UP	94	-0.19	-2.20	<0.001	0.02233259
GSE19888_CTRL_VS_A3R_ACTIVATION_MAST_CELL_DN	97	-0.18	-2.19	<0.001	0.022382274
KRAS.KIDNEY_UP.V1_UP	88	-0.20	-2.19	<0.001	0.02235209
GATA1_01	173	-0.14	-2.19	<0.001	0.02235335
CORRE_MULTIPLE_MYELOMA_DN	47	-0.28	-2.19	0.001988072	0.022408051
MCAATNNNNNGCG_UNKNOWN	450	-0.09	-2.19	0.001964637	0.022690028
LEE_LIVER_CANCER_ACOX1_DN	38	-0.31	-2.19	0.006507592	0.02265759
RICKMAN_TUMOR_DIFFERENTIATED_WELL_VS_POORLY_UP	196	-0.14	-2.19	<0.001	0.022702096
HUMMERICH_MALIGNANT_SKIN_TUMOR_DN	16	-0.45	-2.19	0.00422833	0.022717305
ATGTTAA_MIR302C	188	-0.14	-2.19	<0.001	0.02268875
BOYAULT_LIVER_CANCER_SUBCLASS_G1_DN	36	-0.31	-2.19	<0.001	0.023050245
YAO_HOXA10_TARGETS_VIA_PROGESTERONE_UP	70	-0.23	-2.19	<0.001	0.0230246
GO_VITAMIN_METABOLIC_PROCESS	79	-0.22	-2.19	<0.001	0.023009913
WTTGKCTG_UNKNOWN	503	-0.09	-2.19	<0.001	0.02299177
MODULE_113	55	-0.25	-2.19	<0.001	0.023070773

S8_01	154	-0.15	-2.19	0.002012072	0.02305785
GSE6674_UNSTIM_VS_ANTI_IGM_AND_CPG_STIM_BCELL_UP	178	-0.14	-2.19	<0.001	0.023120249
NKX22_01	132	-0.16	-2.18	<0.001	0.023173373
GO_MITOCHONDRIAL_RESPIRATORY_CHAIN_COMPLEX_ASSEMBLY	64	-0.23	-2.18	<0.001	0.023374364
GSE18804_SPLEEN_MACROPHAGE_VS_COLON_TUMORAL_MACROPHAGE_DN	179	-0.14	-2.18	0.001937985	0.023463823
GO_CELLULAR_MACROMOLECULAR_COMPLEX_ASSEMBLY	549	-0.08	-2.18	0.004040404	0.023553085
MOOTHA_GLUCONEOGENESIS	28	-0.35	-2.18	0.00189394	0.023698417
GSE14350_TREG_VS_TEFF_UP	184	-0.14	-2.18	0.004016064	0.023670183
GSE18281_SUBCAPSULAR_CORTICAL_REGION_VS_WHOLE_CORTEX_THYMUS_U	168	-0.14	-2.18	0.002079002	0.023666749
GO_COMPLEMENT_ACTIVATION	27	-0.35	-2.18	0.002057613	0.02363695
GATA1_04	151	-0.15	-2.18	<0.001	0.023801986
KRAS.600.LUNG.BREAST_UP.V1_DN	130	-0.17	-2.18	<0.001	0.023835083
GSE9601_NFKB_INHIBITOR_VS_PI3K_INHIBITOR_TREATED_HCMV_INF_MONOC	186	-0.14	-2.17	<0.001	0.024333736
GO_CARBOXY_LYASE_ACTIVITY	28	-0.35	-2.17	0.001919386	0.024328955
CHRXQ12	13	-0.51	-2.17	0.002028398	0.024709899
BENPORATH_SUZ12_TARGETS	462	-0.09	-2.17	0.001988072	0.025212402
GO_CHROMATIN_MODIFICATION	440	-0.09	-2.17	0.003846154	0.025276575
GO_FATTY_ACID_TRANSPORT	40	-0.29	-2.17	<0.001	0.025287045
GO_FLAVIN_ADENINE_DINUCLEOTIDE_BINDING	66	-0.23	-2.17	0.005780347	0.025319986
KARLSSON_TGFB1_TARGETS_DN	186	-0.14	-2.16	0.003992016	0.02539893
MORF_ACP1	194	-0.14	-2.16	<0.001	0.025369713
GSE11057_NAIVE_VS_MEMORY_CD4_TCELL_UP	129	-0.17	-2.16	0.003898636	0.025523711
KEGG_GLYOXYLATE_AND_DICARBOXYLATE_METABOLISM	12	-0.53	-2.16	0.002070393	0.02562566
LANDIS_ERBB2_BREAST_TUMORS_324_DN	144	-0.15	-2.16	0.001912046	0.025589118
HNF1_01	136	-0.16	-2.16	0.004376368	0.025661634
MORF_DAP3	181	-0.14	-2.16	0.003984064	0.025858007
WEST_ADRENOCORTICAL_TUMOR_DN	454	-0.09	-2.16	0.004056795	0.026085477
BRIDEAU_IMPRINTED_GENES	41	-0.29	-2.16	0.006097561	0.02622568
GO_ONE_CARBON_METABOLIC_PROCESS	27	-0.35	-2.16	<0.001	0.026341822
GO_CARBON_OXYGEN_LYASE_ACTIVITY	58	-0.24	-2.15	0.003883495	0.026654184
BENPORATH_PRC2_TARGETS	241	-0.12	-2.15	<0.001	0.026676588
CEBP_C	143	-0.16	-2.15	<0.001	0.0268296
GO_OXIDOREDUCTASE_ACTIVITY_ACTING_ON_NAD_P_H_QUINONE_OR_SIMILA	43	-0.28	-2.15	0.001919386	0.026863167
FREAC2_01	185	-0.14	-2.15	0.001972387	0.026988544

MORF_DCC	65	-0.23	-2.15	<0.001	0.02706328
KEGG_RETINOL_METABOLISM	16	-0.44	-2.15	<0.001	0.027109586
MORF_MAGEA8	137	-0.16	-2.15	<0.001	0.027122483
MCBRYAN_PUBERTAL_BREAST_4_5WK_UP	190	-0.14	-2.15	0.001984127	0.027078955
GO_CATION_TRANSMEMBRANE_TRANSPORTER_ACTIVITY	350	-0.10	-2.15	0.001984127	0.027024087
GO_ESTABLISHMENT_OF_TISSUE_POLARITY	14	-0.47	-2.15	<0.001	0.027242923
GO_STEROID_METABOLIC_PROCESS	132	-0.16	-2.15	0.006048387	0.0272506
GO_MYELIN_SHEATH	145	-0.15	-2.15	<0.001	0.027253073
YANG_MUC2_TARGETS_DUODENUM_6MO_DN	13	-0.49	-2.15	<0.001	0.027252316
PLASARI_TGFB1_SIGNALING_VIA_NFIC_1HR_DN	90	-0.20	-2.15	0.001964637	0.027279273
LU_AGING_BRAIN_DN	120	-0.17	-2.15	<0.001	0.027265372
GSE11961_FOLLICULAR_BCELL_VS_GERMINAL_CENTER_BCELL_DAY7_UP	167	-0.14	-2.15	0.001964637	0.027225517
GO_MULTICELLULAR_ORGANISMAL_SIGNALING	81	-0.20	-2.14	0.002145923	0.027411498
GO_TRANSFERASE_ACTIVITY_TRANSFERRING_ACYL_GROUPS_OTHER_THAN_AM	161	-0.15	-2.14	0.003952569	0.027512435
GO_CELLULAR_IRON_ION_HOMEOSTASIS	37	-0.30	-2.14	<0.001	0.027693879
BENPORATH_ES_WITH_H3K27ME3	490	-0.08	-2.14	0.001930502	0.02766476
GSE1925_CTRL_VS_24H_IFNG_STIM_IFNG_PRIMED_MACROPHAGE_UP	154	-0.15	-2.14	<0.001	0.027616479
GO_TRANSMEMBRANE_TRANSPORTER_ACTIVITY	570	-0.08	-2.14	<0.001	0.02790567
CHEMELLO_SOLEUS_VS_EDL_MYOFIBERS_DN	16	-0.44	-2.14	0.003891051	0.027935611
WTGAAAT_UNKNOWN	651	-0.07	-2.14	<0.001	0.028050222
WANG_MLL_TARGETS	213	-0.13	-2.14	0.002132196	0.028027128
GSE411_UNSTIM_VS_400MIN_IL6_STIM_MACROPHAGE_UP	167	-0.14	-2.14	0.002024292	0.028125072
GO_PRESYNAPTIC_ACTIVE_ZONE	18	-0.42	-2.14	<0.001	0.028267493
GSE41867_NAIVE_VS_DAY8_LCMV_ARMSTRONG_EFFECTOR_CD8_TCELL_UP	153	-0.15	-2.14	0.00203252	0.028244141
HFH3_01	135	-0.16	-2.13	0.001992032	0.028860722
CSR_LATE_UP.V1_DN	137	-0.16	-2.13	0.003738318	0.028896201
GO_PROTEIN_IMPORT_INTO_PEROXISOME_MATRIX	12	-0.51	-2.13	<0.001	0.029269375
OCT1_03	155	-0.14	-2.13	0.003921569	0.029460166
GO_RETINA_HOMEOSTASIS	38	-0.29	-2.13	<0.001	0.029665614
OHGUCHI_LIVER_HNF4A_TARGETS_UP	31	-0.32	-2.13	<0.001	0.029606752
DAVICIONI_MOLECULAR_ARMS_VS_ERMS_UP	265	-0.11	-2.13	0.008	0.029581076
KEGG_SPLICEOSOME	118	-0.17	-2.13	0.001919386	0.029593002
MORF_ATF2	188	-0.13	-2.13	<0.001	0.029592335
ENK_UV_RESPONSE_KERATINOCYTE_DN	438	-0.09	-2.13	0.001968504	0.02979724

WENG_POR_TARGETS_GLOBAL_UP	16	-0.45	-2.13	0.004065041	0.029748011
MORF_MDM2	152	-0.15	-2.12	<0.001	0.02992404
GO_RESPONSE_TO_CAFFEINE	18	-0.41	-2.12	0.006048387	0.03010224
ATACTGT_MIR144	172	-0.14	-2.12	0.004008016	0.030097418
GO_INTRINSIC_COMPONENT_OF_PEROXISOMAL_MEMBRANE	14	-0.47	-2.12	0.004140787	0.030162038
GCM_NF2	264	-0.11	-2.12	0.004338395	0.030206544
MILI_PSEUDOPODIA_HAPTOTAXIS_UP	484	-0.08	-2.12	0.003984064	0.030291962
GO_ASPARTATE_FAMILY_AMINO_ACID_CATABOLIC_PROCESS	12	-0.51	-2.12	0.001968504	0.030290065
GSE23925_LIGHT_ZONE_VS_NAIVE_BCELL_DN	173	-0.14	-2.12	0.002004008	0.030233033
MEF2_02	174	-0.14	-2.12	0.002057613	0.03034861
GO_BEHAVIOR	311	-0.11	-2.12	0.002066116	0.03053548
GCM_GSPT1	146	-0.15	-2.12	<0.001	0.03076743
MORF_ANP32B	179	-0.14	-2.12	0.00204918	0.030775076
REACTOME_CHOLESTEROL_BIOSYNTHESIS	19	-0.41	-2.12	0.004040404	0.03093407
GSE5542_UNTREATED_VS_IFNA_TREATED_EPITHELIAL_CELLS_6H_DN	146	-0.15	-2.11	0.003846154	0.031556718
KEGG_CALCIUM_SIGNALING_PATHWAY	120	-0.17	-2.11	0.001897533	0.031694166
GSE5679_CTRL_VS_PPARG_LIGAND_ROSIGLITAZONE_TREATED_DC_DN	173	-0.14	-2.11	0.004237288	0.03205995
MORF_RAD21	170	-0.14	-2.11	0.00204499	0.03202606
GO_REGULATION_OF_STRIATED_MUSCLE_CONTRACTION	71	-0.21	-2.11	0.002040816	0.031972844
TCF11MAFG_01	149	-0.15	-2.11	0.003891051	0.031948194
MORF_MSH2	61	-0.23	-2.11	<0.001	0.03211069
TAL1BETAITF2_01	174	-0.13	-2.11	0.003960396	0.032276783
GSE27786_CD8_TCELL_VS_MONO_MAC_UP	181	-0.13	-2.11	0.004008016	0.032355864
GO_ION_TRANSMEMBRANE_TRANSPORT	471	-0.08	-2.11	0.002016129	0.032298014
GSE14415_INDUCED_VS_NATURAL_TREG_UP	119	-0.17	-2.10	0.003984064	0.03309493
REACTOME_METABOLISM_OF_LIPIDS_AND_LIPOPROTEINS	371	-0.10	-2.10	<0.001	0.033154335
GSE26488_CTRL_VS_PEPTIDE_INJECTION_HDAC7_DELTAP_TG_OT2_THYMOCYT	116	-0.17	-2.10	0.001930502	0.033244733
GNF2_XRCC5	70	-0.21	-2.10	<0.001	0.03319593
GO_RIBONUCLEOPROTEIN_COMPLEX	592	-0.07	-2.10	0.006085193	0.033510994
GO_N_ACETYLTRANSFERASE_ACTIVITY	71	-0.21	-2.10	0.001984127	0.03378834
VERHAAK_GLIOBLASTOMA_PRONEURAL	117	-0.17	-2.10	0.004192872	0.03392511
GO_PRIMARY_ALCOHOL_METABOLIC_PROCESS	28	-0.34	-2.09	0.006369427	0.03402335
GO_POSITIVE_REGULATION_OF_GLUCONEOGENESIS	11	-0.53	-2.09	0.002057613	0.03409794
MORF_TPR	137	-0.15	-2.09	0.008163265	0.03411976

GO_SECONDARY_METABOLIC_PROCESS	23	-0.36	-2.09	0.002159827	0.034202427
BIOCARTA_PPARA_PATHWAY	52	-0.25	-2.09	<0.001	0.034170073
CHR5Q14	27	-0.34	-2.09	0.001848429	0.03425527
RRCCGTTA_UNKNOWN	360	-0.10	-2.09	0.001937985	0.034270883
SMTTTTGT_UNKNOWN	201	-0.13	-2.09	<0.001	0.034254473
GO_REGULATION_OF_CARDIAC_MUSCLE_CONTRACTION	62	-0.23	-2.09	0.00203252	0.034200624
REACTOME_VITAMIN_B5_PANTOTHENATE_METABOLISM	11	-0.52	-2.09	<0.001	0.03452724
CHX10_01	135	-0.15	-2.09	0.001992032	0.034516342
GO_KERATINIZATION	11	-0.52	-2.09	0.002118644	0.0346706
GO_NEUROPEPTIDE_SIGNALING_PATHWAY	14	-0.46	-2.09	0.00204499	0.034880042
MODULE_112	149	-0.15	-2.09	<0.001	0.034864087
RICKMAN_HEAD_AND_NECK_CANCER_F	47	-0.26	-2.09	0.002012072	0.034915328
GSE2405_0H_VS_3H_A_PHAGOCYTOPHILUM_STIM_NEUTROPHIL_DN	90	-0.19	-2.09	0.003759399	0.034937948
GNF2_CYP2B6	21	-0.38	-2.08	0.007648184	0.035522427
REACTOME_GLUCONEOGENESIS	26	-0.34	-2.08	0.002012072	0.03546862
GSE22935_WT_VS_MYD88_KO_MACROPHAGE_DN	163	-0.14	-2.08	0.001912046	0.03546587
MODULE_286	29	-0.32	-2.08	0.003921569	0.035568215
GSE41176_UNSTIM_VS_ANTI_IGM_STIM_TAK1_KO_BCELL_6H_UP	148	-0.15	-2.08	0.006048387	0.035561126
GSE23321_CENTRAL_VS_EFFECTOR_MEMORY_CD8_TCELL_DN	167	-0.14	-2.08	0.006024096	0.03573197
GSE27786_BCELL_VS_NEUTROPHIL_UP	186	-0.13	-2.08	0.002028398	0.03599616
CHR1P31	53	-0.24	-2.08	<0.001	0.036020663
GO_ORGANIC_ACID_TRANSPORT	157	-0.14	-2.08	0.007827789	0.036000278
GO_INORGANIC_CATION_TRANSMEMBRANE_TRANSPORTER_ACTIVITY	297	-0.10	-2.08	0.004264392	0.035988383
LIU_SOX4_TARGETS_DN	267	-0.11	-2.08	0.001984127	0.03628804
GSE11961_MEMORY_BCELL_DAY7_VS_GERMINAL_CENTER_BCELL_DAY40_DN	157	-0.14	-2.08	0.002061856	0.036303557
HNF3_Q6	126	-0.16	-2.08	0.003891051	0.036394224
KUNINGER_IGF1_VS_PDGFB_TARGETS_UP	69	-0.21	-2.08	0.001964637	0.03643034
SOX5_01	198	-0.13	-2.08	0.003976143	0.0363734
GSE20715_0H_VS_6H_OZONE_TLR4_KO_LUNG_DN	168	-0.14	-2.08	0.005847953	0.036553416
GO_DELAYED_RECTIFIER_POTASSIUM_CHANNEL_ACTIVITY	21	-0.38	-2.07	0.002028398	0.03664174
GSE2770_UNTREATED_VS_IL4_TREATED_ACT_CD4_TCELL_2H_DN	177	-0.13	-2.07	0.00409836	0.03658606
GO_TRANSLATIONAL_TERMINATION	87	-0.19	-2.07	0.001926782	0.036838006
GO_PCG_PROTEIN_COMPLEX	42	-0.27	-2.07	<0.001	0.03684131
GSE2585_CD80_HIGH_VS_LOW_MTEC_UP	94	-0.18	-2.07	0.00203666	0.037058804

LEE_LIVER_CANCER_MYC_TGFA_DN	39	-0.28	-2.07	0.002109705	0.03769889
GO_REGULATION_OF_INSULIN_LIKE_GROWTH_FACTOR_RECEPTOR_SIGNALING	13	-0.47	-2.07	0.005952381	0.03766504
GNF2_SPTA1	57	-0.23	-2.07	<0.001	0.037815813
GSE21774_CD62L_POS_CD56_BRIGHT_VS_CD62L_NEG_CD56_DIM_NK_CELL_DI	170	-0.14	-2.07	0.005802708	0.037814777
HADDAD_T_LYMPHOCYTE_AND_NK_PROGENITOR_UP	65	-0.22	-2.07	0.002096436	0.038073577
REACTOME_PROCESSING_OF_CAPPED_INTRON_CONTAINING_PRE_MRNA	125	-0.16	-2.07	0.002169197	0.038191568
FLECHNER_BIOPSY_KIDNEY_TRANSPLANT_OK_VS_DONOR_UP	497	-0.08	-2.07	0.003929273	0.03826582
MODULE_333	21	-0.38	-2.06	0.009765625	0.038422305
HALLMARK_KRAS_SIGNALING_DN	87	-0.18	-2.06	0.006012024	0.039109632
GO_APPENDAGE_DEVELOPMENT	114	-0.17	-2.06	0.0041841	0.039334342
GO_HOMEOSTATIC_PROCESS	924	-0.06	-2.06	0.009689922	0.039709143
GNF2_SPTB	51	-0.24	-2.06	0.005376344	0.039660428
BOCHKIS_FOXA2_TARGETS	320	-0.10	-2.06	0.003921569	0.039653655
GO_PRIMARY_CILIUM	143	-0.15	-2.06	<0.001	0.03964945
ZHANG_GATA6_TARGETS_UP	11	-0.51	-2.06	0.003952569	0.039686657
GTGTTGA_MIR505	87	-0.19	-2.06	0.002008032	0.039634302
GO_CELLULAR_TRANSITION_METAL_ION_HOMEOSTASIS	60	-0.22	-2.05	0.004016064	0.040016755
MORF_ERH	107	-0.17	-2.05	0.003883495	0.04017314
GO_HISTONE_H3_ACETYLATION	42	-0.27	-2.05	0.003838772	0.040168084
GO_AMINO_ACID_ACTIVATION	50	-0.25	-2.05	0.002020202	0.040230677
AREB6_04	190	-0.13	-2.05	0.003913894	0.040411014
MODULE_135	10	-0.54	-2.05	0.006085193	0.04037654
GNF2_ANK1	51	-0.24	-2.05	<0.001	0.040891323
GSE14415_NATURAL_TREG_VS_FOXP3_KO_NATURAL_TREG_DN	116	-0.16	-2.05	0.005617978	0.04111208
KEGG_CARDIAC_MUSCLE_CONTRACTION	56	-0.23	-2.05	0.005976096	0.04107524
GO_NUCLEOSIDE_MONOPHOSPHATE_METABOLIC_PROCESS	201	-0.13	-2.05	0.001964637	0.041031964
GO_ORGANIC_HYDROXY_COMPOUND_METABOLIC_PROCESS	320	-0.10	-2.05	0.002008032	0.04119747
KUROKAWA_LIVER_CANCER_EARLY_RECURRENCE_UP	11	-0.50	-2.05	0.007952286	0.041227456
KEGG_NITROGEN_METABOLISM	14	-0.44	-2.05	0.004175365	0.041459627
GO_REGULATION_OF_SYNAPSE_ASSEMBLY	43	-0.26	-2.05	0.006423983	0.041421175
REACTOME_TIGHT_JUNCTION_INTERACTIONS	11	-0.51	-2.05	0.005988024	0.041479245
MORF_FSHR	154	-0.14	-2.04	0.002012072	0.041472092
GO_OXIDOREDUCTASE_ACTIVITY_ACTING_ON_A_HEME_GROUP_OF_DONORS	20	-0.38	-2.04	0.007782101	0.04165642
GO_POTASSIUM_CHANNEL_COMPLEX	53	-0.23	-2.04	0.003773585	0.041831862

GO_REGULATION_OF_FATTY_ACID_OXIDATION	26	-0.34	-2.04	0.005988024	0.041996386
REACTOME_MRNA_SPLICING	99	-0.18	-2.04	0.005813954	0.042117666
GO_HISTONE_H4_ACETYLATION	42	-0.27	-2.04	<0.001	0.042468164
GSE40666_WT_VS_STAT4_KO_CD8_TCELL_DN	165	-0.13	-2.04	0.001996008	0.042454377
NF1_Q6	187	-0.12	-2.04	0.006465518	0.042648945
KEGG_ALANINE_ASPARTATE_AND_GLUTAMATE_METABOLISM	25	-0.34	-2.04	0.002028398	0.042663164
GO_RNA_POLYMERASE_BINDING	31	-0.30	-2.04	0.005847953	0.042597823
GERHOLD_ADIPOGENESIS_UP	49	-0.25	-2.04	0.003944773	0.04263122
REACTOME_PYRUVATE_METABOLISM	17	-0.41	-2.04	0.001964637	0.04274497
MCBRYAN_PUBERTAL_BREAST_4_5WK_DN	174	-0.14	-2.04	0.004	0.0429453
HP1SITEFACTOR_Q6	157	-0.14	-2.03	0.008064516	0.0436553
GO_PHOSPHOLIPID_CATABOLIC_PROCESS	22	-0.37	-2.03	0.002079002	0.043713417
MORF_MAP2K2	127	-0.16	-2.03	0.007692308	0.043672524
GO_POSITIVE_REGULATION_OF_FATTY_ACID_METABOLIC_PROCESS	25	-0.34	-2.03	0.003984064	0.04363163
GO_CILIUM	294	-0.11	-2.03	0.001949318	0.043795887
MORF_PPP1CA	158	-0.14	-2.03	<0.001	0.043797843
TEF_Q6	165	-0.13	-2.03	0.003944773	0.043878023
GSE9601_UNTREATED_VS_NFKB_INHIBITOR_TREATED_HCMV_INF_MONOCYTE_	165	-0.14	-2.03	0.003944773	0.043874815
AAANWWTGC_UNKNOWN	149	-0.14	-2.03	0.003913894	0.04401751
YAUCH_HEDGEHOG_SIGNALING_PARACRINE_UP	88	-0.18	-2.03	0.005825243	0.044229954
GO_ORGANIC_HYDROXY_COMPOUND_BIOSYNTHETIC_PROCESS	121	-0.16	-2.03	0.006134969	0.044392776
GO_FATTY_ACID_LIGASE_ACTIVITY	11	-0.50	-2.03	0.008130081	0.04467936
GSE37301_RAG2_KO_VS_RAG2_AND_ETS1_KO_NK_CELL_DN	169	-0.14	-2.02	0.003913894	0.045033567
GO_PROTEIN_TRANSPORT_ALONG_MICROTUBULE	25	-0.34	-2.02	0.004032258	0.045048483
MIKKELSEN_MEF_ICP_WITH_H3K4ME3_AND_H3K27ME3	19	-0.38	-2.02	0.007393715	0.045055985
CEBPA_01	176	-0.13	-2.02	0.004140787	0.04547598
BONOME_OVARIAN_CANCER_POOR_SURVIVAL_UP	31	-0.30	-2.02	0.005940594	0.04561574
GTACAGG_MIR486	48	-0.24	-2.02	0.002074689	0.045612328
NFY_C	181	-0.13	-2.02	0.001964637	0.04559533
GO_ACTIVE_TRANSMEMBRANE_TRANSPORTER_ACTIVITY	214	-0.12	-2.02	0.005976096	0.046139598
GO_CELLULAR_MODIFIED_AMINO_ACID_METABOLIC_PROCESS	146	-0.14	-2.02	0.004132231	0.04640918
GO_CATION_TRANSPORT	481	-0.08	-2.02	0.011070111	0.046564642
GO_LIGASE_ACTIVITY_FORMING_CARBON_OXYGEN_BONDS	43	-0.26	-2.02	0.00204918	0.04693893
SMITH_LIVER_CANCER	37	-0.28	-2.02	0.005976096	0.04689427

CHR9P22	15	-0.43	-2.01	0.002024292	0.047005527
MYAATNNNNNNNGGC_UNKNOWN	303	-0.10	-2.01	0.001872659	0.046956
GO_MITOCHONDRIAL_RESPIRATORY_CHAIN_COMPLEX_I_BIOGENESIS	50	-0.24	-2.01	0.009652509	0.047292925
WENG_POR_TARGETS_LIVER_UP	31	-0.30	-2.01	0.007797271	0.047769126
HNF3ALPHA_Q6	148	-0.14	-2.01	0.003831418	0.04801094
STEIN_ESRRA_TARGETS_UP	324	-0.10	-2.01	<0.001	0.04809279
GSE19888_ADENOSINE_A3R_INH_VS_ACT_WITH_INHIBITOR_PRETREATMENT_I	146	-0.14	-2.01	0.005802708	0.048891284
GSE17721_12H_VS_24H_LPS_BMDC_DN	175	-0.13	-2.00	0.004040404	0.04920445
EPPERT_CE_HSC_LSC	29	-0.31	-2.00	0.003875969	0.04919316
GO_TRANSPORTER_COMPLEX	170	-0.13	-2.00	0.002012072	0.049186833
GSE13522_CTRL_VS_T_CRUZI_Y_STRAIN_INF_SKIN_BALBC_MOUSE_DN	93	-0.18	-2.00	0.003929273	0.049374893
GSE3982_MAC_VS_BASOPHIL_DN	142	-0.15	-2.00	0.00814664	0.049438275
MODY_HIPPOCAMPUS_POSTNATAL	54	-0.24	-2.00	0.001992032	0.04954577
GSE45365_HEALTHY_VS_MCMV_INFECTION_CD11B_DC_UP	128	-0.15	-2.00	0.006060606	0.049588114
ENK_UV_RESPONSE_KERATINOCYTE_UP	415	0.08	1.86	0.001949318	0.049994744
GO_NEURON_PROJECTION_MORPHOGENESIS	279	0.10	1.86	0.008350731	0.04996444
TAGHAVI_NEOPLASTIC_TRANSFORMATION	11	0.46	1.86	0.01192843	0.04996511
GSE9878_CTRL_VS_EBF_TRANSDUCED_PAX5_KO_PRO_BCELL_UP	156	0.13	1.86	0.009940358	0.049951088
GO_PROTEIN_LOCALIZATION_TO_KINETOCHORE	10	0.48	1.86	0.01764706	0.049767923
JAZAG_TGFB1_SIGNALING_VIA_SMAD4_DN	49	0.22	1.86	0.029469548	0.049730048
GO_MYELOID_DENDRITIC_CELL_DIFFERENTIATION	11	0.46	1.86	0.012448133	0.049734917
REACTOME_RIP_MEDIATED_NFKB_ACTIVATION_VIA_DAI	14	0.42	1.86	0.01629328	0.049573924
PID_GLYPICAN_1PATHWAY	23	0.33	1.86	0.006012024	0.049521554
GSE17721_CTRL_VS_LPS_8H_BMDC_DN	149	0.13	1.86	0.010204081	0.04942217
GSE25123_ROSIGLITAZONE_VS_IL4_AND_ROSIGLITAZONE_STIM_MACROPHAGE	79	0.18	1.86	0.014799154	0.049373426
NIKOLSKY_BREAST_CANCER_20Q12_Q13_AMPLICON	82	0.18	1.86	0.011741683	0.049333185
GSE40443_INDUCED_VS_TOTAL_TREG_DN	162	0.13	1.86	0.01705757	0.049155302
GSE5542_UNTREATED_VS_IFNA_TREATED_EPITHELIAL_CELLS_24H_UP	172	0.12	1.86	0.013513514	0.049045566
GSE45739_NRAS_KO_VS_WT_UNSTIM_CD4_TCELL_UP	170	0.12	1.86	0.010373444	0.04877695
GO_GANGLIOSIDE_METABOLIC_PROCESS	20	0.35	1.86	0.012072435	0.048735317
GSE19941_UNSTIM_VS_LPS_AND_IL10_STIM_IL10_KO_MACROPHAGE_UP	145	0.13	1.86	0.012269938	0.048756145
GO_REGULATION_OF_CELL_KILLING	34	0.27	1.86	0.002040816	0.04877485
GSE28783_CTRL_ANTI_MIR_VS_UNTREATED_ATHEROSCLEROSIS_MACROPHAGE	137	0.14	1.86	0.009784736	0.0487517
KIM_LRRC3B_TARGETS	18	0.36	1.86	0.011976048	0.04877098

GSE25123_CTRL_VS_ROSIGLITAZONE_STIM_MACROPHAGE_UP	178	0.12	1.86	0.017716536	0.048746698
GSE14000_TRANSLATED_RNA_VS_MRNA_4H_LPS_DC_DN	123	0.14	1.86	0.008	0.048671912
MODULE_108	27	0.30	1.87	0.01183432	0.0486222
GO_REGULATION_OF_CELLULAR_PROTEIN_LOCALIZATION	465	0.08	1.87	0.009708738	0.048592012
VECCHI_GASTRIC_CANCER_ADVANCED_VS_EARLY_UP	141	0.13	1.87	0.010309278	0.04860699
DELACROIX_RAR_TARGETS_UP	38	0.26	1.87	0.006224067	0.048481073
GSE40666_STAT1_KO_VS_STAT4_KO_CD8_TCELL_WITH_IFNA_STIM_90MIN_DN	100	0.16	1.87	0.013833992	0.04846086
REACTOME_SIGNALING_BY_RHO_GTPASES	98	0.16	1.87	0.00591716	0.04845664
GO_REGULATION_OF_CELL_CELL_ADHESION	253	0.10	1.87	0.017374517	0.0483044
REACTOME_IKK_COMPLEX_RECRUITMENT_MEDIATED_BY_RIP1	10	0.49	1.87	0.015503876	0.04832246
GO_POSITIVE_REGULATION_OF_NF_KAPPAB_IMPORT_INTO_NUCLEUS	17	0.38	1.87	0.009803922	0.04834218
GO_SIGNALING_RECEPTOR_ACTIVITY	482	0.08	1.87	0.00952381	0.04834166
GO_RUFFLE_MEMBRANE	73	0.18	1.87	0.007905139	0.048360333
GSE9988_LPS_VS_LPS_AND_ANTI_TREM1_MONOCYTE_UP	169	0.13	1.87	0.016949153	0.048372597
GSE6090_UNSTIM_VS_DC-SIGN_STIM_DC_DN	68	0.19	1.87	0.009746589	0.048366208
GSE5142_CTRL_VS_HTERT_TRANSDUCED_CD8_TCELL_EARLY_PASSAGE_CLONE_	171	0.12	1.87	0.006465518	0.04835175
GO_REGULATION_OF_CELLULAR_COMPONENT_BIOGENESIS	625	0.06	1.87	0.008333334	0.048219673
GSE8868_SPLEEN_VS_INTESTINE_CD11B_POS_CD11C_NEG_DC_DN	169	0.12	1.87	0.01004016	0.048209425
GO_ATP_DEPENDENT_MICROTUBULE_MOTOR_ACTIVITY	15	0.39	1.87	0.022821577	0.04815053
GO_NEURON_PROJECTION_DEVELOPMENT	382	0.08	1.87	0.002004008	0.048087154
GSE14308_TH1_VS_TH17_UP	168	0.13	1.87	0.004106776	0.047754105
GSE23568_ID3_TRANSDUCED_VS_ID3_KO_CD8_TCELL_UP	159	0.13	1.87	0.009861933	0.047773663
GSE24671_BAKIMULC_VS_SENDAI_VIRUS_INFECTED_MOUSE_SPLENOCYTES_UF	164	0.13	1.87	0.012295082	0.047777683
GO_ARP2_3_PROTEIN_COMPLEX	10	0.49	1.87	0.009881423	0.047792975
ZHANG_TLX_TARGETS_UP	94	0.17	1.87	0.014799154	0.04777875
GSE34205_RSV_VS_FLU_INF_INFANT_PBMC_DN	133	0.14	1.87	0.015873017	0.047781706
DEMAGALHAES_AGING_UP	43	0.23	1.87	0.008264462	0.047800247
GSE22432_CONVENTIONAL_CDC_VS_PLASMACYTOID_PDC_DN	169	0.13	1.87	0.014084507	0.047733445
GO_PROTEIN_UBIQUITINATION_INVOLVED_IN_UBIQUITIN_DEPENDENT_PROTEI	120	0.15	1.87	0.010330578	0.04771915
GO_REGULATION_OF_SEQUENCE_SPECIFIC_DNA_BINDING_TRANSCRIPTION_FA	281	0.10	1.87	0.010204081	0.047738228
GO_POSITIVE_REGULATION_OF_AXON_EXTENSION	35	0.27	1.87	0.005836576	0.04759182
SIG_CHEMOTAXIS	38	0.25	1.87	0.00204499	0.047477435
LFA1_Q6	168	0.12	1.87	0.013182675	0.0474905
GSE35543_IN_VITRO_ITREG_VS_CONVERTED_EX_ITREG_UP	170	0.12	1.87	0.007874016	0.047440078

REACTOME_SIGNAL_AMPLIFICATION	24	0.31	1.87	0.016064256	0.047404602
GSE17721_PAM3CSK4_VS_CPG_4H_BMDC_DN	163	0.13	1.87	0.020120725	0.047372904
GSE32423_MEMORY_VS_NAIVE_CD8_TCELL_UP	136	0.14	1.87	0.00967118	0.047387548
MODULE_317	335	0.09	1.87	0.014373717	0.047384404
GSE8685_IL15_ACT_IL2_STARVED_VS_IL21_ACT_IL2_STARVED_CD4_TCELL_UP	133	0.14	1.87	0.012295082	0.04727179
GO_CELL_CYCLE	1083	0.05	1.87	0.005836576	0.047277797
GSE22045_TREG_VS_TCONV_UP	118	0.15	1.87	0.00845666	0.04725516
PID_ERBB1_DOWNSTREAM_PATHWAY	103	0.16	1.87	0.006185567	0.047163814
MORF_PPP2R4	44	0.24	1.87	0.00814664	0.0471784
GSE36095_WT_VS_HDAC9_KO_TREG_UP	158	0.13	1.87	0.004115226	0.047084138
RUTELLA_RESPONSE_TO_CSF2RB_AND_IL4_UP	281	0.10	1.87	0.013307985	0.047061425
GNF2_FEN1	53	0.22	1.87	0.008032128	0.047061358
BIOCARTA_PS1_PATHWAY	12	0.46	1.87	0.010245902	0.047025498
GO_RESPONSE_TO_INTERFERON_BETA	17	0.38	1.88	0.010080645	0.046940286
GSE9988_LPS_VS_CTRL_TREATED_MONOCYTE_DN	174	0.13	1.88	0.013944224	0.046813764
GO_REGULATION_OF_CELL_JUNCTION_ASSEMBLY	61	0.20	1.88	0.010416667	0.046795182
GSE12001_MIR223_KO_VS_WT_NEUTROPHIL_UP	157	0.13	1.88	0.00591716	0.046800997
KRISHNAN_FURIN_TARGETS_UP	10	0.49	1.88	0.008179959	0.046755824
GO_REGULATION_OF_NITRIC_OXIDE_SYNTHASE_BIOSYNTHETIC_PROCESS	15	0.40	1.88	0.015936255	0.04670948
GSE46606_UNSTIM_VS_CD40L_IL2_IL5_DAY3_STIMULATED_BCELL_DN	162	0.13	1.88	0.011650485	0.046662487
HALLMARK_UNFOLDED_PROTEIN_RESPONSE	107	0.16	1.88	0.010162601	0.046649728
GSE40274_CTRL_VS_FOXP3_AND_EOS_TRANSDUCED_ACTIVATED_CD4_TCELL_[illegible]	134	0.14	1.88	0.00811359	0.046482664
PRAMOONJAGO_SOX4_TARGETS_UP	50	0.22	1.88	0.011811024	0.046431195
AP1_Q4	200	0.12	1.88	0.009940358	0.04640854
RODRIGUES_NTN1_AND_DCC_TARGETS	24	0.33	1.88	0.008097166	0.04639847
GSE411_WT_VS_SOCS3_KO_MACROPHAGE_IL6_STIM_400MIN_UP	172	0.13	1.88	0.02020202	0.046379577
HELLER_HDAC_TARGETS_SILENCED_BY_METHYLATION_DN	232	0.11	1.88	0.007889546	0.046302862
REACTOME_REGULATION_OF_SIGNALING_BY_CBL	18	0.37	1.88	0.015873017	0.046214227
GO_POSITIVE_REGULATION_OF_CYTOKINE_SECRETION	60	0.21	1.88	0.008048289	0.0462203
TSAI_RESPONSE_TO_IONIZING_RADIATION	123	0.14	1.88	0.014227643	0.046201393
GSE360_L_DONOVANI_VS_B_MALAYI_HIGH_DOSE_DC_UP	143	0.14	1.88	0.006160164	0.04613811
HOSHIDA_LIVER_CANCER_LATE_RECURRENCE_UP	50	0.22	1.88	0.014	0.04612951
GSE10240_CTRL_VS_IL17_AND_IL22_STIM_PRIMARY_BRONCHIAL_EPITHELIAL_[illegible]	164	0.12	1.88	0.007874016	0.04608682
MODULE_126	149	0.13	1.88	0.01183432	0.04603876

DORN_ADENOVIRUS_INFECTION_48HR_DN	37	0.26	1.88	0.02079002	0.045916744
GSE25123_WT_VS_PPARG_KO_MACROPHAGE_UP	135	0.14	1.88	0.013333334	0.04590086
GSE36078_WT_VS_IL1R_KO_LUNG_DC_UP	129	0.14	1.88	0.011605416	0.04563029
GO_ESTABLISHMENT_OF_PROTEIN_LOCALIZATION	1148	0.05	1.88	0.016666668	0.045612574
LEE_METASTASIS_AND_ALTERNATIVE_SPLICING_UP	65	0.19	1.88	0.008281574	0.04559869
GSE360_L_DONOVANI_VS_L_MAJOR_MAC_DN	133	0.14	1.88	0.01004016	0.04534621
GO_CELL_CYCLE_PHASE_TRANSITION	231	0.11	1.88	0.00967118	0.0453624
GO_KERATAN_SULFATE_BIOSYNTHETIC_PROCESS	21	0.34	1.88	0.008032128	0.04518104
GSE22527_ANTI_CD3_INVIVO_VS_UNTREATED_MOUSE_TREG_UP	131	0.14	1.88	0.012145749	0.045128483
REACTOME_ACTIVATION_OF_IRF3_IRF7_MEDIATED_BY_TBK1_IKK_EPSILON	12	0.45	1.88	0.004273505	0.045080334
TAKEDA_TARGETS_OF_NUP98_HOXA9_FUSION_10D_DN	85	0.17	1.89	0.00886918	0.04502116
GO_SMALL_GTPASE_MEDIATED_SIGNAL_TRANSDUCTION	294	0.10	1.89	0.005802708	0.045036677
GO_MICROTUBULE_END	21	0.34	1.89	0.014028057	0.044857465
GO_SPINDLE	247	0.10	1.89	0.010224949	0.044796992
GO_GANGLIOSIDE_BIOSYNTHETIC_PROCESS	14	0.41	1.89	0.012219959	0.044700574
GSE22025_TGFB1_VS_TGFB1_AND_PROGESTERONE_TREATED_CD4_TCELL_DN	136	0.14	1.89	0.006382979	0.04468461
GSE6674_UNSTIM_VS_CPG_STIM_BCELL_UP	112	0.15	1.89	0.005952381	0.044586986
ROZANOV_MMP14_TARGETS_UP	202	0.11	1.89	0.01417004	0.044529032
GO_INTRACELLULAR_PROTEIN_TRANSPORT	638	0.07	1.89	0.007662835	0.044518963
GSE28726_NAIVE_VS_ACTIVATED_CD4_TCELL_UP	168	0.13	1.89	0.014736842	0.04453208
VERNELL_RETINOBLASTOMA_PATHWAY_DN	18	0.37	1.89	0.010121457	0.044531412
GSE6259_FLT3L_INDUCED_33D1_POS_DC_VS_BCELL_UP	166	0.13	1.89	0.01039501	0.044494305
PID_LYSOPHOSPHOLIPID_PATHWAY	60	0.21	1.89	0.018036073	0.044504162
GO_RESPONSE_TO_INTERLEUKIN_1	81	0.18	1.89	0.011741683	0.044501323
WEIGEL_OXIDATIVE_STRESS_BY_HNE_AND_TBH	53	0.22	1.89	0.007648184	0.044493947
GO_REGULATION_OF_PROTEIN_BINDING	141	0.14	1.89	0.017110266	0.044379942
GO_CELL_MIGRATION_INVOLVED_IN_SPROUTING_ANGIOGENESIS	13	0.43	1.89	0.010504202	0.044322867
GSE360_CTRL_VS_B_MALAYI_HIGH_DOSE_MAC_DN	165	0.13	1.89	0.011538462	0.044315465
CHARAFE_BREAST_CANCER_BASAL_VS_MESENCHYMAL_DN	41	0.25	1.89	0.009505703	0.044185627
GO_BANDED_COLLAGEN_FIBRIL	10	0.50	1.89	0.013944224	0.044106163
GSE12845_IGD_NEG_BLOOD_VS_PRE_GC_TONSIL_BCELL_DN	170	0.13	1.89	0.00998004	0.043864112
GSE3720_UNSTIM_VS_PMA_STIM_VD1_GAMMADELTA_TCELL_UP	144	0.14	1.89	0.00610998	0.043870933
GO_REGULATION_OF_CYTOKINE_PRODUCTION_INVOLVED_IN_INFLAMMATORY	12	0.45	1.89	0.006147541	0.04389052
REACTOME_INTRINSIC_PATHWAY_FOR_APOPTOSIS	23	0.33	1.89	0.017964073	0.04383569

GSE11057_PBMC_VS_MEM_CD4_TCELL_UP	147	0.13	1.89	0.01048218	0.043764763
ETS_Q4	212	0.12	1.89	0.003968254	0.04374483
BURTON_ADIPOGENESIS_7	45	0.24	1.89	0.013944224	0.04358929
VANOEVELEN_MYOGENESIS_SIN3A_TARGETS	201	0.12	1.89	0.013618677	0.043600433
SANA_TNF_SIGNALING_DN	80	0.18	1.89	0.009861933	0.04352095
WESTON_VEGFA_TARGETS_3HR	61	0.21	1.89	0.012738854	0.04353765
GO_POSITIVE_REGULATION_OF_CHEMOKINE_PRODUCTION	37	0.26	1.89	0.017021276	0.04346857
GSE17721_POLYIC_VS_GARDIQUIMOD_0.5H_BMDC_DN	174	0.12	1.90	0.006289308	0.043071683
GSE29618_BCELL_VS_PDC_DN	172	0.12	1.90	0.013833992	0.043056946
REACTOME_G_ALPHA_Q_SIGNALLING_EVENTS	88	0.17	1.90	0.010183299	0.04294961
GNF2_CASP8	23	0.32	1.90	0.00990099	0.04283045
GSE14000_UNSTIM_VS_16H_LPS_DC_DN	163	0.13	1.90	0.005976096	0.042815626
GSE15330_LYMPHOID_MULTIPOTENT_VS_GRANULOCYTE_MONOCYTE_PROGEN	172	0.12	1.90	0.021526419	0.042779498
KEGG_COLORECTAL_CANCER	58	0.22	1.90	0.003868472	0.042792574
GSE3982_MAC_VS_NKCELL_UP	167	0.13	1.90	0.012219959	0.042715702
GSE10240_IL22_VS_IL22_AND_IL17_STIM_PRIMARY_BRONCHIAL_EPITHELIAL_C	151	0.13	1.90	0.008298756	0.04272541
GESERICK_TERT_TARGETS_DN	21	0.34	1.90	0.010752688	0.04265
GSE17301_ACD3_ACD28_VS_ACD3_ACD28_AND_IFNA5_STIM_CD8_TCELL_UP	147	0.14	1.90	0.004016064	0.042568985
GO_RIBOSOMAL_LARGE_SUBUNIT_BIOGENESIS	39	0.26	1.90	0.007766991	0.042540595
GO_PROTEIN_LOCALIZATION	1466	0.05	1.90	0.014141414	0.04242972
GO_REGULATION_OF_LYMPHOCYTE_APOPTOTIC_PROCESS	38	0.26	1.90	0.00407332	0.042370345
GSE12392_WT_VS_IFNB_KO_CD8A_NEG_SPLEEN_DC_UP	184	0.12	1.90	0.01002004	0.04236364
MODULE_257	145	0.13	1.90	0.008298756	0.042355813
PID_AVB3_OPN_PATHWAY	30	0.30	1.90	0.02008032	0.042362016
GO_ENDOTHELIAL_TUBE_MORPHOGENESIS	11	0.47	1.90	0.005836576	0.041962124
REACTOME_KINESINS	21	0.35	1.90	0.008097166	0.04184281
GO_POSITIVE_REGULATION_OF_ALPHA_BETA_T_CELL_DIFFERENTIATION	24	0.33	1.90	0.00811359	0.041823518
GO_NUCLEOLAR_PART	59	0.21	1.90	0.01984127	0.041821033
GSE42088_UNINF_VS_LEISHMANIA_INF_DC_24H_DN	164	0.13	1.90	0.012195122	0.041738037
GSE41867_DAY6_EFFECTOR_VS_DAY30_MEMORY_CD8_TCELL_LCMV_ARMSTR(	177	0.12	1.90	0.009596929	0.04170396
GSE17721_LPS_VS_GARDIQUIMOD_12H_BMDC_UP	167	0.13	1.90	0.013333334	0.041678347
LIU_BREAST_CANCER	19	0.36	1.90	0.008179959	0.041687623
GO_REGULATION_OF_ENDOCYTOSIS	155	0.13	1.90	0.007707129	0.041590963
MODULE_165	46	0.23	1.90	0.007827789	0.0415912

GO_REGULATION_OF_MYOBLAST_FUSION	12	0.45	1.90	0.007952286	0.041563112
GO_NEGATIVE_REGULATION_OF_ENDOCYTOSIS	30	0.30	1.90	0.01446281	0.041502394
GO_CYTOKINE_RECEPTOR_BINDING	148	0.13	1.90	0.009560229	0.041515004
GSE46606_IRF4HIGH_VS_IRF4MID_CD40L_IL2_IL5_DAY3_STIMULATED_BCELL_D	157	0.13	1.90	0.00591716	0.041388616
KEGG_SMALL_CELL_LUNG_CANCER	79	0.18	1.91	0.006072875	0.041364383
SASAI_RESISTANCE_TO_NEOPLASTIC_TRANSFROMATION	47	0.23	1.91	0.009746589	0.041318066
GO_RNA_CATABOLIC_PROCESS	178	0.12	1.91	0.01183432	0.041304577
GSE37532_TREG_VS_TCONV_CD4_TCELL_FROM_LN_UP	161	0.13	1.91	0.011881189	0.041124057
GSE45739_UNSTIM_VS_ACD3_ACD28_STIM_NRAS_KO_CD4_TCELL_DN	129	0.14	1.91	0.014227643	0.041088365
GSE17721_PAM3CSK4_VS_GADIQUIMOD_1H_BMDC_UP	137	0.14	1.91	0.005758158	0.04102504
GO_PROTEIN_LOCALIZATION_TO_ORGANELLE	454	0.08	1.91	0.008016032	0.040936522
MODULE_92	78	0.18	1.91	0.003846154	0.040905744
GSE21546_WT_VS_SAP1A_KO_AND_ELK1_KO_ANTI_CD3_STIM_DP_THYMOCYT	167	0.13	1.91	0.006134969	0.0408319
SASSON_RESPONSE_TO_FORSKOLIN_UP	82	0.18	1.91	0.00617284	0.04083515
GO_PLASMA_MEMBRANE_RAFT	76	0.19	1.91	0.013645224	0.04084694
GSE24574_BCL6_HIGH_TFH_VS_TCONV_CD4_TCELL_UP	141	0.14	1.91	0.007952286	0.040837187
PETROVA_PROX1_TARGETS_DN	56	0.21	1.91	0.002040816	0.04084218
GSE45382_UNTREATED_VS_TGFB_TREATED_MACROPHAGES_UP	180	0.12	1.91	0.013043478	0.040814776
KYNG_WERNER_SYNDROM_UP	16	0.39	1.91	0.011560693	0.040735487
GSE32164_ALTERNATIVELY_ACT_M2_VS_CMYC_INHIBITED_MACROPHAGE_DN	168	0.13	1.91	0.01369863	0.040601432
GSE11057_NAIVE_CD4_VS_PBMC_CD4_TCELL_DN	174	0.13	1.91	0.012371134	0.04048327
LIM_MAMMARY_LUMINAL_MATURE_DN	89	0.17	1.91	0.009633912	0.040457305
GSE40277_EOS_AND_LEF1_TRANSDUCED_VS_CTRL_CD4_TCELL_DN	177	0.13	1.91	0.004310345	0.040414236
GO_ESTABLISHMENT_OF_LOCALIZATION_IN_CELL	1346	0.05	1.91	0.009416196	0.040409945
MODULE_378	332	0.09	1.91	0.005758158	0.040345665
TGTTTGY_HNF3_Q6	659	0.06	1.91	0.009920635	0.04033614
GSE360_CTRL_VS_L_DONOVANI_DC_DN	124	0.15	1.91	0.01417004	0.040315803
GO_CELL_SUBSTRATE_ADHESION	142	0.14	1.91	0.00845666	0.040260475
MODULE_456	89	0.17	1.91	0.008163265	0.040266886
GSE44955_MCSF_VS_MCSF_AND_IL27_STIM_MACROPHAGE_UP	165	0.13	1.91	0.00610998	0.040226385
SASSON_RESPONSE_TO_GONADOTROPHINS_UP	82	0.18	1.91	0.005928854	0.04024543
GSE28237_FOLLICULAR_VS_LATE_GC_BCELL_DN	160	0.13	1.91	0.003968254	0.040254198
TORCHIA_TARGETS_OF_EWSR1_FLI1_FUSION_DN	267	0.10	1.91	0.00984252	0.040202126
GSE40068_CXCR5NEG_BCL6NEG_CD4_TCELL_VS_CXCR5POS_BCL6NEG_TFH_DN	160	0.13	1.91	0.001926782	0.040218905

GO_RUFFLE_ORGANIZATION	19	0.36	1.91	0.009560229	0.04002873
GSE17721_0.5H_VS_8H_GARDIQUIMOD_BMDC_UP	176	0.12	1.91	0.006036217	0.04001157
BIOCARTA_DEATH_PATHWAY	27	0.31	1.91	0.018255578	0.039985187
XU_AKT1_TARGETS_6HR	18	0.38	1.91	0.016260162	0.03996344
GSE26928_EFF_MEM_VS_CENTR_MEM_CD4_TCELL_UP	123	0.15	1.91	0.004140787	0.039858177
GSE24634_TEFF_VS_TCONV_DAY7_IN_CULTURE_DN	170	0.13	1.91	0.00203252	0.039840326
SP1_Q6_01	202	0.12	1.91	0.004040404	0.039825425
SENGUPTA_EBNA1_ANTICORRELATED	111	0.15	1.91	0.005825243	0.039836347
HOSHIDA_LIVER_CANCER_SURVIVAL_UP	60	0.21	1.92	<0.001	0.039789114
GO_MORPHOGENESIS_OF_AN_EPITHELIAL_SHEET	31	0.29	1.92	0.010141988	0.03970323
GSE25123_WT_VS_PPARG_KO_MACROPHAGE_DN	160	0.13	1.92	0.01192843	0.03946233
GRADE_COLON_CANCER_UP	697	0.07	1.92	0.011741683	0.039472524
VEGF_A_UP.V1_UP	143	0.14	1.92	0.007677543	0.039434243
GO_MYELOID_LEUKOCYTE_DIFFERENTIATION	69	0.20	1.92	0.003992016	0.03937691
GSE9650_EFFECTOR_VS_MEMORY_CD8_TCELL_UP	172	0.12	1.92	0.011904762	0.039352987
GO_CELLULAR_RESPONSE_TO_ENDOGENOUS_STIMULUS	754	0.06	1.92	0.007968128	0.039308347
NUTT_GBM_VS_AO_GLIOMA_UP	42	0.25	1.92	0.014	0.03932077
QI_PLASMACYTOMA_DN	86	0.18	1.92	0.006	0.039334383
GSE15330_WT_VS_IKAROS_KO_LYMPHOID_MULTIPOTENT_PROGENITOR_DN	169	0.13	1.92	0.005952381	0.039338123
PID_AP1_PATHWAY	55	0.22	1.92	0.01010101	0.03933209
DITTMER_PTHLH_TARGETS_DN	66	0.20	1.92	0.005791506	0.03905165
GO_1_PHOSPHATIDYLINOSITOL_BINDING	17	0.38	1.92	0.007874016	0.03906631
KEGG_PHOSPHATIDYLINOSITOL_SIGNALING_SYSTEM	68	0.20	1.92	0.00203252	0.03892381
GSE1460_CD4_THYMOCYTE_VS_NAIVE_CD4_TCELL_ADULT_BLOOD_UP	168	0.12	1.92	0.008064516	0.038868748
GO_TISSUE_MIGRATION	63	0.21	1.92	0.014256619	0.038761474
GSE37301_MULTIPOTENT_PROGENITOR_VS_GRAN_MONO_PROGENITOR_UP	150	0.14	1.92	0.008179959	0.03869587
GSE45365_CD8A_DC_VS_CD11B_DC_IFNAR_KO_MCMV_INFECTION_DN	78	0.18	1.92	0.012145749	0.038691837
GSE11961_GERMINAL_CENTER_BCELL_DAY7_VS_PLASMA_CELL_DAY7_DN	173	0.12	1.92	0.01775148	0.03852143
GSE1112_HY_CD8AB_VS_HY_CD8AA_THYMOCYTE_RTOC_CULTURE_DN	138	0.14	1.92	0.006024096	0.03844631
BIOCARTA_41BB_PATHWAY	13	0.44	1.92	0.011764706	0.038412996
GSE35685_CD34POS_CD38NEG_VS_CD34POS_CD10NEG_CD62LPOS_BONE_MA	152	0.14	1.92	0.001980198	0.038267832
GO_REGULATION_OF_LEUKOCYTE_MEDIATED_IMMUNITY	103	0.16	1.92	0.005928854	0.038122088
GSE2585_CTEC_VS_MTEC_THYMUS_UP	161	0.13	1.92	0.009652509	0.03803146
SHIN_B_CELL_LYMPHOMA_CLUSTER_8	31	0.29	1.93	0.015936255	0.038010776

GO_POSITIVE_REGULATION_OF_ALPHA_BETA_T_CELL_ACTIVATION	33	0.28	1.93	0.013916501	0.038009882
GSE43863_DAY6_EFF_VS_DAY150_MEM_TH1_CD4_TCELL_DN	165	0.13	1.93	0.005988024	0.03800025
GAVIN_FOXP3_TARGETS_CLUSTER_P4	79	0.18	1.93	0.00998004	0.037905354
HENDRICKS_SMARCA4_TARGETS_UP	45	0.24	1.93	0.004123712	0.03792308
REACTOME_NUCLEOTIDE_BINDING_DOMAIN_LEUCINE_RICH_REPEAT_CONTAIN	41	0.26	1.93	0.007827789	0.037817
GO_NEGATIVE_REGULATION_OF_OSTEOCLAST_DIFFERENTIATION	15	0.42	1.93	0.007707129	0.037661135
GO_RECEPTOR_METABOLIC_PROCESS	67	0.20	1.93	0.013806706	0.037630253
KIM_GERMINAL_CENTER_T_HELPER_DN	18	0.37	1.93	0.005825243	0.037620332
GO_NEGATIVE_REGULATION_OF_IMMUNE_RESPONSE	73	0.19	1.93	0.002012072	0.03752733
GSE21380_TFH_VS_GERMINAL_CENTER_TFH_CD4_TCELL_UP	140	0.14	1.93	0.007920792	0.037542574
GO_REGULATION_OF_ENDOTHELIAL_CELL_PROLIFERATION	82	0.18	1.93	0.017110266	0.03753855
GSE34156_UNTREATED_VS_6H_NOD2_LIGAND_TREATED_MONOCYTE_UP	150	0.14	1.93	0.00390625	0.03745708
GO_NEGATIVE_REGULATION_OF_CYTOKINE_PRODUCTION_INVOLVED_IN_IMMU	14	0.43	1.93	0.005928854	0.037448853
GSE22103_UNSTIM_VS_LPS_STIM_NEUTROPHIL_DN	160	0.13	1.93	0.003960396	0.037399568
GO_PROTEIN_N_LINKED_GLYCOSYLATION	65	0.20	1.93	0.003891051	0.037275597
GSE5679_PPARG_LIGAND_ROSIGLITAZONE_VS_ROSIGLITAZONE_AND_RARA_AG	161	0.13	1.93	0.003944773	0.03727845
GO_PLATELET_DERIVED_GROWTH_FACTOR_RECEPTOR_SIGNALING_PATHWAY	34	0.27	1.93	0.004048583	0.037195574
GSE13547_CTRL_VS_ANTI_IGM_STIM_ZFX_KO_BCELL_2H_UP	140	0.14	1.93	0.00390625	0.037211314
GO_REGULATION_OF_EPITHELIAL_CELL_DIFFERENTIATION	80	0.19	1.93	0.008196721	0.03719061
GO_RAC_GTPASE_BINDING	34	0.28	1.93	0.010162601	0.03714169
GSE17721_CTRL_VS_PAM3CSK4_6H_BMDC_DN	156	0.13	1.93	0.008350731	0.037155643
ZAMORA_NOS2_TARGETS_DN	83	0.18	1.93	0.003960396	0.037095446
GSE25088_ROSIGLITAZONE_VS_IL4_AND_ROSIGLITAZONE_STIM_STAT6_KO_MA	167	0.13	1.93	0.014141414	0.037060484
GO_ENDOTHELIAL_CELL_MIGRATION	47	0.24	1.93	0.00203666	0.036856826
GSE22886_TCELL_VS_BCELL_NAIVE_UP	165	0.13	1.93	0.003976143	0.036692575
ZHANG_TLX_TARGETS_DN	82	0.18	1.93	0.008639309	0.03665199
PRC2_EED_UP.V1_DN	168	0.13	1.93	0.005952381	0.03665632
GSE13738_TCR_VS_BYSTANDER_ACTIVATED_CD4_TCELL_DN	150	0.13	1.93	0.009208103	0.0365932
GSE1432_CTRL_VS_IFNG_1H_MICROGLIA_UP	143	0.14	1.93	0.01417004	0.036601644
GSE6875_WT_VS_FOXP3_KO_TREG_UP	165	0.13	1.93	0.00621118	0.03655513
GO_REGULATION_OF_SMOOTH_MUSCLE_CELL_PROLIFERATION	85	0.18	1.93	0.005988024	0.03652214
KAMMINGA_EZH2_TARGETS	37	0.27	1.93	0.007952286	0.036475446
MULLIGHAN_MLL_SIGNATURE_2_UP	336	0.09	1.94	0.008385744	0.036477346
GSE3982_NEUTROPHIL_VS_TH1_DN	177	0.12	1.94	0.008048289	0.036301494

GSE46606_UNSTIM_VS_CD40L_IL2_IL5_1DAY_STIMULATED_IRF4_KO_BCELL_UF	175	0.13	1.94	0.003944773	0.03631218
ZHU_CMV_8_HR_UP	34	0.28	1.94	0.003976143	0.03626717
GSE18791_UNSTIM_VS_NEWCATSLE_VIRUS_DC_18H_DN	113	0.16	1.94	0.011952192	0.036263023
GSE40274_IRF4_VS_FOXP3_AND_IRF4_TRANSDUCED_ACTIVATED_CD4_TCELL_U	158	0.13	1.94	0.007782101	0.036241677
GSE19772_HCMV_INFL_VS_HCMV_INF_MONOCYTES_AND_PI3K_INHIBITION_U	131	0.14	1.94	0.010060363	0.03624938
GO_EXTRINSIC_COMPONENT_OF_CYTOPLASMIC_SIDE_OF_PLASMA_MEMBRAN	71	0.20	1.94	0.005836576	0.036109686
GNF2_SPI1	25	0.32	1.94	0.005964215	0.035956115
GSE21546_WT_VS_SAP1A_KO_AND_ELK1_KO_DP_THYMOCYTES_UP	162	0.13	1.94	0.014842301	0.03581378
GO_NEGATIVE_REGULATION_OF_B_CELL_APOPTOTIC_PROCESS	10	0.50	1.94	0.005905512	0.03578798
KOKKINAKIS_METHIONINE_DEPRIVATION_96HR_UP	103	0.16	1.94	0.010183299	0.035623863
PID_INTEGRIN_A9B1_PATHWAY	22	0.34	1.94	0.00390625	0.035572805
GSE28737_WT_VS_BCL6_KO_FOLLICULAR_BCELL_DN	172	0.13	1.94	0.00210084	0.03550691
GSE360_CTRL_VS_B_MALAYI_LOW_DOSE_MAC_DN	158	0.13	1.94	0.013833992	0.035505913
GO_REGULATION_OF_STRESS_ACTIVATED_PROTEIN_KINASE_SIGNALING_CASCA	163	0.13	1.94	0.001934236	0.035513896
GSE1432_1H_VS_6H_IFNG_MICROGLIA_DN	166	0.13	1.94	0.005780347	0.03538819
HALLMARK_HEDGEHOG_SIGNALING	30	0.30	1.94	0.01004016	0.03534358
GO_CELL_PROLIFERATION	492	0.08	1.94	0.009784736	0.03528945
GSE11961_MEMORY_BCELL_DAY7_VS_GERMINAL_CENTER_BCELL_DAY7_UP	157	0.13	1.94	0.011904762	0.03521138
GO_NEGATIVE_REGULATION_OF_MULTI_ORGANISM_PROCESS	97	0.17	1.94	0.00409836	0.03520669
GO_POSITIVE_REGULATION_OF_CELL_MORPHOGENESIS_INVOLVED_IN_DIFFERE	141	0.14	1.94	0.012195122	0.03512593
GO_CYTOSOLIC_LARGE_RIBOSOMAL_SUBUNIT	31	0.29	1.94	0.00189394	0.035089977
GO_REGULATION_OF_BINDING	232	0.11	1.94	0.006036217	0.034925427
GO_HOMOTYPIC_CELL_CELL_ADHESION	45	0.24	1.95	0.001865672	0.034771334
REACTOME_N_GLYCAN_ANTENNAE_ELONGATION_IN_THE_MEDIAL_TRANS_GC	15	0.42	1.95	0.00408998	0.034672245
CAMPS_COLON_CANCER_COPY_NUMBER_UP	52	0.23	1.95	0.009803922	0.03467684
GO_LEUKOCYTE_DIFFERENTIATION	211	0.12	1.95	0.013806706	0.034594454
TAKEDA_TARGETS_OF_NUP98_HOXA9_FUSION_3D_DN	22	0.35	1.95	0.007858546	0.034463264
GSE10325_BCELL_VS_LUPUS_BCELL_DN	158	0.13	1.95	0.012345679	0.034480385
GO_CYTOSOLIC_SMALL_RIBOSOMAL_SUBUNIT	30	0.31	1.95	0.004	0.03447651
GGAMTNNNNNTCCY_UNKNOWN	22	0.35	1.95	0.001956947	0.034483444
REACTOME_GASTRIN_CREB_SIGNALLING_PATHWAY_VIA_PKC_AND_MAPK	109	0.16	1.95	0.002053388	0.0344561
GSE360_T_GONDII_VS_B_MALAYI_HIGH_DOSE_MAC_DN	158	0.13	1.95	0.004246285	0.034271177
GSE369_PRE_VS_POST_IL6_INJECTION_IFNG_WT_LIVER_UP	124	0.15	1.95	0.008213553	0.034281034
KEGG_P53_SIGNALING_PATHWAY	57	0.22	1.95	0.006012024	0.0342891

GO_REGULATION_OF_TUMOR_NECROSIS_FACTOR_BIOSYNTHETIC_PROCESS	12	0.46	1.95	0.006072875	0.034288697
GSE23114_WT_VS_SLE2C1_MOUSE_SPLEEN_B1A_BCELL_UP	178	0.13	1.95	0.008350731	0.034254603
MURAKAMI_UV_RESPONSE_6HR_UP	30	0.30	1.95	0.013916501	0.034258407
GSE3982_DC_VS_NEUTROPHIL_DN	142	0.14	1.95	0.006134969	0.03396403
JAATINEN_HEMATOPOIETIC_STEM_CELL_DN	142	0.14	1.95	0.009727626	0.033861395
GSE40274_CTRL_VS_IRF4_TRANSDUCED_ACTIVATED_CD4_TCELL_UP	143	0.14	1.95	0.001960784	0.033729725
GO_DEFENSE_RESPONSE_TO_BACTERIUM	99	0.17	1.95	0.012422361	0.03373204
GO_RESPONSE_TO_AMMONIUM_ION	33	0.28	1.95	0.006160164	0.033669703
GO_NEGATIVE_REGULATION_OF_RESPONSE_TO_STIMULUS	1025	0.06	1.95	0.001964637	0.03366109
BIOCARTA_VITCB_PATHWAY	10	0.52	1.95	0.007561437	0.033616826
GSE29617_CTRL_VS_TIV_FLU_VACCINE_PBMC_2008_UP	139	0.14	1.95	0.006342495	0.033632457
GO_REGULATION_OF_NEUROBLAST_PROLIFERATION	20	0.37	1.95	0.012320329	0.033481956
GO_REGULATION_OF_MULTI_ORGANISM_PROCESS	328	0.09	1.95	0.00998004	0.033461086
MORI_LARGE_PRE_BII_LYMPHOCYTE_DN	52	0.23	1.95	0.004032258	0.033449948
BIOCARTA_TOLL_PATHWAY	34	0.28	1.95	0.01039501	0.03339208
GO_REGULATION_OF_NEURAL_PRECURSOR_CELL_PROLIFERATION	50	0.23	1.95	0.011811024	0.033350505
WOO_LIVER_CANCER_RECURRENCE_UP	88	0.18	1.95	0.005769231	0.03333082
TAKEDA_TARGETS_OF_NUP98_HOXA9_FUSION_16D_UP	109	0.16	1.96	0.004032258	0.03331654
GO_PROTEIN_PHOSPHATASE_TYPE_1_COMPLEX	10	0.52	1.96	0.001937985	0.033259656
GSE3982_MAST_CELL_VS_MAC_DN	162	0.13	1.96	0.007782101	0.03326483
GO_MULTI_ORGANISM_METABOLIC_PROCESS	99	0.17	1.96	0.011741683	0.03326214
GO_VASCULAR_ENDOTHELIAL_GROWTH_FACTOR_SIGNALING_PATHWAY	14	0.44	1.96	0.006097561	0.03327098
GSE17974_1.5H_VS_72H_IL4_AND_ANTI_IL12_ACT_CD4_TCELL_UP	165	0.14	1.96	0.005882353	0.03291351
KRIEG_HYPOXIA_VIA_KDM3A	41	0.25	1.96	<0.001	0.032747686
GSE6674_ANTI_IGM_VS_PL2_3_STIM_BCELL_DN	188	0.12	1.96	0.00862069	0.032750797
GO_NEGATIVE_REGULATION_OF_SIGNAL_TRANSDUCTION_IN_ABSENCE_OF_LIG	22	0.35	1.96	0.006289308	0.03273439
GSE21360_NAIVE_VS_PRIMARY_MEMORY_CD8_TCELL_DN	162	0.13	1.96	0.001996008	0.03268868
GO_PLATELET_DERIVED_GROWTH_FACTOR_BINDING	10	0.51	1.96	0.006237006	0.032583717
REACTOME_G_ALPHA1213_SIGNALLING_EVENTS	62	0.22	1.96	0.009940358	0.032444414
GSE14415_TCONV_VS_FOXP3_KO_INDUCED_TREG_DN	155	0.14	1.96	0.006134969	0.03234344
GSE33424_CD161_HIGH_VS_INT_CD8_TCELL_DN	159	0.13	1.96	0.010141988	0.032327402
GO_POSITIVE_REGULATION_OF_ENDOTHELIAL_CELL_MIGRATION	63	0.21	1.96	0.006134969	0.032300893
REACTOME_CHONDROITIN_SULFATE_DERMATAN_SULFATE_METABOLISM	38	0.27	1.96	0.007858546	0.03230379
PID_NFKAPPAB_CANONICAL_PATHWAY	22	0.35	1.96	0.004115226	0.03215338

REACTOME_NFKB_ACTIVATION_THROUGH_FADD_RIP1_PATHWAY_MEDIATED_	11	0.49	1.96	0.008016032	0.03216968
GO_ENZYME_ACTIVATOR_ACTIVITY	385	0.09	1.96	0.003992016	0.03214924
GO_NEGATIVE_REGULATION_OF_VASCULATURE_DEVELOPMENT	61	0.22	1.96	<0.001	0.032154515
GO_POSITIVE_REGULATION_OF_VACUOLE_ORGANIZATION	10	0.50	1.96	0.012219959	0.032105174
GSE39820_TGFBETA1_VS_TGFBETA3_IN_IL6_IL23A_TREATED_CD4_TCELL_DN	147	0.14	1.96	0.005964215	0.032045957
MARSON_FOXP3_TARGETS_UP	58	0.22	1.96	0.008281574	0.032029632
GSE41867_DAY15_EFFECTOR_VS_DAY30_MEMORY_CD8_TCELL_LCMV_ARMSTF	145	0.14	1.96	0.004056795	0.032037932
GSE22886_NAIVE_TCELL_VS_MONOCYTE_DN	174	0.13	1.96	0.013333334	0.032046225
MODULE_532	310	0.10	1.96	0.009881423	0.031955447
GO_REGULATION_OF_VIRAL_GENOME_REPLICATION	57	0.22	1.96	0.013435701	0.031842344
GO_REGULATION_OF_CHEMOKINE_PRODUCTION	47	0.24	1.97	0.010141988	0.03178386
GO_POSITIVE_REGULATION_OF_INTERLEUKIN_1_PRODUCTION	21	0.36	1.97	0.007889546	0.03159292
GSE2128_C57BL6_VS_NOD_THYMOCYTE_UP	174	0.13	1.97	0.003816794	0.031603497
GSE34156_UNTREATED_VS_24H_NOD2_LIGAND_TREATED_MONOCYTE_DN	160	0.13	1.97	0.004264392	0.031584416
GNF2_BNIP2	32	0.30	1.97	0.007766991	0.03159562
GSE14000_4H_VS_16H_LPS_DC_TRANSLATED_RNA_UP	163	0.13	1.97	0.005639098	0.031580213
GSE15139_GMCSF_TREATED_VS_UNTREATED_NEUTROPHILS_DN	182	0.12	1.97	0.005976096	0.031556193
GO_ENZYME_REGULATOR_ACTIVITY	703	0.07	1.97	0.003984064	0.031527143
JECHLINGER_EPITHELIAL_TO_MESENCHYMAL_TRANSITION_DN	57	0.22	1.97	0.001980198	0.031524114
GO_CORTICAL_CYTOSKELETON_ORGANIZATION	32	0.30	1.97	0.003853565	0.031528503
GSE29164_DAY3_VS_DAY7_UNTREATED_MELANOMA_DN	181	0.13	1.97	0.004132231	0.031474207
GO_ENZYME_BINDING	1448	0.05	1.97	0.003853565	0.031245481
YAATNANRNNNCAG_UNKNOWN	99	0.17	1.97	0.006085193	0.031177204
FERREIRA_EWINGS_SARCOMA_UNSTABLE_VS_STABLE_UP	141	0.14	1.97	0.010822511	0.031182641
GO_KINESIN_COMPLEX	46	0.24	1.97	0.002053388	0.031163875
GSE22589_SIV_VS_HIV_AND_SIV_INFECTED_DC_UP	131	0.15	1.97	0.003898636	0.031148795
TURASHVILI_BREAST_DUCTAL_CARCINOMA_VS_LOBULAR_NORMAL_UP	62	0.21	1.97	<0.001	0.031144885
GSE40274_CTRL_VS_GATA1_TRANSDUCED_ACTIVATED_CD4_TCELL_DN	129	0.15	1.97	0.003853565	0.031123534
GSE37301_GRANULOCYTE_MONOCYTE_PROGENITOR_VS_RAG2_KO_NK_CELL_I	169	0.13	1.97	<0.001	0.031086095
GSE42021_TREG_PLN_VS_TREG_PRECURSORS_THYMUS_UP	152	0.14	1.97	0.00203252	0.031028066
GNF2_CKS1B	35	0.29	1.97	0.006012024	0.030976877
RAMALHO_STEMNESS_DN	58	0.21	1.97	0.009708738	0.030992836
GSE42021_CD24INT_VS_CD24LOW_TCONV_THYMUS_DN	143	0.14	1.97	0.004123712	0.030974548
NRF2_01	237	0.11	1.97	0.005905512	0.030826567

GO_CELLULAR_DEFENSE_RESPONSE	29	0.31	1.97	0.007889546	0.030821288
GO_LUNG_MORPHOGENESIS	32	0.30	1.97	0.007797271	0.030725382
GSE3720_UNSTIM_VS_LPS_STIM_VD2_GAMMADELTA_TCELL_UP	130	0.15	1.97	0.001996008	0.030669441
DASU_IL6_SIGNALING_SCAR_DN	15	0.41	1.97	0.001912046	0.03059601
CHIBA_RESPONSE_TO_TSA_DN	21	0.36	1.97	0.005940594	0.030591806
GO_ESTABLISHMENT_OF_PROTEIN_LOCALIZATION_TO_MEMBRANE	212	0.12	1.97	0.005703422	0.030593222
GO_PHOSPHATIDYLINOSITOL_3_4_5_TRISPHOSPHATE_BINDING	30	0.30	1.97	0.012345679	0.030498208
GSE22443_IL2_VS_IL12_TREATED_ACT_CD8_TCELL_DN	176	0.13	1.97	0.006122449	0.030503318
GSE43863_TH1_VS_TFH_MEMORY_CD4_TCELL_DN	174	0.13	1.97	<0.001	0.030507833
GSE3039_CD4_TCELL_VS_ALPHAALPHA_CD8_TCELL_UP	172	0.13	1.98	0.003992016	0.030468546
KEGG_GLYCOSPHINGOLIPID_BIOSYNTHESIS_GANGLIO_SERIES	13	0.45	1.98	0.008179959	0.030264158
GSE21379_WT_VS_SAP_KO_CD4_TCELL_UP	164	0.13	1.98	0.008163265	0.030268526
GSE45365_NK_CELL_VS_CD11B_DC_MCMV_INFECTION_DN	164	0.13	1.98	0.01509434	0.030252216
GO_NEGATIVE_REGULATION_OF_CELL_DEATH	644	0.07	1.98	0.006	0.030254181
GO_CELLULAR_RESPONSE_TO_INTERLEUKIN_1	59	0.22	1.98	0.003976143	0.030241543
GO_RESPONSE_TO_ACETYLCHOLINE	12	0.46	1.98	0.011695907	0.030244723
RUTELLA_RESPONSE_TO_HGF_VS_CSF2RB_AND_IL4_UP	329	0.09	1.98	0.00996016	0.030199425
ESC_J1_UP_LATE.V1_UP	140	0.14	1.98	0.003921569	0.030147078
CHIARADONNA_NEOPLASTIC_TRANSFORMATION_KRAS_DN	132	0.15	1.98	<0.001	0.030092303
YAN_ESCAPE_FROM_ANOIKIS	18	0.40	1.98	0.008213553	0.030069582
GSE40666_WT_VS_STAT1_KO_CD8_TCELL_WITH_IFNA_STIM_90MIN_DN	165	0.13	1.98	<0.001	0.029983057
ROSS_AML_WITH_CBFB_MYH11_FUSION	40	0.26	1.98	0.00589391	0.029861836
GSE6259_FLT3L_INDUCED_DEC205_POS_DC_VS_BCELL_DN	135	0.15	1.98	0.009940358	0.029874252
GSE2128_C57BL6_VS_NOD_CD4CD8_DP_THYMOCYTE_UP	160	0.13	1.98	0.004132231	0.029848736
GSE9988_ANTI_TREM1_VS_LOW_LPS_MONOCYTE_UP	162	0.14	1.98	0.010504202	0.02970448
MODULE_75	243	0.11	1.98	0.003875969	0.02969598
AMUNDSON_GENOTOXIC_SIGNATURE	89	0.18	1.98	0.004040404	0.02967231
MORF_NPM1	124	0.15	1.98	0.001869159	0.029658709
GARGALOVIC_RESPONSE_TO_OXIDIZED_PHOSPHOLIPIDS_MAGENTA_UP	23	0.34	1.98	0.003984064	0.02965213
SEITZ_NEOPLASTIC_TRANSFORMATION_BY_8P_DELETION_UP	53	0.23	1.98	0.006024096	0.029642308
GSE46606_IRF4_KO_VS_WT_UNSTIM_BCELL_DN	143	0.14	1.98	0.005725191	0.02953999
HOFFMANN_PRE_BI_TO_LARGE_PRE_BII_LYMPHOCYTE_DN	60	0.21	1.98	0.004	0.029551039
GSE17721_CTRL_VS_GARDIQUIMOD_8H_BMDC_DN	170	0.13	1.98	0.003960396	0.029494207
GO_RESPONSE_TO_LAMINAR_FLUID_SHEAR_STRESS	14	0.45	1.98	0.005905512	0.029439861

GO_POSITIVE_REGULATION_OF_CHEMOTAXIS	91	0.18	1.98	0.004106776	0.029344175
GSE3337_CTRL_VS_16H_IFNG_IN_CD8POS_DC_UP	167	0.13	1.98	0.002070393	0.029245736
GO_MYELOID_CELL_DIFFERENTIATION	144	0.14	1.98	0.00204918	0.029247744
LEE_NAIVE_T_LYMPHOCYTE	11	0.50	1.98	0.008032128	0.029224336
GSE7852_TREG_VS_TCONV_UP	167	0.13	1.99	0.00390625	0.029173503
GGGACCA_MIR133A_MIR133B	143	0.14	1.99	<0.001	0.02913034
GO_ENDOLYSOSOME_MEMBRANE	11	0.49	1.99	0.00845666	0.02903361
BILANGES_SERUM_AND_RAPAMYCIN_SENSITIVE_GENES	50	0.24	1.99	0.002008032	0.029031731
GO_EPITHELIAL_CELL_MORPHOGENESIS	29	0.31	1.99	0.001919386	0.029036202
GO_CELLULAR_RESPONSE_TO_GLUCOSE_STARVATION	29	0.31	1.99	0.005905512	0.029048972
GO_RETROGRADE_VESICLE_MEDIATED_TRANSPORT_GOLGI_TO_ER	70	0.20	1.99	0.001960784	0.029062396
KRIGE_RESPONSE_TO_TOSEDOSTAT_24HR_DN	844	0.06	1.99	0.002070393	0.029004317
GO_CYTOKINETIC_PROCESS	13	0.45	1.99	0.003731343	0.028994748
GSE4535_BM_DERIVED_DC_VS_FOLLICULAR_DC_UP	160	0.13	1.99	0.007843138	0.02899599
GO_PHOSPHATIDYLINOSITOL_PHOSPHATE_BINDING	99	0.17	1.99	0.007889546	0.029010069
GSE27786_NKTCELL_VS_NEUTROPHIL_DN	154	0.14	1.99	0.008032128	0.029006243
GSE3920_IFNA_VS_IFNG_TREATED_FIBROBLAST_DN	165	0.13	1.99	0.002053388	0.028905733
GRAESSMANN_APOPTOSIS_BY_SERUM_DEPRIVATION_UP	430	0.08	1.99	0.001984127	0.0288967
GSE26030_UNSTIM_VS_RESTIM_TH17_DAY5_POST_POLARIZATION_UP	154	0.14	1.99	0.00811359	0.028890874
REACTOME_SEMA3A_PLEXIN_REPULSION_SIGNALING_BY_INHIBITING_INTEGRI	13	0.46	1.99	0.005494506	0.028892057
CAIRO_PML_TARGETS_BOUND_BY_MYC_UP	21	0.36	1.99	0.001968504	0.028717272
CHIN_BREAST_CANCER_COPY_NUMBER_UP	21	0.36	1.99	0.011673152	0.028706877
GNF2_TNFSF10	23	0.35	1.99	0.008048289	0.028687524
MODULE_459	329	0.10	1.99	0.008097166	0.0286867
GSE13738_RESTING_VS_BYSTANDER_ACTIVATED_CD4_TCELL_DN	168	0.13	1.99	0.002057613	0.028662069
GO_AMINOGLYCAN_METABOLIC_PROCESS	118	0.16	1.99	0.004016064	0.028661883
CRX_NRL_DN.V1_UP	90	0.18	1.99	0.006072875	0.028574811
GO_EXTRINSIC_COMPONENT_OF_PLASMA_MEMBRANE	100	0.17	1.99	<0.001	0.028545534
GO_BASAL_PLASMA_MEMBRANE	21	0.36	1.99	0.00984252	0.0285498
THUM_SYSTOLIC_HEART_FAILURE_UP	350	0.09	1.99	0.004048583	0.028558556
GSE43955_1H_VS_20H_ACT_CD4_TCELL_WITH_TGFB_IL6_DN	151	0.14	1.99	0.00998004	0.028570566
GCGCCTT_MIR525_MIR524	13	0.45	1.99	0.00210084	0.028510256
GO_REGULATION_OF_INTERLEUKIN_1_SECRETION	19	0.38	1.99	0.007462686	0.028311541
GSE19888_NO_PRETREAT_VS_ADENOSINE_A3R_INHIBITOR_PRETREATED_MAST	183	0.13	1.99	0.005964215	0.028285332

GSE43955_10H_VS_60H_ACT_CD4_TCELL_WITH_TGFB_IL6_DN	160	0.14	1.99	0.006185567	0.028271325
GSE4590_LARGE_PRE_BCELL_VS_VPREB_POS_LARGE_PRE_BCELL_DN	160	0.13	1.99	0.006036217	0.028268352
GO_POSITIVE_REGULATION_OF_DEFENSE_RESPONSE	265	0.11	1.99	0.001980198	0.028258871
COATES_MACROPHAGE_M1_VS_M2_DN	60	0.21	1.99	0.005825243	0.028258443
LOPEZ_MESOTELIOMA_SURVIVAL_TIME_UP	11	0.49	1.99	0.002012072	0.028248955
SATO_SILENCED_BY_METHYLATION_IN_PANCREATIC_CANCER_1	251	0.11	1.99	0.007889546	0.028199919
KRAS.AMP.LUNG_UP.V1_DN	69	0.20	1.99	0.006048387	0.028207913
GO_POSITIVE_REGULATION_OF_B_CELL_DIFFERENTIATION	12	0.48	1.99	0.006160164	0.028213343
GO_FILOPODIUM_ASSEMBLY	15	0.43	1.99	0.005769231	0.028220069
GO_RESPONSE_TO_MURAMYL_DIPEPTIDE	15	0.43	1.99	0.008421052	0.028152142
ESC_J1_UP_EARLY.V1_DN	139	0.14	1.99	0.00998004	0.028069234
GO_REGULATION_OF_TUMOR_NECROSIS_FACTOR_MEDIATED_SIGNALING_PAT	40	0.26	2.00	0.004081633	0.028025705
GO_MICROTUBULE	320	0.10	2.00	0.004032258	0.027901525
PID_IL2_PI3K_PATHWAY	31	0.30	2.00	0.005952381	0.027913952
PID_NECTIN_PATHWAY	28	0.31	2.00	0.003960396	0.027927682
GSE10273_HIGH_IL7_VS_HIGH_IL7_AND_IRF4_IN_IRF4_8_NULL_PRE_BCELL_DN	168	0.13	2.00	0.003913894	0.027925767
GO_LEUKOCYTE_CELL_CELL_ADHESION	178	0.13	2.00	0.002074689	0.02788927
GSE26928_NAIVE_VS_CXCR5_POS_CD4_TCELL_DN	152	0.14	2.00	0.003952569	0.027895818
GSE42088_UNINF_VS_LEISHMANIA_INF_DC_24H_UP	181	0.13	2.00	0.002061856	0.027849447
SASSON_RESPONSE_TO_GONADOTROPHINS_DN	76	0.20	2.00	0.001937985	0.027845506
GO_REGULATION_OF_INTERLEUKIN_1_BETA_PRODUCTION	28	0.32	2.00	0.002008032	0.02782001
GSE46606_IRF4HIGH_VS_WT_CD40L_IL2_IL5_DAY1_STIMULATED_BCELL_DN	161	0.14	2.00	0.008	0.027740179
GSE46242_TH1_VS_ANERGIC_TH1_CD4_TCELL_UP	142	0.14	2.00	0.001984127	0.0276911
GO_CELL_ACTIVATION_INVOLVED_IN_IMMUNE_RESPONSE	94	0.18	2.00	0.008097166	0.027642502
LINDSTEDT_DENDRITIC_CELL_MATURATION_A	48	0.24	2.00	0.001941748	0.027631242
GSE13484_12H_UNSTIM_VS_YF17D_VACCINE_STIM_PBMC_DN	154	0.14	2.00	0.005940594	0.02752687
WUNDER_INFLAMMATORY_RESPONSE_AND_CHOLESTEROL_UP	40	0.26	2.00	<0.001	0.027529374
GO_POSITIVE_REGULATION_OF_EPITHELIAL_CELL_MIGRATION	93	0.18	2.00	0.004048583	0.027469581
GSE13522_CTRL_VS_T_CRUZI_Y_STRAIN_INF_SKIN_IFNAR_KO_UP	154	0.14	2.00	0.008298756	0.027448457
VANTVEER_BREAST_CANCER_ESR1_DN	183	0.12	2.00	0.005859375	0.027429208
GSE46242_CTRL_VS_EGR2_DELETED_TH1_CD4_TCELL_UP	146	0.14	2.00	0.008032128	0.027398808
GO_INTERLEUKIN_1_MEDIATED_SIGNALING_PATHWAY	13	0.46	2.00	0.001964637	0.027109977
GO_REGULATION_OF_MACROPHAGE_CHEMOTAXIS	12	0.48	2.00	0.003861004	0.027091872
GSE45365_HEALTHY_VS_MCMV_INFECTION_CD8A_DC_DN	161	0.14	2.00	0.006147541	0.027042132

GSE24634_TREG_VS_TCONV_POST_DAY7_IL4_CONVERSION_UP	159	0.14	2.00	0.00203666	0.027046343
GSE12366_GC_BCELL_VS_PLASMA_CELL_UP	165	0.14	2.00	0.006060606	0.027047224
PID_VEGFR1_2_PATHWAY	67	0.21	2.00	0.002024292	0.027029598
GSE5589_IL6_KO_VS_IL10_KO_LPS_AND_IL10_STIM_MACROPHAGE_180MIN_U	181	0.13	2.00	0.004056795	0.027039096
GO_ESTABLISHMENT_OF_PROTEIN_LOCALIZATION_TO_ORGANELLE	281	0.10	2.00	0.004158004	0.026973955
GNF2_ICAM3	33	0.29	2.01	0.00811359	0.026860505
GO_REGULATION_OF_ALPHA_BETA_T_CELL_ACTIVATION	43	0.26	2.01	0.004016064	0.026842162
GO_REGULATION_OF_T_HELPER_1_TYPE_IMMUNE_RESPONSE	14	0.44	2.01	0.001937985	0.026814481
MODULE_27	215	0.12	2.01	0.007462686	0.026671084
VERRECCHIA_DELAYED_RESPONSE_TO_TGFB1	36	0.28	2.01	0.002083333	0.026638003
GSE3039_CD4_TCELL_VS_B2_BCELL_DN	177	0.13	2.01	0.004115226	0.026584458
GO_POSITIVE_REGULATION_OF_CELL_CELL_ADHESION	160	0.14	2.01	0.002132196	0.02651022
GSE40685_NAIVE_CD4_TCELL_VS_FOXP3_KO_TREG_PRECURSOR_UP	151	0.14	2.01	0.00407332	0.026501568
GO_REGULATION_OF_ENDOTHELIAL_CELL_DIFFERENTIATION	24	0.34	2.01	0.003861004	0.02646117
E2F3_UP.V1_DN	80	0.20	2.01	0.004024145	0.026429893
MUNSHI_MULTIPLE_MYELOMA_UP	65	0.21	2.01	0.005988024	0.026426462
PID_PLK1_PATHWAY	42	0.27	2.01	0.003861004	0.026310077
HINATA_NFKB_TARGETS_FIBROBLAST_UP	66	0.21	2.01	0.004048583	0.026276719
GO_REGULATION_OF_CELL_MATRIX_ADHESION	74	0.20	2.01	0.005847953	0.026273906
SENESE_HDAC1_TARGETS_UP	373	0.09	2.01	0.00591716	0.026165947
GO_REGULATION_OF_LIPOPOLYSACCHARIDE_MEDIATED_SIGNALING_PATHWAY	16	0.42	2.01	0.003968254	0.026115144
GSE6092_CTRL_VS_BORRELIA_BIRGDOFERI_INF_ENDOTHELIAL_CELL_DN	105	0.17	2.01	0.005714286	0.026072785
DELACROIX_RARG_BOUND_MEF	307	0.10	2.01	0.009765625	0.026056457
GO_POSITIVE_REGULATION_OF_CELL_DEVELOPMENT	357	0.09	2.01	0.002155172	0.026049485
GSE45365_WT_VS_IFNAR_KO_BCELL_MCMV_INFECTION_DN	117	0.16	2.01	0.014344262	0.02602243
GO_POSITIVE_REGULATION_OF_MACROPHAGE_CHEMOTAXIS	10	0.53	2.01	0.002070393	0.026013399
REACTOME_G_BETA_GAMMA_SIGNALLING_THROUGH_PI3KGAMMA	19	0.38	2.01	0.006122449	0.026017742
GSE29614_CTRL_VS_DAY7_TIV_FLU_VACCINE_PBMC_DN	152	0.14	2.01	0.004040404	0.02600403
GSE17721_LPS_VS_PAM3CSK4_2H_BMDC_UP	164	0.14	2.01	<0.001	0.02600839
FOURNIER_ACINAR_DEVELOPMENT_LATE_2	254	0.11	2.01	0.008281574	0.026007392
KAUFFMANN_MELANOMA_RELAPSE_UP	57	0.22	2.01	0.001949318	0.02600439
GSE29164_UNTREATED_VS_CD8_TCELL_TREATED_MELANOMA_DAY7_UP	177	0.13	2.01	0.008230452	0.025917593
GO_FATTY_ACID_DERIVATIVE_BIOSYNTHETIC_PROCESS	29	0.31	2.01	0.004	0.025924576
GO_REGULATION_OF_CELL_PROJECTION_ASSEMBLY	124	0.15	2.01	0.007604563	0.025886063

REACTOME_G_PROTEIN_BETA_GAMMA_SIGNALLING	22	0.36	2.02	0.006289308	0.025709255
GSE17186_NAIVE_VS_CD21LOW_TRANSITIONAL_BCELL_CORD_BLOOD_DN	178	0.13	2.02	0.00408998	0.025676562
PID_IL4_2PATHWAY	47	0.24	2.02	0.002040816	0.025665304
REACTOME_RNA_POL_I_PROMOTER_OPENING	16	0.42	2.02	0.003710575	0.025638552
KRAS.600_UP.V1_UP	141	0.14	2.02	0.001972387	0.025565993
SIMBULAN_UV_RESPONSE_NORMAL_DN	31	0.31	2.02	0.006097561	0.025576176
FORTSCHEGGER_PHF8_TARGETS_DN	649	0.07	2.02	0.004056795	0.025489455
GO_REGULATION_OF_VASCULAR_ENDOTHELIAL_GROWTH_FACTOR_RECEPTOR	26	0.33	2.02	0.006085193	0.0254673
GO_LARGE_RIBOSOMAL_SUBUNIT	64	0.21	2.02	0.00610998	0.025476769
HALLMARK_HYPOXIA	173	0.13	2.02	0.002028398	0.02542096
GO_MITOTIC_SPINDLE_ORGANIZATION	63	0.22	2.02	0.001988072	0.025422327
GSE23502_WT_VS_HDC_KO_MYELOID_DERIVED_SUPPRESSOR_CELL_BM_DN	177	0.13	2.02	0.0056926	0.025432466
GSE3565_CTRL_VS_LPS_INJECTED_DUSP1_KO_SPLENOCYTES_UP	146	0.14	2.02	0.012244898	0.02537171
ALFANO_MYC_TARGETS	209	0.12	2.02	0.002057613	0.02536626
GSE3565_CTRL_VS_LPS_INJECTED_SPLENOCYTES_UP	147	0.15	2.02	<0.001	0.025318211
GSE21033_3H_VS_12H_POLYIC_STIM_DC_UP	131	0.15	2.02	0.007858546	0.025251178
GSE27786_CD8_TCELL_VS_NKTCELL_DN	167	0.14	2.02	0.005725191	0.025262624
GSE42088_2H_VS_24H_LEISHMANIA_INF_DC_UP	168	0.13	2.02	0.007968128	0.024967805
ISHIDA_E2F_TARGETS	50	0.25	2.02	<0.001	0.024966924
HELLER_HDAC_TARGETS_DN	245	0.11	2.02	0.003795066	0.024831671
GSE43955_10H_VS_30H_ACT_CD4_TCELL_UP	160	0.14	2.02	0.004032258	0.024802797
GSE30083_SP1_VS_SP4_THYMOCYTE_DN	167	0.14	2.02	0.007407407	0.024758307
GSE41867_DAY6_VS_DAY15_LCMV_ARMSTRONG_EFFECTOR_CD8_TCELL_UP	112	0.16	2.02	0.001934236	0.024771532
GSE15735_CTRL_VS_HDAC_INHIBITOR_TREATED_CD4_TCELL_2H_DN	155	0.14	2.02	0.004115226	0.024760336
GSE6674_ANTI_IGM_VS_ANTI_IGM_AND_CPG_STIM_BCELL_DN	179	0.13	2.02	0.004032258	0.02465052
GSE22886_DAY0_VS_DAY1_MONOCYTE_IN_CULTURE_DN	165	0.14	2.03	0.005725191	0.024555633
MODULE_44	249	0.11	2.03	<0.001	0.02455107
GSE29617_DAY3_VS_DAY7_TIV_FLU_VACCINE_PBMC_2008_UP	128	0.15	2.03	0.001964637	0.024538385
GSE24142_DN2_VS_DN3_THYMOCYTE_ADULT_UP	150	0.15	2.03	0.00998004	0.024530418
GSE26669_CTRL_VS_COSTIM_BLOCK_MLR_CD4_TCELL_UP	157	0.14	2.03	0.004024145	0.024530668
PID_P53_DOWNSTREAM_PATHWAY	113	0.16	2.03	<0.001	0.024509028
KAYO_CALORIE_RESTRICTION_MUSCLE_UP	76	0.20	2.03	0.006048387	0.024506468
GSE26030_TH1_VS_TH17_RESTIMULATED_DAY15_POST_POLARIZATION_UP	161	0.14	2.03	0.006085193	0.024511462
LI_WILMS_TUMOR_VS_FETAL_KIDNEY_1_DN	145	0.14	2.03	0.008097166	0.02447751

MISHRA_CARCINOMA_ASSOCIATED_FIBROBLAST_UP	15	0.44	2.03	0.001996008	0.024481088
GSE13411_PLASMA_CELL_VS_MEMORY_BCELL_UP	140	0.15	2.03	0.003875969	0.024419647
GSE16385_UNTREATED_VS_12H_ROSIGLITAZONE_IFNG_TNF_TREATED_MACRO	128	0.16	2.03	0.001968504	0.024410838
GSE369_PRE_VS_POST_IL6_INJECTION_IFNG_WT_LIVER_DN	171	0.14	2.03	0.00204499	0.024410278
GO_ORGANELLE_FISSION	388	0.09	2.03	0.006122449	0.024399497
DAZARD_RESPONSE_TO_UV_NHEK_UP	174	0.13	2.03	0.002057613	0.024389325
MITSIADES_RESPONSE_TO_APLIDIN_DN	230	0.12	2.03	0.010729614	0.024382615
GO_REGULATION_OF_NUCLEAR_DIVISION	131	0.15	2.03	0.004158004	0.024319516
GSE17721_LPS_VS_POLYIC_12H_BMDC_UP	175	0.13	2.03	0.010141988	0.02424744
IL2_UP.V1_UP	121	0.16	2.03	<0.001	0.024186254
GSE17721_0.5H_VS_4H_CPG_BMDC_DN	166	0.14	2.03	0.006160164	0.024071213
KEGG_APOPTOSIS	72	0.21	2.03	0.003861004	0.023872247
GSE21670_TGFB_VS_IL6_TREATED_STAT3_KO_CD4_TCELL_UP	160	0.14	2.03	0.002105263	0.02378407
GSE5503_LIVER_DC_VS_MLN_DC_ACTIVATED_ALLOGENIC_TCELL_DN	177	0.13	2.03	0.005791506	0.023719095
GNF2_PTPN6	37	0.28	2.03	0.002024292	0.0236499
GSE25088_IL4_VS_IL4_AND_ROSIGLITAZONE_STIM_MACROPHAGE_DAY10_DN	166	0.13	2.03	0.003992016	0.023619264
PID_TXA2PATHWAY	52	0.24	2.03	0.001976285	0.02358304
GSE46242_CTRL_VS_EGR2_DELETED_ANERGIC_TH1_CD4_TCELL_DN	150	0.14	2.03	0.01171875	0.02358064
GO_REGULATION_OF_NERVOUS_SYSTEM_DEVELOPMENT	534	0.08	2.04	<0.001	0.023508394
GSE17721_0.5H_VS_24H_CPG_BMDC_DN	173	0.13	2.04	0.002	0.023495551
GNF2_H2AFX	31	0.30	2.04	0.001930502	0.023421818
GSE17721_CPG_VS_GARDIQUIMOD_4H_BMDC_DN	178	0.13	2.04	0.002024292	0.023406826
GSE2706_R848_VS_R848_AND_LPS_2H_STIM_DC_DN	113	0.17	2.04	0.003968254	0.023404974
GSE10239_NAIVE_VS_KLRG1INT_EFF_CD8_TCELL_DN	162	0.13	2.04	0.016129032	0.023400353
GSE17721_0.5H_VS_8H_POLYIC_BMDC_DN	173	0.13	2.04	<0.001	0.023339678
GSE37563_WT_VS_CTLA4_KO_CD4_TCELL_D4_POST_IMMUNIZATION_DN	158	0.14	2.04	0.00422833	0.023197003
GO_MICROTUBULE_PLUS_END	16	0.43	2.04	0.007797271	0.023062367
FRASOR_RESPONSE_TO_SERM_OR_FULVESTRANT_DN	47	0.25	2.04	0.002028398	0.023058983
REACTOME_THROMBIN_SIGNALLING_THROUGH_PROTEINASE_ACTIVATED_REC	23	0.35	2.04	0.004065041	0.022839624
GSE24634_TEFF_VS_TCONV_DAY10_IN_CULTURE_UP	168	0.14	2.04	0.003898636	0.022827037
GO_REGULATION_OF_CELL_MORPHOGENESIS_INVOLVED_IN_DIFFERENTIATION	264	0.11	2.04	0.008179959	0.022771996
GO_NEGATIVE_REGULATION_OF_MYELOID_CELL_APOPTOTIC_PROCESS	11	0.50	2.04	0.00390625	0.022784414
GSE12198_CTRL_VS_HIGH_IL2_STIM_NK_CELL_DN	166	0.14	2.04	<0.001	0.022793356
BILD_MYC_ONCOGENIC_SIGNATURE	172	0.14	2.04	<0.001	0.022758402

GO_POSITIVE_REGULATION_OF_INNATE_IMMUNE_RESPONSE	201	0.13	2.04	0.001908397	0.022763154
GO_POSITIVE_REGULATION_OF_TRANSCRIPTION_FROM_RNA_POLYMERASE_II_	20	0.38	2.04	0.001912046	0.022696832
GSE46242_CTRL_VS_EGR2_DELETED_ANERGIC_TH1_CD4_TCELL_UP	169	0.14	2.04	0.004081633	0.022674955
GSE15930_STIM_VS_STIM_AND_IFNAB_48H_CD8_T_CELL_UP	183	0.13	2.04	0.003992016	0.02262042
LOPEZ_MESOTHELIOMA_SURVIVAL_WORST_VS_BEST_UP	12	0.49	2.04	<0.001	0.022609007
GSE37416_CTRL_VS_3H_F_TULARENSIS_LVS_NEUTROPHIL_DN	140	0.15	2.04	0.001934236	0.022575935
MODULE_12	292	0.11	2.04	0.003891051	0.022546306
GO_REGULATION_OF_CYTOKINE_SECRETION	92	0.19	2.04	0.006147541	0.022549512
PEDRIOLI_MIR31_TARGETS_DN	285	0.11	2.04	0.006147541	0.022554869
GSE22935_WT_VS_MYD88_KO_MACROPHAGE_12H_MBOVIS_BCG_STIM_DN	166	0.14	2.05	0.003968254	0.022457212
GSE5589_IL6_KO_VS_IL10_KO_LPS_AND_IL6_STIM_MACROPHAGE_45MIN_UP	180	0.13	2.05	0.007633588	0.022395331
GSE41867_NAIVE_VS_DAY15_LCMV_EFFECTOR_CD8_TCELL_DN	181	0.13	2.05	0.004024145	0.022219617
GO_FIBRIL_ORGANIZATION	16	0.41	2.05	0.005649718	0.02212162
HUANG_DASATINIB_RESISTANCE_UP	73	0.21	2.05	<0.001	0.022098627
MODULE_407	10	0.55	2.05	0.001980198	0.022065876
GSE17721_POLYIC_VS_PAM3CSK4_4H_BMDC_DN	168	0.14	2.05	0.006355932	0.022062544
GSE37533_UNTREATED_VS_PIOGLIZATONE_TREATED_CD4_TCELL_PPARG1_ANI	161	0.14	2.05	0.003944773	0.021891672
GSE7218_UNSTIM_VS_ANTIGEN_STIM_THROUGH_IGG_BCELL_DN	112	0.17	2.05	<0.001	0.02189595
GSE12845_IGD_NEG_BLOOD_VS_NAIVE_TONSIL_BCELL_DN	162	0.14	2.05	<0.001	0.02181843
GSE34156_UNTREATED_VS_6H_TLR1_TLR2_LIGAND_TREATED_MONOCYTE_DN	156	0.14	2.05	0.002109705	0.021821981
GSE2770_UNTREATED_VS_ACT_CD4_TCELL_6H_UP	159	0.14	2.05	0.004081633	0.021787427
GO_POSITIVE_REGULATION_OF_CELL_DEATH	485	0.08	2.05	0.002066116	0.021798734
GSE37532_WT_VS_PPARG_KO_VISCERAL_ADIPOSE_TISSUE_TREG_UP	163	0.14	2.05	0.006122449	0.021789616
GSE17721_CPG_VS_GARDIQUIMOD_2H_BMDC_UP	134	0.15	2.05	0.008130081	0.02178397
GSE2770_IL4_ACT_VS_ACT_CD4_TCELL_6H_DN	174	0.14	2.05	0.001960784	0.021731067
BIOCARTA_TNFR2_PATHWAY	16	0.42	2.05	0.001972387	0.021650335
GO_POSITIVE_REGULATION_OF_INTERLEUKIN_8_PRODUCTION	34	0.29	2.05	0.002066116	0.021612782
KEGG_MAPK_SIGNALING_PATHWAY	211	0.12	2.05	0.004081633	0.021510694
GSE32901_NAIVE_VS_TH17_NEG_CD4_TCELL_DN	74	0.21	2.05	0.001984127	0.021513399
LEONARD_HYPOXIA	43	0.26	2.06	<0.001	0.021317458
GO_LIPOPOLYSACCHARIDE_MEDIATED_SIGNALING_PATHWAY	29	0.32	2.06	0.003883495	0.021302238
GO_SPINDLE_LOCALIZATION	32	0.31	2.06	0.001988072	0.021186935
GSE13493_DP_VS_CD8POS_THYMOCYTE_DN	163	0.13	2.06	<0.001	0.021197256
PID_P38_MKK3_6PATHWAY	26	0.34	2.06	<0.001	0.021191226

GO_NEGATIVE_REGULATION_OF_CELL_DIFFERENTIATION	408	0.09	2.06	0.003976143	0.021188745
MORI_IMMATURE_B_LYMPHOCYTE_UP	48	0.25	2.06	<0.001	0.02107167
GO_ENDODERM_DEVELOPMENT	50	0.24	2.06	0.004106776	0.021027086
GSE24634_IL4_VS_CTRL_TREATED_NAIVE_CD4_TCELL_DAY5_UP	161	0.14	2.06	0.001972387	0.021022368
GSE21360_SECONDARY_VS_QUATERNARY_MEMORY_CD8_TCELL_DN	138	0.15	2.06	0.00209205	0.020993391
GSE2405_0H_VS_24H_A_PHAGOCYTOPHILUM_STIM_NEUTROPHIL_UP	163	0.14	2.06	0.005769231	0.020883108
GSE11057_CD4_CENT_MEM_VS_PBMC_DN	161	0.14	2.06	0.002008032	0.02086684
BAELDE_DIABETIC_NEPHROPATHY_UP	62	0.22	2.06	0.009689922	0.02076913
GO_POSITIVE_REGULATION_OF_SMOOTH_MUSCLE_CELL_PROLIFERATION	52	0.24	2.06	0.001976285	0.020743577
GSE39022_LN_VS_SPLEEN_DC_UP	156	0.14	2.06	<0.001	0.020747963
LEE_LIVER_CANCER_E2F1_UP	43	0.27	2.06	0.002004008	0.020740934
GSE43863_TFH_VS_LY6C_LOW_CXCR5NEG_EFFECTOR_CD4_TCELL_DN	133	0.15	2.06	0.00210084	0.020717435
GSE22935_UNSTIM_VS_12H_MBOVIS_BCG_STIM_MYD88_KO_MACROPHAGE_U	132	0.16	2.06	0.005769231	0.020726848
LABBE_TARGETS_OF_TGFB1_AND_WNT3A_UP	88	0.19	2.06	0.001968504	0.020692585
GO_REGULATION_OF_RESPONSE_TO_WOUNDING	281	0.10	2.06	0.003861004	0.020650381
MODULE_151	254	0.11	2.06	0.009505703	0.020646833
AMIT_EGF_RESPONSE_120_MCF10A	35	0.29	2.06	0.001897533	0.020654045
CHR14Q32	72	0.21	2.06	0.003992016	0.020574361
BUYTAERT_PHOTODYNAMIC_THERAPY_STRESS_DN	537	0.08	2.06	<0.001	0.020543355
GSE40274_CTRL_VS_FOXP3_AND_XBP1_TRANSDUCED_ACTIVATED_CD4_TCELL_	129	0.16	2.06	0.00204918	0.02051534
GSE2770_IL12_AND_TGFB_VS_IL4_TREATED_ACT_CD4_TCELL_48H_UP	169	0.14	2.06	0.003960396	0.020495832
GO_GOLGI_MEMBRANE	546	0.08	2.06	<0.001	0.020500114
TARTE_PLASMA_CELL_VS_B_LYMPHOCYTE_DN	32	0.30	2.07	0.001980198	0.020338615
NAKAMURA_ADIPOGENESIS_LATE_DN	33	0.30	2.07	0.004040404	0.020329144
KANG_AR_TARGETS_DN	14	0.45	2.07	0.003976143	0.020327576
MODULE_6	312	0.10	2.07	<0.001	0.020295698
CHEN_LVAD_SUPPORT_OF_FAILING_HEART_UP	90	0.19	2.07	0.003968254	0.020294812
SHETH_LIVER_CANCER_VS_TXNIP_LOSS_PAM3	48	0.25	2.07	<0.001	0.020180339
LEI_MYB_TARGETS	265	0.11	2.07	<0.001	0.020096952
BURTON_ADIPOGENESIS_9	83	0.19	2.07	0.003960396	0.020078579
GO_ACTIN_FILAMENT	63	0.23	2.07	0.004149378	0.020074666
GSE10211_UV_INACT_SENDAI_VS_LIVE_SENDAI_VIRUS_TRACHEAL_EPITHELIAL_	133	0.16	2.07	0.002	0.020041829
GO_NEGATIVE_REGULATION_OF_RESPONSE_TO_EXTERNAL_STIMULUS	191	0.13	2.07	<0.001	0.019945798
GSE22611_UNSTIM_VS_6H_MDP_STIM_NOD2_TRANSDUCED_HEK293T_CELL_U	130	0.16	2.07	<0.001	0.019942509

SHEPARD_BMYB_MORPHOLINO_DN	144	0.15	2.07	<0.001	0.019953022
GSE37533_UNTREATED_VS_PIOGLIZATONE_TREATED_CD4_TCELL_FOXP3_TRAS	122	0.16	2.07	0.002004008	0.019960653
AMIT_EGF_RESPONSE_40_HELA	40	0.28	2.07	0.001988072	0.019950096
MODULE_312	39	0.28	2.07	0.001945525	0.019959176
GSE36826_WT_VS_IL1R_KO_SKIN_STAPH_AUREUS_INF_UP	180	0.13	2.07	0.001960784	0.019948637
GO_INTERSPECIES_INTERACTION_BETWEEN_ORGANISMS	524	0.08	2.07	0.008	0.019897304
GSE27859_MACROPHAGE_VS_CD11C_INT_F480_HI_MACROPHAGE_UP	149	0.15	2.07	<0.001	0.019906374
GO_POSITIVE_REGULATION_OF_CELL_ACTIVATION	204	0.13	2.07	0.001945525	0.019762274
GSE19401_UNSTIM_VS_RETINOIC_ACID_STIM_FOLLICULAR_DC_UP	162	0.14	2.07	0.005607477	0.019772723
GO_REGULATION_OF_CYTOKINE_PRODUCTION_INVOLVED_IN_IMMUNE_RESPC	42	0.27	2.07	0.005660377	0.01975834
ACOSTA_PROLIFERATION_INDEPENDENT_MYC_TARGETS_DN	96	0.18	2.07	0.003868472	0.019760054
GO_CELLULAR_RESPONSE_TO_EXOGENOUS_DSRNA	10	0.55	2.07	0.005681818	0.01975011
GSE17721_PAM3CSK4_VS_CPG_16H_BMDC_DN	157	0.14	2.07	0.002096436	0.019760586
GO_GOLGI_APPARATUS	1096	0.06	2.07	0.002016129	0.01976961
VARELA_ZMPSTE24_TARGETS_UP	36	0.29	2.07	0.001949318	0.019730993
NADERI_BREAST_CANCER_PROGNOSIS_UP	31	0.31	2.07	0.003984064	0.019727532
GSE26928_NAIVE_VS_EFF_MEMORY_CD4_TCELL_DN	151	0.15	2.07	0.002016129	0.019635454
GSE40273_GATA1_KO_VS_WT_TREG_DN	184	0.13	2.07	0.008016032	0.019615125
SA_PROGRAMMED_CELL_DEATH	11	0.51	2.08	0.00390625	0.019579431
REACTOME_NONSENSE_MEDIATED_DECAY_ENHANCED_BY_THE_EXON_JUNCTI	70	0.21	2.08	<0.001	0.019566406
GO_MACROMOLECULE_CATABOLIC_PROCESS	741	0.07	2.08	<0.001	0.019399252
GSE40274_CTRL_VS_FOXP3_AND_GATA1_TRANSDUCED_ACTIVATED_CD4_TCEL	99	0.18	2.08	<0.001	0.019400006
GSE8515_CTRL_VS_IL1_4H_STIM_MAC_DN	149	0.14	2.08	0.002150538	0.019399278
GO_RIBONUCLEOPROTEIN_COMPLEX_BIOGENESIS	366	0.10	2.08	0.001926782	0.019273318
MORF_ACTG1	104	0.17	2.08	<0.001	0.019253355
GSE11961_GERMINAL_CENTER_BCELL_DAY7_VS_GERMINAL_CENTER_BCELL_D/	157	0.14	2.08	0.01010101	0.0192555
GO_REGULATION_OF_PLASMA_MEMBRANE_ORGANIZATION	60	0.23	2.08	<0.001	0.019235518
GO_REGULATION_OF_CELL_SIZE	138	0.15	2.08	0.005976096	0.019181564
VEGF_A_UP.V1_DN	167	0.14	2.08	<0.001	0.019178567
JNK_DN.V1_DN	121	0.16	2.08	0.00204918	0.019148923
GSE17186_BLOOD_VS_CORD_BLOOD_NAIVE_BCELL_UP	179	0.13	2.08	0.002024292	0.019139897
GO_RIBOSOME	173	0.14	2.08	0.006122449	0.019107241
GO_MITOTIC_CELL_CYCLE	673	0.07	2.08	<0.001	0.019058324
GSE10500_ARTHRITIC_SYNOVIAL_FLUID_VS_HEALTHY_MACROPHAGE_UP	120	0.16	2.08	0.002164502	0.018946458

GO_RESPONSE_TO_SALT_STRESS	12	0.49	2.08	0.002079002	0.01895064
GO_REGULATION_OF_WOUND_HEALING	93	0.19	2.08	0.002061856	0.018954119
GSE12845_PRE_GC_VS_DARKZONE_GC_TONSIL_BCELL_UP	143	0.15	2.08	0.00209205	0.018765396
GSE9988_ANTI_TREM1_VS_ANTI_TREM1_AND_LPS_MONOCYTE_DN	131	0.15	2.08	0.003976143	0.018776173
GSE39556_CD8A_DC_VS_NK_CELL_MOUSE_3H_POST_POLYIC_INJ_UP	175	0.14	2.08	<0.001	0.018691095
GSE17721_PAM3CSK4_VS_CPG_0.5H_BMDC_UP	172	0.14	2.09	0.002012072	0.01860516
GSE15330_LYMPHOID_MULTIPOTENT_VS_MEGAKARYOCYTE_ERYTHROID_PROG	147	0.15	2.09	0.004081633	0.018611388
GO_CELL_CYCLE_PROCESS	890	0.06	2.09	0.005940594	0.018619861
CHR5Q35	71	0.21	2.09	0.001976285	0.018571766
GO_POSITIVE_REGULATION_OF_EPITHELIAL_CELL_DIFFERENTIATION	32	0.31	2.09	0.002028398	0.018568333
KARAKAS_TGFB1_SIGNALING	14	0.45	2.09	0.003992016	0.018570034
GSE45365_HEALTHY_VS_MCMV_INFECTION_CD11B_DC_IFNAR_KO_DN	112	0.17	2.09	0.001996008	0.018497257
GSE17974_IL4_AND_ANTI_IL12_VS_UNTREATED_12H_ACT_CD4_TCELL_DN	98	0.18	2.09	0.002024292	0.018461714
REACTOME_TRAF6_MEDIATED_NFKB_ACTIVATION	17	0.43	2.09	0.008080808	0.018468639
GO_CYTOKINE_BINDING	60	0.23	2.09	0.003824092	0.01847707
GSE17721_POLYIC_VS_PAM3CSK4_2H_BMDC_DN	140	0.15	2.09	0.00811359	0.018457938
JEPSEN_SMRT_TARGETS	29	0.32	2.09	0.001980198	0.01843866
REACTOME_INTERFERON_SIGNALING	109	0.17	2.09	0.004065041	0.018407393
RODRIGUES_THYROID_CARCINOMA_ANAPLASTIC_UP	598	0.08	2.09	0.00617284	0.018393338
GO_VIRAL_LIFE_CYCLE	216	0.12	2.09	0.002	0.018392775
PRC2_EZH2_UP.V1_DN	141	0.15	2.09	0.009708738	0.018390661
PID_PI3KCI_PATHWAY	46	0.27	2.09	<0.001	0.018384036
KAECH_NAIVE_VS_MEMORY_CD8_TCELL_DN	160	0.14	2.09	0.002012072	0.018270086
GSE27786_LSK_VS_NKCELL_DN	176	0.13	2.09	0.003802281	0.01822877
BEGUM_TARGETS_OF_PAX3_FOXO1_FUSION_DN	40	0.28	2.09	0.004065041	0.01821827
VERHAAK_AML_WITH_NPM1_MUTATED_DN	186	0.13	2.09	0.005859375	0.01815207
GSE15930_NAIVE_VS_48H_IN_VITRO_STIM_IFNAB_CD8_TCELL_UP	154	0.15	2.09	<0.001	0.018101756
GSE26030_TH1_VS_TH17_DAY15_POST_POLARIZATION_DN	175	0.14	2.09	0.00204499	0.018081376
PID_CD40_PATHWAY	26	0.35	2.09	0.002020202	0.018040624
LIANG_SILENCED_BY_METHYLATION_2	32	0.31	2.09	<0.001	0.01801795
GO_ADENYL_NUCLEOTIDE_BINDING	1219	0.05	2.09	0.001988072	0.01782249
GO_POSITIVE_REGULATION_OF_LYMPHOCYTE_DIFFERENTIATION	52	0.25	2.09	<0.001	0.017817019
GSE8921_3H_VS_24H_TLR1_2_STIM_MONOCYTE_DN	150	0.15	2.09	0.002	0.017825868
GSE2770_IL12_AND_TGFB_ACT_VS_ACT_CD4_TCELL_6H_DN	172	0.14	2.09	0.007936508	0.017821135

QUINTENS_EMBRYONIC_BRAIN_RESPONSE_TO_IR	68	0.21	2.09	<0.001	0.017802812
GSE3920_IFNB_VS_IFNG_TREATED_ENDOTHELIAL_CELL_UP	136	0.15	2.10	0.001926782	0.017570134
COULOUARN_TEMPORAL_TGFB1_SIGNATURE_UP	96	0.19	2.10	0.001960784	0.017516008
MCBRYAN_PUBERTAL_TGFB1_TARGETS_UP	154	0.15	2.10	<0.001	0.017517138
GO_CELLULAR_RESPONSE_TO_EXTERNAL_STIMULUS	216	0.13	2.10	<0.001	0.017519742
GSE2585_CTEC_VS_MTEC_THYMUS_DN	145	0.15	2.10	0.003929273	0.017494347
GSE24726_WT_VS_E2-2_KO_PDC_DN	178	0.14	2.10	0.001897533	0.017478716
KEGG_RIG_I_LIKE_RECEPTOR_SIGNALING_PATHWAY	49	0.26	2.10	0.001923077	0.017400002
NUNODA_RESPONSE_TO_DASATINIB_IMATINIB_UP	28	0.33	2.10	0.002183406	0.01737449
GSE17580_TREG_VS_TEFF_S_MANSONI_INF_UP	159	0.14	2.10	<0.001	0.017305475
WILCOX_RESPONSE_TO_PROGESTERONE_DN	48	0.25	2.10	0.005736138	0.017288215
GSE7831_1H_VS_4H_INFLUENZA_STIM_PDC_DN	154	0.14	2.10	<0.001	0.017275453
GO_ACTIVATION_OF_MAPK_ACTIVITY	103	0.18	2.10	<0.001	0.017271837
GSE8621_UNSTIM_VS_LPS_PRIMED_AND_LPS_STIM_MACROPHAGE_UP	167	0.14	2.10	0.00407332	0.017278915
GSE14000_4H_VS_16H_LPS_DC_UP	156	0.15	2.10	0.003968254	0.01717586
GO_REGULATION_OF_DEFENSE_RESPONSE	537	0.08	2.10	0.002066116	0.017153863
GSE27786_CD8_TCELL_VS_MONO_MAC_DN	156	0.14	2.10	0.002024292	0.017159367
WINNEPENNINCKX_MELANOMA_METASTASIS_UP	146	0.15	2.10	<0.001	0.017131245
GSE2405_S_AUREUS_VS_UNTREATED_NEUTROPHIL_DN	165	0.14	2.10	0.004008016	0.017009797
GILMORE_CORE_NFKB_PATHWAY	14	0.46	2.10	<0.001	0.01692033
GO_IMMUNE_SYSTEM_DEVELOPMENT	437	0.09	2.11	0.001972387	0.016852077
GO_NEGATIVE_REGULATION_OF_INTERLEUKIN_2_PRODUCTION	11	0.51	2.11	0.003809524	0.016862761
GSE17721_POLYIC_VS_GARDIQUIMOD_16H_BMDC_UP	173	0.13	2.11	<0.001	0.01686737
GSE16385_ROSIGLITAZONE_IFNG_TNF_VS_IL4_STIM_MACROPHAGE_UP	151	0.15	2.11	0.004024145	0.016746119
GSE7218_IGM_VS_IGG_SIGNAL_THGOUGH_ANTIGEN_BCELL_DN	120	0.17	2.11	0.004048583	0.016723726
MODULE_18	370	0.10	2.11	0.001956947	0.016732834
RHODES_UNDIFFERENTIATED_CANCER	63	0.23	2.11	<0.001	0.016741939
GO_PROTEIN_KINASE_ACTIVITY	520	0.08	2.11	0.001945525	0.016701164
CHR20Q13	105	0.18	2.11	<0.001	0.01668559
GSE557_WT_VS_CIITA_KO_DC_DN	172	0.14	2.11	0.004106776	0.016684685
GO_CYTOSKELETAL_PART	1068	0.06	2.11	0.004132231	0.016636722
BHATI_G2M_ARREST_BY_2METHOXYESTRADIOL_UP	85	0.19	2.11	0.003992016	0.016583245
WEST_ADRENOCORTICAL_TUMOR_MARKERS_UP	18	0.41	2.11	<0.001	0.016539017
SIMBULAN_UV_RESPONSE_IMMORTALIZED_DN	30	0.32	2.11	0.002066116	0.016476208

TURASHVILI_BREAST_DUCTAL_CARCINOMA_VS_DUCTAL_NORMAL_UP	35	0.30	2.11	<0.001	0.016426388
GSE5099_UNSTIM_VS_MCSF_TREATED_MONOCYTE_DAY3_DN	177	0.14	2.11	0.001988072	0.016406681
GSE21360_NAIVE_VS_PRIMARY_MEMORY_CD8_TCELL_UP	147	0.15	2.11	0.004040404	0.016405538
RUTELLA_RESPONSE_TO_CSF2RB_AND_IL4_DN	252	0.12	2.11	<0.001	0.016380494
GO_MOTOR_ACTIVITY	94	0.18	2.11	0.00204499	0.01638014
GO_POSITIVE_REGULATION_OF_ENDOTHELIAL_CELL_DIFFERENTIATION	12	0.49	2.11	0.002132196	0.016385995
GSE23321_EFFECTOR_MEMORY_VS_NAIVE_CD8_TCELL_DN	164	0.14	2.11	0.006024096	0.016349945
GSE14415_INDUCED_TREG_VS_TCONV_UP	152	0.15	2.11	0.005882353	0.016299149
GO_REGULATION_OF_TRANSFERASE_ACTIVITY	755	0.07	2.11	0.001996008	0.016196152
GSE2128_C57BL6_VS_NOD_THYMOCYTE_DN	159	0.15	2.11	<0.001	0.016204245
RGTTAMWNATT_HNF1_01	72	0.21	2.11	0.002079002	0.016212333
GO_ACTIN_DEPENDENT_ATPASE_ACTIVITY	12	0.50	2.11	<0.001	0.016160524
GSE18281_PERIMEDULLARY_CORTICAL_REGION_VS_WHOLE_CORTEX_THYMUS_	155	0.15	2.11	0.003809524	0.016156942
PID_FCER1_PATHWAY	56	0.24	2.11	0.001968504	0.016157221
GO_NEGATIVE_REGULATION_OF_VIRAL_PROCESS	61	0.23	2.11	0.002053388	0.016155183
GO_CORTICAL_CYTOSKELETON	68	0.22	2.11	0.004040404	0.01615468
REACTOME_CYTOKINE_SIGNALING_IN_IMMUNE_SYSTEM	205	0.13	2.12	0.005847953	0.01611118
KEGG_CYTOSOLIC_DNA_SENSING_PATHWAY	38	0.29	2.12	0.001953125	0.016059777
MODULE_439	16	0.44	2.12	<0.001	0.015945874
GSE21360_NAIVE_VS_SECONDARY_MEMORY_CD8_TCELL_DN	151	0.15	2.12	0.001956947	0.015859194
STAMBOLSKY_TARGETS_OF_MUTATED_TP53_DN	31	0.31	2.12	0.001949318	0.01586632
GSE7831_1H_VS_4H_CPG_STIM_PDC_DN	157	0.15	2.12	0.006134969	0.015865615
GSE24574_BCL6_HIGH_TFH_VS_NAIVE_CD4_TCELL_DN	172	0.14	2.12	0.001919386	0.01585474
GO_NEGATIVE_REGULATION_OF_LOCOMOTION	200	0.13	2.12	0.002020202	0.01581483
GO_RHO_PROTEIN_SIGNAL_TRANSDUCTION	45	0.27	2.12	0.001972387	0.015722238
KINSEY_TARGETS_OF_EWSR1_FLII_FUSION_DN	273	0.11	2.12	0.002114165	0.015720667
KAPOSI_LIVER_CANCER_MET_UP	16	0.44	2.12	0.004106776	0.0156649
GSE3982_DC_VS_NEUTROPHIL_LPS_STIM_DN	139	0.15	2.12	<0.001	0.015667979
MODULE_79	75	0.21	2.12	<0.001	0.015660858
NABA_MATRISOME_ASSOCIATED	345	0.10	2.12	0.003846154	0.015655326
GSE7509_UNSTIM_VS_IFNA_STIM_IMMATURE_DC_UP	158	0.15	2.12	0.001956947	0.015645849
GSE3565_DUSP1_VS_WT_SPLENOCYTES_UP	136	0.15	2.12	0.002079002	0.015625266
GSE30083_SP1_VS_SP2_THYMOCYTE_UP	157	0.14	2.12	0.00189394	0.015622826
GO_REGULATION_OF_EXTRINSIC_APOPTOTIC_SIGNALING_PATHWAY_IN_ABSEN	36	0.30	2.12	<0.001	0.015595946

GO_CORTICAL_ACTIN_CYTOSKELETON	51	0.25	2.12	0.002012072	0.015599029
PID_S1P_S1P3_PATHWAY	27	0.33	2.12	0.003968254	0.015553098
GSE21546_SAP1A_KO_VS_SAP1A_KO_AND_ELK1_KO_DP_THYMOCYTES_UP	168	0.14	2.12	0.001972387	0.015543538
GSE46606_UNSTIM_VS_CD40L_IL2_IL5_3DAY_STIMULATED_IRF4_KO_BCELL_DN	146	0.15	2.12	<0.001	0.015470665
GSE17974_IL4_AND_ANTI_IL12_VS_UNTREATED_24H_ACT_CD4_TCELL_DN	98	0.19	2.12	0.001980198	0.01544044
GSE14000_UNSTIM_VS_4H_LPS_DC_TRANSLATED_RNA_DN	139	0.15	2.12	<0.001	0.015433124
GSE360_CTRL_VS_T_GONDII_DC_DN	142	0.16	2.12	0.001992032	0.015432109
GSE16385_MONOCYTE_VS_12H_IFNG_TNF_TREATED_MACROPHAGE_UP	172	0.14	2.12	0.002016129	0.015427967
GO_REGULATION_OF_CHEMOTAXIS	133	0.16	2.13	<0.001	0.015209285
GSE39864_WT_VS_GATA3_KO_TREG_UP	149	0.14	2.13	<0.001	0.015169199
GSE12366_GC_VS_NAIVE_BCELL_DN	148	0.15	2.13	0.001901141	0.015137017
GSE12963_ENV_NEF_VS_ENV_NEF_AND_VPR_DEFICIENT_HIV1_INF_CD4_TCELL	99	0.18	2.13	0.004024145	0.015088017
GSE21380_TFH_VS_GERMINAL_CENTER_TFH_CD4_TCELL_DN	159	0.15	2.13	0.002061856	0.01507489
GROSS_HYPOXIA_VIA_ELK3_AND_HIF1A_DN	98	0.18	2.13	0.002118644	0.014974896
GSE2935_UV_INACTIVATED_VS_LIVE_SENDAI_VIRUS_INF_MACROPHAGE_UP	136	0.15	2.13	0.002	0.014921736
AMIT_SERUM_RESPONSE_240_MCF10A	43	0.27	2.13	0.002105263	0.014686692
GSE26488_WT_VS_VP16_TRANSGENIC_HDAC7_KO_DOUBLE_POSITIVE_THYMO	134	0.16	2.13	0.007843138	0.014663582
REACTOME_THE_NLRP3_INFLAMMASOME	10	0.55	2.13	0.003968254	0.014667694
REACTOME_N_GLYCAN_ANTENNAE_ELONGATION	11	0.53	2.13	<0.001	0.014647055
GSE43955_TH0_VS_TGFB_IL6_TH17_ACT_CD4_TCELL_52H_UP	159	0.15	2.13	<0.001	0.014653562
GO_EMBRYO_DEVELOPMENT	638	0.08	2.13	0.003795066	0.014662463
GSE3982_CENT_MEMORY_CD4_TCELL_VS_NKCELL_DN	147	0.15	2.13	0.001984127	0.014559958
LTE2_UP.V1_DN	152	0.15	2.14	<0.001	0.014544818
GSE7460_TCONV_VS_TREG_LN_DN	171	0.14	2.14	0.002066116	0.014553679
FLECHNER_BIOPSY_KIDNEY_TRANSPLANT_REJECTED_VS_OK_UP	68	0.21	2.14	0.004008016	0.014536038
GSE11057_NAIVE_VS_CENT_MEMORY_CD4_TCELL_DN	173	0.14	2.14	<0.001	0.014519958
GSE9006_TYPE_1_VS_TYPE_2_DIABETES_PBMC_AT_DX_UP	180	0.14	2.14	<0.001	0.014500726
GSE26488_CTRL_VS_PEPTIDE_INJECTION_HDAC7_DELTAP_TG_OT2_THYMOCYT	181	0.14	2.14	0.005660377	0.014505561
GSE42724_MEMORY_BCELL_VS_PLASMABLAST_UP	174	0.14	2.14	0.004	0.014489444
GSE29618_MONOCYTE_VS_PDC_DAY7_FLU_VACCINE_UP	172	0.15	2.14	0.001872659	0.014497491
GSE6269_FLU_VS_STREP_PNEUMO_INF_PBMC_UP	132	0.16	2.14	0.002087683	0.014495058
GSE37301_CD4_TCELL_VS_GRANULOCYTE_MONOCYTE_PROGENITOR_DN	144	0.15	2.14	0.002020202	0.014466011
UDAYAKUMAR_MED1_TARGETS_DN	207	0.12	2.14	0.002053388	0.014452287
GSE8685_IL2_ACT_IL2_STARVED_VS_IL21_ACT_IL2_STARVED_CD4_TCELL_UP	147	0.15	2.14	0.004255319	0.014438535

MACLACHLAN_BRCA1_TARGETS_DN	13	0.50	2.14	0.003898636	0.014431175
GSE24574_NAIVE_VS_TCONV_CD4_TCELL_UP	152	0.15	2.14	0.001972387	0.014386631
PID_CD8_TCR_PATHWAY	42	0.28	2.14	<0.001	0.014388946
GSE37301_LYMPHOID_PRIMED_MPP_VS_GRAN_MONO_PROGENITOR_UP	164	0.14	2.14	0.001996008	0.014396978
GSE43955_TGFB_IL6_VS_TGFB_IL6_IL23_TH17_ACT_CD4_TCELL_52H_DN	175	0.14	2.14	0.001980198	0.014386414
DOUGLAS_BMI1_TARGETS_UP	461	0.09	2.14	0.001964637	0.014351627
XU_HGF_SIGNALING_NOT_VIA_AKT1_6HR	23	0.37	2.14	<0.001	0.014314168
GO_MACROPHAGE_ACTIVATION	25	0.35	2.14	0.00203666	0.0142327
GO_CELL_CELL_JUNCTION	277	0.11	2.14	0.003960396	0.014228326
GO_REGULATION_OF_INTERLEUKIN_1_PRODUCTION	34	0.32	2.14	0.004016064	0.014140968
JOHNSTONE_PARVB_TARGETS_2_UP	119	0.17	2.14	0.003752345	0.01411389
GSE40274_CTRL_VS_HELIOS_TRANSDUCED_ACTIVATED_CD4_TCELL_DN	140	0.16	2.14	<0.001	0.014123393
AP2ALPHA_01	185	0.13	2.14	0.002057613	0.01402055
GSE22432_MULTIPOTENT_VS_COMMON_DC_PROGENITOR_DN	159	0.14	2.14	0.002087683	0.014012832
ONDER_CDH1_TARGETS_2_DN	301	0.11	2.14	0.001984127	0.013917016
MOLENAAR_TARGETS_OF_CCND1_AND_CDK4_DN	48	0.26	2.15	0.004	0.013812189
KAECH_NAIVE_VS_DAY15_EFF_CD8_TCELL_DN	160	0.14	2.15	<0.001	0.013763457
GROSS_HYPOXIA_VIA_ELK3_UP	190	0.14	2.15	<0.001	0.013738457
GSE30971_CTRL_VS_LPS_STIM_MACROPHAGE_WBP7_HET_2H_UP	141	0.16	2.15	0.002024292	0.013742855
ELK1_02	225	0.12	2.15	<0.001	0.013682523
GSE43955_TH0_VS_TGFB_IL6_TH17_ACT_CD4_TCELL_1H_UP	157	0.15	2.15	0.001988072	0.013508854
GSE32986_UNSTIM_VS_CURDLAN_LOWDOSE_STIM_DC_UP	177	0.14	2.15	0.002123142	0.013461391
GSE30083_SP2_VS_SP4_THYMOCYTE_DN	162	0.14	2.15	0.001937985	0.01346398
GO_REGULATION_OF_CELL_DIVISION	209	0.13	2.15	0.001992032	0.013460796
GSE15215_CD2_POS_VS_NEG_PDC_UP	145	0.16	2.15	0.001976285	0.013463372
RUTELLA_RESPONSE_TO_HGF_DN	191	0.13	2.15	<0.001	0.013451966
ZHANG_TLX_TARGETS_60HR_DN	248	0.12	2.15	0.008264462	0.013427347
GO_CALCIUM_ION_BINDING	393	0.09	2.15	0.001919386	0.013298319
WINZEN_DEGRADED_VIA_KHSRP	75	0.21	2.15	0.004040404	0.013306548
GSE21380_NON_TFH_VS_TFH_CD4_TCELL_UP	177	0.14	2.15	0.004405286	0.013313977
BIOCARTA_NFKB_PATHWAY	21	0.40	2.15	0.00209205	0.013264451
GSE41978_ID2_KO_VS_ID2_KO_AND_BIM_KO_KLRG1_LOW_EFFECTOR_CD8_TC	159	0.14	2.15	0.003891051	0.013271053
GSE15624_CTRL_VS_3H_HALOFUGINONE_TREATED_CD4_TCELL_DN	129	0.16	2.15	0.004310345	0.013198328
LIU_PROSTATE_CANCER_DN	369	0.10	2.15	0.00203252	0.013170993

GO_AMINOGLYCAN_BIOSYNTHETIC_PROCESS	81	0.21	2.15	0.003759399	0.013178341
GSE36888_UNTREATED_VS_IL2_TREATED_TCELL_2H_UP	154	0.15	2.16	0.003937008	0.01316336
GSE45739_NRAS_KO_VS_WT_ACD3_ACD28_STIM_CD4_TCELL_UP	167	0.14	2.16	0.001968504	0.013155817
GSE43863_NAIVE_VS_TFH_CD4_EFF_TCELL_D6_LCMV_UP	155	0.15	2.16	0.002012072	0.013161526
GSE13484_UNSTIM_VS_3H_YF17D_VACCINE_STIM_PBMC_DN	159	0.15	2.16	0.001838235	0.013166415
GSE4748_CYANOBACTERIUM_LPSLIKE_VS_LPS_AND_CYANOBACTERIUM_LPSLIK	105	0.18	2.16	0.005940594	0.013147973
PHONG_TNF_RESPONSE_VIA_P38_COMPLETE	206	0.13	2.16	0.001851852	0.013149527
GSE26890_CXCR1_NEG_VS_POS_EFFECTOR_CD8_TCELL_DN	171	0.14	2.16	0.006396588	0.013076354
GSE13485_CTRL_VS_DAY21_YF17D_VACCINE_PBMC_UP	172	0.14	2.16	<0.001	0.013035469
ANASTASSIOU_MULTICANCER_INVASIVENESS_SIGNATURE	54	0.25	2.16	0.001949318	0.012983684
BIOCARTA_UCALPAIN_PATHWAY	16	0.45	2.16	<0.001	0.012963524
HOLLEMAN_ASPARAGINASE_RESISTANCE_B_ALL_DN	14	0.48	2.16	0.002096436	0.012923234
PID_LYMPH_ANGIOGENESIS_PATHWAY	25	0.37	2.16	0.004048583	0.012931321
LANDIS_ERBB2_BREAST_TUMORS_65_UP	20	0.40	2.16	0.001988072	0.012899435
GO_POSITIVE_REGULATION_OF_ACTIN_FILAMENT_BUNDLE_ASSEMBLY	39	0.30	2.16	<0.001	0.012836505
LI_WILMS_TUMOR_ANAPLASTIC_UP	18	0.42	2.16	0.002066116	0.012802839
GSE3982_EFF_MEMORY_VS_CENT_MEMORY_CD4_TCELL_UP	132	0.16	2.16	0.002061856	0.01272558
GO_REGULATION_OF_CELL_ACTIVATION	327	0.10	2.16	0.005725191	0.012550977
RPS14_DN.V1_UP	138	0.16	2.16	0.002012072	0.01254794
DORSEY_GAB2_TARGETS	21	0.39	2.17	<0.001	0.012484596
GO_NUCLEAR_TRANSCRIBED_MRNA_CATABOLIC_PROCESS_NONSENSE_MEDIAT	83	0.20	2.17	0.002	0.012493255
GO_KERATAN_SULFATE_METABOLIC_PROCESS	26	0.35	2.17	0.002074689	0.012427177
GSE21670_IL6_VS_TGFB_AND_IL6_TREATED_STAT3_KO_CD4_TCELL_DN	162	0.15	2.17	<0.001	0.012434144
GSE11864_CSF1_VS_CSF1_IFNG_PAM3CYS_IN_MAC_DN	144	0.16	2.17	0.006024096	0.012426813
PID_RHOA_REG_PATHWAY	41	0.29	2.17	0.002109705	0.012382561
GO_RESPONSE_TO_PROGESTERONE	34	0.31	2.17	0.003875969	0.012259139
HOXA9_DN.V1_UP	157	0.15	2.17	0.001960784	0.012198654
GO_ENDOCYTIC_VESICLE	186	0.13	2.17	0.00204499	0.012020191
GO_TRANSFERASE_ACTIVITY_TRANSFERRING_PHOSPHORUS_CONTAINING_GRC	818	0.07	2.17	<0.001	0.012016725
GO_POSITIVE_REGULATION_OF_MAPK_CASCADE	338	0.11	2.17	0.001956947	0.011970252
GSE18893_TCONV_VS_TREG_24H_CULTURE_UP	167	0.14	2.17	0.001980198	0.011955841
HUMMERICH_SKIN_CANCER_PROGRESSION_UP	72	0.22	2.17	0.004040404	0.011947323
MARSON_BOUND_BY_E2F4_UNSTIMULATED	603	0.08	2.17	0.002057613	0.011938803
SAKAI_TUMOR_INFILTRATING_MONOCYTES_UP	23	0.38	2.17	0.003976143	0.011947157

GSE339_CD8POS_VS_CD4CD8DN_DC_IN_CULTURE_UP	165	0.15	2.17	<0.001	0.011952975
GSE21033_CTRL_VS_POLYIC_STIM_DC_6H_UP	139	0.16	2.17	0.00408998	0.01192827
DER_IFN_BETA_RESPONSE_UP	85	0.20	2.17	<0.001	0.011908675
REACTOME_RIG_I_MDA5_MEDIATED_INDUCTION_OF_IFN_ALPHA_BETA_PATHW	51	0.26	2.17	0.002020202	0.011913646
GO_INACTIVATION_OF_MAPK_ACTIVITY	21	0.39	2.17	0.002012072	0.011910941
GSE15767_MED_VS_SCS_MAC_LN_UP	161	0.15	2.17	0.001908397	0.011897264
GO_FORMATION_OF_PRIMARY_GERM_LAYER	83	0.21	2.18	0.00203252	0.011818907
MODULE_315	16	0.45	2.18	<0.001	0.011817025
BIOCARTA_INTEGRIN_PATHWAY	36	0.31	2.18	0.003861004	0.011622813
GO_POSITIVE_REGULATION_OF_RESPONSE_TO_EXTERNAL_STIMULUS	201	0.13	2.18	<0.001	0.011580703
GSE45739_UNSTIM_VS_ACD3_ACD28_STIM_WT_CD4_TCELL_DN	147	0.15	2.18	<0.001	0.011562429
GSE10240_CTRL_VS_IL17_AND_IL22_STIM_PRIMARY_BRONCHIAL_EPITHELIAL_(	164	0.14	2.18	0.006134969	0.011518575
GO_CARGO_RECEPTOR_ACTIVITY	39	0.30	2.18	<0.001	0.011507964
KRIGE_RESPONSE_TO_TOSEDOSTAT_6HR_DN	761	0.07	2.18	0.002164502	0.011442523
SERVITJA_ISLET_HNF1A_TARGETS_UP	122	0.17	2.18	<0.001	0.011404438
GO_AMEBOIDAL_TYPE_CELL_MIGRATION	111	0.18	2.18	<0.001	0.011403052
MODULE_451	29	0.34	2.18	<0.001	0.011408542
GO_POSITIVE_REGULATION_OF_MAP_KINASE_ACTIVITY	163	0.15	2.18	0.002083333	0.011323228
GSE9509_LPS_VS_LPS_AND_IL10_STIM_IL10_KO_MACROPHAGE_30MIN_DN	141	0.15	2.18	0.00189394	0.011316653
GSE24726_WT_VS_E2-2_KO_PDC_DAY4_POST_DELETION_UP	176	0.14	2.18	0.00203252	0.011300609
REACTOME_INTEGRIN_ALPHAIIB_BETA3_SIGNALING	27	0.35	2.18	0.001988072	0.011287198
GO_NEGATIVE_REGULATION_OF_WOUND_HEALING	39	0.29	2.18	0.004048583	0.011294371
GSE29618_BCELL_VS_MONOCYTE_DAY7_FLU_VACCINE_DN	169	0.15	2.18	<0.001	0.011295533
MODULE_114	272	0.12	2.18	0.006036217	0.011294098
GSE37534_PIOGLITAZONE_VS_ROSIGLITAZONE_TREATED_CD4_TCELL_PPARG1_	125	0.17	2.18	0.001992032	0.011252208
GSE7348_UNSTIM_VS_LPS_STIM_MACROPHAGE_DN	137	0.16	2.18	0.001897533	0.011251592
GO_NCRNA_PROCESSING	323	0.11	2.19	0.00204918	0.011210437
GSE34006_WT_VS_A2AR_KO_TREG_DN	170	0.15	2.19	<0.001	0.011197726
GSE3982_NEUTROPHIL_VS_TH2_UP	156	0.15	2.19	<0.001	0.011173794
GSE9988_ANTI_TREM1_VS_LPS_MONOCYTE_DN	154	0.15	2.19	<0.001	0.011161861
GSE6269_FLU_VS_STAPH_AUREUS_INF_PBMC_DN	149	0.15	2.19	0.003861004	0.011134377
GSE17721_CTRL_VS_CPG_6H_BMDC_DN	161	0.15	2.19	<0.001	0.011031425
GO_SPINDLE_ASSEMBLY	63	0.23	2.19	0.001941748	0.011028933
GSE14769_UNSTIM_VS_20MIN_LPS_BMDM_DN	149	0.15	2.19	<0.001	0.011021233

GSE7831_UNSTIM_VS_CPG_STIM_PDC_1H_UP	172	0.14	2.19	<0.001	0.011027413
GSE28737_FOLLICULAR_VS_MARGINAL_ZONE_BCELL_BCL6_HET_DN	139	0.16	2.19	<0.001	0.011033591
GO_REGULATION_OF_ATTACHMENT_OF_SPINDLE_MICROTUBULES_TO_KINETO	10	0.56	2.19	<0.001	0.011038047
GSE360_LOW_DOSE_B_MALAYI_VS_M_TUBERCULOSIS_MAC_UP	155	0.15	2.19	<0.001	0.011008548
GO_CYTOKINESIS	78	0.20	2.19	<0.001	0.010917354
GSE40666_UNTREATED_VS_IFNA_STIM_STAT1_KO_CD8_TCELL_90MIN_UP	117	0.18	2.19	<0.001	0.010890324
GSE7852_TREG_VS_TCONV_DN	166	0.14	2.19	<0.001	0.010859013
GSE7509_UNSTIM_VS_FCGRIIB_STIM_DC_DN	133	0.17	2.19	0.004008016	0.010848529
GO_BLOOD_VESSEL_ENDOTHELIAL_CELL_MIGRATION	21	0.40	2.19	<0.001	0.010828487
MORI_PLASMA_CELL_DN	24	0.37	2.19	<0.001	0.010776969
GSE21546_SAP1A_KO_VS_SAP1A_KO_AND_ELK1_KO_ANTI_CD3_STIM_DP_THY	174	0.14	2.20	<0.001	0.01066167
BIOCARTA_G2_PATHWAY	22	0.39	2.20	0.001908397	0.010609056
GRAESSMANN_RESPONSE_TO_MC_AND_SERUM_DEPRIVATION_UP	168	0.14	2.20	0.002114165	0.010522285
GO_NEGATIVE_REGULATION_OF_COAGULATION	31	0.33	2.20	<0.001	0.010478226
GO_GOLGI_APPARATUS_PART	666	0.08	2.20	0.001992032	0.010468311
GSE8515_IL1_VS_IL6_4H_STIM_MAC_UP	159	0.15	2.20	0.005836576	0.010463648
GO_LYMPHOCYTE_ACTIVATION	242	0.12	2.20	0.002070393	0.010465962
GO_REGULATION_OF_INTERFERON_BETA_PRODUCTION	41	0.29	2.20	0.002040816	0.010421818
GERHOLD_ADIPOGENESIS_DN	61	0.24	2.20	<0.001	0.010410029
SANSOM_APC_TARGETS_REQUIRE_MYC	180	0.14	2.20	<0.001	0.010391162
KEGG_LEUKOCYTE_TRANSENDOTHELIAL_MIGRATION	88	0.20	2.20	0.002057613	0.010389053
DURCHDEWALD_SKIN_CARCINOGENESIS_DN	226	0.13	2.20	0.001953125	0.010383368
GO_KINASE_ACTIVITY	687	0.07	2.20	0.003780718	0.010374248
GO_CELL_PROJECTION	1256	0.06	2.20	<0.001	0.010327154
MODULE_403	44	0.28	2.20	<0.001	0.010302966
GO_MIDBODY	118	0.17	2.20	0.005882353	0.010243442
GSE17186_MEMORY_VS_NAIVE_BCELL_DN	169	0.15	2.20	0.001915709	0.010229695
GSE24634_IL4_VS_CTRL_TREATED_NAIVE_CD4_TCELL_DAY7_UP	159	0.15	2.20	<0.001	0.010237178
GO_NEGATIVE_REGULATION_OF_VIRAL_GENOME_REPLICATION	34	0.31	2.20	0.00203252	0.010235826
MISSIAGLIA_REGULATED_BY_METHYLATION_UP	94	0.20	2.20	0.001930502	0.010223001
GO_CELL_CORTEX_PART	100	0.19	2.20	0.001897533	0.010211859
GSE37532_WT_VS_PPARG_KO_VISCERAL_ADIPOSE_TISSUE_TCONV_UP	164	0.15	2.20	0.001980198	0.010212252
GO_MICROTUBULE_BASED_PROCESS	410	0.10	2.20	<0.001	0.010211742
GSE37301_CD4_TCELL_VS_RAG2_KO_NK_CELL_UP	103	0.18	2.20	<0.001	0.010208586

GSE1460_INTRATHYMIC_T_PROGENITOR_VS_THYMIC_STROMAL_CELL_DN	164	0.15	2.20	0.001980198	0.01020987
GSE39820_CTRL_VS_IL1B_IL6_IL23A_CD4_TCELL_DN	169	0.14	2.20	0.001984127	0.010210314
FIGUEROA_AML_METHYLATION_CLUSTER_3_DN	27	0.35	2.21	<0.001	0.010126992
GAURNIER_PSMD4_TARGETS	35	0.31	2.21	<0.001	0.010096125
GSE20715_0H_VS_48H_OZONE_TLR4_KO_LUNG_DN	177	0.14	2.21	<0.001	0.010084854
KEGG_GLYCOSAMINOGLYCAN_BIOSYNTHESIS_CHONDROITIN_SULFATE	18	0.43	2.21	<0.001	0.010056576
GSE9988_ANTI_TREM1_AND_LPS_VS_VEHICLE_TREATED_MONOCYTES_UP	125	0.18	2.21	<0.001	0.010047018
LEF1_UP.V1_UP	122	0.17	2.21	<0.001	0.0100071
DIAZ_CHRONIC_MEYLOGENOUS_LEUKEMIA_DN	83	0.21	2.21	0.002057613	0.010009148
GNF2_EIF3S6	83	0.21	2.21	<0.001	0.009945008
GSE3982_EOSINOPHIL_VS_NEUTROPHIL_DN	148	0.16	2.21	<0.001	0.009912964
GSE11961_MARGINAL_ZONE_BCELL_VS_GERMINAL_CENTER_BCELL_DAY40_DN	152	0.15	2.21	<0.001	0.009875556
GO_POSITIVE_REGULATION_OF_STRESS_ACTIVATED_PROTEIN_KINASE_SIGNALI	110	0.18	2.21	0.004376368	0.009849661
GSE4748_LPS_VS_LPS_AND_CYANOBACTERIUM_LPSLIKE_STIM_DC_3H_UP	154	0.15	2.21	<0.001	0.009833651
MONNIER_POSTRADIATION_TUMOR_ESCAPE_UP	360	0.10	2.21	<0.001	0.009824812
ABE_VEGFA_TARGETS_30MIN	22	0.39	2.21	<0.001	0.009792619
GO_MYD88_INDEPENDENT_TOLL_LIKE_RECEPTOR_SIGNALING_PATHWAY	28	0.36	2.21	0.002028398	0.009796316
LABBE_TGFB1_TARGETS_UP	72	0.22	2.21	<0.001	0.009748543
GO_TOLL_LIKE_RECEPTOR_9_SIGNALING_PATHWAY	13	0.50	2.21	<0.001	0.009693634
GSE17186_NAIVE_VS_CD21LOW_TRANSITIONAL_BCELL_UP	179	0.14	2.21	<0.001	0.009688259
REACTOME_INTERFERON_GAMMA_SIGNALING	40	0.29	2.22	0.002096436	0.009654816
GSE42021_CD24HI_VS_CD24LOW_TCONV_THYMUS_DN	154	0.15	2.22	<0.001	0.009577943
GO_PROTEIN_DOMAIN_SPECIFIC_BINDING	494	0.09	2.22	<0.001	0.00958329
PDGF_ERK_DN.V1_DN	124	0.17	2.22	<0.001	0.009519884
GSE14769_UNSTIM_VS_240MIN_LPS_BMDM_DN	178	0.15	2.22	<0.001	0.0095261
GSE6092_B_BURGDOFERI_VS_B_BURGDORFERI_AND_IFNG_STIM_ENDOTHELIA	161	0.15	2.22	0.001879699	0.009528703
RASHI_NFKB1_TARGETS	17	0.44	2.22	0.003838772	0.009499586
GSE41978_ID2_KO_VS_BIM_KO_KLRG1_LOW_EFFECTOR_CD8_TCELL_UP	148	0.16	2.22	0.002066116	0.009490394
GO_NEGATIVE_REGULATION_OF_HEMOPOIESIS	81	0.21	2.22	<0.001	0.009489336
LEE_TARGETS_OF_PTCH1_AND_SUFU_UP	44	0.29	2.22	<0.001	0.009435473
GNF2_PTPRC	55	0.25	2.22	0.00390625	0.009428017
GSE39820_CTRL_VS_IL1B_IL6_CD4_TCELL_DN	161	0.15	2.22	0.001926782	0.009412372
GSE7852_LN_VS_FAT_TCONV_DN	175	0.15	2.22	<0.001	0.009375873
GO_REGULATION_OF_SMOOTH_MUSCLE_CELL_MIGRATION	47	0.27	2.22	0.001949318	0.009362847

EGUCHI_CELL_CYCLE_RB1_TARGETS	22	0.40	2.22	0.003937008	0.009351675
GSE5679_CTRL_VS_PPARG_LIGAND_ROSIGLITAZONE_TREATED_DC_UP	176	0.14	2.22	0.003921569	0.009356904
GNF2_CD97	32	0.33	2.22	0.003952569	0.00936397
GSE14000_TRANSLATED_RNA_VS_MRNA_16H_LPS_DC_DN	142	0.16	2.22	<0.001	0.009360975
GSE24634_TREG_VS_TCONV_POST_DAY7_IL4_CONVERSION_DN	167	0.15	2.22	0.001988072	0.009354387
MOREAUX_MULTIPLE_MYELOMA_BY_TACI_UP	251	0.12	2.22	<0.001	0.00928922
GNF2_FOS	33	0.33	2.22	<0.001	0.009293504
GSE21033_1H_VS_24H_POLYIC_STIM_DC_DN	132	0.17	2.22	0.005836576	0.009283158
GO_SH3_DOMAIN_BINDING	97	0.20	2.22	<0.001	0.009289282
GSE36527_CD62L_HIGH_CD69_NEG_VS_CD62L_LOW_CD69_POS_TREG_KLRG1_	155	0.15	2.22	<0.001	0.009285323
GO_CELL_MORPHOGENESIS_INVOLVED_IN_DIFFERENTIATION	364	0.10	2.22	0.002016129	0.009266648
GSE17721_0.5H_VS_4H_LPS_BMDC_DN	149	0.15	2.22	0.001941748	0.009259933
MODULE_174	78	0.22	2.22	<0.001	0.009258704
PID_FAK_PATHWAY	56	0.25	2.22	<0.001	0.009228879
GO_RIBOSOMAL_SUBUNIT	117	0.18	2.23	<0.001	0.009192583
GSE7768_OVA_ALONE_VS_OVA_WITH_MPL_IMMUNIZED_MOUSE_WHOLE_SPL	145	0.16	2.23	0.00203666	0.009150727
GSE32533_MIR17_KO_VS_MIR17_OVEREXPRESS_ACT_CD4_TCELL_DN	126	0.17	2.23	0.002	0.008950877
CHIARADONNA_NEOPLASTIC_TRANSFORMATION_CDC25_DN	146	0.16	2.23	<0.001	0.008952179
GSE411_UNSTIM_VS_100MIN_IL6_STIM_SOCS3_KO_MACROPHAGE_DN	177	0.14	2.23	<0.001	0.008928513
GSE16385_UNTREATED_VS_12H_ROSIGLITAZONE_IL4_TREATED_MACROPHAGE	178	0.14	2.23	0.003861004	0.00890117
BOWIE_RESPONSE_TO_EXTRACELLULAR_MATRIX	13	0.52	2.23	<0.001	0.008898753
LINDGREN_BLADDER_CANCER_HIGH_RECURRENCE	44	0.28	2.23	0.001949318	0.00890279
GO_MITOTIC_NUCLEAR_DIVISION	315	0.11	2.23	<0.001	0.008868809
YAMAZAKI_TCEB3_TARGETS_UP	151	0.15	2.23	0.002087683	0.008845881
AMIT_EGF_RESPONSE_60_MCF10A	36	0.31	2.23	<0.001	0.00882207
PU1_Q6	176	0.14	2.23	0.002020202	0.008693981
MARKEY_RB1_ACUTE_LOF_UP	202	0.14	2.24	0.002028398	0.008660675
GO_REGULATION_OF_CELL_DEVELOPMENT	611	0.08	2.24	<0.001	0.008663599
GSE2770_UNTREATED_VS_IL4_TREATED_ACT_CD4_TCELL_2H_UP	146	0.16	2.24	<0.001	0.008645047
GO_CHONDROITIN_SULFATE_PROTEOGLYCAN_BIOSYNTHETIC_PROCESS	23	0.38	2.24	0.001996008	0.008642398
GSE36888_UNTREATED_VS_IL2_TREATED_TCELL_17H_DN	140	0.16	2.24	0.001915709	0.008637875
GSE23568_ID3_KO_VS_WT_CD8_TCELL_DN	183	0.14	2.24	0.003898636	0.008617482
GSE11961_GERMINAL_CENTER_BCELL_DAY7_VS_PLASMA_CELL_DAY7_UP	153	0.16	2.24	0.002040816	0.00857091
GO_POSITIVE_REGULATION_OF_INTERFERON_BETA_PRODUCTION	30	0.34	2.24	0.002197802	0.008570062

GO_PLATELET_AGGREGATION	35	0.32	2.24	0.003984064	0.008531708
GSE15330_MEGAKARYOCYTE_ERYTHROID_PROGENITOR_VS_PRO_BCELL_UP	179	0.15	2.24	0.002053388	0.008481212
GO_COMPLEX_OF_COLLAGEN_TRIMERS	21	0.41	2.24	0.001941748	0.008431459
BILBAN_B_CLL_LPL_UP	53	0.26	2.24	0.002109705	0.008420204
GSE21546_UNSTIM_VS_ANTI_CD3_STIM_SAP1A_KO_AND_ELK1_KO_DP_THYM(	101	0.19	2.24	0.003937008	0.008422955
GSE3994_WT_VS_PAC1_KO_ACTIVATED_MAST_CELL_DN	160	0.15	2.24	<0.001	0.008426664
GO_REGULATION_OF_MAPK_CASCADE	484	0.09	2.24	0.002070393	0.008430394
GO_TRANSMEMBRANE_RECEPTOR_PROTEIN_TYROSINE_KINASE_SIGNALING_PA	401	0.10	2.24	<0.001	0.008403919
GO_SPINDLE_MIDZONE	24	0.38	2.24	<0.001	0.008392547
REACTOME_TOLL_RECEPTOR_CASCADES	105	0.18	2.24	<0.001	0.008362349
GSE5542_UNTREATED_VS_IFNG_TREATED_EPITHELIAL_CELLS_24H_UP	160	0.15	2.24	<0.001	0.008344306
MARKS_HDAC_TARGETS_DN	13	0.52	2.24	<0.001	0.008311223
GO_REGULATION_OF_ACTIN_FILAMENT_LENGTH	128	0.17	2.24	<0.001	0.008282685
GSE42088_UNINF_VS_LEISHMANIA_INF_DC_2H_DN	149	0.16	2.24	<0.001	0.008286327
FEVR_CTNNB1_TARGETS_UP	520	0.09	2.25	<0.001	0.008200178
GO_POSITIVE_REGULATION_OF_SMOOTH_MUSCLE_CELL_MIGRATION	29	0.35	2.25	<0.001	0.008200899
HARRIS_HYPOXIA	67	0.24	2.25	<0.001	0.008175126
GO_REGULATION_OF_INTERLEUKIN_2_PRODUCTION	37	0.31	2.25	<0.001	0.008128467
GSE3982_CTRL_VS_LPS_48H_DC_DN	154	0.16	2.25	0.003944773	0.008108278
BURTON_ADIPOGENESIS_1	29	0.35	2.25	<0.001	0.008104188
CLASPER_LYMPHATIC_VESSELS_DURING_METASTASIS_DN	34	0.32	2.25	<0.001	0.008078229
MARCHINI_TRABECTEDIN_RESISTANCE_DN	45	0.29	2.25	<0.001	0.008077913
GSE41978_WT_VS_ID2_KO_KLRG1_LOW_EFFECTOR_CD8_TCELL_DN	154	0.16	2.25	0.002087683	0.008047105
GSE29614_CTRL_VS_TIV_FLU_VACCINE_PBMC_2007_DN	99	0.20	2.25	<0.001	0.00798955
LEE_AGING_NEOCORTEX_UP	77	0.22	2.25	<0.001	0.007971937
YGCANTGCR_UNKNOWN	949	0.07	2.25	<0.001	0.007954394
GSE43863_TH1_VS_LY6C_INT_CXCR5POS_MEMORY_CD4_TCELL_UP	178	0.14	2.25	<0.001	0.0079138
GSE36078_UNTREATED_VS_AD5_INF_MOUSE_LUNG_DC_DN	169	0.15	2.25	0.003868472	0.007915253
GO_PROTEIN_SERINE_THREONINE_KINASE_ACTIVITY	376	0.10	2.26	<0.001	0.007719684
GO_NEGATIVE_REGULATION_OF_PROTEIN_BINDING	65	0.24	2.26	0.002061856	0.007661694
TONKS_TARGETS_OF_RUNX1_RUNX1T1_FUSION_HSC_DN	144	0.16	2.26	<0.001	0.007660073
GNF2_PTX3	34	0.33	2.26	<0.001	0.007606696
GARGALOVIC_RESPONSE_TO_OXIDIZED_PHOSPHOLIPIDS_TURQUOISE_UP	68	0.24	2.26	<0.001	0.00757815
GSE15930_STIM_VS_STIM_AND_IFNAB_48H_CD8_T_CELL_DN	162	0.15	2.26	<0.001	0.007582254

FULCHER_INFLAMMATORY_RESPONSE_LECTIN_VS_LPS_DN	363	0.10	2.26	0.002070393	0.007573877
GSE12366_GC_VS_NAIVE_BCELL_UP	164	0.15	2.26	0.002096436	0.007577976
GSE22103_UNSTIM_VS_GMCSF_AND_IFNG_STIM_NEUTROPHIL_DN	167	0.15	2.26	<0.001	0.007525295
KEGG_CHRONIC_MYELOID_LEUKEMIA	70	0.23	2.26	<0.001	0.007524552
REACTOME_METABOLISM_OF_MRNA	168	0.15	2.26	<0.001	0.007529579
GSE20366_EX_VIVO_VS_HOMEOSTATIC_CONVERSION_TREG_DN	158	0.15	2.26	<0.001	0.007521141
GO_CYTOSOLIC_PART	152	0.16	2.26	<0.001	0.007501038
GO_PROTEINACEOUS_EXTRACELLULAR_MATRIX	231	0.13	2.26	<0.001	0.00750414
GO_EXTRINSIC_APOPTOTIC_SIGNALING_PATHWAY_VIA_DEATH_DOMAIN_RECE	29	0.34	2.26	0.004123712	0.007498568
BIOCARTA_PLATELETAPP_PATHWAY	10	0.60	2.26	0.003861004	0.007459964
GSE17812_WT_VS_THPOK_KO_MEMORY_CD8_TCELL_DN	178	0.15	2.26	<0.001	0.007459194
GO_MORPHOGENESIS_OF_AN_ENDOTHELIUM	15	0.48	2.26	0.001956947	0.007459408
AMIT_EGF_RESPONSE_40_MCF10A	18	0.44	2.26	0.001972387	0.007427657
GO_EXTRINSIC_COMPONENT_OF_MEMBRANE	196	0.14	2.26	<0.001	0.007427787
GO_CELLULAR_RESPONSE_TO_MECHANICAL_STIMULUS	67	0.24	2.26	0.001964637	0.007431833
REACTOME_L1CAM_INTERACTIONS	69	0.23	2.26	<0.001	0.007411565
GSE6674_CPG_VS_CPG_AND_ANTI_IGM_STIM_BCELL_DN	176	0.15	2.26	0.003976143	0.007415584
GO_REGULATION_OF_EXTRINSIC_APOPTOTIC_SIGNALING_PATHWAY	129	0.17	2.26	<0.001	0.007417665
HALLMARK_MYC_TARGETS_V2	53	0.27	2.27	0.001960784	0.007402247
GSE6269_FLU_VS_E_COLI_INF_PBMC_UP	135	0.16	2.27	0.002057613	0.007390686
GSE15330_LYMPHOID_MULTIPOTENT_VS_PRO_BCELL_DN	172	0.15	2.27	<0.001	0.007390804
AMIT_EGF_RESPONSE_240_HELA	55	0.26	2.27	<0.001	0.007375334
GSE22432_MULTIPOTENT_VS_COMMON_DC_PROGENITOR_UNTREATED_UP	188	0.14	2.27	<0.001	0.007343106
MODULE_128	73	0.23	2.27	0.004237288	0.007311815
GO_REGULATION_OF_SYNCYTIUM_FORMATION_BY_PLASMA_MEMBRANE_FUS	15	0.48	2.27	<0.001	0.007305967
GO_VACUOLE	931	0.07	2.27	<0.001	0.007309915
PID_CXCR4_PATHWAY	93	0.20	2.27	0.001984127	0.007307995
NIELSEN_LEIOMYOSARCOMA_UP	17	0.45	2.27	<0.001	0.007302132
GSE5463_CTRL_VS_DEXAMETHASONE_TREATED_THYMOCYTE_UP	179	0.15	2.27	0.004016064	0.007294327
REACTOME_PLATELET_AGGREGATION_PLUG_FORMATION	28	0.36	2.27	<0.001	0.007264899
GSE33424_CD161_HIGH_VS_NEG_CD8_TCELL_DN	155	0.15	2.27	<0.001	0.007227397
GSE17721_4_VS_24H_GARDIQUIMOD_BMDC_UP	154	0.16	2.27	<0.001	0.007221484
HALLMARK_COMPLEMENT	151	0.16	2.27	0.001976285	0.007218484
GSE17721_CTRL_VS_POLYIC_8H_BMDC_DN	157	0.15	2.27	0.001934236	0.007223381

GSE40666_UNTREATED_VS_IFNA_STIM_STAT4_KO_EFFECTOR_CD8_TCELL_90M	165	0.15	2.27	<0.001	0.007221368
GSE7348_LPS_VS_TOLERIZED_AND_LPS_STIM_MACROPHAGE_UP	167	0.15	2.27	0.00408998	0.007177815
GO_MACROMOLECULAR_COMPLEX_BINDING	1110	0.06	2.27	<0.001	0.007167862
GSE22886_NAIVE_VS_IGM_MEMORY_BCELL_DN	170	0.15	2.27	<0.001	0.007143997
TONKS_TARGETS_OF_RUNX1_RUNX1T1_FUSION_SUSTAINDED_IN_ERYTHROCYT	36	0.32	2.27	<0.001	0.007102307
GSE7831_UNSTIM_VS_INFLUENZA_STIM_PDC_4H_UP	162	0.15	2.27	0.004149378	0.007073441
GO_ACTIN_FILAMENT_ORGANIZATION	141	0.16	2.27	<0.001	0.007073243
GSE43863_NAIVE_VS_MEMORY_TFH_CD4_TCELL_D150_LCMV_DN	170	0.15	2.28	<0.001	0.007061177
GSE24634_TREG_VS_TCONV_POST_DAY3_IL4_CONVERSION_DN	174	0.15	2.28	0.001934236	0.007001383
GSE37532_VISCERAL_ADIPOSE_TISSUE_VS_LN_DERIVED_PPARG_KO_TREG_CD4	99	0.19	2.28	0.002066116	0.006975279
GO_EPITHELIAL_CELL_DEVELOPMENT	133	0.17	2.28	0.002024292	0.006946175
BIOCARTA_NKT_PATHWAY	12	0.53	2.28	0.001980198	0.006936933
SWEET_LUNG_CANCER_KRAS_UP	392	0.10	2.28	0.003929273	0.006906789
GSE11386_NAIVE_VS_MEMORY_BCELL_UP	165	0.15	2.28	<0.001	0.006896447
GSE21546_WT_VS_SAP1A_KO_AND_ELK1_KO_DP_THYMOCYTES_DN	175	0.15	2.28	<0.001	0.00688218
GO_FC_RECEPTOR_SIGNALING_PATHWAY	169	0.15	2.28	<0.001	0.00687889
GSE24634_IL4_VS_CTRL_TREATED_NAIVE_CD4_TCELL_DAY3_DN	142	0.17	2.28	<0.001	0.006884585
GO_EPITHELIAL_CELL_DIFFERENTIATION	303	0.11	2.28	<0.001	0.006734156
GO_REGULATION_OF_LEUKOCYTE_DIFFERENTIATION	157	0.16	2.28	<0.001	0.006730765
GO_TAXIS	303	0.11	2.28	<0.001	0.006733328
SRF_01	39	0.31	2.28	0.002087683	0.006720875
GSE39382_IL3_VS_IL3_IL33_TREATED_MAST_CELL_DN	158	0.16	2.28	<0.001	0.006716386
GO_ACTIVATION_OF_INNATE_IMMUNE_RESPONSE	174	0.15	2.29	<0.001	0.006634467
KEGG_LEISHMANIA_INFECTION	51	0.27	2.29	0.002083333	0.006569489
OSWALD_HEMATOPOIETIC_STEM_CELL_IN_COLLAGEN_GEL_UP	183	0.15	2.29	<0.001	0.006574964
PID_ATF2_PATHWAY	49	0.28	2.29	<0.001	0.006568313
GO_REGULATION_OF_BLOOD_VESSEL_ENDOTHELIAL_CELL_MIGRATION	45	0.28	2.29	0.001945525	0.006570772
ERB2_UP.V1_UP	151	0.16	2.29	<0.001	0.006543943
CORDENONSI_YAP_CONSERVED_SIGNATURE	57	0.25	2.29	<0.001	0.006539318
VERRECCHIA_RESPONSE_TO_TGFB1_C2	23	0.40	2.29	<0.001	0.006534674
GSE9509_10MIN_VS_30MIN_LPS_AND_IL10_STIM_IL10_KO_MACROPHAGE_DN	131	0.17	2.29	<0.001	0.006536095
GO_REGULATION_OF_COAGULATION	64	0.24	2.29	<0.001	0.00652132
DORN_ADENOVIRUS_INFECTION_24HR_DN	42	0.30	2.29	0.004016064	0.006507505
DAVIES_MULTIPLE_MYELOMA_VS_MGUS_DN	22	0.40	2.29	<0.001	0.006483502

RODWELL_AGING_KIDNEY_NO_BLOOD_UP	167	0.15	2.29	<0.001	0.006488951
REACTOME_ACTIVATION_OF_THE_MRNA_UPON_BINDING_OF_THE_CAP_BINDI	42	0.30	2.29	0.001941748	0.006476122
GO_POSITIVE_REGULATION_OF_CELL_PROJECTION_ORGANIZATION	249	0.12	2.29	<0.001	0.006416488
GSE17721_PAM3CSK4_VS_GADIQUIMOD_12H_BMDC_DN	168	0.15	2.29	0.001976285	0.006390334
KEGG_ECM_RECEPTOR_INTERACTION	70	0.24	2.29	<0.001	0.006381462
ZHOU_CELL_CYCLE_GENES_IN_IR_RESPONSE_6HR	75	0.22	2.29	<0.001	0.006383807
TGCCAAR_NF1_Q6	1065	0.06	2.29	<0.001	0.006363678
GO_CHONDROITIN_SULFATE_BIOSYNTHETIC_PROCESS	20	0.43	2.29	<0.001	0.006364963
GO_REGULATION_OF_MICROTUBULE_BASED_PROCESS	206	0.14	2.29	<0.001	0.006369319
REACTOME_GPVI_MEDIATED_ACTIVATION_CASCADE	30	0.34	2.29	<0.001	0.006370637
GSE1448_ANTI_VALPHA2_VS_VBETA5_DP_THYMOCYTE_DN	158	0.16	2.29	0.001941748	0.006354439
TONKS_TARGETS_OF_RUNX1_RUNX1T1_FUSION_ERYTHROCYTE_UP	125	0.18	2.30	0.001908397	0.006308517
GO_STEM_CELL_DIFFERENTIATION	129	0.17	2.30	<0.001	0.006313873
GSE22886_DAY1_VS_DAY7_MONOCYTE_IN_CULTURE_UP	162	0.16	2.30	<0.001	0.006289492
MARZEC_IL2_SIGNALING_UP	90	0.21	2.30	0.006097561	0.006293801
TGGTGCT_MIR29A_MIR29B_MIR29C	425	0.10	2.30	0.002096436	0.006250809
FOSTER_TOLERANT_MACROPHAGE_UP	128	0.18	2.30	0.002105263	0.006246865
GSE9988_LOW_LPS_VS_ANTI_TREM1_AND_LPS_MONOCYTE_DN	149	0.16	2.30	<0.001	0.006251162
DACOSTA_UV_RESPONSE_VIA_ERCC3_TTD_UP	58	0.25	2.30	<0.001	0.006254426
KEGG_PATHOGENIC_ESCHERICHIA_COLI_INFECTION	47	0.28	2.30	<0.001	0.006249455
GO_NEGATIVE_REGULATION_OF_CYTOKINE_PRODUCTION	144	0.16	2.30	<0.001	0.006239271
GSE25088_WT_VS_STAT6_KO_MACROPHAGE_IL4_STIM_DN	147	0.16	2.30	0.002066116	0.006230102
BRUECKNER_TARGETS_OF_MIRLET7A3_DN	58	0.25	2.30	<0.001	0.006228189
GO_POSITIVE_REGULATION_OF_ORGANELLE_ORGANIZATION	469	0.09	2.30	0.00409836	0.00620346
GSE7509_UNSTIM_VS_FCGRIIB_STIM_DC_UP	138	0.17	2.30	<0.001	0.006196335
GRANDVAUX_IRF3_TARGETS_UP	11	0.56	2.30	<0.001	0.006193306
GSE46468_LUNG_INNATE_LYMPHOID_CELL_VS_SPLEEN_CD4_TCELL_DN	178	0.15	2.30	<0.001	0.006189316
GO_CELL_ADHESION_MEDIATED_BY_INTEGRIN	12	0.54	2.30	<0.001	0.006112362
MODULE_524	28	0.37	2.30	<0.001	0.006052031
REACTOME_INTERFERON_ALPHA_BETA_SIGNALING	37	0.32	2.30	<0.001	0.006053052
BAKER_HEMATOPOIESIS_STAT3_TARGETS	16	0.47	2.30	0.002053388	0.006044725
GSE12366_GC_VS_MEMORY_BCELL_UP	157	0.16	2.30	<0.001	0.006023942
GSE19923_HEB_KO_VS_HEB_AND_E2A_KO_DP_THYMOCYTE_DN	163	0.15	2.31	<0.001	0.005994625
GSE11961_MARGINAL_ZONE_BCELL_VS_GERMINAL_CENTER_BCELL_DAY7_DN	164	0.15	2.31	<0.001	0.005986244

GO_NUCLEOSIDE_TRIPHOSPHATASE_REGULATOR_ACTIVITY	283	0.12	2.31	<0.001	0.005968399
MORF_NME2	124	0.18	2.31	<0.001	0.005955814
GSE1740_UNSTIM_VS_IFNA_STIMULATED_MCSF_DERIVED_MACROPHAGE_DN	119	0.18	2.31	0.001912046	0.005956798
SWEET_LUNG_CANCER_KRAS_DN	377	0.10	2.31	<0.001	0.005959876
BOYAULT_LIVER_CANCER_SUBCLASS_G5_DN	21	0.42	2.31	<0.001	0.005954543
GSE15330_HSC_VS_LYMPHOID_PRIMED_MULTIPOTENT_PROGENITOR_UP	158	0.15	2.31	<0.001	0.005943968
GSE11057_NAIVE_VS_MEMORY_CD4_TCELL_DN	173	0.15	2.31	0.003976143	0.005923896
GSE13522_CTRL_VS_T_CRUZI_Y_STRAIN_INF_SKIN_IFNG_KO_DN	89	0.21	2.31	0.002016129	0.005922737
GO_TRANSFORMING_GROWTH_FACTOR_BETA_RECEPTOR_SIGNALING_PATHW.	77	0.22	2.31	<0.001	0.005914223
GSE42021_CD24INT_VS_CD24LOW_TREG_THYMUS_DN	129	0.18	2.31	<0.001	0.005915154
ZWANG_TRANSIENTLY_UP_BY_2ND_EGF_PULSE_ONLY	702	0.08	2.31	0.001972387	0.005893909
VERRECCHIA_RESPONSE_TO_TGFB1_C1	17	0.46	2.31	<0.001	0.005869418
WIERENGA_STAT5A_TARGETS_DN	163	0.16	2.31	<0.001	0.005824812
GREENBAUM_E2A_TARGETS_UP	33	0.34	2.31	0.002066116	0.005805619
GSE19941_IL10_KO_VS_IL10_KO_AND_NFKBP50_KO_LPS_AND_IL10_STIM_MA(	161	0.15	2.31	0.001912046	0.005809642
DIRMEIER_LMP1_RESPONSE_LATE_UP	50	0.28	2.31	<0.001	0.00576383
GO_REGULATION_OF_RESPONSE_TO_CYTOKINE_STIMULUS	111	0.19	2.31	<0.001	0.005713784
GSE14699_DELETIONAL_TOLERANCE_VS_ACTIVATED_CD8_TCELL_UP	159	0.16	2.31	<0.001	0.005712426
GSE5099_CLASSICAL_M1_VS_ALTERNATIVE_M2_MACROPHAGE_UP	139	0.17	2.32	<0.001	0.005672733
GSE37416_CTRL_VS_6H_F_TULARENSIS_LVS_NEUTROPHIL_DN	161	0.16	2.32	<0.001	0.005604206
GSE46242_TH1_VS_ANERGIC_TH1_CD4_TCELL_WITH_EGR2_DELETED_UP	160	0.16	2.32	<0.001	0.005596368
AMIT_DELAYED_EARLY_GENES	17	0.48	2.32	<0.001	0.005590663
GO_CYTOPLASMIC_SIDE_OF_MEMBRANE	131	0.17	2.32	<0.001	0.005590235
MORI_IMMATURE_B_LYMPHOCYTE_DN	85	0.21	2.32	<0.001	0.005558852
GSE24102_GRANULOCYSTIC_MDSC_VS_NEUTROPHIL_DN	169	0.15	2.32	<0.001	0.005552034
ETS1_B	210	0.14	2.32	<0.001	0.005547392
HIRSCH_CELLULAR_TRANSFORMATION_SIGNATURE_UP	206	0.14	2.32	<0.001	0.005548024
GSE17721_PAM3CSK4_VS_CPG_24H_BMDC_DN	172	0.15	2.32	<0.001	0.005539011
GSE29618_BCELL_VS_PDC_DAY7_FLU_VACCINE_UP	154	0.16	2.32	<0.001	0.005518195
PID_ILK_PATHWAY	44	0.29	2.32	0.002016129	0.00549731
IL15_UP.V1_UP	126	0.18	2.32	0.002008032	0.005477426
GSE14699_NAIVE_VS_DELETIONAL_TOLERANCE_CD8_TCELL_DN	163	0.16	2.32	<0.001	0.005461804
GSE26890_CXCR1_NEG_VS_POS_EFFECTOR_CD8_TCELL_UP	176	0.15	2.32	<0.001	0.005466663
PID_AURORA_A_PATHWAY	30	0.35	2.32	<0.001	0.005441439

KOKKINAKIS_METHIONINE_DEPRIVATION_48HR_UP	115	0.19	2.32	<0.001	0.00543117
GSE17721_CTRL_VS_LPS_1H_BMDC_DN	137	0.17	2.32	<0.001	0.005436015
GSE10325_CD4_TCELL_VS_LUPUS_CD4_TCELL_DN	147	0.17	2.33	<0.001	0.00541502
GHO_ATF5_TARGETS_DN	14	0.50	2.33	<0.001	0.005378794
FUJII_YBX1_TARGETS_DN	174	0.15	2.33	<0.001	0.005377089
GSE27859_MACROPHAGE_VS_CD11C_INT_F480_INT_DC_UP	137	0.17	2.33	<0.001	0.005363455
GSE4984_UNTREATED_VS_GALECTIN1_TREATED_DC_UP	152	0.16	2.33	<0.001	0.005360683
GSE6259_FLT3L_INDUCED_VS_WT_SPLENIC_DC_33D1_POS_UP	148	0.17	2.33	<0.001	0.00531894
TRACEY_RESISTANCE_TO_IFNA2_DN	28	0.37	2.33	<0.001	0.005305293
GSE43863_TH1_VS_TFH_MEMORY_CD4_TCELL_UP	184	0.15	2.33	<0.001	0.00529592
REACTOME_INFLUENZA_LIFE_CYCLE	97	0.20	2.33	0.001953125	0.005276834
UROSEVIC_RESPONSE_TO_IMIQUIMOD	15	0.50	2.33	<0.001	0.00524902
GSE24634_TREG_VS_TCONV_POST_DAY5_IL4_CONVERSION_DN	163	0.16	2.33	<0.001	0.005181887
GSE17721_CTRL_VS_PAM3CSK4_1H_BMDC_DN	139	0.17	2.33	<0.001	0.005171246
GO_RIBOSOME_BIOGENESIS	253	0.13	2.33	<0.001	0.005143214
GO_REGULATION_OF_MYELOID_LEUKOCYTE_DIFFERENTIATION	74	0.23	2.33	<0.001	0.00514022
GSE36392_TYPE_2_MYELOID_VS_NEUTROPHIL_IL25_TREATED_LUNG_UP	167	0.15	2.33	<0.001	0.005121946
HESS_TARGETS_OF_HOXA9_AND_MEIS1_DN	63	0.25	2.33	<0.001	0.005119992
GSE25088_WT_VS_STAT6_KO_MACROPHAGE_DN	157	0.16	2.33	0.001980198	0.00511145
KOBAYASHI_EGFR_SIGNALING_6HR_DN	14	0.52	2.34	<0.001	0.005099613
GSE42724_NAIVE_VS_B1_BCELL_UP	164	0.16	2.34	<0.001	0.005064836
GO_ACTIVATION_OF_PROTEIN_KINASE_ACTIVITY	216	0.14	2.34	<0.001	0.005068339
GO_NEGATIVE_REGULATION_OF_CELLULAR_COMPONENT_ORGANIZATION	568	0.09	2.34	<0.001	0.00506636
GSE40068_BCL6_POS_VS_NEG_CXCR5_POS_TFH_UP	162	0.16	2.34	<0.001	0.005066551
GSE360_L_DONOVANI_VS_B_MALAYI_HIGH_DOSE_MAC_DN	170	0.15	2.34	0.001890359	0.005061253
LU_TUMOR_ANGIOGENESIS_UP	24	0.40	2.34	<0.001	0.00502074
DUTERTRE_ESTRADIOL_RESPONSE_24HR_DN	437	0.10	2.34	<0.001	0.005003227
GO_ENDOLYSOSOME	14	0.51	2.34	<0.001	0.005007796
GSE5589_UNSTIM_VS_45MIN_LPS_STIM_MACROPHAGE_UP	175	0.15	2.34	<0.001	0.004977058
GO_RESPONSE_TO_DSRNA	46	0.29	2.34	0.001964637	0.004962736
GO_POSITIVE_REGULATION_OF_INTERLEUKIN_6_PRODUCTION	51	0.27	2.34	<0.001	0.004967282
GSE3039_ALPHAALPHA_VS_ALPHABETA_CD8_TCELL_UP	164	0.16	2.34	<0.001	0.004949658
GSE24142_DN2_VS_DN3_THYMOCYTE_FETAL_UP	172	0.15	2.34	<0.001	0.004941963
GSE9650_NAIVE_VS_MEMORY_CD8_TCELL_DN	160	0.16	2.34	<0.001	0.004940967

GCM_TPT1	44	0.30	2.34	<0.001	0.004938859
GO_EXTERNAL_SIDE_OF_PLASMA_MEMBRANE	143	0.17	2.34	<0.001	0.00492117
WIERENGA_STAT5A_TARGETS_GROUP2	40	0.32	2.34	<0.001	0.004911193
GSE9650_NAIVE_VS_EFF_CD8_TCELL_DN	164	0.16	2.34	<0.001	0.004906789
GSE360_L_DONOVANI_VS_M_TUBERCULOSIS_MAC_DN	137	0.17	2.34	0.004024145	0.004857718
GSE34179_THPOK_KO_VS_WT_VA14I_NKTCELL_DN	147	0.17	2.35	<0.001	0.004829885
GNF2_PAK2	27	0.38	2.35	0.001964637	0.00483323
GO_MEMBRANE_MICRODOMAIN	239	0.13	2.35	<0.001	0.004837701
GSE9960_GRAM_NEG_VS_GRAM_POS_SEPSIS_PBMC_UP	150	0.17	2.35	<0.001	0.004827675
GO_TRANSMEMBRANE_RECEPTOR_PROTEIN_SERINE_THREONINE_KINASE_SIGN	140	0.17	2.35	<0.001	0.004764951
GNF2_RRM1	82	0.22	2.35	0.003875969	0.004759301
GO_MYELOID_LEUKOCYTE_MIGRATION	62	0.26	2.35	<0.001	0.004760346
REACTOME_TRAFFICKING_AND_PROCESSING_OF_ENDOSOMAL_TLR	10	0.60	2.35	0.002028398	0.004744533
TENEDINI_MEGAKARYOCYTE_MARKERS	54	0.27	2.35	0.002053388	0.004724216
GSE25123_WT_VS_PPARG_KO_MACROPHAGE_IL4_STIM_DN	159	0.16	2.35	<0.001	0.00471962
GSE42724_MEMORY_VS_B1_BCELL_DN	150	0.17	2.35	<0.001	0.004699186
MANALO_HYPOXIA_UP	189	0.15	2.35	<0.001	0.004700183
DAUER_STAT3_TARGETS_UP	43	0.30	2.35	<0.001	0.004698894
RAGHAVACHARI_PLATELET_SPECIFIC_GENES	61	0.25	2.35	<0.001	0.004695406
GSE339_EX_VIVO_VS_IN_CULTURE_CD8POS_DC_DN	174	0.15	2.35	<0.001	0.004694132
MODULE_122	114	0.19	2.35	<0.001	0.004687212
GSE13485_DAY3_VS_DAY21_YF17D_VACCINE_PBMC_UP	172	0.15	2.35	<0.001	0.004667838
GSE2585_CD80_HIGH_VS_LOW_AIRE_KO_MTEC_DN	125	0.18	2.35	0.002070393	0.004665435
REACTOME_NCAM1_INTERACTIONS	30	0.36	2.36	<0.001	0.004600498
REACTOME_INNATE_IMMUNE_SYSTEM	182	0.15	2.36	0.002012072	0.004538963
NABA_BASEMENT_MEMBRANES	33	0.35	2.36	0.001992032	0.004540952
PID_TGFBR_PATHWAY	52	0.28	2.36	<0.001	0.004531562
GSE9988_LPS_VS_CTRL_TREATED_MONOCYTE_UP	132	0.18	2.36	<0.001	0.004534697
KEGG_TOLL_LIKE_RECEPTOR_SIGNALING_PATHWAY	81	0.23	2.36	<0.001	0.004538975
BASSO_HAIRY_CELL_LEUKEMIA_UP	69	0.24	2.36	<0.001	0.004539825
GO_PROTEIN_LOCALIZATION_TO_ENDOPLASMIC_RETICULUM	88	0.22	2.36	<0.001	0.004498321
GO_MITOTIC_CYTOKINESIS	30	0.36	2.36	<0.001	0.004472875
BURTON_ADIPOGENESIS_8	76	0.23	2.36	<0.001	0.004475961
BORLAK_LIVER_CANCER_EGF_UP	45	0.30	2.36	<0.001	0.004419474

GSE9960_HEALTHY_VS_GRAM_NEG_SEPSIS_PBMC_DN	163	0.16	2.36	0.002016129	0.004416775
MORI_MATURE_B_LYMPHOCYTE_UP	76	0.23	2.36	0.001960784	0.004387673
GSE21927_SPLEEN_VS_BONE_MARROW_MONOCYTE_BALBC_DN	139	0.18	2.36	<0.001	0.004360789
HALLMARK_ANGIOGENESIS	29	0.37	2.37	<0.001	0.004325818
GSE17974_IL4_AND_ANTI_IL12_VS_UNTREATED_72H_ACT_CD4_TCELL_DN	149	0.17	2.37	0.002004008	0.004324163
GSE24671_CTRL_VS_BAKIMULC_INFECTED_MOUSE_SPLENOCYTES_DN	130	0.18	2.37	<0.001	0.004325976
GO_REGULATION_OF_INTERFERON_ALPHA_PRODUCTION	18	0.47	2.37	<0.001	0.004317419
GSE43955_TH0_VS_TGFB_IL6_IL23_TH17_ACT_CD4_TCELL_60H_DN	163	0.16	2.37	<0.001	0.004303091
GSE17721_CTRL_VS_POLYIC_4H_BMDC_DN	163	0.16	2.37	<0.001	0.004306044
GO_POSITIVE_REGULATION_OF_HEMOPOIESIS	113	0.19	2.37	<0.001	0.0043044
GSE32986_CURDLAN_LOWDOSE_VS_GMCSF_AND_CURDLAN_LOWDOSE_STIM_	174	0.16	2.37	<0.001	0.004280798
GSE46606_IRF4HIGH_VS_WT_CD40L_IL2_IL5_DAY3_STIMULATED_BCELL_UP	181	0.15	2.37	<0.001	0.004283741
VILIMAS_NOTCH1_TARGETS_UP	33	0.35	2.37	<0.001	0.00427273
GSE5679_CTRL_VS_PPARG_LIGAND_ROSIGLITAZONE_AND_RARA_AGONIST_AM	155	0.16	2.37	<0.001	0.004239609
PID_RAC1_REG_PATHWAY	35	0.33	2.37	<0.001	0.004221543
GO_SINGLE_ORGANISM_CELLULAR_LOCALIZATION	707	0.08	2.37	<0.001	0.004205847
GSE23568_CTRL_TRANSDUCED_VS_WT_CD8_TCELL_UP	182	0.15	2.37	<0.001	0.004200595
GSE40274_CTRL_VS_XBP1_TRANSDUCED_ACTIVATED_CD4_TCELL_UP	143	0.17	2.37	<0.001	0.004202322
GO_POSITIVE_REGULATION_OF_CYTOSKELETON_ORGANIZATION	146	0.17	2.37	<0.001	0.004150384
GO_LAMELLIPODIUM_ASSEMBLY	28	0.37	2.37	<0.001	0.004113456
GO_EPIBOLY	14	0.52	2.38	<0.001	0.004115094
GSE21774_CD62L_POS_CD56_BRIGHT_VS_CD62L_NEG_CD56_DIM_NK_CELL_UI	144	0.17	2.38	<0.001	0.004115568
GO_CELL_CORTEX	199	0.14	2.38	0.00204918	0.00410665
ZHANG_PROLIFERATING_VS_QUIESCENT	42	0.32	2.38	<0.001	0.004103599
GSE36527_CD62L_HIGH_VS_CD62L_LOW_TREG_CD69_NEG_KLRG1_NEG_UP	175	0.15	2.38	<0.001	0.004104055
GSE11057_NAIVE_VS_EFF_MEMORY_CD4_TCELL_DN	158	0.16	2.38	<0.001	0.004100973
REACTOME_FORMATION_OF_THE_TERNARY_COMPLEX_AND_SUBSEQUENTLY_	35	0.34	2.38	<0.001	0.004101431
GSE18893_TCONV_VS_TREG_24H_CULTURE_DN	169	0.16	2.38	<0.001	0.004077143
GSE5679_PPARG_LIGAND_ROSIGLITAZONE_VS_RARA_AGONIST_AM580_TREAT	168	0.16	2.38	<0.001	0.004078758
TAKEDA_TARGETS_OF_NUP98_HOXA9_FUSION_6HR_DN	33	0.35	2.38	<0.001	0.004042644
GO_POSITIVE_REGULATION_OF_INTERFERON_ALPHA_PRODUCTION	16	0.50	2.38	<0.001	0.004039524
GO_REGULATION_OF_MYELOID_CELL_DIFFERENTIATION	127	0.18	2.38	<0.001	0.004034064
XU_HGF_TARGETS_INDUCED_BY_AKT1_6HR	16	0.49	2.38	<0.001	0.004003671
GO_REGULATION_OF_ORGANELLE_ORGANIZATION	972	0.07	2.38	<0.001	0.003989863

GSE41867_DAY6_EFFECTOR_VS_DAY30_EXHAUSTED_CD8_TCELL_LCMV_CLONE	159	0.16	2.38	0.00204499	0.0039724
GO_POSITIVE_REGULATION_OF_CYTOKINE_BIOSYNTHETIC_PROCESS	42	0.31	2.38	0.001879699	0.003943012
GO_INTRACELLULAR_VESICLE	901	0.07	2.38	<0.001	0.003880264
GSE8835_CD4_VS_CD8_TCELL_CLL_PATIENT_UP	142	0.17	2.39	<0.001	0.003846017
REACTOME_METABOLISM_OF_PROTEINS	331	0.11	2.39	<0.001	0.003843867
GO_POSITIVE_REGULATION_OF_PROTEIN_MODIFICATION_PROCESS	850	0.07	2.39	<0.001	0.003834509
GSE9988_LPS_VS_LPS_AND_ANTI_TREM1_MONOCYTE_DN	146	0.17	2.39	<0.001	0.003818076
PID_TCR_PATHWAY	55	0.27	2.39	0.002057613	0.003795551
MORI_PRE_BI_LYMPHOCYTE_UP	69	0.25	2.39	<0.001	0.003796903
PTEN_DN.V2_UP	82	0.22	2.39	0.002079002	0.003795889
GSE36078_WT_VS_IL1R_KO_LUNG_DC_AFTER_AD5_T425A_HEXON_INF_UP	160	0.16	2.39	<0.001	0.00379607
MODULE_241	60	0.27	2.39	<0.001	0.003682476
ESC_V6.5_UP_EARLY.V1_DN	127	0.18	2.39	<0.001	0.003686129
GSE21360_PRIMARY_VS_TERTIARY_MEMORY_CD8_TCELL_DN	161	0.16	2.39	<0.001	0.003651324
REACTOME_SIGNALING_BY_GPCR	298	0.12	2.39	<0.001	0.003654953
GSE41087_WT_VS_FOXP3_MUT_ANTI_CD3_CD28_STIM_CD4_TCELL_UP	150	0.17	2.39	<0.001	0.003652563
GO_CELL_JUNCTION_ASSEMBLY	109	0.19	2.39	<0.001	0.003641754
GO_DEATH_RECEPTOR_BINDING	16	0.49	2.40	<0.001	0.003628501
FARMER_BREAST_CANCER_CLUSTER_1	28	0.38	2.40	<0.001	0.003614001
FIRESTEIN_CTNNB1_PATHWAY	29	0.38	2.40	<0.001	0.003617612
DANG_REGULATED_BY_MYC_DN	223	0.14	2.40	<0.001	0.003611593
GSE12963_UNINF_VS_ENV_AND_NEF_DEFICIENT_HIV1_INF_CD4_TCELL_DN	108	0.20	2.40	<0.001	0.003603069
GNF2_VAV1	28	0.38	2.40	<0.001	0.003590946
GSE22935_UNSTIM_VS_24H_MBOVIS_BCG_STIM_MYD88_KO_MACROPHAGE_[illegible]	139	0.17	2.40	<0.001	0.003587284
GO_ACTIN_FILAMENT_BUNDLE_ORGANIZATION	39	0.32	2.40	<0.001	0.003590886
GSE9988_ANTI_TREM1_VS_LOW_LPS_MONOCYTE_DN	152	0.17	2.40	<0.001	0.003584794
MODULE_46	234	0.13	2.40	0.001912046	0.003576277
GSE21063_WT_VS_NFATC1_KO_8H_ANTI_IGM_STIM_BCELL_UP	173	0.16	2.40	<0.001	0.003540931
GSE3982_NEUTROPHIL_VS_BASOPHIL_UP	148	0.17	2.40	<0.001	0.003517662
GSE23321_CENTRAL_VS_EFFECTOR_MEMORY_CD8_TCELL_UP	168	0.16	2.40	<0.001	0.003495509
RUTELLA_RESPONSE_TO_HGF_VS_CSF2RB_AND_IL4_DN	204	0.14	2.40	<0.001	0.003491725
TAKEDA_TARGETS_OF_NUP98_HOXA9_FUSION_10D_UP	121	0.19	2.40	<0.001	0.003490367
WHITFIELD_CELL_CYCLE_LITERATURE	40	0.32	2.40	<0.001	0.003447358
MODULE_291	53	0.28	2.41	<0.001	0.003415359

PID_PDGFRB_PATHWAY	127	0.19	2.41	<0.001	0.003384522
GO_REGULATION_OF_EPITHELIAL_CELL_MIGRATION	144	0.17	2.41	<0.001	0.003384276
GO_PEPTIDYL_SERINE_MODIFICATION	123	0.19	2.41	<0.001	0.003355799
GSE9650_NAIVE_VS_EFF_CD8_TCELL_UP	182	0.16	2.41	<0.001	0.003343214
GSE40277_EOS_AND_LEF1_TRANSDUCED_VS_GATA1_AND_SATB1_TRANSDUCE	173	0.16	2.41	<0.001	0.003335482
GO_I_KAPPAB_KINASE_NF_KAPPAB_SIGNALING	60	0.26	2.41	0.001934236	0.003337639
BLALOCK_ALZHEIMERS_DISEASE_UP	1380	0.06	2.41	0.001960784	0.003339819
REACTOME_SEMA3A_PAK_DEPENDENT_AXON_REPULSION	14	0.54	2.41	<0.001	0.003340754
GNF2_CD33	41	0.32	2.41	0.002016129	0.003294604
GSE39152_BRAIN_VS_SPLEEN_CD103_NEG_MEMORY_CD8_TCELL_DN	176	0.16	2.41	<0.001	0.003271936
AMIT_EGF_RESPONSE_120_HELA	58	0.27	2.41	<0.001	0.003248003
ONKEN_UVEAL_MELANOMA_UP	681	0.08	2.41	0.001923077	0.003251334
GO_MULTICELLULAR_ORGANISM_METABOLIC_PROCESS	66	0.25	2.41	<0.001	0.003233546
GSE5542_IFNA_VS_IFNA_AND_IFNG_TREATED_EPITHELIAL_CELLS_24H_UP	156	0.17	2.41	<0.001	0.003231901
BOWIE_RESPONSE_TO_TAMOXIFEN	14	0.54	2.42	<0.001	0.003206577
GO_DEFENSE_RESPONSE_TO_OTHER_ORGANISM	278	0.13	2.42	<0.001	0.003201159
GSE18791_CTRL_VS_NEWCASTLE_VIRUS_DC_18H_DN	124	0.18	2.42	<0.001	0.00319071
GSE24634_TEFF_VS_TCONV_DAY7_IN_CULTURE_UP	165	0.16	2.42	0.001988072	0.003194003
GO_CYTOPLASMIC_REGION	237	0.13	2.42	<0.001	0.003182343
GSE22589_HEALTHY_VS_HIV_INFECTED_DC_DN	151	0.17	2.42	<0.001	0.003179433
GSE3920_UNTREATED_VS_IFNA_TREATED_ENDOTHELIAL_CELL_UP	157	0.16	2.42	<0.001	0.003180204
PID_FOXM1_PATHWAY	37	0.34	2.42	0.002008032	0.003179716
GSE6269_E_COLI_VS_STAPH_AUREUS_INF_PBMC_DN	150	0.17	2.42	<0.001	0.003144174
GSE29618_MONOCYTE_VS_PDC_UP	164	0.16	2.42	0.001872659	0.00314744
GSE22229_RENAL_TRANSPLANT_IMMUNOSUPP_THERAPY_VS_HEALTHY_PBMC_	168	0.16	2.42	<0.001	0.003144405
GSE18791_CTRL_VS_NEWCASTLE_VIRUS_DC_14H_DN	120	0.19	2.42	<0.001	0.003136411
CSR_EARLY_UP.V1_UP	144	0.17	2.42	0.001956947	0.003138437
GO_CELLULAR_RESPONSE_TO_VASCULAR_ENDOTHELIAL_GROWTH_FACTOR_ST	29	0.39	2.42	<0.001	0.003132866
GO_MAP_KINASE_PHOSPHATASE_ACTIVITY	10	0.62	2.42	<0.001	0.003128539
GSE7460_FOXP3_MUT_VS_WT_ACT_WITH_TGFB_TCONV_UP	148	0.17	2.42	<0.001	0.003131809
CROONQUIST_STROMAL_STIMULATION_UP	48	0.29	2.42	<0.001	0.003127487
GO_SIGNAL_TRANSDUCER_ACTIVITY	751	0.08	2.42	<0.001	0.003102942
GSE25087_TREG_VS_TCONV_ADULT_UP	155	0.17	2.42	0.002040816	0.003101126
PANGAS_TUMOR_SUPPRESSION_BY_SMAD1_AND_SMAD5_UP	117	0.19	2.42	0.001968504	0.003095566

GO_POSITIVE_REGULATION_OF_CELLULAR_COMPONENT_ORGANIZATION	905	0.07	2.42	0.001964637	0.003096289
GO_REGULATION_OF_I_KAPPAB_KINASE_NF_KAPPAB_SIGNALING	190	0.15	2.42	<0.001	0.003090609
GSE4748_CTRL_VS_CYANOBACTERIUM_LPSLIKE_STIM_DC_3H_DN	178	0.15	2.42	<0.001	0.003091325
SNIJDERS_AMPLIFIED_IN_HEAD_AND_NECK_TUMORS	34	0.35	2.43	<0.001	0.003074126
GSE37532_WT_VS_PPARG_KO_LN_TREG_UP	159	0.17	2.43	<0.001	0.003073575
WORSCHECH_TUMOR_EVASION_AND_TOLEROGENICITY_UP	18	0.48	2.43	<0.001	0.003051212
GSE22886_NAIVE_VS_IGG_IGA_MEMORY_BCELL_DN	163	0.16	2.43	<0.001	0.003051876
GSE36078_WT_VS_IL1R_KO_LUNG_DC_AFTER_AD5_INF_UP	154	0.17	2.43	<0.001	0.003038441
GSE15735_2H_VS_12H_HDAC_INHIBITOR_TREATED_CD4_TCELL_DN	177	0.16	2.43	<0.001	0.003024992
MORF_JUND	45	0.30	2.43	<0.001	0.002992256
GSE9988_ANTI_TREM1_VS_ANTI_TREM1_AND_LPS_MONOCYTE_UP	176	0.15	2.43	<0.001	0.002981313
VERHAAK_AML_WITH_NPM1_MUTATED_UP	126	0.18	2.43	<0.001	0.002984481
GO_REGULATION_OF_PROTEIN_MODIFICATION_PROCESS	1298	0.06	2.43	<0.001	0.002981212
SHETH_LIVER_CANCER_VS_TXNIP_LOSS_PAM1	181	0.16	2.43	<0.001	0.002971466
GO_EPHRIN_RECEPTOR_SIGNALING_PATHWAY	70	0.25	2.43	<0.001	0.002959101
REACTOME_IMMUNE_SYSTEM	700	0.08	2.43	<0.001	0.002960973
GSE17721_POLYIC_VS_CPG_6H_BMDC_DN	162	0.16	2.43	<0.001	0.002956389
KEGG_CHEMOKINE_SIGNALING_PATHWAY	139	0.18	2.43	0.002136752	0.002946611
BIOCARTA_RANKL_PATHWAY	11	0.61	2.43	<0.001	0.002904434
GO_MOLECULAR_FUNCTION_REGULATOR	976	0.07	2.43	<0.001	0.002904955
GSE19401_PLN_VS_PEYERS_PATCH_FOLLICULAR_DC_DN	174	0.16	2.43	<0.001	0.002904178
GSE15330_MEGAKARYOCYTE_ERYTHROID_VS_GRANULOCYTE_MONOCYTE_PRC	179	0.15	2.43	<0.001	0.002900779
GO_REGULATION_OF_MICROTUBULE_POLYMERIZATION_OR_DEPOLYMERIZATIC	155	0.17	2.43	<0.001	0.002903898
GO_MULTICELLULAR_ORGANISMAL_MACROMOLECULE_METABOLIC_PROCESS	57	0.28	2.43	<0.001	0.00290573
GO_REGULATION_OF_MAP_KINASE_ACTIVITY	258	0.13	2.44	<0.001	0.002902354
GSE13522_WT_VS_IFNAR_KO_SKING_T_CRUZI_Y_STRAIN_INF_UP	156	0.17	2.44	<0.001	0.002888483
GSE3039_ALPHAALPHA_CD8_TCELL_VS_B2_BCELL_DN	175	0.16	2.44	0.003992016	0.00284713
JECHLINGER_EPITHELIAL_TO_MESENCHYMAL_TRANSITION_UP	61	0.26	2.44	<0.001	0.002788811
GSE360_DC_VS_MAC_B_MALAYI_HIGH_DOSE_DN	154	0.17	2.44	<0.001	0.002789213
GSE40666_WT_VS_STAT1_KO_CD8_TCELL_DN	170	0.16	2.44	<0.001	0.002790923
MAHADEVAN_RESPONSE_TO_MP470_DN	17	0.50	2.44	<0.001	0.002786092
GSE37416_12H_VS_48H_F_TULARENSIS_LVS_NEUTROPHIL_UP	172	0.16	2.44	<0.001	0.002741814
GO_RRNA_METABOLIC_PROCESS	207	0.15	2.44	<0.001	0.002742166
WHITFIELD_CELL_CYCLE_G2	154	0.17	2.44	<0.001	0.002741202

GO_RESPONSE_TO_TYPE_I_INTERFERON	40	0.33	2.44	<0.001	0.002737616
GSE2706_UNSTIM_VS_8H_LPS_DC_DN	147	0.18	2.44	<0.001	0.002740602
REACTOME_EXTRACELLULAR_MATRIX_ORGANIZATION	58	0.28	2.44	<0.001	0.002706592
GO_REGULATION_OF_CELL_PROJECTION_ORGANIZATION	436	0.10	2.45	<0.001	0.002702986
GRAESSMANN_APOPTOSIS_BY_DOXORUBICIN_UP	961	0.07	2.45	<0.001	0.002690068
GSE34515_CD16_NEG_MONOCYTE_VS_DC_UP	162	0.17	2.45	0.001980198	0.00267974
TONKS_TARGETS_OF_RUNX1_RUNX1T1_FUSION_HSC_UP	152	0.17	2.45	<0.001	0.00265874
GO_ENDOTHELIAL_CELL_DEVELOPMENT	40	0.33	2.45	<0.001	0.002640423
GSE21546_ELK1_KO_VS_SAP1A_KO_AND_ELK1_KO_ANTI_CD3_STIM_DP_THYM	157	0.17	2.45	<0.001	0.002591438
GSE33162_HDAC3_KO_VS_HDAC3_KO_MACROPHAGE_UP	154	0.17	2.45	<0.001	0.002584989
MODULE_170	76	0.23	2.45	<0.001	0.002566582
GO_TISSUE_MORPHOGENESIS	384	0.11	2.45	<0.001	0.002538804
GSE9650_EFFECTOR_VS_MEMORY_CD8_TCELL_DN	177	0.16	2.45	0.002053388	0.002522942
GSE1791_CTRL_VS_NEUROMEDINU_IN_T_CELL_LINE_6H_UP	165	0.16	2.46	<0.001	0.002519041
GO_REGULATION_OF_INNATE_IMMUNE_RESPONSE	274	0.13	2.46	<0.001	0.002512453
FARMER_BREAST_CANCER_CLUSTER_2	31	0.37	2.46	<0.001	0.002499177
NABA_CORE_MATRISOME	173	0.16	2.46	<0.001	0.002493911
GSE45365_NK_CELL_VS_CD8A_DC_UP	171	0.16	2.46	<0.001	0.002477844
GSE21033_1H_VS_12H_POLYIC_STIM_DC_UP	151	0.17	2.46	0.00204499	0.002463151
GO_POSITIVE_REGULATION_OF_TRANSFERASE_ACTIVITY	505	0.10	2.46	<0.001	0.002463202
SAGIV_CD24_TARGETS_DN	40	0.33	2.46	<0.001	0.002448466
GSE27859_MACROPHAGE_VS_DC_UP	139	0.18	2.46	<0.001	0.002447123
SCIAN_INVERSED_TARGETS_OF_TP53_AND_TP73_DN	30	0.37	2.46	<0.001	0.002449854
GSE14415_INDUCED_TREG_VS_FAILED_INDUCED_TREG_DN	150	0.18	2.46	<0.001	0.002437678
GSE41176_WT_VS_TAK1_KO_ANTI_IGM_STIM_BCELL_3H_DN	176	0.16	2.46	<0.001	0.002425554
GSE5679_CTRL_VS_RARA_AGONIST_AM580_TREATED_DC_UP	176	0.16	2.46	<0.001	0.002425543
GSE36476_CTRL_VS_TSST_ACT_72H_MEMORY_CD4_TCELL_OLD_DN	180	0.16	2.46	<0.001	0.002413334
GSE10856_CTRL_VS_TNFRSF6B_IN_MACROPHAGE_UP	138	0.18	2.46	<0.001	0.002407867
GSE360_DC_VS_MAC_M_TUBERCULOSIS_UP	147	0.17	2.46	<0.001	0.00238482
TONKS_TARGETS_OF_RUNX1_RUNX1T1_FUSION_MONOCYTE_UP	169	0.16	2.46	<0.001	0.002384757
EINAV_INTERFERON_SIGNATURE_IN_CANCER	22	0.44	2.47	<0.001	0.002341181
GSE36476_CTRL_VS_TSST_ACT_72H_MEMORY_CD4_TCELL_YOUNG_DN	177	0.16	2.47	<0.001	0.002330204
GSE2197_IMMUNOSUPPRESSIVE_DNA_VS_UNTREATED_IN_DC_UP	166	0.16	2.47	0.002061856	0.002328743
GSE3337_CTRL_VS_4H_IFNG_IN_CD8POS_DC_DN	165	0.16	2.47	<0.001	0.002321815

GSE25123_IL4_VS_IL4_AND_ROSIGLITAZONE_STIM_MACROPHAGE_DAY10_UP	159	0.17	2.47	<0.001	0.002324441
GSE21063_CTRL_VS_ANTI_IGM_STIM_BCELL_NFATC1_KO_16H_UP	144	0.17	2.47	<0.001	0.002317473
GO_NEGATIVE_REGULATION_OF_IMMUNE_SYSTEM_PROCESS	249	0.14	2.47	<0.001	0.002306351
GO_SPROUTING_ANGIOGENESIS	40	0.33	2.47	<0.001	0.00229799
GSE13522_WT_VS_IFNAR_KO_SKIN_DN	147	0.17	2.47	<0.001	0.002296452
GSE39110_UNTREATED_VS_IL2_TREATED_CD8_TCELL_DAY3_POST_IMMUNIZAT	163	0.17	2.47	<0.001	0.002299065
BRUINS_UVC_RESPONSE_VIA_TP53_GROUP_B	417	0.11	2.47	<0.001	0.002298931
PETROVA_PROX1_TARGETS_UP	27	0.39	2.47	<0.001	0.002301552
GSE3982_CTRL_VS_LPS_4H_MAC_DN	159	0.17	2.47	<0.001	0.002277989
HAMAI_APOPTOSIS_VIA_TRAIL_DN	123	0.19	2.47	<0.001	0.00224187
GSE22935_UNSTIM_VS_24H_MBOVIS_BCG_STIM_MYD88_KO_MACROPHAGE_U	177	0.16	2.48	<0.001	0.002179545
YGTCCTTGR_UNKNOWN	388	0.11	2.48	<0.001	0.00216959
GSE21360_NAIVE_VS_QUATERNARY_MEMORY_CD8_TCELL_UP	171	0.16	2.48	<0.001	0.002162385
WHITEFORD_PEDIATRIC_CANCER_MARKERS	105	0.20	2.48	<0.001	0.002160679
GSE30971_WBP7_HET_VS_KO_MACROPHAGE_4H_LPS_STIM_UP	153	0.17	2.48	<0.001	0.002157613
GO_VASCULOGENESIS	50	0.29	2.48	<0.001	0.002125232
KAECH_DAY8_EFF_VS_MEMORY_CD8_TCELL_UP	170	0.17	2.48	<0.001	0.002126258
REACTOME_SEMAPHORIN_INTERACTIONS	62	0.27	2.48	<0.001	0.00211619
GSE2770_IL4_ACT_VS_ACT_CD4_TCELL_2H_DN	173	0.16	2.48	<0.001	0.002082268
GSE46606_UNSTIM_VS_CD40L_IL2_IL5_DAY1_STIMULATED_BCELL_DN	157	0.17	2.49	<0.001	0.002076229
GSE42724_NAIVE_VS_MEMORY_BCELL_DN	171	0.16	2.49	<0.001	0.002053427
GO_REGULATION_OF_ENDOTHELIAL_CELL_MIGRATION	101	0.21	2.49	<0.001	0.002033443
GO_CELL_JUNCTION_ORGANIZATION	150	0.18	2.49	0.002079002	0.00201758
WIELAND_UP_BY_HBV_INFECTION	77	0.24	2.49	<0.001	0.002007297
GSE24142_EARLY_THYMIC_PROGENITOR_VS_DN3_THYMOCYTE_ADULT_UP	172	0.16	2.49	<0.001	0.001994155
GSE17721_PAM3CSK4_VS_CPG_12H_BMDC_DN	167	0.16	2.49	<0.001	0.001969821
GNF2_GLTSCR2	23	0.43	2.49	<0.001	0.001969298
GO_ENDOSOME	622	0.09	2.49	<0.001	0.001950369
GO_NEGATIVE_REGULATION_OF_MYELOID_LEUKOCYTE_DIFFERENTIATION	32	0.37	2.49	<0.001	0.001952648
GO_NEGATIVE_REGULATION_OF_MYELOID_CELL_DIFFERENTIATION	55	0.29	2.50	<0.001	0.001946474
DUTERTRE_ESTRADIOL_RESPONSE_6HR_UP	198	0.15	2.50	<0.001	0.001936001
GO_POSITIVE_REGULATION_OF_PRI_MIRNA_TRANSCRIPTION_FROM_RNA_POL	10	0.65	2.50	<0.001	0.001918436
REACTOME_GLYCOSAMINOGLYCAN_METABOLISM	80	0.24	2.50	<0.001	0.001912172
GO_MUCOPOLYSACCHARIDE_METABOLIC_PROCESS	81	0.23	2.50	<0.001	0.001891741

FOURNIER_ACINAR_DEVELOPMENT_LATE_DN	17	0.50	2.50	<0.001	0.001888311
GSE34156_UNTREATED_VS_6H_NOD2_AND_TLR1_TLR2_LIGAND_TREATED_MC	133	0.19	2.50	<0.001	0.001877672
HONRADO_BREAST_CANCER_BRCA1_VS_BRCA2	15	0.53	2.50	<0.001	0.001871259
GSE18791_CTRL_VS_NEWCASTLE_VIRUS_DC_12H_DN	142	0.18	2.50	<0.001	0.00187204
GO_NEGATIVE_REGULATION_OF_PROTEIN_COMPLEX_DISASSEMBLY	147	0.18	2.50	<0.001	0.001871387
KAECH_DAY15_EFF_VS_MEMORY_CD8_TCELL_DN	176	0.16	2.50	0.002028398	0.001863582
GSE5679_CTRL_VS_PPARG_LIGAND_ROSIGLITAZONE_AND_RARA_AGONIST_AM	174	0.17	2.50	0.001926782	0.00186579
GO_CELL_CELL_ADHESION	368	0.12	2.50	<0.001	0.001860815
GSE6259_CD4_TCELL_VS_CD8_TCELL_UP	160	0.17	2.51	<0.001	0.001860134
GSE27241_CTRL_VS_DIGOXIN_TREATED_CD4_TCELL_IN_TH17_POLARIZING_CO	153	0.17	2.51	0.002040816	0.001858029
GO_REGULATION_OF_INTERLEUKIN_6_PRODUCTION	73	0.25	2.51	<0.001	0.00186024
TBK1.DF_UP	239	0.14	2.51	<0.001	0.001852412
DAZARD_UV_RESPONSE_CLUSTER_G1	54	0.29	2.51	<0.001	0.001848846
GSE15930_STIM_VS_STIM_AND_IL-12_48H_CD8_T_CELL_UP	183	0.16	2.51	<0.001	0.001849598
GSE22886_NAIVE_BCELL_VS_NEUTROPHIL_DN	147	0.18	2.51	<0.001	0.001848918
GSE46606_DAY1_VS_DAY3_CD40L_IL2_IL5_STIMULATED_IRF4HIGH_BCELL_DN	164	0.17	2.51	<0.001	0.001846766
GSE9988_ANTI_TREM1_AND_LPS_VS_VEHICLE_TREATED_MONOCYTES_DN	178	0.16	2.51	<0.001	0.001841751
GSE3982_NEUTROPHIL_VS_BCELL_UP	139	0.18	2.51	<0.001	0.001826527
GSE9960_HEALTHY_VS_GRAM_POS_SEPSIS_PBMC_DN	146	0.18	2.51	<0.001	0.001812717
GO_REGULATION_OF_MONOCYTE_DIFFERENTIATION	11	0.62	2.51	<0.001	0.001814899
GSE17974_0H_VS_24H_IN_VITRO_ACT_CD4_TCELL_DN	154	0.17	2.51	<0.001	0.001812742
GSE22196_HEALTHY_VS_OBESE_MOUSE_SKIN_GAMMADELTA_TCELL_DN	175	0.17	2.51	<0.001	0.001814929
GO_EXTRACELLULAR_MATRIX_COMPONENT	108	0.20	2.51	<0.001	0.001793719
PHONG_TNF_RESPONSE_VIA_P38_PARTIAL	137	0.19	2.51	<0.001	0.001795888
GO_POSITIVE_REGULATION_OF_LEUKOCYTE_DIFFERENTIATION	89	0.22	2.52	<0.001	0.001758521
HORIUCHI_WTAP_TARGETS_DN	262	0.13	2.52	<0.001	0.001754824
GO_CYTOSKELETON_DEPENDENT_CYTOKINESIS	36	0.35	2.52	<0.001	0.001755497
GSE12845_IGD_POS_BLOOD_VS_PRE_GC_TONSIL_BCELL_DN	175	0.16	2.52	<0.001	0.001744421
SHEDDEN_LUNG_CANCER_POOR_SURVIVAL_A6	381	0.11	2.52	<0.001	0.001745061
GO_RESPONSE_TO_GROWTH_FACTOR	372	0.11	2.52	<0.001	0.001736883
GSE11961_FOLLICULAR_BCELL_VS_MEMORY_BCELL_DAY7_UP	160	0.17	2.52	<0.001	0.001731605
GSE339_EX_VIVO_VS_IN_CULTURE_CD4CD8DN_DC_DN	157	0.17	2.52	<0.001	0.001710116
GSE37416_CTRL_VS_12H_F_TULARENSIS_LVS_NEUTROPHIL_DN	164	0.17	2.52	<0.001	0.001712207
GSE40666_WT_VS_STAT4_KO_CD8_TCELL_WITH_IFNA_STIM_90MIN_DN	170	0.16	2.52	<0.001	0.001712834

GO_EARLY_ENDOSOME	236	0.14	2.52	<0.001	0.00170304
GSE36826_WT_VS_IL1R_KO_SKIN_DN	162	0.17	2.52	<0.001	0.001696169
WU_CELL_MIGRATION	130	0.19	2.52	<0.001	0.001693817
BIOCARTA_TID_PATHWAY	17	0.50	2.53	<0.001	0.001667625
GO_PROTEIN_TARGETING	323	0.12	2.53	0.004008016	0.001665198
REACTOME_GPCR_DOWNSTREAM_SIGNALING	230	0.14	2.53	<0.001	0.001659795
GNF2_TYK2	31	0.38	2.53	<0.001	0.001652818
GO_NEGATIVE_REGULATION_OF_LEUKOCYTE_APOPTOTIC_PROCESS	31	0.39	2.53	<0.001	0.001654861
GO_RECEPTOR_BINDING	948	0.07	2.53	<0.001	0.001652408
GSE3920_UNTREATED_VS_IFNA_TREATED_FIBROBLAST_UP	142	0.18	2.53	<0.001	0.001631964
GSE1740_UNSTIM_VS_IFNA_STIMULATED_MCSF_IFNG_DERIVED_MACROPHAGI	113	0.20	2.53	<0.001	0.001626505
GO_RHO_GTPASE_BINDING	69	0.27	2.53	<0.001	0.001627036
ROETH_TERT_TARGETS_UP	11	0.62	2.53	<0.001	0.001626063
GO_IMMUNE_RESPONSE_REGULATING_CELL_SURFACE_RECEPTOR_SIGNALING_	232	0.14	2.53	<0.001	0.001623563
GSE360_T_GONDII_VS_M_TUBERCULOSIS_DC_DN	136	0.19	2.53	<0.001	0.001616484
BHAT_ESR1_TARGETS_VIA_AKT1_UP	221	0.15	2.53	<0.001	0.001595879
GSE20727_DNFB_ALLERGEN_VS_ROS_INH_AND_DNFB_ALLERGEN_TREATED_DC	163	0.17	2.54	<0.001	0.001585824
HALLMARK_IL6_JAK_STAT3_SIGNALING	71	0.26	2.54	<0.001	0.001587809
GSE14415_INDUCED_TREG_VS_FOXP3_KO_INDUCED_TREG_IL2_CULTURE_UP	140	0.18	2.54	<0.001	0.001576127
GO_REGULATION_OF_LEUKOCYTE_APOPTOTIC_PROCESS	56	0.29	2.54	<0.001	0.001575084
GSE17721_LPS_VS_POLYIC_16H_BMDC_DN	169	0.17	2.54	<0.001	0.001529948
GSE7548_NAIVE_VS_DAY7_PCC_IMMUNIZATION_CD4_TCELL_DN	169	0.17	2.54	<0.001	0.001530367
GO_REGULATION_OF_ANATOMICAL_STRUCTURE_SIZE	359	0.12	2.54	<0.001	0.001530789
GSE17721_LPS_VS_POLYIC_24H_BMDC_DN	171	0.17	2.54	<0.001	0.001525135
GO_CYTOSKELETON	1488	0.06	2.54	<0.001	0.001519426
GO_CYTOSOLIC_RIBOSOME	69	0.26	2.54	<0.001	0.001521347
GO_BASEMENT_MEMBRANE	80	0.24	2.54	<0.001	0.001520188
WIEDERSCHAIN_TARGETS_OF_BMI1_AND_PCGF2	46	0.32	2.54	<0.001	0.00151596
GO_EXTRACELLULAR_MATRIX	278	0.13	2.54	<0.001	0.001516355
GO_RAS_PROTEIN_SIGNAL_TRANSDUCTION	134	0.19	2.54	<0.001	0.001513678
THUM_MIR21_TARGETS_HEART_DISEASE_UP	16	0.52	2.54	<0.001	0.001501713
GO_MEMBRANE_REGION	754	0.08	2.54	<0.001	0.00149596
GO_REGULATION_OF_HEMOPOIESIS	214	0.15	2.55	<0.001	0.001479364
GO_INTRACELLULAR_SIGNAL_TRANSDUCTION	1245	0.07	2.55	<0.001	0.001475122

GRAHAM_NORMAL_QUIESCENT_VS_NORMAL_DIVIDING_DN	74	0.26	2.55	<0.001	0.001469261
REACTOME_NCAM_SIGNALING_FOR_NEURITE_OUT_GROWTH	53	0.29	2.55	<0.001	0.001455651
GO_MORPHOGENESIS_OF_A_BRANCHING_STRUCTURE	120	0.20	2.55	<0.001	0.001407884
GSE21360_PRIMARY_VS_QUATERNARY_MEMORY_CD8_TCELL_UP	153	0.18	2.55	<0.001	0.001408127
GSE22886_NAIVE_CD4_TCELL_VS_MONOCYTE_DN	168	0.17	2.55	0.002020202	0.001378792
ZHONG_RESPONSE_TO_AZACITIDINE_AND_TSA_UP	131	0.19	2.56	<0.001	0.001366556
GSE17721_CPG_VS_GARDIQUIMOD_12H_BMDC_UP	140	0.18	2.56	<0.001	0.001363635
HALLMARK_WNT_BETA_CATENIN_SIGNALING	36	0.36	2.56	<0.001	0.001310779
WANG_RESPONSE_TO_GSK3_INHIBITOR_SB216763_DN	317	0.12	2.56	<0.001	0.001310912
BURTON_ADIPOGENESIS_PEAK_AT_24HR	39	0.35	2.56	<0.001	0.001306349
GNF2_CD1D	34	0.36	2.56	<0.001	0.001293939
GSE42021_TREG_PLN_VS_CD24HI_TREG_THYMUS_UP	171	0.17	2.56	<0.001	0.00127681
GO_ACTIN_FILAMENT_BINDING	100	0.22	2.56	<0.001	0.001275291
HTF_01	55	0.29	2.56	<0.001	0.001272249
GSE26030_UNSTIM_VS_RESTIM_TH1_DAY15_POST_POLARIZATION_DN	181	0.17	2.56	<0.001	0.001273906
GO_ANGIOGENESIS_INVOLVED_IN_WOUND_HEALING	14	0.57	2.57	<0.001	0.001264462
HALLMARK_IL2_STAT5_SIGNALING	168	0.17	2.57	<0.001	0.001256602
SNF5_DN.V1_UP	138	0.19	2.57	0.003944773	0.001244035
MODULE_419	32	0.40	2.57	<0.001	0.001220274
GNF2_MCL1	50	0.31	2.57	<0.001	0.001217137
GO_POSITIVE_REGULATION_OF_VASCULATURE_DEVELOPMENT	106	0.21	2.57	<0.001	0.00120921
TIAN_TNF_SIGNALING_VIA_NFKB	23	0.46	2.57	<0.001	0.001201313
WESTON_VEGFA_TARGETS_12HR	29	0.41	2.57	<0.001	0.001202893
GSE24634_IL4_VS_CTRL_TREATED_NAIVE_CD4_TCELL_DAY5_DN	136	0.19	2.57	<0.001	0.001193369
GSE11864_CSF1_IFNG_VS_CSF1_PAM3CYS_IN_MAC_DN	139	0.19	2.57	<0.001	0.001191742
GSE11961_FOLLICULAR_BCELL_VS_GERMINAL_CENTER_BCELL_DAY7_DN	145	0.19	2.57	<0.001	0.001190097
GSE9988_ANTI_TREM1_VS_LPS_MONOCYTE_UP	166	0.17	2.57	<0.001	0.001188463
GO_REGULATION_OF_CELL_DEATH	1102	0.07	2.58	<0.001	0.00117239
GNF2_CASP4	21	0.47	2.58	0.001976285	0.001172347
GSE11961_UNSTIM_VS_ANTI_IGM_AND_CD40_STIM_6H_FOLLICULAR_BCELL_U	154	0.18	2.58	<0.001	0.001167413
GO_RESPONSE_TO_MECHANICAL_STIMULUS	164	0.17	2.58	<0.001	0.00115609
GSE39110_UNTREATED_VS_IL2_TREATED_CD8_TCELL_DAY6_POST_IMMUNIZAT	161	0.18	2.58	<0.001	0.001156034
DAVICIONI_TARGETS_OF_PAX_FOXO1_FUSIONS_UP	217	0.15	2.58	<0.001	0.001154338
GSE36078_UNTREATED_VS_AD5_T425A_HEXON_INF_MOUSE_LUNG_DC_DN	162	0.18	2.58	<0.001	0.001151047

GO_RESPONSE_TO_TRANSFORMING_GROWTH_FACTOR_BETA	119	0.20	2.58	<0.001	0.001139619
PEDERSEN_METASTASIS_BY_ERBB2_ISOFORM_1	36	0.37	2.58	<0.001	0.00113787
GO_POSITIVE_REGULATION_OF_LOCOMOTION	330	0.12	2.58	<0.001	0.001132917
GSE3920_IFNA_VS_IFNG_TREATED_ENDOTHELIAL_CELL_UP	145	0.18	2.58	<0.001	0.001132811
AMIT_EGF_RESPONSE_60_HELA	42	0.34	2.58	<0.001	0.001127807
GSE23502_WT_VS_HDC_KO_MYELOID_DERIVED_SUPPRESSOR_CELL_COLON_TU	159	0.18	2.58	<0.001	0.001127698
BASSO_CD40_SIGNALING_UP	77	0.26	2.58	<0.001	0.001116194
GSE45365_HEALTHY_VS_MCMV_INFECTION_CD11B_DC_DN	110	0.21	2.58	<0.001	0.001114435
GSE20500_CTRL_VS_RARA_ANTAGONIST_TREATED_CD4_TCELL_UP	154	0.18	2.59	<0.001	0.001112669
CLASPER_LYMPHATIC_VESSELS_DURING_METASTASIS_UP	18	0.52	2.59	<0.001	0.001109255
GO_ESTABLISHMENT_OF_PROTEIN_LOCALIZATION_TO_ENDOPLASMIC_RETICUL	70	0.27	2.59	<0.001	0.001107506
GSE5542_UNTREATED_VS_IFNA_TREATED_EPITHELIAL_CELLS_6H_UP	143	0.19	2.59	<0.001	0.00110571
REACTOME_SRP_DEPENDENT_COTRANSLATIONAL_PROTEIN_TARGETING_TO_M	73	0.26	2.59	<0.001	0.001097359
GSE7768_OVA_WITH_LPS_VS_OVA_WITH_MPL_IMMUNIZED_MOUSE_WHOLE_	145	0.18	2.59	<0.001	0.001082329
GO_CELLULAR_RESPONSE_TO_INTERFERON_GAMMA	66	0.27	2.59	<0.001	0.001077172
GSE16385_IFNG_TNF_VS_IL4_STIM_MACROPHAGE_UP	169	0.17	2.59	<0.001	0.001078642
RADAEVA_RESPONSE_TO_IFNA1_UP	39	0.35	2.59	<0.001	0.001078501
VALK_AML_CLUSTER_11	34	0.37	2.59	0.001992032	0.001061729
PID_SYNDECAN_1_PATHWAY	38	0.35	2.59	<0.001	0.001061547
MODULE_16	400	0.11	2.59	<0.001	0.001061347
GO_NEGATIVE_REGULATION_OF_CYTOSKELETON_ORGANIZATION	195	0.16	2.59	<0.001	0.00105618
TONKS_TARGETS_OF_RUNX1_RUNX1T1_FUSION_SUSTAINED_IN_MONOCYTE_U	16	0.54	2.59	<0.001	0.001049367
GO_RESPONSE_TO_MOLECULE_OF_BACTERIAL_ORIGIN	243	0.15	2.60	<0.001	0.001042419
AZARE_NEOPLASTIC_TRANSFORMATION_BY_STAT3_UP	87	0.24	2.60	<0.001	0.001040526
LOPEZ_MESOTHELIOMA_SURVIVAL_OVERALL_DN	13	0.60	2.60	<0.001	0.001038675
REACTOME_DEVELOPMENTAL_BIOLOGY	312	0.13	2.60	<0.001	0.001040112
SCHLOSSER_SERUM_RESPONSE_UP	106	0.21	2.60	<0.001	0.001038215
GO_CYTOKINE_MEDIATED_SIGNALING_PATHWAY	275	0.14	2.60	<0.001	0.001031272
SCIAN_CELL_CYCLE_TARGETS_OF_TP53_AND_TP73_DN	19	0.49	2.60	<0.001	0.001032704
LIAO_METASTASIS	435	0.11	2.60	<0.001	0.00100548
GSE10240_IL22_VS_IL22_AND_IL17_STIM_PRIMARY_BRONCHIAL_EPITHELIAL_C	163	0.18	2.60	<0.001	9.90E-04
GO_LEUKOCYTE_CHEMOTAXIS	73	0.26	2.60	<0.001	9.90E-04
GSE9006_TYPE_1_DIABETES_AT_DX_VS_4MONTH_POST_DX_PBMC_UP	180	0.16	2.60	<0.001	9.91E-04
BOSCO_INTERFERON_INDUCED_ANTIVIRAL_MODULE	61	0.28	2.60	<0.001	9.87E-04

GSE29618_BCELL_VS_PDC_UP	159	0.17	2.60	<0.001	9.89E-04
TURASHVILI_BREAST_LOBULAR_CARCINOMA_VS_LOBULAR_NORMAL_DN	58	0.30	2.61	<0.001	9.75E-04
GO_RAS_GUANYL_NUCLEOTIDE_EXCHANGE_FACTOR_ACTIVITY	181	0.17	2.61	<0.001	9.76E-04
RASHI_RESPONSE_TO_IONIZING_RADIATION_2	107	0.22	2.61	<0.001	9.76E-04
GNF2_BUB1B	46	0.32	2.61	<0.001	9.77E-04
VART_KSHV_INFECTION_ANGIOGENIC_MARKERS_UP	126	0.20	2.61	<0.001	9.74E-04
GO_TUBE_MORPHOGENESIS	247	0.14	2.61	<0.001	9.60E-04
GO_TOLL_LIKE_RECEPTOR_SIGNALING_PATHWAY	76	0.25	2.61	<0.001	9.59E-04
CHYLA_CBFA2T3_TARGETS_UP	291	0.13	2.61	<0.001	9.50E-04
GSE42021_CD24HI_VS_CD24LOW_TREG_THYMUS_DN	131	0.20	2.61	<0.001	9.40E-04
GSE34156_UNTREATED_VS_24H_NOD2_AND_TLR1_TLR2_LIGAND_TREATED_M	120	0.21	2.61	<0.001	9.38E-04
GSE35825_IFNA_VS_IFNG_STIM_MACROPHAGE_UP	132	0.20	2.61	<0.001	9.32E-04
GNF2_BUB1	26	0.44	2.61	<0.001	9.26E-04
GO_NEGATIVE_REGULATION_OF_DEVELOPMENTAL_PROCESS	555	0.10	2.61	<0.001	9.24E-04
GSE43863_NAIVE_VS_MEMORY_TH1_CD4_TCELL_D150_LCMV_UP	96	0.23	2.61	<0.001	9.22E-04
GO_MORPHOGENESIS_OF_AN_EPITHELIUM	289	0.14	2.61	<0.001	9.18E-04
GNF2_SELL	39	0.35	2.61	<0.001	9.18E-04
GSE42021_TCONV_PLN_VS_TREG_PRECURSORS_THYMUS_UP	169	0.17	2.62	<0.001	8.90E-04
GSE32986_UNSTIM_VS_CURDLAN_LOWDOSE_STIM_DC_DN	177	0.17	2.62	<0.001	8.89E-04
MODULE_321	89	0.24	2.62	<0.001	8.91E-04
YU_MYC_TARGETS_UP	34	0.38	2.62	<0.001	8.83E-04
GO_SUPRAMOLECULAR_FIBER	418	0.11	2.62	<0.001	8.85E-04
GSE12392_CD8A_POS_VS_NEG_SPLEEN_DC_DN	176	0.17	2.62	<0.001	8.86E-04
GSE22589_HEALTHY_VS_SIV_INFECTED_DC_UP	170	0.17	2.62	<0.001	8.85E-04
GSE7852_TREG_VS_TCONV_THYMUS_UP	157	0.18	2.62	<0.001	8.78E-04
GSE40273_XBP1_KO_VS_WT_TREG_DN	169	0.17	2.62	<0.001	8.79E-04
GO_MICROTUBULE_CYTOSKELETON_ORGANIZATION	276	0.13	2.62	<0.001	8.80E-04
GSE17721_12H_VS_24H_LPS_BMDC_UP	166	0.18	2.62	<0.001	8.78E-04
GNF2_CKS2	48	0.33	2.62	<0.001	8.57E-04
GSE1112_HY_CD8AB_VS_HY_CD8AA_THYMOCYTE_RTOC_CULTURE_UP	123	0.21	2.62	<0.001	8.58E-04
REACTOME_CHONDROITIN_SULFATE_BIOSYNTHESIS	14	0.56	2.63	<0.001	8.48E-04
GSE2405_0H_VS_3H_A_PHAGOCYTOPHILUM_STIM_NEUTROPHIL_UP	156	0.18	2.63	<0.001	8.46E-04
YAGUE_PRETUMOR_DRUG_RESISTANCE_DN	12	0.62	2.63	<0.001	8.38E-04
ZHAN_MULTIPLE_MYELOMA_LB_DN	30	0.40	2.63	<0.001	8.29E-04

GO_REGULATION_OF_ACTIN_FILAMENT_BASED_PROCESS	262	0.14	2.63	<0.001	8.18E-04
GSE10325_MYELOID_VS_LUPUS_MYELOID_DN	162	0.18	2.63	<0.001	8.12E-04
GO_LEUKOCYTE_MIGRATION	183	0.17	2.63	0.001953125	8.10E-04
GSE14386_UNTREATED_VS_IFNA_TREATED_ACT_PBMC_MS_PATIENT_DN	122	0.20	2.63	<0.001	7.96E-04
GSE41176_UNSTIM_VS_ANTI_IGM_STIM_BCELL_3H_UP	136	0.19	2.64	<0.001	7.87E-04
HALLMARK_TGF_BETA_SIGNALING	49	0.32	2.64	<0.001	7.86E-04
CHASSOT_SKIN_WOUND	10	0.70	2.64	<0.001	7.87E-04
GO_REGULATION_OF_KINASE_ACTIVITY	613	0.09	2.64	<0.001	7.87E-04
GSE7509_UNSTIM_VS_IFNA_STIM_IMMATURE_DC_DN	136	0.20	2.64	<0.001	7.79E-04
GSE1460_INTRATHYMIC_T_PROGENITOR_VS_CD4_THYMOCYTE_DN	150	0.19	2.64	<0.001	7.58E-04
GSE13411_PLASMA_CELL_VS_MEMORY_BCELL_DN	166	0.18	2.64	<0.001	7.52E-04
RICKMAN_TUMOR_DIFFERENTIATED_MODERATELY_VS_POORLY_UP	100	0.23	2.64	<0.001	7.48E-04
MARTORIATI_MDM4_TARGETS_NEUROEPITHELIUM_UP	144	0.19	2.64	<0.001	7.42E-04
GO_LEUKOCYTE_ACTIVATION	292	0.14	2.64	<0.001	7.40E-04
GSE10239_NAIVE_VS_DAY4.5_EFF_CD8_TCELL_DN	165	0.18	2.65	<0.001	7.41E-04
WHITFIELD_CELL_CYCLE_G2_M	189	0.17	2.65	<0.001	7.42E-04
GSE10325_LUPUS_CD4_TCELL_VS_LUPUS_MYELOID_DN	172	0.18	2.65	<0.001	7.39E-04
SANA_RESPONSE_TO_IFNG_UP	47	0.33	2.65	<0.001	7.22E-04
REICHERT_MITOSIS_LIN9_TARGETS	27	0.42	2.65	0.002028398	7.23E-04
GNF2_S100A4	37	0.37	2.65	<0.001	7.23E-04
GSE21927_C26GM_VS_4T1_TUMOR_MONOCYTE_BALBC_UP	162	0.18	2.65	<0.001	7.20E-04
GO_REGULATION_OF_CELLULAR_COMPONENT_SIZE	269	0.14	2.65	<0.001	7.19E-04
GO_LAMELLIPODIUM_ORGANIZATION	33	0.39	2.65	<0.001	7.04E-04
GSE22886_NAIVE_CD8_TCELL_VS_MONOCYTE_DN	173	0.17	2.65	<0.001	7.03E-04
GSE31622_WT_VS_KLF3_KO_BCELL_DN	154	0.19	2.65	<0.001	7.00E-04
STK33_NOMO_UP	230	0.15	2.66	<0.001	7.01E-04
GSE2770_TGFB_AND_IL4_VS_IL12_TREATED_ACT_CD4_TCELL_6H_UP	184	0.17	2.66	<0.001	6.99E-04
GSE17721_LPS_VS_PAM3CSK4_1H_BMDC_DN	168	0.18	2.66	<0.001	6.98E-04
GSE1460_DP_VS_CD4_THYMOCYTE_DN	151	0.19	2.66	<0.001	6.97E-04
GSE360_T_GONDII_VS_B_MALAYI_HIGH_DOSE_DC_UP	137	0.19	2.66	<0.001	6.91E-04
GSE36826_NORMAL_VS_STAPH_AUREUS_INF_IL1R_KO_SKIN_DN	170	0.17	2.66	<0.001	6.92E-04
GSE24634_TREG_VS_TCONV_POST_DAY10_IL4_CONVERSION_UP	172	0.17	2.66	<0.001	6.93E-04
LAMB_CCND1_TARGETS	16	0.55	2.66	<0.001	6.92E-04
GSE14000_UNSTIM_VS_4H_LPS_DC_DN	148	0.19	2.66	<0.001	6.89E-04

CONCANNON_APOPTOSIS_BY_EPOXOMICIN_UP	201	0.16	2.66	<0.001	6.91E-04
GSE13484_3H_UNSTIM_VS_YF17D_VACCINE_STIM_PBMC_DN	148	0.19	2.66	<0.001	6.73E-04
GSE14000_UNSTIM_VS_16H_LPS_DC_TRANSLATED_RNA_DN	144	0.19	2.66	<0.001	6.74E-04
GSE17721_ALL_VS_24H_PAM3CSK4_BMDC_UP	167	0.18	2.66	<0.001	6.71E-04
GSE43863_TFH_VS_LY6C_LOW_CXCR5NEG_EFFECTOR_CD4_TCELL_UP	176	0.18	2.66	<0.001	6.72E-04
BENPORATH_CYCLING_GENES	551	0.10	2.66	<0.001	6.66E-04
GSE36888_UNTREATED_VS_IL2_TREATED_STAT5_AB_KNOCKIN_TCELL_2H_UP	143	0.19	2.67	<0.001	6.61E-04
GSE45365_WT_VS_IFNAR_KO_CD11B_DC_MCMV_INFECTION_DN	89	0.24	2.67	<0.001	6.55E-04
GSE2128_CTRL_VS_MIMETOPE_NEGATIVE_SELECTION_DP_THYMOCYTE_NOD_U	173	0.17	2.67	<0.001	6.54E-04
GSE6259_33D1_POS_VS_DEC205_POS_FLT3L_INDUCED_SPLENIC_DC_UP	139	0.19	2.67	<0.001	6.53E-04
GO_RESPONSE_TO_BACTERIUM	315	0.13	2.67	<0.001	6.54E-04
GSE20727_ROS_INH_VS_ROS_INH_AND_DNFB_ALLERGEN_TREATED_DC_UP	168	0.18	2.67	<0.001	6.53E-04
GO_CELL_DIVISION	401	0.12	2.67	<0.001	6.35E-04
GSE1432_CTRL_VS_IFNG_24H_MICROGLIA_DN	156	0.18	2.67	<0.001	6.26E-04
ZHANG_TLX_TARGETS_36HR_UP	198	0.16	2.67	<0.001	6.25E-04
HALLMARK_APICAL_JUNCTION	159	0.18	2.67	<0.001	6.26E-04
GSE15330_WT_VS_IKAROS_KO_HSC_DN	179	0.18	2.67	<0.001	6.08E-04
GSE22601_DOUBLE_NEGATIVE_VS_CD4_SINGLE_POSITIVE_THYMOCYTE_DN	183	0.17	2.68	<0.001	6.05E-04
AMUNDSON_GAMMA_RADIATION_RESPONSE	36	0.39	2.68	<0.001	6.06E-04
NAKAYAMA_SOFT_TISSUE_TUMORS_PCA1_UP	57	0.31	2.68	<0.001	6.07E-04
GO_REGULATION_OF_CYTOKINE_BIOSYNTHETIC_PROCESS	65	0.28	2.68	<0.001	6.08E-04
CHANG_CYCLING_GENES	121	0.21	2.68	<0.001	6.02E-04
GSE21546_ELK1_KO_VS_SAP1A_KO_AND_ELK1_KO_DP_THYMOCYTES_UP	125	0.20	2.68	<0.001	5.99E-04
GSE26343_WT_VS_NFAT5_KO_MACROPHAGE_DN	172	0.17	2.68	<0.001	5.94E-04
GO_POSITIVE_REGULATION_OF_CELL_PROLIFERATION	558	0.10	2.68	<0.001	5.70E-04
GSE34156_UNTREATED_VS_24H_TLR1_TLR2_LIGAND_TREATED_MONOCYTE_UI	111	0.22	2.69	<0.001	5.43E-04
GSE9988_LOW_LPS_VS_CTRL_TREATED_MONOCYTE_UP	140	0.19	2.69	<0.001	5.38E-04
GSE9988_LOW_LPS_VS_ANTI_TREM1_AND_LPS_MONOCYTE_UP	159	0.19	2.69	<0.001	5.39E-04
GO_REGULATION_OF_CELL_MORPHOGENESIS	444	0.11	2.69	<0.001	5.34E-04
WANG_METHYLATED_IN_BREAST_CANCER	28	0.42	2.69	<0.001	5.31E-04
GSE17721_LPS_VS_PAM3CSK4_8H_BMDC_UP	168	0.18	2.69	<0.001	5.26E-04
BERENJENO_TRANSFORMED_BY_RHOA_FOREVER_DN	30	0.41	2.69	<0.001	5.27E-04
GSE17721_POLYIC_VS_PAM3CSK4_1H_BMDC_DN	167	0.18	2.69	<0.001	5.28E-04
GEORGES_CELL_CYCLE_MIR192_TARGETS	60	0.29	2.69	<0.001	5.27E-04

GO_POSITIVE_REGULATION_OF_CELL_ADHESION	267	0.15	2.69	<0.001	5.28E-04
GSE6259_DEC205_POS_DC_VS_BCELL_DN	151	0.19	2.69	<0.001	5.28E-04
GSE43863_DAY6_EFF_VS_DAY150_MEM_TFH_CD4_TCELL_UP	170	0.18	2.69	<0.001	5.29E-04
GO_REGULATION_OF_CELL_SUBSTRATE_ADHESION	136	0.20	2.69	<0.001	5.22E-04
GSE10325_CD4_TCELL_VS_MYELOID_DN	161	0.18	2.69	<0.001	5.23E-04
GNF2_CD53	48	0.34	2.69	<0.001	5.18E-04
GSE42021_TREG_PLN_VS_CD24INT_TREG_THYMUS_UP	177	0.17	2.70	<0.001	5.11E-04
ZHANG_INTERFERON_RESPONSE	19	0.51	2.70	<0.001	5.12E-04
IZADPANAH_STEM_CELL_ADIPOSE_VS_BONE_UP	96	0.24	2.70	<0.001	5.08E-04
FRIDMAN_SENESCENCE_UP	67	0.28	2.70	<0.001	5.09E-04
GO_NEGATIVE_REGULATION_OF_MULTICELLULAR_ORGANISMAL_PROCESS	673	0.09	2.70	<0.001	5.10E-04
KEGG_RIBOSOME	52	0.32	2.70	<0.001	5.11E-04
GSE17721_12H_VS_24H_GARDIQUIMOD_BMDC_UP	162	0.18	2.70	<0.001	5.10E-04
SHEPARD_CRUSH_AND_BURN_MUTANT_DN	131	0.20	2.70	<0.001	5.09E-04
GO_KINASE_BINDING	513	0.11	2.70	<0.001	5.10E-04
GSE34006_A2AR_KO_VS_A2AR_AGONIST_TREATED_TREG_UP	174	0.17	2.70	<0.001	5.10E-04
HOFFMANN_LARGE_TO_SMALL_PRE_BII_LYMPHOCYTE_UP	147	0.19	2.70	<0.001	5.07E-04
GSE37534_UNTREATED_VS_GW1929_TREATED_CD4_TCELL_PPARG1_AND_FOX	166	0.18	2.70	<0.001	5.08E-04
GSE1448_CTRL_VS_ANTI_VBETA5_DP_THYMOCYTE_DN	167	0.18	2.70	<0.001	5.05E-04
GO_REGULATION_OF_CELL_PROLIFERATION	1057	0.08	2.70	<0.001	5.06E-04
GSE22282_HYPOXIA_VS_NORMOXIA_MYELOID_DC_UP	153	0.19	2.71	<0.001	5.02E-04
GSE39110_DAY3_VS_DAY6_POST_IMMUNIZATION_CD8_TCELL_WITH_IL2_TREA	160	0.18	2.71	<0.001	4.99E-04
GSE45365_WT_VS_IFNAR_KO_CD8A_DC_DN	167	0.18	2.71	<0.001	4.98E-04
GO_CELL_SURFACE	476	0.11	2.71	<0.001	4.99E-04
GSE9601_UNTREATED_VS_PI3K_INHIBITOR_TREATED_HCMV_INF_MONOCYTE_	154	0.19	2.71	<0.001	4.96E-04
GSE43863_TFH_VS_LY6C_INT_CXCR5POS_EFFECTOR_CD4_TCELL_UP	168	0.18	2.71	<0.001	4.92E-04
IGLESIAS_E2F_TARGETS_UP	145	0.19	2.71	<0.001	4.93E-04
GO_REGULATION_OF_RESPONSE_TO_EXTERNAL_STIMULUS	661	0.09	2.71	<0.001	4.90E-04
WANG_ESOPHAGUS_CANCER_VS_NORMAL_UP	89	0.25	2.71	<0.001	4.85E-04
GSE36476_CTRL_VS_TSST_ACT_40H_MEMORY_CD4_TCELL_YOUNG_DN	170	0.17	2.71	<0.001	4.83E-04
GSE15330_HSC_VS_LYMPHOID_PRIMED_MULTIPOTENT_PROGENITOR_DN	158	0.19	2.71	<0.001	4.76E-04
GNF2_MCM4	49	0.33	2.71	<0.001	4.77E-04
GSE21360_SECONDARY_VS_QUATERNARY_MEMORY_CD8_TCELL_UP	143	0.19	2.71	<0.001	4.71E-04
ROY_WOUND_BLOOD_VESSEL_UP	44	0.34	2.71	<0.001	4.72E-04

RICKMAN_TUMOR_DIFFERENTIATED_WELL_VS_MODERATELY_DN	64	0.29	2.71	<0.001	4.69E-04
GSE27670_BLIMP1_VS_LMP1_TRANSDUCED_GC_BCELL_UP	159	0.18	2.71	<0.001	4.69E-04
ZHOU_INFLAMMATORY_RESPONSE_LPS_UP	235	0.16	2.72	<0.001	4.62E-04
GSE23568_CTRL_VS_ID3_TRANSDUCED_CD8_TCELL_DN	163	0.18	2.72	<0.001	4.46E-04
GSE14415_ACT_TCONV_VS_ACT_NATURAL_TREG_DN	156	0.19	2.72	<0.001	4.38E-04
GO_ACTIN_CYTOSKELETON	378	0.12	2.72	<0.001	4.39E-04
CYCLIN_D1_KE_.V1_UP	144	0.19	2.72	<0.001	4.31E-04
HALLMARK_KRAS_SIGNALING_UP	167	0.18	2.72	<0.001	4.32E-04
GSE18791_CTRL_VS_NEWCASTLE_VIRUS_DC_16H_DN	135	0.20	2.72	<0.001	4.20E-04
GSE2405_HEAT_KILLED_LYSATE_VS_LIVE_A_PHAGOCYTOPHILUM_STIM_NEUTR	169	0.18	2.73	<0.001	4.19E-04
BROCKE_APOPTOSIS_REVERSED_BY_IL6	129	0.21	2.73	<0.001	4.19E-04
GSE9988_ANTI_TREM1_AND_LPS_VS_CTRL_TREATED_MONOCYTES_UP	144	0.19	2.73	<0.001	4.20E-04
GSE3720_UNSTIM_VS_PMA_STIM_VD2_GAMMADELTA_TCELL_DN	162	0.18	2.73	<0.001	4.19E-04
GSE44649_WT_VS_MIR155_KO_ACTIVATED_CD8_TCELL_UP	173	0.18	2.73	<0.001	4.15E-04
CROONQUIST_NRAS_SIGNALING_DN	67	0.29	2.73	<0.001	4.09E-04
REACTOME_INFLAMMASOMES	15	0.58	2.73	<0.001	4.06E-04
BMI1_DN.V1_UP	111	0.22	2.73	<0.001	4.07E-04
GNF2_SMC2L1	31	0.41	2.73	<0.001	4.05E-04
LIM_MAMMARY_STEM_CELL_UP	406	0.12	2.73	<0.001	4.06E-04
GO_RESPONSE_TO_EXOGENOUS_DSRNA	27	0.44	2.73	<0.001	4.04E-04
MODULE_53	301	0.14	2.73	<0.001	4.05E-04
GSE20754_WT_VS_TCF1_KO_MEMORY_CD8_TCELL_UP	174	0.17	2.74	<0.001	3.93E-04
MULLIGHAN_MLL_SIGNATURE_1_UP	307	0.14	2.74	<0.001	3.94E-04
GO_INFLAMMATORY_RESPONSE	275	0.15	2.74	<0.001	3.92E-04
GSE26343_UNSTIM_VS_LPS_STIM_NFAT5_KO_MACROPHAGE_UP	176	0.18	2.74	<0.001	3.88E-04
PID_INTEGRIN1_PATHWAY	63	0.30	2.74	<0.001	3.87E-04
PLASARI_TGFB1_TARGETS_1HR_UP	32	0.41	2.74	<0.001	3.85E-04
CHEMNITZ_RESPONSE_TO_PROSTAGLANDIN_E2_UP	122	0.22	2.74	<0.001	3.71E-04
GSE30962_ACUTE_VS_CHRONIC_LCMV_SECONDARY_INF_CD8_TCELL_DN	170	0.18	2.74	<0.001	3.69E-04
GNF2_TTK	36	0.39	2.74	<0.001	3.63E-04
GSE6269_HEALTHY_VS_FLU_INF_PBMC_DN	127	0.21	2.75	<0.001	3.59E-04
GO_CELL_CHEMOTAXIS	109	0.22	2.75	<0.001	3.60E-04
GO_PHOSPHOLIPID_BINDING	265	0.15	2.75	<0.001	3.54E-04
GO_ENZYME_LINKED_RECEPTOR_PROTEIN_SIGNALING_PATHWAY	539	0.11	2.75	<0.001	3.55E-04

GUENTHER_GROWTH_SPHERICAL_VS_ADHERENT_DN	25	0.48	2.75	<0.001	3.53E-04
GSE43863_LY6C_INT_CXCR5POS_VS_LY6C_LOW_CXCR5NEG_EFFECTOR_CD4_TC	148	0.19	2.75	<0.001	3.54E-04
GSE6674_UNSTIM_VS_ANTI_IGM_AND_CPG_STIM_BCELL_DN	178	0.18	2.75	<0.001	3.52E-04
NAKAMURA_TUMOR_ZONE_PERIPHERAL_VS_CENTRAL_UP	247	0.15	2.75	<0.001	3.53E-04
GSE12963_UNINF_VS_ENV_AND_NEF_AND_VPR_DEFICIENT_HIV1_INF_CD4_TC	88	0.25	2.76	<0.001	3.45E-04
GSE9988_ANTI_TREM1_AND_LPS_VS_CTRL_TREATED_MONOCYTES_DN	176	0.18	2.76	<0.001	3.39E-04
GO_ENDOTHELIUM_DEVELOPMENT	80	0.26	2.76	<0.001	3.28E-04
GSE22140_GERMFREE_VS_SPF_ARTHRITIC_MOUSE_CD4_TCELL_UP	161	0.19	2.76	<0.001	3.22E-04
GRUETZMANN_PANCREATIC_CANCER_UP	309	0.14	2.76	<0.001	3.23E-04
GO_POSITIVE_REGULATION_OF_PROTEIN_METABOLIC_PROCESS	1141	0.07	2.77	<0.001	3.19E-04
GO_SINGLE_ORGANISM_CELL_ADHESION	324	0.13	2.77	<0.001	3.17E-04
KHETCHOUMIAN_TRIM24_TARGETS_UP	43	0.36	2.77	<0.001	3.15E-04
GSE28726_NAIVE_CD4_TCELL_VS_NAIVE_NKTCELL_UP	168	0.18	2.77	<0.001	3.16E-04
MEL18_DN.V1_UP	110	0.22	2.77	<0.001	3.10E-04
GSE3982_NEUTROPHIL_VS_CENT_MEMORY_CD4_TCELL_UP	167	0.18	2.77	<0.001	3.08E-04
GO_INTERFERON_GAMMA_MEDIATED_SIGNALING_PATHWAY	43	0.35	2.77	<0.001	3.04E-04
GSE29618_BCELL_VS_MONOCYTE_DN	177	0.18	2.77	<0.001	3.05E-04
GO_CELLULAR_RESPONSE_TO_BIOTIC_STIMULUS	136	0.20	2.78	<0.001	3.05E-04
FURUKAWA_DUSP6_TARGETS_PCI35_DN	58	0.31	2.78	<0.001	3.06E-04
PID_RAC1_PATHWAY	51	0.33	2.78	<0.001	3.04E-04
GSE360_LOW_DOSE_B_MALAYI_VS_M_TUBERCULOSIS_DC_DN	160	0.18	2.78	<0.001	3.00E-04
GSE23308_CTRL_VS_CORTICOSTERONE_TREATED_MACROPHAGE_DN	173	0.18	2.78	<0.001	2.91E-04
GSE22432_MULTIPOTENT_PROGENITOR_VS_CDC_UP	142	0.20	2.78	<0.001	2.87E-04
GO_PROTEIN_TARGETING_TO_MEMBRANE	117	0.22	2.78	<0.001	2.86E-04
GSE40274_CTRL_VS_FOXP3_AND_PBX1_TRANSDUCED_ACTIVATED_CD4_TCELL_	130	0.21	2.78	<0.001	2.84E-04
SARTIPY_BLUNTED_BY_INSULIN_RESISTANCE_UP	16	0.59	2.78	<0.001	2.82E-04
DIRMEIER_LMP1_RESPONSE_EARLY	51	0.32	2.78	<0.001	2.82E-04
SENGUPTA_NASOPHARYNGEAL_CARCINOMA_UP	246	0.15	2.79	<0.001	2.81E-04
GSE26495_NAIVE_VS_PD1HIGH_CD8_TCELL_DN	155	0.19	2.79	<0.001	2.74E-04
GSE9509_LPS_VS_LPS_AND_IL10_STIM_IL10_KO_MACROPHAGE_20MIN_DN	174	0.18	2.79	<0.001	2.75E-04
GSE17186_BLOOD_VS_CORD_BLOOD_CD21HIGH_TRANSITIONAL_BCELL_UP	187	0.18	2.79	<0.001	2.68E-04
GO_REGULATION_OF_RHO_PROTEIN_SIGNAL_TRANSDUCTION	91	0.25	2.79	<0.001	2.66E-04
GSE7348_UNSTIM_VS_TOLERIZED_AND_LPS_STIM_MACROPHAGE_DN	133	0.21	2.80	<0.001	2.62E-04
PETROVA_ENDOTHELIUM_LYMPHATIC_VS_BLOOD_DN	136	0.20	2.80	<0.001	2.60E-04

GRAHAM_CML_QUIESCENT_VS_NORMAL_QUIESCENT_UP	65	0.29	2.80	<0.001	2.61E-04
ZHOU_INFLAMMATORY_RESPONSE_FIMA_UP	324	0.14	2.80	<0.001	2.61E-04
LINDSTEDT_DENDRITIC_CELL_MATURATION_B	43	0.35	2.80	<0.001	2.62E-04
GO_RECEPTOR_MEDIATED_ENDOCYTOSIS	157	0.19	2.80	<0.001	2.62E-04
JISON_SICKLE_CELL_DISEASE_UP	151	0.19	2.80	<0.001	2.63E-04
ROZANOV_MMP14_TARGETS_SUBSET	30	0.43	2.80	<0.001	2.59E-04
LABBE_WNT3A_TARGETS_UP	93	0.26	2.81	<0.001	2.54E-04
GSE17186_CD21LOW_VS_CD21HIGH_TRANSITIONAL_BCELL_DN	176	0.18	2.81	<0.001	2.55E-04
JOHNSTONE_PARVB_TARGETS_3_UP	365	0.13	2.81	<0.001	2.46E-04
GO_PATTERN_RECOGNITION_RECEPTOR_SIGNALING_PATHWAY	91	0.25	2.81	<0.001	2.46E-04
TSAI_RESPONSE_TO_RADIATION_THERAPY	26	0.46	2.81	<0.001	2.47E-04
GSE41176_UNSTIM_VS_ANTI_IGM_STIM_BCELL_1H_UP	148	0.20	2.81	<0.001	2.45E-04
GSE32986_GMCSF_VS_GMCSF_AND_CURDLAN_LOWDOSE_STIM_DC_UP	169	0.19	2.81	<0.001	2.40E-04
GSE25088_IL4_VS_IL4_AND_ROSIGLITAZONE_STIM_STAT6_KO_MACROPHAGE_	156	0.19	2.81	<0.001	2.38E-04
GO_ENDOTHELIAL_CELL_DIFFERENTIATION	64	0.30	2.82	<0.001	2.39E-04
MARKEY_RB1_ACUTE_LOF_DN	186	0.18	2.82	<0.001	2.34E-04
GO_RUFFLE	141	0.21	2.82	<0.001	2.35E-04
GO_SIDE_OF_MEMBRANE	281	0.15	2.82	<0.001	2.30E-04
GO_POSITIVE_REGULATION_OF_KINASE_ACTIVITY	386	0.13	2.82	<0.001	2.31E-04
GSE360_HIGH_DOSE_B_MALAYI_VS_M_TUBERCULOSIS_DC_DN	149	0.20	2.82	<0.001	2.31E-04
SMIRNOV_RESPONSE_TO_IR_6HR_DN	91	0.26	2.82	<0.001	2.32E-04
GSE45365_WT_VS_IFNAR_KO_BCELL_DN	104	0.24	2.82	<0.001	2.32E-04
GO_REGULATION_OF_CELL_SHAPE	122	0.22	2.82	<0.001	2.33E-04
DELYS_THYROID_CANCER_UP	335	0.13	2.83	<0.001	2.21E-04
GSE14769_UNSTIM_VS_40MIN_LPS_BMDM_DN	166	0.19	2.83	<0.001	2.16E-04
GSE360_CTRL_VS_M_TUBERCULOSIS_DC_DN	141	0.21	2.83	<0.001	2.14E-04
GO_REGULATION_OF_TYPE_I_INTERFERON_PRODUCTION	98	0.24	2.83	<0.001	2.15E-04
GSE37416_0H_VS_6H_F_TULARENSIS_LVS_NEUTROPHIL_DN	169	0.19	2.83	<0.001	2.13E-04
GO_VESICLE_MEDIATED_TRANSPORT	963	0.08	2.84	<0.001	1.98E-04
GO_ACTIN_FILAMENT_BASED_PROCESS	392	0.13	2.84	<0.001	1.96E-04
GO_BRANCHING_MORPHOGENESIS_OF_AN_EPITHELIAL_TUBE	98	0.24	2.84	<0.001	1.94E-04
WESTON_VEGFA_TARGETS	90	0.26	2.84	0.00204499	1.94E-04
GSE15330_WT_VS_IKAROS_KO_GRANULOCYTE_MONOCYTE_PROGENITOR_DN	160	0.20	2.84	<0.001	1.95E-04
GSE9006_HEALTHY_VS_TYPE_1_DIABETES_PBMC_AT_DX_DN	142	0.21	2.84	<0.001	1.93E-04

MONTERO_THYROID_CANCER_POOR_SURVIVAL_UP	12	0.66	2.85	<0.001	1.93E-04
GNF2_MSN	23	0.50	2.85	<0.001	1.93E-04
PID_INTEGRIN3_PATHWAY	39	0.38	2.85	<0.001	1.94E-04
GSE32986_UNSTIM_VS_GMCSF_STIM_DC_DN	161	0.20	2.85	<0.001	1.94E-04
DAUER_STAT3_TARGETS_DN	38	0.39	2.85	<0.001	1.90E-04
GO_CELLULAR_RESPONSE_TO_CYTOKINE_STIMULUS	398	0.12	2.85	<0.001	1.90E-04
REACTOME_COLLAGEN_FORMATION	46	0.36	2.86	<0.001	1.85E-04
SEKI_INFLAMMATORY_RESPONSE_LPS_UP	64	0.30	2.86	<0.001	1.83E-04
GO_CELL_DEVELOPMENT	974	0.08	2.86	<0.001	1.83E-04
GO_POSITIVE_REGULATION_OF_CELL_DIFFERENTIATION	599	0.10	2.86	<0.001	1.74E-04
GSE7852_THYMUS_VS_FAT_TCONV_DN	172	0.19	2.87	<0.001	1.71E-04
TURASHVILI_BREAST_LOBULAR_CARCINOMA_VS_DUCTAL_NORMAL_UP	61	0.31	2.87	<0.001	1.69E-04
REACTOME_TRANSLATION	105	0.24	2.87	<0.001	1.67E-04
GSE35825_UNTREATED_VS_IFNA_STIM_MACROPHAGE_DN	137	0.22	2.87	<0.001	1.67E-04
GSE34156_NOD2_LIGAND_VS_TLR1_TLR2_LIGAND_6H_TREATED_MONOCYTE_U	170	0.19	2.87	<0.001	1.68E-04
GO_COLLAGEN_TRIMER	56	0.33	2.87	<0.001	1.68E-04
GO_REGULATION_OF_PROTEIN_COMPLEX_DISASSEMBLY	188	0.18	2.87	<0.001	1.68E-04
GO_CYTOSKELETAL_PROTEIN_BINDING	673	0.10	2.87	<0.001	1.66E-04
KIM_GLIS2_TARGETS_UP	70	0.29	2.87	<0.001	1.64E-04
GSE10325_LUPUS_BCELL_VS_LUPUS_MYELOID_DN	176	0.19	2.87	<0.001	1.64E-04
GSE37416_0H_VS_3H_F_TULARENSIS_LVS_NEUTROPHIL_DN	171	0.19	2.87	<0.001	1.62E-04
GO_PHOSPHATIDYLINOSITOL_BINDING	168	0.20	2.88	<0.001	1.59E-04
GSE36891_UNSTIM_VS_POLYIC_TLR3_STIM_PERITONEAL_MACROPHAGE_DN	182	0.18	2.88	<0.001	1.60E-04
GO_RHO_GUANYL_NUCLEOTIDE_EXCHANGE_FACTOR_ACTIVITY	62	0.31	2.88	<0.001	1.60E-04
GO_RESPONSE_TO_BIOTIC_STIMULUS	556	0.11	2.88	<0.001	1.61E-04
GSE14415_INDUCED_VS_NATURAL_TREG_DN	150	0.21	2.88	<0.001	1.56E-04
GO_REGULATION_OF_HYDROLASE_ACTIVITY	990	0.08	2.88	<0.001	1.56E-04
LEE_BMP2_TARGETS_DN	779	0.09	2.89	<0.001	1.48E-04
TGFB_UP.V1_UP	149	0.20	2.89	<0.001	1.46E-04
WESTON_VEGFA_TARGETS_6HR	51	0.34	2.89	<0.001	1.46E-04
SWEET_KRAS_TARGETS_UP	75	0.28	2.89	<0.001	1.46E-04
GO_RESPONSE_TO_INTERFERON_GAMMA	77	0.28	2.89	<0.001	1.41E-04
SENESE_HDAC1_AND_HDAC2_TARGETS_UP	172	0.19	2.89	<0.001	1.42E-04
HALLMARK_ALLOGRAFT_REJECTION	141	0.21	2.90	<0.001	1.34E-04

SHETH_LIVER_CANCER_VS_TXNIP_LOSS_PAM2	124	0.22	2.90	<0.001	1.34E-04
SMID_BREAST_CANCER_BASAL_UP	426	0.12	2.90	<0.001	1.34E-04
MODULE_1	330	0.14	2.90	<0.001	1.35E-04
STK33_UP	224	0.17	2.90	<0.001	1.35E-04
GO_CELL_ADHESION_MOLECULE_BINDING	147	0.21	2.90	<0.001	1.35E-04
GSE22935_WT_VS_MYD88_KO_MACROPHAGE_12H_MBOVIS_BCG_STIM_UP	147	0.21	2.90	<0.001	1.33E-04
AMIT_SERUM_RESPONSE_60_MCF10A	53	0.33	2.91	<0.001	1.30E-04
GSE40666_UNTREATED_VS_IFNA_STIM_EFFECTOR_CD8_TCELL_90MIN_UP	156	0.20	2.91	<0.001	1.31E-04
GO_TRANSLATIONAL_INITIATION	102	0.24	2.91	<0.001	1.31E-04
MORF_TPT1	72	0.29	2.91	<0.001	1.31E-04
GO_POSITIVE_REGULATION_OF_TYPE_I_INTERFERON_PRODUCTION	68	0.30	2.91	<0.001	1.32E-04
GSE3337_4H_VS_16H_IFNG_IN_CD8POS_DC_DN	165	0.19	2.91	<0.001	1.32E-04
GSE21360_TERTIARY_VS_QUATERNARY_MEMORY_CD8_TCELL_DN	152	0.20	2.91	<0.001	1.32E-04
GU_PDEF_TARGETS_UP	63	0.30	2.91	<0.001	1.33E-04
KEGG_REGULATION_OF_ACTIN_CYTOSKELETON	179	0.19	2.91	<0.001	1.30E-04
STK33_SKM_UP	207	0.17	2.91	<0.001	1.28E-04
MEK_UP.V1_UP	161	0.20	2.91	<0.001	1.25E-04
TAKEDA_TARGETS_OF_NUP98_HOXA9_FUSION_3D_UP	118	0.22	2.91	<0.001	1.25E-04
GO_RESPONSE_TO_VIRUS	167	0.19	2.92	<0.001	1.23E-04
GNF2_INPP5D	36	0.41	2.92	<0.001	1.17E-04
GO_PHAGOCYTOSIS	140	0.22	2.92	<0.001	1.18E-04
GSE37301_MULTIPOTENT_PROGENITOR_VS_GRAN_MONO_PROGENITOR_DN	170	0.19	2.92	<0.001	1.18E-04
GSE32164_RESTING_DIFFERENTIATED_VS_ALTERNATIVELY_ACT_M2_MACROPH	155	0.20	2.92	<0.001	1.18E-04
GSE7219_WT_VS_NIK_NFKB2_KO_DC_UP	169	0.19	2.92	<0.001	1.13E-04
GO_INTEGRIN_MEDIATED_SIGNALING_PATHWAY	71	0.30	2.92	<0.001	1.13E-04
GO_EXTRACELLULAR_MATRIX_STRUCTURAL_CONSTITUENT	41	0.38	2.92	<0.001	1.10E-04
GSE26030_TH1_VS_TH17_DAY5_POST_POLARIZATION_DN	166	0.20	2.93	<0.001	9.90E-05
GSE17721_12H_VS_24H_PAM3CSK4_BMDC_UP	171	0.20	2.93	<0.001	9.92E-05
MODULE_57	49	0.35	2.93	<0.001	9.36E-05
ETS2_B	230	0.16	2.93	<0.001	9.09E-05
SANA_TNF_SIGNALING_UP	61	0.32	2.94	<0.001	8.82E-05
ICHIBA_GRAFT_VERSUS_HOST_DISEASE_D7_UP	85	0.27	2.94	<0.001	8.84E-05
GSE29618_BCELL_VS_MDC_DAY7_FLU_VACCINE_DN	167	0.20	2.94	<0.001	8.86E-05
GSE17301_CTRL_VS_48H_ACD3_ACD28_STIM_CD8_TCELL_DN	166	0.20	2.94	<0.001	8.88E-05

GSE3920_UNTREATED_VS_IFNG_TREATED_ENDOTHELIAL_CELL_UP	108	0.24	2.94	<0.001	8.61E-05
GSE38681_WT_VS_LYL1_KO_LYMPHOID_PRIMED_MULTIPOTENT_PROGENITOR_	172	0.20	2.94	<0.001	8.33E-05
GO_REGULATION_OF_BODY_FLUID_LEVELS	343	0.14	2.94	<0.001	8.35E-05
GSE24142_EARLY_THYMIC_PROGENITOR_VS_DN2_THYMOCYTE_FETAL_UP	162	0.20	2.95	<0.001	8.37E-05
GO_REGULATION_OF_PHOSPHORUS_METABOLIC_PROCESS	1173	0.08	2.95	<0.001	8.39E-05
GO_PROTEIN_COMPLEX_BINDING	746	0.10	2.95	<0.001	8.11E-05
FINETTI_BREAST_CANCER_KINOME_RED	16	0.62	2.95	<0.001	8.13E-05
BMI1_DN_MEL18_DN.V1_UP	111	0.23	2.95	<0.001	8.15E-05
MODULE_52	354	0.14	2.95	<0.001	8.17E-05
GSE37532_WT_VS_PPARG_KO_LN_TCONV_DN	151	0.21	2.95	<0.001	7.89E-05
GALINDO_IMMUNE_RESPONSE_TO_ENTEROTOXIN	70	0.30	2.96	<0.001	7.30E-05
GSE24142_DN2_VS_DN3_THYMOCYTE_UP	167	0.19	2.96	<0.001	7.32E-05
BILD_HRAS_ONCOGENIC_SIGNATURE	195	0.18	2.96	<0.001	6.73E-05
ZHOU_INFLAMMATORY_RESPONSE_LIVE_UP	298	0.15	2.96	<0.001	6.75E-05
GSE29617_CTRL_VS_DAY7_TIV_FLU_VACCINE_PBMC_2008_UP	150	0.21	2.96	<0.001	6.77E-05
REACTOME_INFLUENZA_VIRAL_RNA_TRANSCRIPTION_AND_REPLICATION	66	0.31	2.96	<0.001	6.48E-05
GO_POSITIVE_REGULATION_OF_PROTEIN_SERINE_THREONINE_KINASE_ACTIVIT	233	0.17	2.97	<0.001	6.49E-05
GO_GUANYL_NUCLEOTIDE_EXCHANGE_FACTOR_ACTIVITY	243	0.17	2.97	<0.001	6.51E-05
GSE32901_NAIVE_VS_TH17_NEG_CD4_TCELL_UP	139	0.22	2.97	<0.001	6.53E-05
GO_RESPONSE_TO_EXTERNAL_STIMULUS	1176	0.08	2.97	<0.001	6.23E-05
GSE24142_EARLY_THYMIC_PROGENITOR_VS_DN2_THYMOCYTE_UP	175	0.19	2.97	<0.001	6.25E-05
BROWN_MYELOID_CELL_DEVELOPMENT_UP	121	0.23	2.97	<0.001	6.26E-05
GSE3039_NKT_CELL_VS_B2_BCELL_DN	158	0.20	2.97	<0.001	6.28E-05
GSE31082_DP_VS_CD8_SP_THYMOCYTE_DN	170	0.19	2.97	<0.001	6.30E-05
GSE27241_WT_VS_RORGT_KO_TH17_POLARIZED_CD4_TCELL_UP	131	0.22	2.98	<0.001	5.99E-05
DUTERTRE_ESTRADIOL_RESPONSE_24HR_UP	273	0.16	2.98	<0.001	6.01E-05
MCLACHLAN_DENTAL_CARIES_UP	179	0.19	2.98	<0.001	6.02E-05
HALLMARK_INFLAMMATORY_RESPONSE	155	0.21	2.98	<0.001	6.04E-05
GO_ANATOMICAL_STRUCTURE_FORMATION_INVOLVED_IN_MORPHOGENESIS	717	0.10	2.98	<0.001	5.74E-05
GSE30083_SP3_VS_SP4_THYMOCYTE_DN	161	0.20	2.99	<0.001	5.11E-05
GO_POSITIVE_REGULATION_OF_I_KAPPAB_KINASE_NF_KAPPAB_SIGNALING	145	0.21	2.99	<0.001	5.13E-05
GO_PATTERNING_OF_BLOOD_VESSELS	24	0.52	2.99	<0.001	5.14E-05
REACTOME_PEPTIDE_CHAIN_ELONGATION	51	0.36	2.99	<0.001	5.16E-05
HALLMARK_EPITHELIAL_MESENCHYMAL_TRANSITION	174	0.19	2.99	<0.001	5.17E-05

GSE43955_TH0_VS_TGFB_IL6_IL23_TH17_ACT_CD4_TCELL_52H_DN	168	0.20	3.00	<0.001	5.18E-05
DODD_NASOPHARYNGEAL_CARCINOMA_DN	1138	0.08	3.00	<0.001	5.20E-05
GO_REGULATION_OF_CELL_DIFFERENTIATION	1051	0.08	3.00	<0.001	4.89E-05
GSE16755_CTRL_VS_IFNA_TREATED_MAC_DN	158	0.21	3.00	<0.001	4.90E-05
GSE41867_NAIVE_VS_DAY6_LCMV_EFFECTOR_CD8_TCELL_UP	181	0.19	3.00	<0.001	4.91E-05
SARTIPY_NORMAL_AT_INSULIN_RESISTANCE_UP	32	0.45	3.00	<0.001	4.93E-05
KANG_DOXORUBICIN_RESISTANCE_UP	48	0.37	3.00	<0.001	4.94E-05
GSE26343_UNSTIM_VS_LPS_STIM_MACROPHAGE_UP	172	0.20	3.01	<0.001	4.29E-05
REACTOME_INTEGRIN_CELL_SURFACE_INTERACTIONS	73	0.30	3.01	<0.001	4.30E-05
GO_ACTIN_BINDING	324	0.15	3.01	<0.001	4.31E-05
GO_CELLULAR_RESPONSE_TO_ORGANIC_SUBSTANCE	1332	0.08	3.01	<0.001	4.33E-05
GSE31082_DP_VS_CD4_SP_THYMOCYTE_DN	168	0.20	3.01	<0.001	4.00E-05
GSE17721_LPS_VS_PAM3CSK4_12H_BMDC_UP	171	0.20	3.01	<0.001	3.68E-05
GSE37301_PRO_BCELL_VS_RAG2_KO_NK_CELL_DN	155	0.21	3.02	<0.001	3.69E-05
GSE34205_HEALTHY_VS_FLU_INF_INFANT_PBMC_DN	143	0.22	3.02	<0.001	3.70E-05
GSE1432_CTRL_VS_IFNG_6H_MICROGLIA_DN	159	0.21	3.02	<0.001	3.71E-05
GO_POSITIVE_REGULATION_OF_DEVELOPMENTAL_PROCESS	810	0.09	3.02	<0.001	3.72E-05
FULCHER_INFLAMMATORY_RESPONSE_LECTIN_VS_LPS_UP	460	0.13	3.02	<0.001	3.05E-05
GSE18893_TCONV_VS_TREG_2H_CULTURE_DN	181	0.19	3.02	<0.001	3.06E-05
GSE14769_UNSTIM_VS_120MIN_LPS_BMDM_DN	178	0.20	3.03	<0.001	3.06E-05
GO_REGULATION_OF_VASCULATURE_DEVELOPMENT	186	0.19	3.03	<0.001	2.73E-05
GSE10325_BCELL_VS_MYELOID_DN	153	0.22	3.03	<0.001	2.74E-05
GO_INTEGRIN_BINDING	89	0.27	3.04	<0.001	2.75E-05
GO_MYELOID_LEUKOCYTE_ACTIVATION	72	0.30	3.04	<0.001	2.75E-05
GSE11961_MEMORY_BCELL_DAY7_VS_GERMINAL_CENTER_BCELL_DAY40_UP	154	0.21	3.04	<0.001	2.76E-05
GSE9988_LOW_LPS_VS_VEHICLE_TREATED_MONOCYTE_DN	176	0.19	3.04	<0.001	2.77E-05
BURTON_ADIPOGENESIS_3	93	0.28	3.04	<0.001	2.78E-05
KOINUMA_TARGETS_OF_SMAD2_OR_SMAD3	700	0.10	3.04	<0.001	2.78E-05
GNF2_HCK	71	0.30	3.05	<0.001	2.79E-05
GNF2_CARD15	53	0.36	3.05	<0.001	2.80E-05
ODONNELL_TFRC_TARGETS_DN	98	0.27	3.05	<0.001	2.81E-05
GSE10239_NAIVE_VS_KLRG1HIGH_EFF_CD8_TCELL_DN	171	0.20	3.05	<0.001	2.82E-05
NABA_MATRISOME	518	0.12	3.05	<0.001	2.83E-05
GO_TISSUE_DEVELOPMENT	1027	0.08	3.05	<0.001	2.83E-05

LY_AGING_MIDDLE_DN	15	0.65	3.05	<0.001	2.84E-05
GSE26030_UNSTIM_VS_RESTIM_TH1_DAY15_POST_POLARIZATION_UP	174	0.20	3.05	<0.001	2.85E-05
GNF2_CDC2	59	0.34	3.06	<0.001	2.86E-05
ICHIBA_GRAFT_VERSUS_HOST_DISEASE_35D_UP	102	0.26	3.06	<0.001	2.87E-05
KARLSSON_TGFB1_TARGETS_UP	114	0.24	3.06	<0.001	2.88E-05
GSE17721_PAM3CSK4_VS_GADIQUIMOD_4H_BMDC_DN	164	0.21	3.06	<0.001	2.88E-05
MODULE_5	364	0.14	3.06	<0.001	2.89E-05
PILON_KLF1_TARGETS_DN	1829	0.07	3.06	<0.001	2.90E-05
GO_EPITHELIUM_DEVELOPMENT	614	0.11	3.06	<0.001	2.91E-05
JACKSON_DNMT1_TARGETS_UP	69	0.31	3.06	<0.001	2.92E-05
GSE37532_TREG_VS_TCONV_CD4_TCELL_FROM_LN_DN	180	0.20	3.06	<0.001	2.93E-05
NABA_COLLAGENS	32	0.45	3.07	<0.001	2.94E-05
GSE21360_NAIVE_VS_SECONDARY_MEMORY_CD8_TCELL_UP	151	0.21	3.07	<0.001	2.95E-05
ACEVEDO_FGFR1_TARGETS_IN_PROSTATE_CANCER_MODEL_UP	209	0.18	3.07	<0.001	2.96E-05
GSE21927_SPLEEN_C57BL6_VS_4T1_TUMOR_BALBC_MONOCYTES_DN	157	0.21	3.07	<0.001	2.96E-05
EGFR_UP.V1_UP	161	0.21	3.07	<0.001	2.97E-05
GSE7460_TCONV_VS_TREG_THYMUS_DN	154	0.21	3.08	<0.001	2.98E-05
KRIEG_HYPOXIA_NOT_VIA_KDM3A	615	0.11	3.08	<0.001	2.99E-05
GO_REGULATION_OF_PROTEIN_SERINE_THREONINE_KINASE_ACTIVITY	382	0.14	3.08	<0.001	3.00E-05
GNF2_CDH11	25	0.51	3.08	<0.001	3.01E-05
GO_PLATELET_ACTIVATION	113	0.25	3.08	<0.001	3.02E-05
GO_BASAL_PART_OF_CELL	35	0.44	3.09	<0.001	3.03E-05
GSE6092_IFNG_VS_IFNG_AND_B_BURGDORFERI_INF_ENDOTHELIAL_CELL_DN	164	0.21	3.10	<0.001	3.04E-05
GNF2_CENPF	59	0.35	3.10	<0.001	3.05E-05
GSE37416_12H_VS_24H_F_TULARENSIS_LVS_NEUTROPHIL_UP	171	0.20	3.10	<0.001	3.06E-05
JOHANSSON_GLIOMAGENESIS_BY_PDGFB_UP	53	0.35	3.10	<0.001	3.07E-05
GSE19198_CTRL_VS_IL21_TREATED_TCELL_6H_UP	176	0.21	3.10	<0.001	2.31E-05
GSE2770_TGFB_AND_IL4_ACT_VS_ACT_CD4_TCELL_2H_DN	158	0.21	3.10	<0.001	2.32E-05
GSE9988_LPS_VS_VEHICLE_TREATED_MONOCYTE_DN	177	0.20	3.11	<0.001	2.33E-05
GSE1791_CTRL_VS_NEUROMEDINU_IN_T_CELL_LINE_3H_UP	114	0.26	3.11	<0.001	2.33E-05
SENESE_HDAC3_TARGETS_UP	416	0.13	3.11	<0.001	2.34E-05
KAECH_NAIVE_VS_DAY8_EFF_CD8_TCELL_DN	166	0.20	3.12	<0.001	2.35E-05
GSE9988_ANTI_TREM1_VS_VEHICLE_TREATED_MONOCYTES_UP	143	0.22	3.12	<0.001	2.36E-05
BROWNE_INTERFERON_RESPONSIVE_GENES	51	0.37	3.12	<0.001	2.36E-05

REACTOME_HEMOSTASIS	344	0.14	3.12	<0.001	2.37E-05
GSE42021_TREG_PLN_VS_CD24INT_TREG_THYMUS_DN	148	0.22	3.13	<0.001	2.38E-05
GNF2_FGR	28	0.50	3.13	<0.001	2.39E-05
GSE23925_DARK_ZONE_VS_NAIVE_BCELL_DN	151	0.22	3.13	<0.001	2.39E-05
GSE7768_OVA_ALONE_VS_OVA_WITH_LPS_IMMUNIZED_MOUSE_WHOLE_SPLE	135	0.23	3.14	<0.001	2.01E-05
GO_CIRCULATORY_SYSTEM_DEVELOPMENT	655	0.11	3.14	<0.001	2.01E-05
GSE42021_TREG_VS_TCONV_PLN_UP	145	0.22	3.14	<0.001	2.02E-05
CHARAFE_BREAST_CANCER_LUMINAL_VS_BASAL_DN	352	0.15	3.15	<0.001	2.03E-05
POOLA_INVASIVE_BREAST_CANCER_UP	195	0.19	3.15	<0.001	2.03E-05
GSE9988_ANTI_TREM1_VS_VEHICLE_TREATED_MONOCYTES_DN	178	0.20	3.16	<0.001	2.04E-05
HALLMARK_G2M_CHECKPOINT	187	0.20	3.16	<0.001	2.05E-05
GO_FC_GAMMA_RECEPTOR_SIGNALING_PATHWAY	71	0.32	3.16	<0.001	2.05E-05
CROMER_TUMORIGENESIS_UP	45	0.40	3.16	<0.001	2.06E-05
GO_DEFENSE_RESPONSE	696	0.11	3.16	<0.001	2.07E-05
GOTZMANN_EPITHELIAL_TO_MESENCHYMAL_TRANSITION_UP	65	0.33	3.17	<0.001	2.08E-05
GSE24142_EARLY_THYMIC_PROGENITOR_VS_DN3_THYMOCYTE_UP	174	0.21	3.17	<0.001	2.08E-05
GNF2_MKI67	27	0.50	3.17	<0.001	2.09E-05
GO_REGULATION_OF_INTRACELLULAR_SIGNAL_TRANSDUCTION	1273	0.08	3.17	<0.001	2.10E-05
GSE36009_WT_VS_NLRP10_KO_DC_DN	184	0.20	3.17	<0.001	2.10E-05
GSE22886_NAIVE_BCELL_VS_MONOCYTE_DN	177	0.20	3.18	<0.001	2.11E-05
REACTOME_3_UTR_MEDIATED_TRANSLATIONAL_REGULATION	67	0.34	3.18	<0.001	2.12E-05
GO_VASCULAR_ENDOTHELIAL_GROWTH_FACTOR_RECEPTOR_SIGNALING_PATH	71	0.31	3.18	<0.001	2.13E-05
GOLDRATH_ANTIGEN_RESPONSE	290	0.17	3.18	<0.001	2.13E-05
GROSS_HYPOXIA_VIA_ELK3_AND_HIF1A_UP	129	0.24	3.18	<0.001	2.14E-05
UZONYI_RESPONSE_TO_LEUKOTRIENE_AND_THROMBIN	36	0.45	3.18	<0.001	2.15E-05
PETROVA_ENDOTHELIUM_LYMPHATIC_VS_BLOOD_UP	115	0.26	3.18	<0.001	2.16E-05
GSE9988_ANTI_TREM1_VS_CTRL_TREATED_MONOCYTES_DN	177	0.20	3.18	<0.001	2.16E-05
GSE2706_UNSTIM_VS_8H_LPS_AND_R848_DC_DN	138	0.23	3.19	<0.001	1.74E-05
GO_REGULATION_OF_GTPASE_ACTIVITY	531	0.12	3.19	<0.001	1.75E-05
GO_POSITIVE_REGULATION_OF_IMMUNE_RESPONSE	393	0.14	3.19	<0.001	1.75E-05
BENNETT_SYSTEMIC_LUPUS_ERYTHEMATOSUS	22	0.58	3.19	<0.001	1.76E-05
GSE37533_PPARG1_FOXP3_VS_FOXP3_TRANSDUCED_CD4_TCELL_PIOGLITAZON	161	0.21	3.19	<0.001	1.77E-05
HELLER_SILENCED_BY_METHYLATION_UP	181	0.21	3.19	<0.001	1.77E-05
GSE43863_DAY6_EFF_VS_DAY150_MEM_TH1_CD4_TCELL_UP	160	0.22	3.20	<0.001	1.78E-05

GSE34156_NOD2_LIGAND_VS_TLR1_TLR2_LIGAND_6H_TREATED_MONOCYTE_	149	0.23	3.20	<0.001	1.78E-05
GSE21063_CTRL_VS_ANTI_IGM_STIM_BCELL_8H_UP	116	0.25	3.20	<0.001	1.79E-05
GSE37301_LYMPHOID_PRIMED_MPP_VS_COMMON_LYMPHOID_PROGENITOR_	165	0.21	3.20	<0.001	1.80E-05
MODULE_3	356	0.15	3.21	<0.001	1.80E-05
GSE5589_IL6_KO_VS_IL10_KO_LPS_AND_IL10_STIM_MACROPHAGE_45MIN_UP	170	0.21	3.21	<0.001	1.81E-05
GO_DEFENSE_RESPONSE_TO_VIRUS	105	0.26	3.21	<0.001	1.82E-05
GSE37532_TREG_VS_TCONV_PPARG_KO_CD4_TCELL_FROM_LN_DN	170	0.21	3.21	<0.001	1.82E-05
GSE41867_NAIVE_VS_DAY30_LCMV_CLONE13_EXHAUSTED_CD8_TCELL_UP	138	0.23	3.21	<0.001	1.83E-05
GSE21360_NAIVE_VS_QUATERNARY_MEMORY_CD8_TCELL_DN	160	0.22	3.21	<0.001	1.84E-05
GO_RESPONSE_TO_CYTOKINE	485	0.13	3.21	<0.001	1.85E-05
QI_PLASMACYTOMA_UP	200	0.20	3.21	<0.001	1.85E-05
GSE12392_IFNAR_KO_VS_IFNB_KO_CD8_NEG_SPLEEN_DC_DN	152	0.23	3.21	<0.001	1.86E-05
GSE23925_LIGHT_ZONE_VS_NAIVE_BCELL_UP	158	0.22	3.21	<0.001	1.87E-05
GO_REGULATION_OF_MULTICELLULAR_ORGANISMAL_DEVELOPMENT	1199	0.08	3.22	<0.001	1.87E-05
MARTENS_BOUND_BY_PML_RARA_FUSION	378	0.15	3.22	<0.001	1.88E-05
GSE13547_CTRL_VS_ANTI_IGM_STIM_BCELL_12H_UP	159	0.22	3.22	<0.001	1.89E-05
GO_ACTIVATION_OF_IMMUNE_RESPONSE	311	0.16	3.22	<0.001	1.42E-05
GSE26495_NAIVE_VS_PD1LOW_CD8_TCELL_DN	156	0.22	3.23	<0.001	1.43E-05
GSE30962_ACUTE_VS_CHRONIC_LCMV_PRIMARY_INF_CD8_TCELL_DN	168	0.22	3.23	<0.001	1.43E-05
GSE27241_CTRL_VS_DIGOXIN_TREATED_CD4_TCELL_IN_TH17_POLARIZING_CO	132	0.24	3.23	<0.001	1.44E-05
HALLMARK_MITOTIC_SPINDLE	194	0.20	3.23	<0.001	1.44E-05
GSE42021_TREG_PLN_VS_CD24LO_TREG_THYMUS_DN	130	0.24	3.23	<0.001	1.45E-05
KEGG_FOCAL_ADHESION	182	0.20	3.23	<0.001	1.46E-05
GO_REGULATION_OF_ANATOMICAL_STRUCTURE_MORPHOGENESIS	803	0.10	3.24	<0.001	1.46E-05
GNF2_ITGB2	52	0.39	3.24	<0.001	1.47E-05
GSE9601_NFKB_INHIBITOR_VS_PI3K_INHIBITOR_TREATED_HCMV_INF_MONOC	155	0.22	3.24	<0.001	1.47E-05
GSE46606_UNSTIM_VS_CD40L_IL2_IL5_1DAY_STIMULATED_IRF4HIGH_SORTED_	163	0.22	3.24	<0.001	1.48E-05
GSE22103_UNSTIM_VS_LPS_STIM_NEUTROPHIL_UP	185	0.21	3.24	<0.001	9.94E-06
GSE42724_NAIVE_BCELL_VS_PLASMABLAST_UP	153	0.22	3.24	<0.001	9.98E-06
GSE18791_UNSTIM_VS_NEWCATSLE_VIRUS_DC_6H_DN	134	0.24	3.24	<0.001	1.00E-05
GSE19888_ADENOSINE_A3R_ACT_VS_TCELL_MEMBRANES_ACT_IN_MAST_CELL	168	0.22	3.24	<0.001	1.01E-05
GSE18804_SPLEEN_MACROPHAGE_VS_COLON_TUMORAL_MACROPHAGE_UP	161	0.22	3.25	<0.001	1.01E-05
GO_POSITIVE_REGULATION_OF_INTRACELLULAR_SIGNAL_TRANSDUCTION	653	0.11	3.25	<0.001	1.01E-05
GO_REGULATION_OF_CELL_ADHESION	456	0.13	3.25	<0.001	1.02E-05

GO_ACTOMYOSIN	58	0.36	3.25	<0.001	1.02E-05
GSE18791_CTRL_VS_NEWCASTLE_VIRUS_DC_4H_DN	132	0.25	3.25	<0.001	1.03E-05
GSE2706_UNSTIM_VS_8H_R848_DC_DN	142	0.24	3.25	<0.001	1.03E-05
NAKAYAMA_SOFT_TISSUE_TUMORS_PCA2_UP	63	0.34	3.26	<0.001	1.04E-05
GO_HEMOSTASIS	217	0.19	3.26	<0.001	1.04E-05
GSE13522_CTRL_VS_T_CRUZI_Y_STRAIN_INF_SKIN_IFNAR_KO_DN	161	0.23	3.26	<0.001	1.04E-05
GNF2_CENPE	39	0.44	3.26	<0.001	1.05E-05
GSE36891_UNSTIM_VS_POLYIC_TLR3_STIM_PERITONEAL_MACROPHAGE_UP	106	0.27	3.27	<0.001	1.05E-05
GSE37534_UNTREATED_VS_ROSIGLITAZONE_TREATED_CD4_TCELL_PPARG1_AN	157	0.23	3.27	<0.001	1.06E-05
GO_POSITIVE_REGULATION_OF_CELL_COMMUNICATION	1148	0.09	3.27	<0.001	1.06E-05
GROSS_HYPOXIA_VIA_ELK3_DN	139	0.24	3.27	<0.001	1.07E-05
PICCALUGA_ANGIOIMMUNOBLASTIC_LYMPHOMA_UP	182	0.21	3.28	<0.001	1.07E-05
CROONQUIST_IL6_DEPRIVATION_DN	91	0.29	3.28	<0.001	1.08E-05
CUI_TCF21_TARGETS_2_DN	757	0.11	3.28	<0.001	1.08E-05
GSE19198_1H_VS_6H_IL21_TREATED_TCELL_DN	174	0.21	3.29	<0.001	1.09E-05
ZHOU_CELL_CYCLE_GENES_IN_IR_RESPONSE_24HR	112	0.26	3.29	<0.001	5.48E-06
GSE2405_S_AUREUS_VS_A_PHAGOCYTOPHILUM_NEUTROPHIL_UP	148	0.23	3.29	<0.001	5.50E-06
GSE9988_LOW_LPS_VS_VEHICLE_TREATED_MONOCYTE_UP	136	0.24	3.30	<0.001	5.53E-06
GSE18791_CTRL_VS_NEWCASTLE_VIRUS_DC_10H_DN	153	0.23	3.30	<0.001	5.55E-06
MODULE_118	316	0.16	3.30	<0.001	5.58E-06
GSE46606_IRF4MID_VS_WT_CD40L_IL2_IL5_DAY1_STIMULATED_BCELL_DN	141	0.24	3.30	<0.001	5.60E-06
GSE3982_NEUTROPHIL_VS_EFF_MEMORY_CD4_TCELL_UP	162	0.22	3.30	<0.001	5.63E-06
KEGG_FC_GAMMA_R_MEDIATED_PHAGOCYTOSIS	88	0.30	3.30	<0.001	5.65E-06
GSE19923_WT_VS_HEB_AND_E2A_KO_DP_THYMOCYTE_DN	169	0.22	3.31	<0.001	5.68E-06
BURTON_ADIPOGENESIS_PEAK_AT_2HR	48	0.41	3.31	<0.001	5.71E-06
GSE29618_PDC_VS_MDC_DN	161	0.22	3.31	<0.001	5.73E-06
MODULE_54	220	0.19	3.31	<0.001	5.76E-06
GSE36826_WT_VS_IL1R_KO_SKIN_STAPH_AUREUS_INF_DN	163	0.22	3.32	<0.001	5.79E-06
GSE27434_WT_VS_DNMT1_KO_TREG_DN	179	0.21	3.33	<0.001	<0.001
GO_ACTIN_FILAMENT_BUNDLE	51	0.39	3.33	<0.001	<0.001
GO_LAMELLIPODIUM	157	0.23	3.33	<0.001	<0.001
GSE22140_GERMFREE_VS_SPF_MOUSE_CD4_TCELL_UP	156	0.23	3.33	<0.001	<0.001
GSE9316_IL6_KO_VS_IFNG_KO_INVIVO_EXPANDED_CD4_TCELL_DN	166	0.22	3.33	<0.001	<0.001
ZHAN_MULTIPLE_MYELOMA_PR_UP	36	0.47	3.34	<0.001	<0.001

MILI_PSEUDOPODIA_HAPTOTAXIS_DN	617	0.12	3.34	<0.001	<0.001
GSE18791_CTRL_VS_NEWCASTLE_VIRUS_DC_8H_DN	137	0.25	3.34	<0.001	<0.001
GO_REGULATION_OF_RAS_PROTEIN_SIGNAL_TRANSDUCTION	152	0.23	3.34	<0.001	<0.001
GO_POSITIVE_REGULATION_OF_PHOSPHORUS_METABOLIC_PROCESS	735	0.11	3.35	<0.001	<0.001
GNF2_TNFRSF1B	52	0.39	3.35	<0.001	<0.001
GO_POSITIVE_REGULATION_OF_HYDROLASE_ACTIVITY	723	0.11	3.36	<0.001	<0.001
GOLDRATH_NAIVE_VS_EFF_CD8_TCELL_DN	167	0.23	3.36	<0.001	<0.001
GNF2_CCNA2	65	0.36	3.37	<0.001	<0.001
GSE7509_DC_VS_MONOCYTE_WITH_FCGRIIB_STIM_DN	160	0.22	3.37	<0.001	<0.001
GSE19198_CTRL_VS_IL21_TREATED_TCELL_24H_UP	167	0.22	3.37	<0.001	<0.001
GSE2770_TGFB_AND_IL4_VS_IL12_TREATED_ACT_CD4_TCELL_2H_DN	149	0.24	3.37	<0.001	<0.001
WALLACE_PROSTATE_CANCER_RACE_UP	204	0.20	3.37	<0.001	<0.001
GSE29618_PDC_VS_MDC_DAY7_FLU_VACCINE_DN	161	0.23	3.37	<0.001	<0.001
PID_AVB3_INTEGRIN_PATHWAY	68	0.35	3.37	<0.001	<0.001
ZWANG_CLASS_1_TRANSIENTLY_INDUCED_BY_EGF	375	0.15	3.38	<0.001	<0.001
GSE13484_UNSTIM_VS_YF17D_VACCINE_STIM_PBMC_DN	151	0.24	3.38	<0.001	<0.001
GSE37533_PPARG1_FOXP3_VS_PPARG2_FOXP3_TRANSDUCED_CD4_TCELL_PIO	159	0.23	3.38	<0.001	<0.001
GAL_LEUKEMIC_STEM_CELL_DN	171	0.22	3.38	<0.001	<0.001
GSE42021_TCONV_PLN_VS_TREG_PRECURSORS_THYMUS_DN	179	0.22	3.38	<0.001	<0.001
GSE42021_CD24HI_TREG_VS_CD24HI_TCONV_THYMUS_DN	169	0.22	3.39	<0.001	<0.001
GO_REGULATION_OF_SMALL_GTPASE_MEDIATED_SIGNAL_TRANSDUCTION	237	0.19	3.39	<0.001	<0.001
GSE13485_PRE_VS_POST_YF17D_VACCINATION_PBMC_DN	158	0.23	3.40	<0.001	<0.001
LEE_EARLY_T_LYMPHOCYTE_UP	79	0.33	3.40	<0.001	<0.001
GSE9988_LPS_VS_VEHICLE_TREATED_MONOCYTE_UP	133	0.25	3.40	<0.001	<0.001
GSE13485_DAY1_VS_DAY7_YF17D_VACCINE_PBMC_DN	153	0.24	3.41	<0.001	<0.001
GSE29618_BCELL_VS_MDC_DN	177	0.22	3.41	<0.001	<0.001
GNF2_PCNA	64	0.37	3.42	<0.001	<0.001
KIM_WT1_TARGETS_8HR_UP	142	0.24	3.42	<0.001	<0.001
GOLDRATH_EFF_VS_MEMORY_CD8_TCELL_UP	173	0.22	3.42	<0.001	<0.001
GRAHAM_CML_DIVIDING_VS_NORMAL_QUIESCENT_UP	150	0.24	3.42	<0.001	<0.001
GO_CELL_LEADING_EDGE	308	0.17	3.43	<0.001	<0.001
MODULE_38	373	0.15	3.44	<0.001	<0.001
WU_APOPTOSIS_BY_CDKN1A_VIA_TP53	49	0.41	3.44	<0.001	<0.001
PHONG_TNF_TARGETS_UP	52	0.41	3.45	<0.001	<0.001

GO_REGULATION_OF_CELLULAR_COMPONENT_MOVEMENT	611	0.12	3.45	<0.001	<0.001
GSE19888_ADENOSINE_A3R_INH_VS_TCELL_MEMBRANES_ACT_MAST_CELL_UI	152	0.25	3.46	<0.001	<0.001
RUIZ_TNC_TARGETS_DN	122	0.27	3.46	<0.001	<0.001
MODULE_45	421	0.15	3.47	<0.001	<0.001
SMIRNOV_CIRCULATING_ENDOTHELIOCYTES_IN_CANCER_UP	132	0.26	3.48	<0.001	<0.001
GNF2_ESPL1	34	0.51	3.48	<0.001	<0.001
HOSHIDA_LIVER_CANCER_SUBCLASS_S1	211	0.21	3.48	<0.001	<0.001
GSE21379_WT_VS_SAP_KO_TFH_CD4_TCELL_UP	169	0.23	3.49	<0.001	<0.001
VERRECCHIA_EARLY_RESPONSE_TO_TGFB1	52	0.41	3.49	<0.001	<0.001
GO_CELL_ACTIVATION	404	0.15	3.49	<0.001	<0.001
GSE43955_1H_VS_10H_ACT_CD4_TCELL_WITH_TGFB_IL6_DN	161	0.24	3.50	<0.001	<0.001
SHEPARD_BMYB_TARGETS	53	0.41	3.50	<0.001	<0.001
GSE9988_ANTI_TREM1_VS_CTRL_TREATED_MONOCYTES_UP	158	0.23	3.50	<0.001	<0.001
LE_EGR2_TARGETS_UP	98	0.30	3.51	<0.001	<0.001
GSE42021_TREG_PLN_VS_TREG_PRECURSORS_THYMUS_DN	141	0.26	3.51	<0.001	<0.001
GSE42021_CD24HI_VS_CD24INT_TREG_THYMUS_DN	152	0.25	3.52	<0.001	<0.001
GSE33424_CD161_INT_VS_NEG_CD8_TCELL_UP	169	0.23	3.52	<0.001	<0.001
TGCTGAY_UNKNOWN	838	0.11	3.53	<0.001	<0.001
GNF2_RRM2	39	0.46	3.53	<0.001	<0.001
TANG_SENESCENCE_TP53_TARGETS_DN	49	0.43	3.53	<0.001	<0.001
GSE22140_GERMFREE_VS_SPF_MOUSE_CD4_TCELL_DN	177	0.23	3.53	<0.001	<0.001
MOSERLE_IFNA_RESPONSE	20	0.64	3.54	<0.001	<0.001
GSE39110_DAY3_VS_DAY6_POST_IMMUNIZATION_CD8_TCELL_DN	159	0.24	3.54	<0.001	<0.001
GO_EXTRACELLULAR_STRUCTURE_ORGANIZATION	229	0.20	3.54	<0.001	<0.001
NADLER_OBESITY_UP	56	0.40	3.55	<0.001	<0.001
GSE25085_FETAL_BM_VS_ADULT_BM_SP4_THYMIC_IMPLANT_DN	177	0.23	3.55	<0.001	<0.001
GSE7219_UNSTIM_VS_LPS_AND_ANTI_CD40_STIM_NIK_NFKB2_KO_DC_DN	170	0.24	3.55	<0.001	<0.001
GSE19888_CTRL_VS_TCELL_MEMBRANES_ACT_MAST_CELL_PRETREAT_A3R_INI	166	0.24	3.55	<0.001	<0.001
MODULE_84	387	0.16	3.55	<0.001	<0.001
REACTOME_PLATELET_ACTIVATION_SIGNALING_AND_AGGREGATION	164	0.24	3.56	<0.001	<0.001
GO_LOCOMOTION	752	0.12	3.56	<0.001	<0.001
GSE46606_UNSTIM_VS_CD40L_IL2_IL5_3DAY_STIMULATED_IRF4MID_SORTED_	167	0.24	3.56	<0.001	<0.001
GO_POSITIVE_REGULATION_OF_MULTICELLULAR_ORGANISMAL_PROCESS	996	0.10	3.56	<0.001	<0.001
WONG_ADULT_TISSUE_STEM_MODULE	603	0.13	3.57	<0.001	<0.001

GSE13485_CTRL_VS_DAY3_YF17D_VACCINE_PBMC_DN	154	0.25	3.58	<0.001	<0.001
GSE26343_UNSTIM_VS_LPS_STIM_NFAT5_KO_MACROPHAGE_DN	166	0.24	3.58	<0.001	<0.001
GO_POSITIVE_REGULATION_OF_CATALYTIC_ACTIVITY	1196	0.09	3.58	<0.001	<0.001
GNF2_PECAM1	45	0.45	3.58	<0.001	<0.001
ZHENG_GLIOBLASTOMA_PLASTICITY_UP	206	0.22	3.58	<0.001	<0.001
GO_POSITIVE_REGULATION_OF_MOLECULAR_FUNCTION	1403	0.09	3.59	<0.001	<0.001
GSE32533_MIR17_KO_VS_MIR17_OVEREXPRESS_ACT_CD4_TCELL_UP	153	0.25	3.59	<0.001	<0.001
CROONQUIST_NRAS_VS_STROMAL_STIMULATION_DN	88	0.33	3.60	<0.001	<0.001
REACTOME_AXON_GUIDANCE	208	0.21	3.60	<0.001	<0.001
GSE30962_PRIMARY_VS_SECONDARY_ACUTE_LCMV_INF_CD8_TCELL_UP	162	0.25	3.62	<0.001	<0.001
HALLMARK_INTERFERON_ALPHA_RESPONSE	84	0.34	3.62	<0.001	<0.001
GSE37533_PPARG2_FOXP3_VS_FOXP3_TRANSDUCED_CD4_TCELL_DN	154	0.25	3.63	<0.001	<0.001
BOSCO_TH1_CYTOTOXIC_MODULE	59	0.39	3.63	<0.001	<0.001
GSE21546_UNSTIM_VS_ANTI_CD3_STIM_ELK1_KO_DP_THYMOCYTES_UP	100	0.31	3.63	<0.001	<0.001
GO_INNATE_IMMUNE_RESPONSE	336	0.17	3.63	<0.001	<0.001
GO_REGULATION_OF_CYTOSKELETON_ORGANIZATION	425	0.15	3.64	<0.001	<0.001
GO_POSITIVE_REGULATION_OF_CYTOKINE_PRODUCTION	261	0.19	3.64	<0.001	<0.001
GSE6674_ANTI_IGM_VS_CPG_STIM_BCELL_DN	174	0.24	3.65	<0.001	<0.001
KONG_E2F3_TARGETS	79	0.34	3.65	<0.001	<0.001
GSE21546_WT_VS_SAP1A_KO_DP_THYMOCYTES_UP	138	0.26	3.65	<0.001	<0.001
ALTEMEIER_RESPONSE_TO_LPS_WITH_MECHANICAL_VENTILATION	96	0.32	3.65	<0.001	<0.001
GNF2_HMMR	46	0.44	3.66	<0.001	<0.001
GSE22601_IMMATURE_CD4_SINGLE_POSITIVE_VS_CD8_SINGLE_POSITIVE_THYM	179	0.23	3.66	<0.001	<0.001
SARRIO_EPITHELIAL_MESENCHYMAL_TRANSITION_UP	147	0.27	3.66	<0.001	<0.001
GSE22140_HEALTHY_VS_ARTHRITIC_GERMFREE_MOUSE_CD4_TCELL_DN	175	0.24	3.66	<0.001	<0.001
GO_REGULATION_OF_IMMUNE_SYSTEM_PROCESS	955	0.11	3.69	<0.001	<0.001
GSE14415_NATURAL_TREG_VS_TCONV_DN	148	0.26	3.71	<0.001	<0.001
MODULE_47	196	0.23	3.71	<0.001	<0.001
GSE6259_FLT3L_INDUCED_DEC205_POS_DC_VS_CD8_TCELL_DN	146	0.26	3.71	<0.001	<0.001
GSE34156_TLR1_TLR2_LIGAND_VS_NOD2_AND_TLR1_TLR2_LIGAND_24H_TREA	156	0.26	3.71	<0.001	<0.001
GSE19401_NAIVE_VS_IMMUNIZED_MOUSE_PLN_FOLLICULAR_DC_UP	168	0.25	3.72	<0.001	<0.001
GSE32986_CURDLAN_HIGHDOSE_VS_GMCSF_AND_CURDLAN_HIGHDOSE_STIM	165	0.25	3.72	<0.001	<0.001
SOTIRIOU_BREAST_CANCER_GRADE_1_VS_3_UP	134	0.28	3.74	<0.001	<0.001
GO_CELL_MOTILITY	578	0.14	3.74	<0.001	<0.001

GSE37533_PPARG1_FOXP3_VS_FOXP3_TRANSDUCED_CD4_TCELL_DN	154	0.26	3.74	<0.001	<0.001
GSE37605_FOXP3_FUSION_GFP_VS_IRES_GFP_TREG_C57BL6_UP	165	0.25	3.75	<0.001	<0.001
GSE14769_UNSTIM_VS_80MIN_LPS_BMDM_DN	165	0.25	3.75	<0.001	<0.001
GSE40274_CTRL_VS_EOS_TRANSDUCED_ACTIVATED_CD4_TCELL_UP	143	0.27	3.76	<0.001	<0.001
CHICAS_RB1_TARGETS_CONFLUENT	465	0.15	3.76	<0.001	<0.001
GSE18791_CTRL_VS_NEWCASTLE_VIRUS_DC_6H_DN	159	0.25	3.77	<0.001	<0.001
GSE13547_CTRL_VS_ANTI_IGM_STIM_BCELL_2H_UP	157	0.26	3.77	<0.001	<0.001
GSE14769_UNSTIM_VS_60MIN_LPS_BMDM_DN	169	0.25	3.77	<0.001	<0.001
GSE37605_C57BL6_VS_NOD_FOXP3_FUSION_GFP_TREG_DN	161	0.26	3.78	<0.001	<0.001
GSE35825_UNTREATED_VS_IFNG_STIM_MACROPHAGE_UP	172	0.25	3.78	<0.001	<0.001
GSE37534_UNTREATED_VS_PIOGLITAZONE_TREATED_CD4_TCELL_PPARG1_ANI	168	0.25	3.78	<0.001	<0.001
GO_BIOLOGICAL_ADHESION	672	0.13	3.79	<0.001	<0.001
GO_WOUND_HEALING	334	0.18	3.79	<0.001	<0.001
CHIANG_LIVER_CANCER_SUBCLASS_PROLIFERATION_UP	147	0.27	3.80	<0.001	<0.001
GSE35685_CD34POS_CD10NEG_CD62LPOS_VS_CD34POS_CD10POS_BONE_MAI	173	0.24	3.81	<0.001	<0.001
GSE36527_CD69_NEG_VS_POS_TREG_CD62L_LOS_KLRG1_NEG_UP	165	0.25	3.82	<0.001	<0.001
GO_POSITIVE_REGULATION_OF_IMMUNE_SYSTEM_PROCESS	612	0.14	3.82	<0.001	<0.001
GSE18281_CORTICAL_VS_MEDULLARY_THYMOCYTE_UP	164	0.26	3.83	<0.001	<0.001
MEISSNER_BRAIN_HCP_WITH_H3K4ME3_AND_H3K27ME3	674	0.13	3.83	<0.001	<0.001
GO_CYTOSKELETON_ORGANIZATION	674	0.13	3.84	<0.001	<0.001
HALLMARK_INTERFERON_GAMMA_RESPONSE	175	0.26	3.84	<0.001	<0.001
LINDGREN_BLADDER_CANCER_CLUSTER_2B	323	0.19	3.84	<0.001	<0.001
BERENJENO_TRANSFORMED_BY_RHOA_UP	489	0.15	3.85	<0.001	<0.001
GO_RESPONSE_TO_WOUNDING	401	0.17	3.85	<0.001	<0.001
ZHANG_RESPONSE_TO_IKK_INHIBITOR_AND_TNF_UP	175	0.25	3.86	<0.001	<0.001
GO_REGULATION_OF_CYTOKINE_PRODUCTION	396	0.17	3.87	<0.001	<0.001
HECKER_IFNB1_TARGETS	61	0.43	3.89	<0.001	<0.001
GSE13485_DAY3_VS_DAY7_YF17D_VACCINE_PBMC_DN	125	0.30	3.89	<0.001	<0.001
GSE37301_HEMATOPOIETIC_STEM_CELL_VS_CD4_TCELL_UP	145	0.28	3.89	<0.001	<0.001
LI_INDUCED_T_TO_NATURAL_KILLER_UP	237	0.22	3.89	<0.001	<0.001
ZWANG_CLASS_3_TRANSIENTLY_INDUCED_BY_EGF	183	0.25	3.89	<0.001	<0.001
LINDGREN_BLADDER_CANCER_CLUSTER_1_DN	323	0.19	3.90	<0.001	<0.001
GOBERT_OLIGODENDROCYTE_DIFFERENTIATION_UP	499	0.16	3.92	<0.001	<0.001
GSE26030_TH1_VS_TH17_DAY5_POST_POLARIZATION_UP	161	0.27	3.92	<0.001	<0.001

GNF2_CCNB2	54	0.45	3.93	<0.001	<0.001
GO_VASCULATURE_DEVELOPMENT	393	0.18	3.94	<0.001	<0.001
RICKMAN_METASTASIS_DN	198	0.24	3.94	<0.001	<0.001
GNF2_STAT6	66	0.42	3.94	<0.001	<0.001
AMIT_EGF_RESPONSE_480_HELA	145	0.28	3.94	<0.001	<0.001
GO_IMMUNE_RESPONSE	611	0.14	3.95	<0.001	<0.001
NUYTTEN_EZH2_TARGETS_UP	854	0.12	3.96	<0.001	<0.001
GAVIN_FOXP3_TARGETS_CLUSTER_P6	74	0.40	3.97	<0.001	<0.001
PHONG_TNF_RESPONSE_NOT_VIA_P38	293	0.20	3.98	<0.001	<0.001
GO_ENDOCYTOSIS	390	0.18	3.99	<0.001	<0.001
GO_POSITIVE_REGULATION_OF_RESPONSE_TO_STIMULUS	1405	0.10	4.02	<0.001	<0.001
GNF2_CDC20	54	0.47	4.03	<0.001	<0.001
GNF2_CASP1	91	0.36	4.03	<0.001	<0.001
GSE36891_POLYIC_TLR3_VS_PAM_TLR2_STIM_PERITONEAL_MACROPHAGE_UP	114	0.33	4.04	<0.001	<0.001
PLASARI_TGFB1_TARGETS_10HR_UP	166	0.27	4.05	<0.001	<0.001
PASINI_SUZ12_TARGETS_DN	280	0.21	4.05	<0.001	<0.001
GSE18791_UNSTIM_VS_NEWCATSLE_VIRUS_DC_10H_DN	140	0.30	4.05	<0.001	<0.001
VECCHI_GASTRIC_CANCER_EARLY_UP	300	0.20	4.05	<0.001	<0.001
GSE18281_SUBCAPSULAR_VS_CENTRAL_CORTICAL_REGION_OF_THYMUS_DN	174	0.26	4.06	<0.001	<0.001
GSE37605_TREG_VS_TCONV_NOD_FOXP3_FUSION_GFP_UP	106	0.34	4.06	<0.001	<0.001
GSE13485_DAY7_VS_DAY21_YF17D_VACCINE_PBMC_UP	163	0.28	4.06	<0.001	<0.001
GSE25085_FETAL_BM_VS_ADULT_BM_SP4_THYMIC_IMPLANT_UP	179	0.26	4.06	<0.001	<0.001
GSE22886_CTRL_VS_LPS_24H_DC_DN	151	0.29	4.08	<0.001	<0.001
RICKMAN_TUMOR_DIFFERENTIATED_WELL_VS_POORLY_DN	283	0.21	4.08	<0.001	<0.001
GO_REGULATION_OF_IMMUNE_RESPONSE	570	0.15	4.09	<0.001	<0.001
GSE35685_CD34POS_CD38NEG_VS_CD34POS_CD10POS_BONE_MARROW_DN	177	0.26	4.10	<0.001	<0.001
GSE15750_DAY6_VS_DAY10_TRAF6KO_EFF_CD8_TCELL_UP	161	0.28	4.13	<0.001	<0.001
GSE15750_DAY6_VS_DAY10_EFF_CD8_TCELL_UP	165	0.28	4.13	<0.001	<0.001
GO_IMMUNE_EFFECTOR_PROCESS	313	0.21	4.13	<0.001	<0.001
GSE2405_0H_VS_12H_A_PHAGOCYTOPHILUM_STIM_NEUTROPHIL_UP	166	0.27	4.14	<0.001	<0.001
GSE34392_ST2_KO_VS_WT_DAY8_LCMV_EFFECTOR_CD8_TCELL_DN	175	0.27	4.16	<0.001	<0.001
VERHAAK_GLIOBLASTOMA_MESENCHYMAL	187	0.26	4.17	<0.001	<0.001
GSE5589_LPS_AND_IL10_VS_LPS_AND_IL6_STIM_IL6_KO_MACROPHAGE_45MII	171	0.27	4.17	<0.001	<0.001
GO_MOVEMENT_OF_CELL_OR_SUBCELLULAR_COMPONENT	912	0.12	4.18	<0.001	<0.001

GSE13485_CTRL_VS_DAY7_YF17D_VACCINE_PBMC_DN	153	0.29	4.20	<0.001	<0.001
NEMETH_INFLAMMATORY_RESPONSE_LPS_UP	77	0.42	4.29	<0.001	<0.001
BASAKI_YBX1_TARGETS_UP	241	0.24	4.32	<0.001	<0.001
GSE2706_UNSTIM_VS_2H_LPS_DC_DN	139	0.31	4.33	<0.001	<0.001
GNF2_MYD88	53	0.49	4.37	<0.001	<0.001
GO_BLOOD_VESSEL_MORPHOGENESIS	305	0.22	4.48	<0.001	<0.001
GSE2706_UNSTIM_VS_2H_LPS_AND_R848_DC_DN	137	0.33	4.49	<0.001	<0.001
GSE19888_ADENOSINE_A3R_INH_VS_ACT_WITH_INHIBITOR_PRETREATMENT_I	120	0.35	4.52	<0.001	<0.001
KIM_WT1_TARGETS_UP	188	0.29	4.56	<0.001	<0.001
GO_ANGIOGENESIS	246	0.26	4.57	<0.001	<0.001
GSE22935_WT_VS_MYD88_KO_MACROPHAGE_UP	186	0.29	4.58	<0.001	<0.001
GSE13547_2H_VS_12_H_ANTI_IGM_STIM_BCELL_UP	143	0.33	4.60	<0.001	<0.001
GSE45365_NK_CELL_VS_BCELL_UP	184	0.30	4.64	<0.001	<0.001
SCHUETZ_BREAST_CANCER_DUCTAL_INVASIVE_UP	302	0.23	4.64	<0.001	<0.001
ROSTY_CERVICAL_CANCER_PROLIFERATION_CLUSTER	124	0.36	4.65	<0.001	<0.001
GSE2706_UNSTIM_VS_2H_R848_DC_DN	144	0.33	4.70	<0.001	<0.001
GO_IMMUNE_SYSTEM_PROCESS	1323	0.12	4.71	<0.001	<0.001
FOSTER_TOLERANT_MACROPHAGE_DN	372	0.22	4.75	<0.001	<0.001
GSE16266_CTRL_VS_LPS_STIM_MEF_UP	176	0.31	4.76	<0.001	<0.001
CHARAFE_BREAST_CANCER_LUMINAL_VS_MESENCHYMAL_DN	408	0.21	4.79	<0.001	<0.001
KOBAYASHI_EGFR_SIGNALING_24HR_DN	219	0.28	4.79	<0.001	<0.001
GO_CELL_JUNCTION	844	0.15	4.95	<0.001	<0.001
NAGASHIMA_EGF_SIGNALING_UP	51	0.59	4.97	<0.001	<0.001
GSE22935_WT_VS_MYD88_KO_MACROPHAGE_48H_MBOVIS_BCG_STIM_DN	179	0.32	5.03	<0.001	<0.001
REN_ALVEOLAR_RHABDOMYOSARCOMA_DN	390	0.22	5.05	<0.001	<0.001
GO_CELL_SUBSTRATE_JUNCTION	361	0.23	5.06	<0.001	<0.001
GO_ANCHORING_JUNCTION	431	0.22	5.16	<0.001	<0.001
GSE19888_ADENOSINE_A3R_INH_PRETREAT_AND_ACT_BY_A3R_VS_TCELL_ME	165	0.34	5.18	<0.001	<0.001
HALLMARK_TNFA_SIGNALING_VIA_NFKB	177	0.34	5.19	<0.001	<0.001
GSE45365_NK_CELL_VS_CD11B_DC_DN	172	0.35	5.24	<0.001	<0.001
NAGASHIMA_NRG1_SIGNALING_UP	146	0.37	5.30	<0.001	<0.001
RODWELL_AGING_KIDNEY_UP	371	0.25	5.64	<0.001	<0.001
BOQUEST_STEM_CELL_DN	181	0.37	5.70	<0.001	<0.001
CHEN_METABOLIC_SYNDROM_NETWORK	1013	0.18	6.37	<0.001	<0.001

Legend: ES, enrichment score; NES, normalized enrichment score; FDR, false discovery rate.
