# Supplementary material for: Age-Related Differential Structural and Transcriptomic Responses in the Hypertensive Heart
Source: Front Physiol. 2018 Jul 9;9:817. doi: 10.3389/fphys.2018.00817 (PMC6046461; doi:10.3389/fphys.2018.00817)
Supplement: Supplementary file 3 [file Table_10.PDF]

Table S10. MicroRNA that targets (based on the starBase database) are down-regulated in young and aged DOCA.

microRNA name	SetSize	P-value	FDR
hsa-miR-144	335	9.23E-11	5.77E-08
hsa-miR-101	244	3.33E-09	1.04E-06
hsa-miR-548N	395	6.80E-09	1.42E-06
hsa-miR-199A-3P	198	1.54E-07	2.41E-05
hsa-miR-561	270	3.01E-07	3.76E-05
hsa-miR-124	209	6.72E-07	5.27E-05
hsa-miR-15A	474	6.72E-07	5.27E-05
hsa-miR-107	444	6.75E-07	5.27E-05
hsa-miR-513A-3P	170	8.95E-07	6.22E-05
hsa-miR-559	281	1.85E-06	0.0001153
hsa-miR-103	442	2.14E-06	0.0001218
hsa-miR-380	105	2.45E-06	0.0001278
hsa-miR-511	143	3.09E-06	0.0001484
hsa-miR-30A	233	3.90E-06	0.000153
hsa-miR-30C	250	4.12E-06	0.000153
hsa-miR-199B-3P	158	4.12E-06	0.000153
hsa-miR-548M	197	4.16E-06	0.000153
hsa-miR-15B	440	5.22E-06	0.0001811
hsa-miR-300	164	7.12E-06	0.000234
hsa-miR-548D-3P	177	7.66E-06	0.0002394
hsa-miR-381	166	1.00E-05	0.0002919
hsa-miR-889	54	1.03E-05	0.0002919
hsa-miR-548L	233	1.09E-05	0.0002965
hsa-miR-576-5P	81	1.26E-05	0.000318
hsa-miR-16	453	1.27E-05	0.000318
hsa-miR-30E	280	1.37E-05	0.0003285
hsa-miR-548I	275	1.44E-05	0.0003333
hsa-miR-195	428	1.53E-05	0.0003426
hsa-miR-603	89	1.81E-05	0.0003686
hsa-miR-335	115	1.83E-05	0.0003686
hsa-miR-30D	238	1.83E-05	0.0003686
hsa-miR-29C	161	2.48E-05	0.000485
hsa-miR-338-5P	124	2.68E-05	0.000508
hsa-miR-543	108	2.92E-05	0.0005376
hsa-miR-30B	239	3.28E-05	0.0005729
hsa-miR-548P	236	3.30E-05	0.0005729
hsa-miR-424	402	3.58E-05	0.0005977
hsa-miR-515-3P	120	3.63E-05	0.0005977
hsa-miR-31	140	4.33E-05	0.000689
hsa-miR-548D-5P	307	4.41E-05	0.000689
hsa-miR-139-5P	142	5.13E-05	0.0007827
hsa-miR-20B	520	5.86E-05	0.0008715
hsa-miR-29A	152	6.05E-05	0.0008792
hsa-miR-17	498	7.22E-05	0.0010055
hsa-miR-548B-5P	307	7.24E-05	0.0010055
hsa-miR-892A	54	7.99E-05	0.0010862
hsa-miR-577	253	8.85E-05	0.0011766
hsa-miR-122	218	0.0001	0.0013034
hsa-miR-18B	260	0.000116	0.0014734
hsa-miR-330-3P	168	0.000131	0.0016396
hsa-miR-106A	478	0.000166	0.0020392
hsa-miR-19A	404	0.000183	0.0021991
hsa-miR-187	52	0.000192	0.0022619
hsa-miR-411	40	0.000205	0.0023643
hsa-miR-196B	147	0.000208	0.0023643
hsa-miR-221	174	0.000212	0.0023673

hsa-miR-298	146	0.000224	0.0024262
hsa-miR-181A	196	0.000225	0.0024262
hsa-miR-190	148	0.000246	0.0026078
hsa-miR-372	382	0.000252	0.0026205
hsa-miR-34C-5P	260	0.000287	0.002936
hsa-miR-494	131	0.000295	0.0029698
hsa-miR-320A	250	0.0003	0.002977
hsa-miR-181B	202	0.000338	0.0032998
hsa-miR-621	33	0.000357	0.003428
hsa-miR-1244	249	0.000389	0.0036875
hsa-miR-409-5P	46	0.000423	0.0039451
hsa-miR-301A	429	0.000448	0.0040596
hsa-miR-1305	62	0.000448	0.0040596
hsa-miR-497	379	0.00047	0.0041864
hsa-miR-194	118	0.000477	0.0041864
hsa-miR-20A	554	0.000482	0.0041864
hsa-miR-607	158	0.000516	0.0044202
hsa-miR-548J	254	0.000525	0.0044314
hsa-miR-570	257	0.000605	0.0050391
hsa-miR-802	143	0.00063	0.0051798
hsa-miR-491-5P	129	0.000646	0.0052436
hsa-miR-33B	98	0.000658	0.0052701
hsa-miR-186	264	0.00071	0.0056173
hsa-miR-18A	288	0.000727	0.0056789
hsa-miR-499-5P	100	0.000754	0.0058187
hsa-miR-128	161	0.000778	0.0058667
hsa-miR-130B	412	0.000782	0.0058667
hsa-miR-369-3P	152	0.000788	0.0058667
hsa-miR-625	110	0.000815	0.0059388
hsa-miR-520C-3P	289	0.000817	0.0059388
hsa-miR-519E	114	0.000875	0.0061969
hsa-miR-544	93	0.000879	0.0061969
HSA-LET-7D	324	0.000887	0.0061969
hsa-miR-181C	179	0.000897	0.0061969
HSA-LET-7E	307	0.000902	0.0061969
hsa-miR-548A-5P	289	0.000929	0.0063092
hsa-miR-329	42	0.000965	0.0064874
hsa-miR-573	167	0.00102	0.00678
hsa-miR-548C-5P	280	0.001142	0.0075109
hsa-miR-190B	99	0.001179	0.0076281
hsa-miR-325	95	0.001184	0.0076281
hsa-miR-524-5P	219	0.00121	0.0077195
hsa-miR-29B	189	0.001254	0.0079148
hsa-miR-181D	192	0.001282	0.0080104
hsa-miR-200C	140	0.001323	0.0081857
hsa-miR-10B	79	0.001376	0.0084319
hsa-miR-136	126	0.001404	0.0085117
hsa-miR-196A	173	0.001423	0.0085117
hsa-miR-583	141	0.00143	0.0085117
hsa-miR-410	87	0.001454	0.0085718
hsa-miR-152	226	0.001514	0.0088457
hsa-miR-146A	94	0.001551	0.0089749
hsa-miR-222	173	0.001623	0.009304
hsa-miR-450B-5P	142	0.001695	0.0096312
hsa-miR-641	167	0.001748	0.0097814
hsa-miR-219-5P	48	0.001753	0.0097814
hsa-miR-567	74	0.001856	0.0101736
hsa-miR-146B-5P	91	0.001856	0.0101736

hsa-miR-200A	118	0.00194	0.0105396
hsa-miR-520D-3P	302	0.001956	0.0105396
hsa-miR-208A	50	0.002042	0.0107837
hsa-miR-1179	75	0.002045	0.0107837
hsa-miR-539	163	0.002062	0.0107837
hsa-miR-214	218	0.00207	0.0107837
hsa-miR-377	89	0.002178	0.0112273
hsa-miR-371-5P	57	0.002192	0.0112273
hsa-miR-503	270	0.002278	0.0115515
hsa-miR-588	28	0.002292	0.0115515
hsa-miR-582-5P	165	0.002397	0.0119837
hsa-miR-506	135	0.002418	0.0119837
hsa-miR-432	185	0.002435	0.0119837
hsa-miR-26B	213	0.002519	0.0122426
hsa-miR-370	49	0.00255	0.0122426
hsa-miR-302B	283	0.00256	0.0122426
HSA-LET-7F	284	0.002566	0.0122426
hsa-miR-141	143	0.00273	0.012927
hsa-miR-1299	94	0.002801	0.0131607
hsa-miR-19B	396	0.002914	0.0135911
hsa-miR-361-5P	102	0.003	0.013863
HSA-LET-7C	350	0.003017	0.013863
hsa-miR-432STAR	22	0.003042	0.013876
hsa-miR-1259	82	0.003185	0.0144246
hsa-miR-875-3P	102	0.003234	0.014464
hsa-miR-586	169	0.003248	0.014464
hsa-miR-188-3P	80	0.003264	0.014464
hsa-miR-589	31	0.003286	0.014464
hsa-miR-34B	162	0.003378	0.0147631
hsa-miR-548C-3P	271	0.00347	0.0150613
HSA-LET-7B	385	0.003522	0.01518
hsa-miR-548O	147	0.003559	0.0152355
hsa-miR-98	286	0.003605	0.0152587
hsa-miR-509-5P	70	0.003613	0.0152587
HSA-LET-7A	312	0.003761	0.0156561
hsa-miR-562	76	0.003781	0.0156561
hsa-miR-526A	88	0.003783	0.0156561
hsa-miR-376C	107	0.003889	0.0159902
hsa-miR-520B	251	0.003931	0.016056
hsa-miR-301B	363	0.003964	0.0160884
hsa-miR-27B	211	0.004015	0.0161898
hsa-miR-454	349	0.004092	0.0162412
hsa-miR-578	62	0.004105	0.0162412
hsa-miR-326	56	0.004106	0.0162412
hsa-miR-137	153	0.004215	0.0165683
hsa-miR-508-3P	111	0.004279	0.0167156
hsa-miR-130A	414	0.004311	0.016735
hsa-miR-548G	154	0.004361	0.0167771
HSA-LET-7G	296	0.004375	0.0167771
hsa-miR-297	98	0.004606	0.0175117
hsa-miR-23A	159	0.004623	0.0175117
hsa-miR-516A-3P	35	0.004701	0.0176979
hsa-miR-1	162	0.004786	0.0177639
hsa-miR-527	146	0.004791	0.0177639
hsa-miR-574-3P	13	0.004803	0.0177639
hsa-miR-548H	243	0.004934	0.0179581
hsa-miR-548K	157	0.004938	0.0179581
hsa-miR-302C	291	0.004962	0.0179581

hsa-miR-217	103	0.004971	0.0179581
hsa-miR-452	152	0.005375	0.019305
hsa-miR-218	117	0.005536	0.0197712
hsa-miR-431	47	0.005599	0.0198842
HSA-LET-7I	313	0.005665	0.0200032
hsa-miR-660	44	0.005796	0.0203526
hsa-miR-1304	72	0.005832	0.0203621
hsa-miR-590-3P	299	0.006075	0.0210691
hsa-miR-106B	473	0.006102	0.0210691
hsa-miR-448	185	0.006205	0.0211448
hsa-miR-518A-5P	142	0.006218	0.0211448
hsa-miR-30ASTAR	76	0.006225	0.0211448
hsa-miR-1228	21	0.006349	0.0214505
hsa-miR-323-3P	116	0.006458	0.0216988
hsa-miR-1265	80	0.006651	0.0222293
hsa-miR-519D	393	0.006837	0.0227287
hsa-miR-135B	134	0.006958	0.0230088
hsa-miR-27A	198	0.007499	0.0245906
hsa-miR-148A	198	0.007515	0.0245906
hsa-miR-1279	49	0.007685	0.0250149
hsa-miR-33A	178	0.007754	0.0250444
hsa-miR-501-5P	96	0.007833	0.0250444
hsa-miR-545	182	0.007834	0.0250444
hsa-miR-362-3P	46	0.007854	0.0250444
hsa-miR-1252	219	0.007971	0.0252879
hsa-miR-519A	353	0.008094	0.0255504
hsa-miR-491-3P	98	0.008178	0.0255811
hsa-miR-487A	43	0.008186	0.0255811
hsa-miR-148B	191	0.008713	0.027054
hsa-miR-23B	155	0.008744	0.027054
hsa-miR-382	169	0.008824	0.0271687
hsa-miR-202	261	0.009249	0.0282377
hsa-miR-553	39	0.009262	0.0282377
hsa-miR-26A	203	0.009496	0.0288114
hsa-miR-429	143	0.00968	0.0292261
hsa-miR-1290	67	0.009758	0.0293218
hsa-miR-1256	39	0.010064	0.029789
hsa-miR-203	199	0.010121	0.029789
hsa-miR-518D-5P	50	0.010124	0.029789
hsa-miR-520C-5P	50	0.010124	0.029789
hsa-miR-519B-3P	337	0.010159	0.029789
hsa-miR-220A	15	0.0102	0.029789
hsa-miR-1267	61	0.010282	0.0298898
hsa-miR-1262	62	0.010601	0.0306744
hsa-miR-488	126	0.010766	0.0310094
hsa-miR-147	93	0.010905	0.0312654
hsa-miR-320B	152	0.011132	0.0317705
hsa-miR-521	25	0.011496	0.0326593
hsa-miR-519C-3P	366	0.011652	0.0329518
hsa-miR-199A-5P	101	0.011831	0.0333068
hsa-miR-596	20	0.011993	0.0336117
hsa-miR-183	151	0.012069	0.0336749
hsa-miR-149	82	0.01256	0.0348877
hsa-miR-548B-3P	64	0.012765	0.0352385
hsa-miR-367	285	0.012799	0.0352385
hsa-miR-656	127	0.013068	0.0358227
hsa-miR-10A	78	0.01322	0.0360799
hsa-miR-219-2-3P	85	0.013564	0.0367913

hsa-miR-379	54	0.013598	0.0367913
hsa-miR-500	90	0.013755	0.0370547
hsa-miR-1271	98	0.014449	0.0387567
hsa-miR-552	46	0.014671	0.0391862
hsa-miR-224	100	0.01475	0.0392288
hsa-miR-34A	266	0.014865	0.0393674
hsa-miR-302A	291	0.014943	0.0394067
hsa-miR-569	69	0.015243	0.0400285
hsa-miR-199B-5P	122	0.015673	0.0409852
hsa-miR-515-5P	107	0.015997	0.041659
hsa-miR-422A	66	0.016142	0.0418213
hsa-miR-1278	58	0.016193	0.0418213
hsa-miR-942	74	0.016322	0.0419795
hsa-miR-1226	30	0.016946	0.0434063
hsa-miR-9	170	0.017111	0.0434866
hsa-miR-374B	153	0.017153	0.0434866
hsa-miR-215	35	0.017186	0.0434866
hsa-miR-507	97	0.017499	0.0437631
hsa-miR-675	23	0.017522	0.0437631
hsa-miR-1827	69	0.017543	0.0437631
hsa-miR-653	95	0.017575	0.0437631
hsa-miR-96	145	0.01774	0.0439969
hsa-miR-1246	60	0.017976	0.0444064
hsa-miR-302D	305	0.018167	0.0447018
hsa-miR-563	19	0.018296	0.0448434
hsa-miR-142-5P	313	0.018421	0.0449731
hsa-miR-188-5P	73	0.018721	0.0455281
hsa-miR-512-3P	147	0.019162	0.0462455
hsa-miR-330-5P	47	0.019164	0.0462455
hsa-miR-626	29	0.019298	0.0463888
hsa-miR-1248	103	0.019669	0.0470996
hsa-miR-509-3-5P	73	0.019949	0.0475887
hsa-miR-619	70	0.020176	0.0478382
hsa-miR-580	150	0.020327	0.0478382
hsa-miR-655	131	0.020338	0.0478382
hsa-miR-127-5P	63	0.02036	0.0478382
hsa-miR-1277	34	0.020695	0.048443
